# Characterization of the Mycovirome from the Plant-Pathogenic Fungus *Cercospora beticola*

**DOI:** 10.3390/v13101915

**Published:** 2021-09-24

**Authors:** Yingxi Li, Mengke Zhou, Yizhou Yang, Qi Liu, Zongying Zhang, Chenggui Han, Ying Wang

**Affiliations:** 1 State Key Laboratory for Agro-Biotechnology, Ministry of Agriculture and Rural Affairs Key Laboratory of Pest Monitoring and Green Management, College of Plant Protection, China Agricultural University, Beijing 100193, China; liyingxi1998@163.com (Y.L.); s20193192619@cau.edu.cn (M.Z.); liuqi0725@cau.edu.cn (Q.L.); zhangzongying@cau.edu.cn (Z.Z.); hanchenggui@cau.edu.cn (C.H.); 2 State Key Laboratory of Agro-Biotechnology, College of Biological Sciences, China Agricultural University, Beijing 100193, China; yangyzhou@cau.edu.cn

**Keywords:** *Cercospora beticola*, high-throughput sequencing, mycovirome, diversity, biocontrol

## Abstract

Cercospora leaf spot (CLS) caused by *Cercospora beticola* is a devastating foliar disease of sugar beet (*Beta vulgaris*), resulting in high yield losses worldwide. Mycoviruses are widespread fungi viruses and can be used as a potential biocontrol agent for fugal disease management. To determine the presence of mycoviruses in *C. beticola*, high-throughput sequencing analysis was used to determine the diversity of mycoviruses in 139 *C. beticola* isolates collected from major sugar beet production areas in China. The high-throughput sequencing reads were assembled and searched against the NCBI database using BLASTn and BLASTx. The results showed that the obtained 93 contigs were derived from eight novel mycoviruses, which were grouped into 3 distinct lineages, belonging to the families *Hypoviridae, Narnaviridae* and *Botourmiaviridae,* as well as some unclassified (−)ssRNA viruses in the order *Bunyavirales* and *Mononegavirales*. To the best of our knowledge, this is the first identification of highly diverse mycoviruses in *C. beticola.* The novel mycoviruses explored in this study will provide new viral materials to biocontrol Cercospora diseases. Future studies of these mycoviruses will aim to assess the roles of each mycovirus in biological function of *C. beticola* in the future.

## 1. Introduction

Cercospora leaf spot (CLS) is a destructive foliar disease of sugar beet caused by *Cercospora beticola,* responsible for severe yield losses in epidemic years worldwide [1]. As a well-known pathogen of sugar beet and most species of the *Beta* genus, *C. beticola* has also been reported as a pathogen of other members of the family *Chenopodiaceae, Acanthaceae, Apiaceae, Brassicaceae, Malvaceae, Plumbaginaceae,* and *Polygonaceae* [2]. Disease caused by *C. beticola* is very difficult to control, because cultural and chemical controls provide limited effect due to the increasing fungicide resistance of *C. beticola* [3,4,5]. Although elevated resistance has been reported in most employed fungicides [6,7,8], application of fungicides with diverse modes of action is still widely used to manage CLS disease. Mycoviruses, as a potential biocontrol agent, have received increasing attention due to their ability to reduce the virulence of host fungi. Therefore, using mycoviruses as a biocontrol agent is a potential way to reduce the economic impact on sugar beet and other crop species.

Mycoviruses are viruses that can infect and replicate in fungi [9]. With appearance of next-generation sequencing (NGS), many mycoviruses have been discovered. For example, Osaki et al. detected a dsRNA sample from a single strain of *Fusarium poae* by deep sequencing and obtained 14 known mycoviruses and two viral sequences as-yet assigned to species in the genera *Ophiovirus* and *Phlebovirus*, respectively [10]; Yao et al. used RNA-Seq and identified 10 mycoviruses in *F. sacchari* and *F. andiyazi* isolates [11]; Mu et al. obtained 57 mycoviruses from Australian *S. sclerotiorum* isolates and grouped them into 10 distinct lineages [9]; and Chiapello et al. presented an inventory of 283 new RNA viruses from *Plasmopara viticola* in Italy and Spain [12]. Overall, mycoviruses have been grouped into 19 families from the International Committee on the Taxonomy of Viruses (ICTV, https://talk.ictvonline.org/ accessed on 16 August 2021): *Chrysoviridae, Totiviridae, Partitiviridae, Megabirnaviridae, Quadriviridae, Reoviridae, Hypoviridae, Narnaviridae, Mitoviridae, Endornaviridae, Barnaviridae, Botourmiaviridae, Alphaflexiviridae, Gammaflexiviridae, Deltaflexiviridae, Mymonaviridae, Pseudoviridae, Metaviridae,* and *Genomoviridae*. Nonetheless, there are still many mycoviruses that remained unclassified. Most mycoviruses have little impact on their fungal hosts, while some mycoviruses do cause significant effects on host growth, development, reproduction, and virulence. For example, Cryphonectria hypovirus 1 (CHV1), the first discovered mycovirus, has been used to control chestnut blight [13]. However, compared with plant or animal viruses, mycoviruses lack an extracellular route for infection and have no movement protein for the intercellular transmission, leading to difficult biocontrol application of mycovirus [14]. Recently, a few mycoviruses have been found to be able to overcome the transmission barriers and make it possible to use mycoviruses in the biological control of fungal diseases. Alterneria alternaria hypovirus 1 (AaHV1) has been proven to compromise the virulence of *Botryosphaeria dothidea*, a fungal pathogen of apple white rot disease [15]. In addition, Sclerotinia sclerotiorum hypovirulence-associated DNA virus 1 (SsHADV-1), the first discovered DNA mycovirus, can suppress hypovirulence of *Sclerotinia. sclerotiorum* through the transmission of a mycophagous insect, *Lycoriella ingenua* [16,17]. SsHADV-1 can also be used as a “plant vaccine” to enhance the seed yield of rapeseed [18].

Mycoviruses are widespread fungi viruses. However, it remains elusive whether some mycoviruses can infect *C. beticola*. In this study, we used deep RNA sequencing technology and RT-PCR assays to identify mycoviruses in 139 isolates of *C. beticola* collected from major sugar beet-producing regions in China. We obtained 93 contigs derived from eight novel mycoviruses, which makes it possible to look for a potential biocontrol agent to control CLS worldwide.

## 2. Materials and Methods

### 2.1. Cercospora beticola Isolates and Growth Conditions

*C. beticola* isolates were collected from diseased beet leaves from Beijing, Heilongjiang province, the Inner Mongolia Autonomous Region, and the Xinjiang Uygur Autonomous Region in China. The isolation method of the strains was single spore isolation which was performed as described previously [4]. The germinated conidium was transferred to potato dextrose agar (PDA) medium and cultured in darkness at 25 °C for two weeks. The single spore-derived colony was the source for subsequent high-throughput sequencing analysis. In total, 139 *C. beticola* isolates (Supplementary Materials Table S1) were obtained and maintained on PDA throughout these studies.

### 2.2. Total RNA Extraction

A total of 0.2 g mycelium from each isolate was scratched with a blade and put into a 2 mL tube. Total RNA was extracted by thermal phenol method as follows: the mycelia were grounded into fine powder in liquid nitrogen by Vortex mixer (Vortex-Genie 2, 230V (Model G560E), Scientific industries, Inc.). A total of 350 μL 80 °C preheated water-saturated phenol and equal volume of RNA extraction buffer (100 mM Tris-HCL (pH 8.0), 0.1 M LiCl, 10 mM EDTA (pH 8.0), 1.0% SDS) was immediately added into the powdered tissue and vibrated on the vortex mixer for 20 s. After 5 min at room temperature, the thawed mixture was combined with 350 μL chloroform and shaken vigorously for 20 s, then allowed to stand for 5 min at room temperature. The resulting mixture was centrifuged at 13,000 g for 15 min and the supernatant was transferred to a new tube. RNA in the supernatant was precipitated by equal volume of 4 M lithium chloride. The precipitate was washed two times with chilled 75% ethanol. The total RNA was dissolved in a final volume of 40 μL of diethyl pyrocarbonate-treated water and measured by Nanodrop UV spectrophotometer.

The total RNA of *C. beticola* isolates with the same colony morphology (color and shape), collection site, or fungicide (difenoconazole or pyraclostrobin)-resistant isolates were mixed as one group. Library 01 included strains with normal phenotype collected from the Inner Mongolia Autonomous Region in 2019, library 02 included strains with normal phenotype collected from the Inner Mongolia Autonomous Region, Heilongjiang Province and the Xinjiang Uygur Autonomous Region in 2020, library 03 contained strains with fanned-out edges collected from the Inner Mongolia Autonomous Region, Heilongjiang Province and the Xinjiang Uygur Autonomous Region of China in 2020, library 04 were strains with red or yellow pigmentation collected from the Xinjiang Uygur Autonomous Region, Beijing and the Inner Mongolia Autonomous Region in 2020, library 05 included difenoconazole-resistant strains from the Xinjiang Uygur Autonomous Region and the Inner Mongolia Autonomous Region in 2020, library 06 were pyraclostrobin-resistant strains from the Inner Mongolia Autonomous Region in 2020. Library 07 and 08 were strains with irregular edge (detailed information and colony morphology of each group can be seen in Supplementary Materials Table S1 and Figure S1). Finally, 8 groups, each group with mixed total RNA from 9, 23, 20, 19, 24, 17, 15, and 12 strains, were used for sequence analysis.

### 2.3. High-Throughput Sequencing and Sequence Analysis

Transcriptome sequencing of samples was performed by Beijing Biamark Biotechnology Co., Ltd. Novaseq 6000 was used for high-throughput sequencing, and the sequencing read length was PE150 to obtain raw data. Clean data with high quality were obtained by filtering low quality (Q ≤ 19 bases accounted for more than 50% of the total bases), joint contamination (> 5 bp) and reads containing more than 5% N in the original data.

Virus identification referred to the process of Wu et al. [19,20]: the genome sequence of *C. beticola* was filtered out by Hisat2 (version 2.2.1), and then clean data without the host genome were assembled by Mega-Hit (v1.2.9) to obtain primary contigs. Subsequently, Cap3 (Version Date: 02/10/15) and CD-Hit-Est (version 4.8.1) were used for splicing primary contigs and clustering them with 95% homologous data, respectively. Finally, the contigs obtained were then subjected to BLAST against GenBank using BLASTn and BLASTx (the nucleotide sequences of the contigs were converted into amino acid sequences then a BLASTp search was run).

### 2.4. DNA Extraction

Fungal DNA was extracted by the CTAB method as previously described although slightly modified [21]. A total of 0.1 g mycelium from each isolate was scratched with a blade and put into a 2 mL centrifuge tube. The mycelia were ground into fine powder in liquid nitrogen by vortex mixer. A total of 600 μL DNA extraction buffer (50 mM Tris-HCl (pH 8.0); 0.7 M NaCl; 10 mM EDTA; 1% CTAB) was immediately added into the powdered tissue and mixed well. After incubation at 65 °C for 1 h, the mixture was combined with 600 μL chloroform/isopentyl alcohol (24:1) and shaken for 20 s, then allowed to stand for 5 min at room temperature. The resulting mixture was centrifuged at 13,000 g for 20 min and 540 μL supernatant was transferred to a new tube. DNA in the supernatant was precipitated by an equal volume of isopropyl alcohol. The precipitate was washed two times with chilled 75% ethanol. The DNA was dissolved in a final volume of 40 μL ddH_2_O.

### 2.5. Confirmation of the Putative Mycoviruses

To verify the presence of putative mycovirus in strains, assembled contigs that matched viral sequences were used to design detection primers (Supplementary Materials Table S2). The first cDNAs strand was synthesized using Moloney murine leukemia virus (M-MLV) transcriptase M531A (Promega Madison, WI USA). Viral sequences were detected both by RT-PCR and DNA PCR with corresponding primer pairs. Internal Transcribed Spacer (ITS) Region for each *C. beticola* strain was amplified with primers ITS1 and ITS4 (Table S2) as positive control. The specific PCR fragments were purified and sequenced by Tsingke Biotechnology Co., Ltd. 

### 2.6. Phylogenetic Analysis

The nucleotide sequences and translated amino acid sequences of contigs with high similarity to known viral nucleic acids and proteins in GenBank were used for phylogenetic analysis. Alignments were performed by MAFFT (v7.037b) [22] and phylogenetic trees were constructed by the maximum likelihood method with a bootstrap value of 1000 replicates through MEGA-X [23]. The JTT model was selected, and the coding parameters were added to replace type selection 4. The initial tree BIONJ was improved to NNI. For some contigs that were incomplete and could not be used for phylogenetic analysis, the relationships were judged based on the result of the BLASTp. Viruses and accession numbers of viral gene(s) which were selected to perform phylogenetic analysis are listed (Supplementary Materials Table S3).

## 3. Results and Discussion

### 3.1. Mycoviruses Identification from C. beticola

**Figure 1:**
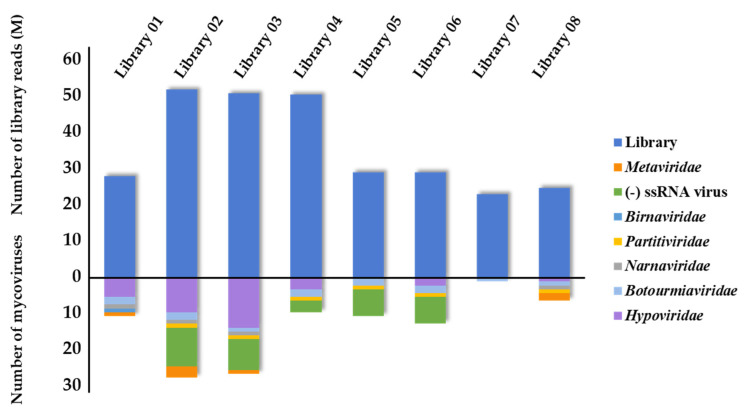
The distribution and diversity of virus in *C. beticola* transcriptomes. The top graph describes the number of reads in each library and the full names of each library has been shown on the top of the bar (dodger blue). The bottom graph describes a summary of the classification of virus species found in this study. The viral families of mycoviruses were indicated by bars with different colors: *Metaviridae*, (−)ssRNA virus, *Birnaviridae*, *Partitiviridae*, *Narnaviridae*, *Botourmiaviridae*, *Hypoviridae*.

Fungal viruses have been reported in several phytopathogenic fungi, such as *S. sclerotiorum*, *F. oxysporum*, *B. cinerea*, etc. [24,25,26]. Here, we first showed the presence of various mycoviruses in *C. beticola*. In this study, 8 RNA sequencing libraries were generated, sequenced to a considerable depth and assembled de novo. The library size of each sample ranged from 2.2–4.9 × 10^7^ reads (Figure 1). Overall, 2.7 × 10^8^ reads were generated. After removal of the host genome, a total number of 458,868 primary contigs were assembled by Mega-Hit and 31,416 final contigs were assembled by Mega-Hit and Cap3 were obtained. As a result of sequence analysis, 99 contigs derived from mycoviruses were obtained and the sequences of all the contigs were listed in Supplementary Materials Table S4. These contigs were assigned to 11 putative novel mycoviruses in five different viral families, including *Partitiviridae, Hypoviridae, Narnaviridae, Botourmiaviridae,* and *Metaviridae,* as well as some unclassified (−)ssRNA viruses (Figure 1). Since some putative mycoviruses contained multiple contigs, we first chose the longest contig as the representative one. Then the alignment of nucleotide or amino acid sequence between the representative and other sequences were carried out and the results were shown in Supplementary Materials Table S5. For example, 33 contigs were all assigned to the virus of the genus *Hypovirus* in Family *Hypoviridae*, and the polyprotein of contig1357 had 86.53%−99.79% sequence identity with other contigs at amino acid level. The result suggested that these contigs could be assigned to one virus although variation in the nucleotide sequence existed in different *C. beticola* strains. Furthermore, contig1357 with the longest length could be selected as representative for further analysis. For each putative novel mycoviruses and their representative contig, provisional names, sequence information and best-matched viruses were listed (Table 1 and Supplementary Materials Table S6).

**Figure 2:**
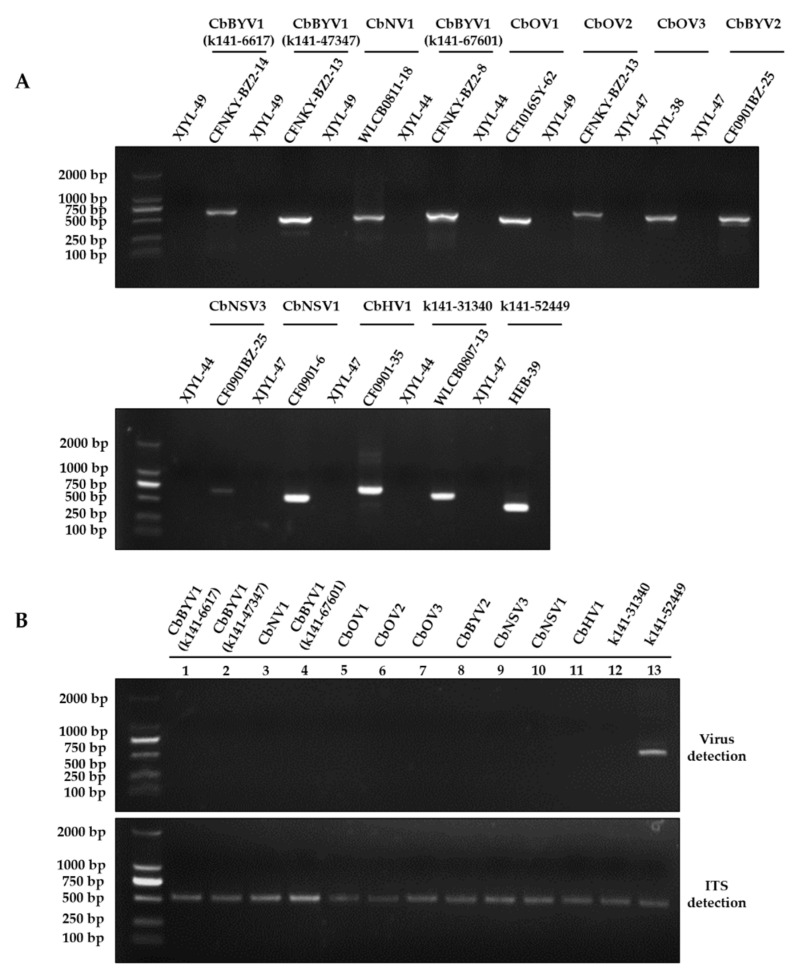
PCR confirmation of mycovirus contigs in *C. beticola* strains. (**A**) RT-PCR confirmation of mycovirus contigs in *C. beticola* strains. The viral primers were designed according to the contig sequences, and primers used are listed in Table S2. (**B**) DNA PCR amplification of each putative mycovirus contigs in *C. beticola*. Each line represented the specific strain of *C. beticola* contained the putative mycovirus. Line 1–13: CFNKY-BZ2-14, CFNKY-BZ2-13, WLCB0811-18, CFNKY-BZ2-8, CF1016SY-62, CFNKY-BZ2-13, XJYL-38, CF0901BZ-25, CF0901BZ-25, CF0901-6, CF0901-35, WLCB0807-13 and HEB-39. k141-52449 (related to partitiviruses) was amplified in strain HEB-39 which indicated the sequence had endogenized in genome of *C. beticola*. PCR amplification of TIS region with primers ITS1 and ITS4 were used as positive control for DNA quality.

First, the existence of these putative mycoviruses in specific strains of *C. beticola* was confirmed by RT-PCR with corresponding primers (Figure 2A). In addition, DNA PCR for *C. beticola* strains containing the putative mycovirus was also performed to verified whether the putative mycoviruses were authentic viruses or endogenous sequences (Figure 2B). As expected, 12 out of 13 putative mycoviruses were not amplified by direct DNA PCR (Figure 2B), demonstrating that these sequences are derived from mycoviruses. However, one putative dsRNA virus (k141-52449) which was closely related to the members of the family *Partitiviridae* was amplified by direct PCR (Figure 2B), suggesting that k141-52449 was an endogenous sequence of *C. beticola* genome rather than a virus sequence. Hence, k141-52449 was excluded from further analysis. In general, 54.84%, 37.63%, and 7.53% of the obtained putative virus harbors (+)ssRNA, (−)ssRNA, and ssRNA-RT genomes, respectively. In the five (+)ssRNA virus contigs, three contigs ((contig965, contig1024 and k141-5161) were from ourmia-like viruses, one contig (contig1357) was related to *Hypovirus*, one contig (k141-72534) was from a virus member of *Narnavirus*. The ssRNA-RT virus (k141-31340) genome was most similar to viruses in *Metaviridae.* There were some additional unclassified (−)ssRNA mycoviruses (contig154, k141-63059, k141-3378, k141-6617, k141-67601, k141-47347, and k141-54286) in *Bunyavirales* and *Mononegavirales*.

### 3.2. Sequences Related to (+)ssRNA Viruses

#### 3.2.1. One Predicted Novel Virus in Family Hypoviridae

**Figure 3:**
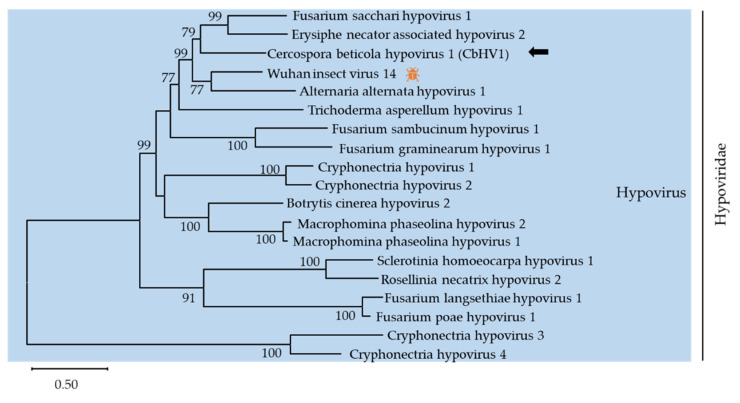
Unrooted phylogenetic tree reconstructed from the alignment of the amino acid sequences of the RdRp of *Hypovirus* representative members. The insect pattern (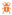) represents viruses which can infect insects. Virus identified in this work is indicated by black arrows.

The family *Hypoviridae*, containing only one genus *Hypovirus*, typically has (+)ssRNA genomes of 9.1–12.7 kb encoding one or two proteins [30]. Here, contig 1357 (accession number MZ546195) showed similarity to members of the family *Hypoviridae*. Contig 1357 was 12624 nt long and contained one large complete ORF that was predicted to encode a putative polyprotein with 3778 amino acids. The polyprotein had two conserved domains, DUF3525 (PF12039) and DEXDc (SM000487). A phylogenic analysis was conducted using the alignment of RdRp amino acid sequences of contig 1357 with representative members of *Hypovirus* (Figure 3). Based on BLASTp analysis, the putative protein showed 54%, 51.7%, and 40.8% identity with the polyprotein of Wuhan insect virus 14 (WIV14), Fusarium sacchari hypovirus 1 (FsHV1) and Erysiphe necator associated hypovirus 2, respectively. According to the demarcation criterion for the genus *Hypovirus* from the ICTV web site, contig 1357 represented a novel hypovirus named Cercospora beticola hypovirus 1 (CbHV1), tentatively.

So far, there are 4 species approved by the ICTV, Cryphonectria hypovirus 1-4 (CHV1-4), and many other hypoviruses reported in the GenBank database. Some hypoviruses have been reported to induce hypovirulence to their host fungi and are considered to be a potential biocontrol resource in plant disease management [15,31]. CHV1 has been successfully applied to chestnut blight control in Europe through altering fungal morphology and reducing virulence on chestnut trees [32]. Based on the CHV1− *Cryphonectria parasitica* pathosystem, many molecular mechanisms of mycovirology, including replication, pathogenicity, and RNA silencing-associated host immunity, have been extensively revealed [33,34]. Recently, a novel hypovirus named Alternaria alternata hypovirus 1 (AaHV1) isolated from *Alternaria alternata* f. sp. *mali* has been identified. Pathogenicity tests suggest that AaHV1 is responsible for the slow growth and hypovirulence of the host, which can be useful for the biocontrol of fungal diseases [15]. Thus, further studies on the molecular characterization and the pathogenicity of CbHV1 make it possible to be used as a biocontrol agent for fungal crop disease management.

#### 3.2.2. One Strain of Previously Reported Narnaviruses

*Narnaviridae* is a family of non-encapsidated (+)ssRNA of 2300–2900 nucleotides that encode a single protein of 80–140 kDa with amino acid sequence motifs characteristic of RdRp [35]. *Narnavirus* is a simple (+)ssRNA virus, since they only encode a single protein, RdRp, without capsid protein or extracellular transmission [36]. They form a ribonucleotide complex with RdRp in a 1:1 stoichiometry and reach a high copy number when under stress conditions, such as nitrogen starvation [37]. There is no apparent phenotype caused by narnaviruses in their host. In addition, due to the difficulty of generating virus-free strains and the fact that there is a complex and highly diverse viral pool in their fungal hosts, it remains difficult to study narnaviruses [38,39,40].

**Figure 4:**
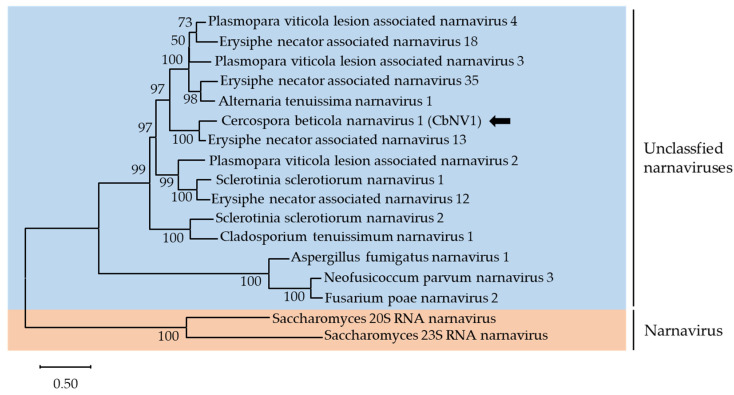
Unrooted phylogenetic tree reconstructed from the alignment of the amino acid sequences of the RdRp of *Narnavirus* representative members and other narnaviruses. Only bootstrap values above 50% are indicated. Virus identified in this work is indicated by black arrows.

According to the latest demarcation criterion from the ICTV, there is only one genus, *Narnavirus*, containing two approved species, *Saccharomyces 20S RNA narnavirus* (ScNV-20S) and *Saccharomyces 23S RNA narnavirus* (ScNV-23S). However, there are 130 tentative narnaviruses reported in the GenBank database. Some contigs have been reported to have multi-segmented genomes, which will be classified into different taxa [41,42,43,44]. In this study, contig k141-72534 (accession number MZ546196) contained a complete ORF encoding a putative protein of 805 amino acid. According to the sequence and phylogenetic analysis of conserved RdRp domains of narnaviruses, k141-72534 shared 77.1% amino acid identity with Erysiphe necator associated narnavirus 13 (EnNV13) and clustered together into a single branch with EnNV13 (Figure 4). The species demarcation criteria for *Narnavirus* are generally less than 50% identity at protein level. Thus, it is likely that k141-72534 is an isolate of EnNV13. We named Cercospora beticola narnavirus 1 (CbNV1) tentatively. After new taxonomic groups are established, CbNV1 will be classified into an exact taxonomic group.

#### 3.2.3. Three Predicted Novel Viruses in Family Botourmiaviridae

*Botourmiaviridae* is a new family approved by the ICTV, containing six genera: *Ourmiavirus*, *Botoulivirus*, *Magoulivirus*, *Penoulivirus, Rhizoulivirus* and *Scleroulivirus* [45,46]. In addition, numerous ourmia-like mycoviruses have been reported and clustered into an unclassified clade which do not belong to five mycovirus genera. In this work, three contigs, contig 965 (accession number MZ568927), contig 1024 (accession number MZ568928), and k141-5161 (accession number MZ568929), showed similarity to members of the family *Botourmiaviridae*. Contig 965 was 2273 nt long and contained one complete ORF encoding a putative protein of 617 amino acid. BLASTp analysis showed that this putative protein was most similar to the RdRp of Erysiphe necator associated ourmia-like virus 10, a member of the *Ourmiavirus* genus, with 68.1% identity. Contig 1024 was 1440 nt and contained one complete ORF, which encode a putative protein of 383 amino acid. The predicted amino acid sequence of this protein was similar to the RdRp of Plasmopara viticola lesion-associated ourmia-like virus 42 with 82% identity. k141-5161 was 2108 nt and the amino acid sequence was most similar to the RdRp of soybean thrips ourmia-like virus 1 (STOLV1) with 82.8% identity. The species demarcation criteria for the four genera in *Botourmiaviridae* is less than 90% amino acid identity in the RdRp or below 70% amino acid identity in CP. Therefore, contig 965, contig 1024, and k141-5161 represented three novel botourmiaviruses that we named Cercospora beticola ourmia-like virus 1 (CbOV1), Cercospora beticola ourmia-like virus 2 (CbOV2), and Cercospora beticola ourmia-like virus 3 (CbOV3), respectively.

**Figure 5:**
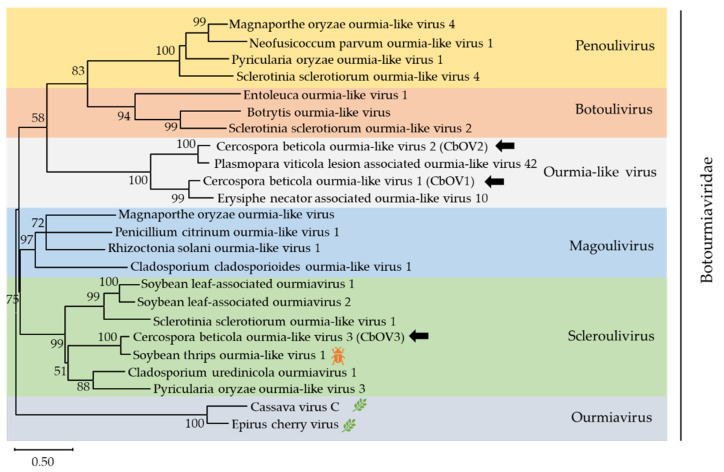
Unrooted phylogenetic tree reconstructed from the alignment of the amino acid sequences of the RdRp of *Botourmiaviridae* representative members. The insect pattern (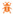) represents viruses which can infect insects, and the leaf pattern (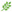) represents viruses which can infect plants. Only bootstrap values above 50% are indicated. Viruses identified in this work are indicated by black arrows.

To elucidate the evolutionary history of CbOV1-3, a phylogenetic analysis was conducted using the conserved RdRp domain encoded by CbOV1-3 and other selected viral sequences, including members of *Penoulivirus, Botoulivirus, Scleroulivirus,* *Magoulivirus*, and *Ourmiavirus* (Figure 5). The phylogenetic tree revealed that CbOV1 and CbOV2 were grouped with the ourmia-like viruses, suggesting that these ourmia-like viruses might represent a separate taxonomical group in the family *Botourmiaviridae*, and CbOV3 were in the group of *Scleroulivirus*.

In recent years, many ourmia-like viruses were found in plant-pathogenic fungi [47,48,49]. For example, the synthesized RNA of Sclerotinia sclerotiorum ourmia-like virus 4 (SsOLV4) can replicate in and transfer among strains of *S. sclerotiorum* via hyphal anastomosis, suggesting that a single RNA encoding an RdRp is sufficient for replication, infection, and transmission of ourmia-like viruses in fungi [47]. These phenomena indicate that ourmia-like viruses might have originated from *Ourmiavirus* by gene-loss events during their adaption to the fungal host [48,50]. Consequently, increasingly more ourmia-like viruses identified can help us have a better understanding of evolution and taxonomy of mycovirus.

### 3.3. Sequences Related to (−)ssRNA Viruses

The existence of (−)ssRNA mycoviruses were first identified in 2013 [51]. Many mycoviruses belonging to the order *Mononegavirales* were first reported [52,53,54]. Subsequently, segmented (−)ssRNA mycoviruses within *Bunyavirales* were reported in 2019 [55]. We identified seven (−)ssRNA viruses in this study. Six contigs were related to members of unclassified families in the order *Bunyavirales*, one contig was related to viruses in the order *Mononegavirales*.

The order Bunyavirales is one of the largest groups of segmented (−)ssRNA viruses. The order includes 12 families: Arenaviridae, Cruliviridae, Fimoviridae, Hantaviridae, Leishbuviridae, Mypoviridae, Nairoviridae, Peribunyaviridae, Phasmaviridae, Phenuiviridae, Tospoviridae, and Wupedeviridae, and numerous unclassified Bunyavirales. Lentinula edodes negative-strand RNA virus 2 is the first segmented (−)ssRNA virus known to infect fungi [55]. Recently, several segmented (−)ssRNA mycoviruses were identified and three new fungal viral families, Alphamycobunyaviridae, Betamycobunyaviridae, and Gammamycobunyaviridae, were proposed. The RNA genomes of Bunyavirales usually contain three segments (Large/L, Medium/M and Small/S segments, respectively) with conserved terminal sequences [56]. In the present study, viral sequences of k141-6617 (4197 nt), k141-67601 (1854 nt), k141-47347 (855 nt), k141-63059 (6457 nt), contig154 (6604 nt), and k141-54286 (566 nt) showed similarity with the members of Bunyavirales. It is worth noting that k141-6617, k141-67601, and k141-47347 have 69.4%, 44.5%, and 69.9% amino acid identity with the RdRp, NS, and NP of Plasmopara viticola lesion-associated mycobunyavirales-like virus 4, respectively. Thus, k141-6617, k141-67601, and k141-47347 represented a common bunya-like virus with different RNA segments and we named Cercospora beticola associated mycobunyavirales-like virus 1 (CbBYV1). k141-63059, encoding a putative protein of 2124 amino acid, has 63.2% identity the RdRp of Plasmopara viticola lesion-associated mycobunyavirales-like virus 10. Therefore, we named Cercospora beticola associated mycobunyavirales-like virus 2 (CbBYV2), representing a new bunya-like virus. Contig 154 had a complete ORF encoding a putative RdRp which shared 57.7% identity with viruses of Aspergillus fumigatus negative-stranded RNA virus 1. Thus, Contig 154 represented a new bunya-like virus and we named Cercospora beticola negative-stranded virus 1 (CbNSV1). The predicted protein of k141-52486 shared 43.1% amino acid identity with NS1 of Penicillium discovirus, suggesting that k141-52486 probably is a new bunya-like virus. Due to the absence of complete genome or the RdRp encoding sequence, further study is needed to confirm whether it represents a new bunya-like virus.

The order *Mononegavirales* includes 11 families and some unclassified viruses, and *Mymonaviridae* is the only classified viral family related to mycoviruses in the order *Mononegavirales* [57,58]. Here, we found that one contig has similarity with (−)ssRNA viruses in the order *Mononegavirales*. k141-3378 (accession number MZ599588) has 6191 nt and contains a complete ORF, which share 62.8% identity with the RdRp of Plasmopara viticola lesion-associated mononegaambi virus 5, representing a new mononega-like virus named Cercospora beticola negative-stranded virus 3 (CbNSV3). Viruses in the order *Mononegavirales* are mostly monopartite with multiple ORFs. A few viruses in the *Mononegavirales* have been reported, such as Sclerotinia sclerotiorum negative-stranded RNA virus 1 (SsNSRV-1) and Fusarium graminearum negative-stranded RNA virus 1 (FgNSRV-1) [53,54]. The infection of SsNSRV-1 leads to attenuated symptoms of the fungal host, including defective growth rate, abnormal colonial morphology, curled hyphal tips, and hypovirulence [53]. Later, it has also been proved that the integrity of SsNSRV-1 genome may be necessary to protect viral mRNA from splicing and inactivation by the host fungi [59]. However, to date there are still many unknown (−)ssRNA viruses and the properties of most reported (−)ssRNA viruses remains unknown.

**Figure 6:**
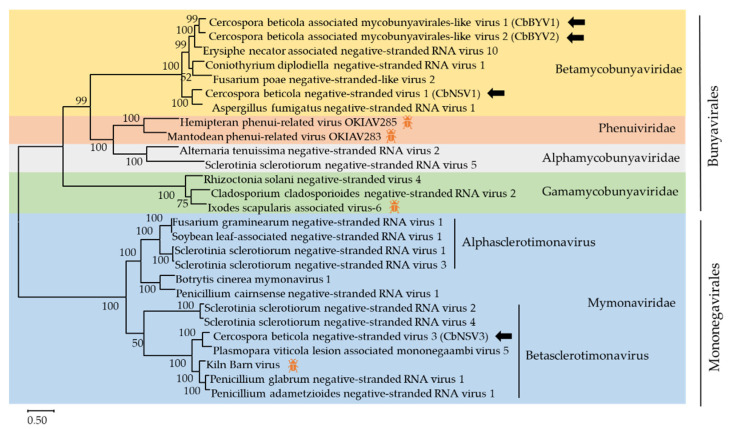
Unrooted phylogenetic tree reconstructed from the alignment of the amino acid sequences of the RdRp of *Bunyavirales* and *Monoegavirales* representative members. The insect pattern (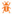) represents viruses which can infect insects. Only bootstrap values above 50% are indicated. Viruses identified in this work are indicated by black arrows.

A phylogenic analysis based on multiple alignments of the RdRp amino acid sequences of four (−)ssRNA viruses and other members in the order *Bunyavirales* and *Mononegavirales* was conducted (Figure 6). In phylogenic analysis, CbNSV1, CbBYV1 (k141-6617), and CbBYV2 clustered together with members in *Betamycobunyaviridae*, a new proposed family in *Bunyavirales* [46]. CbNSV3 grouped with members of the newly proposed genus *Betasclerotimonavirus* in family *Mymonaviridae* [46]. CbBYV1 (k141-67601 and k141-47347) were not included in the phylogenetic tree, because it did not encode a conserved RdRp domain.

### 3.4. Sequence Related to ssRNA-RT Viruses

*Metaviridae* (TY3/Gypsy) is a family of retrotransposons and reverse-transcribing viruses with long terminal repeats (LTRs) that are widely distributed in eukaryotes [60]. There are two genera in this family: *Metavirus* and *Errantivirus*. The RNA genomes of *Metaviridae* are two of the same copies of linear positive-stranded RNA, encoding two polyproteins, Gag and Pol. Some metaviruses, such as Cladosporium fulvum T-1 virus [29], encode an Env protein displaying characteristics of typical transmembrane (TM) and surface (SU) proteins. In this study, k141-31340 has 1247 nt and the predicted amino acid sequence of putative protein was similar to env homologue of Cladosporium fulvum T-1 virus with 64.3% identity. The species demarcation criteria set by the ICTV for *Metavirus* is less than 50% identity in their Gag proteins. However, k141-31340 only carries an ORF for a potential *env*-like gene in this study, which cannot be used for demarcation. Thus, we preliminarily supposed that k141-31340 might be a new metavirus. Due to a lack of a *gag* homologue gene, further study is needed to confirm whether k141-31340 represented a new metavirus.

Ty3/Gypsy elements represent a major class of LTR retrotransposons. As a representative of Ty3/Gypsy group, Saccharomyces cerevisiae Ty3 virus has been extensively characterized at molecular level and studied as a model of targeted integration. The study of Ty3 provide us a relatively comprehensive, albeit incomplete picture of the ongoing remodeling of a eukaryotic genome by an LTR retrotransposon [61]. Although several related viruses have been discovered, most of them are not formally classified to update and revise classification of metavirids. Therefore, further studies are needed to understand *Metaviridae* and retrotransposon.

## 4. Conclusions

Next-generation sequence (NGS) technologies have been widely used for detection and discovery of both known and novel viruses in animals, plants, and fungi [62,63,64]. Here, the sequencing results reveal that mycoviruses are widespread in *C. beticola* in China and some of them are likely to offer significant potential for mobile genetic elements (MGEs) in *C. beticola* and innovative biocontrol of CLS. In the future, characterization and biological function of these mycoviruses will facilitate biocontrol of CLS and expand our understanding of the diversity, ecology, evolution, and taxonomy of mycoviruses.

## Figures and Tables

**Table 1:** Assembled sequences with similarity to previously reported viruses.

Number	Genome Type ^1^	Family/Genus	Contig Number	Genbank Accession Number	Contig Length	Name of Putative Viruses	Best Match	aa Identity (%)	Query Coverage (%)	Reference
1	(+)ssRNA	*Hypoviridae*	contig1357	MZ546195	12624	Cercospora beticola hypovirus 1 (CbHV1)	polyprotein [Wuhan insect virus 14]	54.0	81	[27]
2	(+)ssRNA	*Narnaviridae*	k141-72534	MZ546196	2487	Cercospora beticola narnavirus 1 (CbNV1)	RdRp [Erysiphe necator associated narnavirus 13]	75.2	98	Unpublished
3	(+)ssRNA	*Botourmiaviridae*	contig965	MZ568927	2273	Cercospora beticola ourmia-like virus 1 (CbOV1)	RdRp [Erysiphe necator associated ourmia-like virus 10]	68.6	76	Unpublished
4	(+)ssRNA	*Botourmiaviridae*	contig1024	MZ568928	1440	Cercospora beticola ourmia-like virus 2 (CbOV2)	RdRp [Plasmopara viticola lesion-associated ourmia-like virus 42]	81.2	86	[12]
5	(+)ssRNA	*Botourmiaviridae*	k141-5161	MZ568929	2108	Cercospora beticola ourmia-like virus 3 (CbOV3)	RdRp [Soybean thrips ourmia-like virus 1]	83.4	82	[28]
6	(−)ssRNA	*-*	contig154	MZ599586	6604	Cercospora beticola negative-stranded virus 1 (CbNSV1)	RdRp [Aspergillus fumigatus negative-stranded RNA virus 1]	57.7	96	Unpublished
7	(−)ssRNA	*-*	k141-54286	MZ599593	566	-	NS1 [Penicillium discovirus]	49.2	63	Unpublished
8	(−)ssRNA	*-*	k141-3378	MZ599588	6191	Cercospora beticola negative-stranded virus 3 (CbNSV3)	RdRp [Plasmopara viticola lesion-associated mononegaambi virus 5]	62.8	94	[12]
9	(−)ssRNA	*-*	k141-6617	MZ599589	4197	Cercospora beticola associated mycobunyavirales-like virus 1 segment L (CbBYV1)	RdRp [Plasmopara viticola lesion-associated mycobunyavirales-like virus 4]	69.4	26	[12]
10	(−)ssRNA	*-*	k141-67601	MZ599592	1854	Cercospora beticola associated mycobunyavirales-like virus 1 segment S (CbBYV1)	NS [Plasmopara viticola lesion-associated mycobunyavirales-like virus 4]	46.0	76	[12]
11	(−)ssRNA	*-*	k141-47347	MZ599591	855	Cercospora beticola associated mycobunyavirales-like virus 1 segment S (CbBYV1)	NP [Plasmopara viticola lesion-associated mycobunyavirales-like virus 4]	65.9	92	[12]
12	(−)ssRNA	*-*	k141-63059	MZ599587	6457	Cercospora beticola associated mycobunyavirales-like virus 2 (CbBYV2)	RdRp [Erysiphe necator associated negative-stranded RNA virus 10]	63.2	84	[12]
13	ssRNA-RT	*Metaviridae*	k141-31340	MZ599594	1247	-	env homologue [Cladosporium fulvum T-1 virus]	64.3	33	[29]

^1^ dsRNA: double-stranded RNA, (+)ssRNA: positive-sense single-stranded RNA, (−)ssRNA: negative-sense single-stranded RNA, ssRNA-RT: single-stranded RNA reverse-transcribing.

## Data Availability

The sequences reported in the present manuscript have been deposited in the GenBank database under accession numbers MZ523679, MZ546195-546196, MZ568927-568929, MZ599586-599594.

## Supplementary Materials

The following are available online at https://www.mdpi.com/article/10.3390/v13101915/s1, Figure S1: Phenotypes of *Cercospora beticola* isolates from different groups used for high-throughput sequencing. Table S1: *Cercospora beticola* isolates from China used in this study. Table S2: Primer pairs used to confirm the exitance of viral sequences in *Cercospora beticola*, Table S3: Viruses selected for phylogenetic analysis, Table S4: The sequences of 99 primary contigs assembled from clean data of 139 strains of *Cercospora beticola*, Table S5: Pairwise alignments of sequences assigned to the same virus. Table S6: Details of the 93 contigs obtained from 139 strains of *Cercospora beticola*.

## References

[1] Holtschulte B., Asher M.J.C., Holtschulte B., Molard M.R., Rosso F., Steinrucken G., Beckers R. (2000). Cercospora beticola-worldwide distribution and incidence. Cercospora Beticola Sacc. Biology, Agronomic Influence and Control Measures in Sugar Beet.

[2] Rangel L.I., Spanner R.E., Ebert M.K., Pethybridge S.J., Stukenbrock E.H., de Jonge R., Secor G.A., Bolton M.D. (2020). *Cercospora beticola*: The intoxicating lifestyle of the leaf spot pathogen of sugar beet. Mol. Plant Pathol..

[3] Giannopolitis C.N. (1978). Occurrence of strains of *Cercospora beticola* resistant to triphenyltin fungicides in Greece. Plant Dis. Report..

[4] Bolton M.D., Birla K., Rivera-Varas V., Rudolph K.D., Secor G.A. (2012). Characterization of *CbCyp51* from field isolates of *Cercospora beticola*. Phytopathology.

[5] Weiland J.J., Halloin J.M. (2001). Benzimidazole resistance in *Cercospora beticola* sampled from sugarbeet fields in Michigan, USA ^1^. Can. J. Plant Pathol..

[6] Trueman C.L., Hanson L.E., Somohano P., Rosenzweig N. (2017). First report of DMI-insensitive Cercospora beticola on sugar beet in Ontario, Canada. New Dis. Rep..

[7] Housni Z.E., Ezrari S., Tahiri A., Ouijja A., Lahlali R. (2018). First Report of Benzimidazole, DMI and QoI-insensitive *Cercospora beticola* in sugar beet in Morocco. New Dis. Rep..

[8] Hudec K., Mihók M., Roháčik T., Mišľan Ľ. (2020). Sensitivity of *Cercospora beticola* to fungicides in Slovakia. Acta Fytotech. Et Zootech..

[9] Mu F., Xie J., Cheng S., You M.P., Barbetti M.J., Jia J., Wang Q., Cheng J., Fu Y., Chen T. (2018). Virome characterization of a collection of *S. sclerotiorum* from Australia. Front. Microbiol..

[10] Osaki H., Sasaki A., Nomiyama K., Tomioka K. (2016). Multiple virus infection in a single strain of *Fusarium poae* shown by deep sequencing. Virus Genes.

[11] Yao Z., Zou C., Peng N., Zhu Y., Bao Y., Zhou Q., Wu Q., Chen B., Zhang M. (2020). Virome identification and characterization of *Fusarium sacchari* and *F. Andiyazi*: Causative agents of pokkah boeng disease in sugarcane. Front. Microbiol..

[12] Chiapello M., Rodríguez-Romero J., Ayllón M.A., Turina M. (2020). Analysis of the virome associated to grapevine downy mildew lesions reveals new mycovirus lineages. Virus Evol..

[13] Shapira R., Choi G.H., Nuss D.L. (1991). Virus-like genetic organization and expression strategy for a double-stranded RNA genetic element associated with biological control of chestnut blight. EMBO J..

[14] Son M., Yu J., Kim K.-H. (2015). Five questions about mycoviruses. PLoS Pathog..

[15] Li H., Bian R., Liu Q., Yang L., Pang T., Salaipeth L., Andika I.B., Kondo H., Sun L. (2019). Identification of a novel hypovirulence-inducing hypovirus from *Alternaria*
*Alternata*. Front. Microbiol..

[16] Yu X., Li B., Fu Y., Xie J., Cheng J., Ghabrial S.A., Li G., Yi X., Jiang D. (2013). Extracellular transmission of a DNA mycovirus and its use as a natural fungicide. Proc. Natl. Acad. Sci. USA.

[17] Liu S., Xie J., Cheng J., Li B., Chen T., Fu Y., Li G., Wang M., Jin H., Wan H. (2016). Fungal DNA virus infects a mycophagous insect and utilizes it as a transmission vector. Proc. Natl. Acad. Sci. USA.

[18] Zhang H., Xie J., Fu Y., Cheng J., Qu Z., Zhao Z., Cheng S., Chen T., Li B., Wang Q. (2020). A 2-Kb mycovirus converts a pathogenic fungus into a beneficial endophyte for brassica protection and yield enhancement. Mol. Plant.

[19] Wu Q., Luo Y., Lu R., Lau N., Lai E.C., Li W.-X., Ding S.-W. (2010). Virus discovery by deep sequencing and assembly of virus-derived small silencing RNAs. Proc. Natl. Acad. Sci. USA.

[20] Wu Q., Ding S.-W., Zhang Y., Zhu S. (2015). Identification of viruses and viroids by next-generation sequencing and homology-dependent and homology-independent algorithms. Annu. Rev. Phytopathol..

[21] Moretti M., Saracchi M., Farina G. (2004). Morphological, physiological and genetic diversity within a small population of *Cercospora Beticola* Sacc. Ann. Microbiol..

[22] Katoh K., Standley D.M. (2013). MAFFT multiple sequence alignment software version 7: Improvements in performance and usability. Mol. Biol. Evol..

[23] Kumar S., Stecher G., Li M., Knyaz C., Tamura K. (2018). MEGA X: Molecular evolutionary genetics analysis across computing platforms. Mol. Biol. Evol..

[24] Wang Q., Cheng S., Xiao X., Cheng J., Fu Y., Chen T., Jiang D., Xie J. (2019). Discovery of two mycoviruses by high-throughput sequencing and assembly of mycovirus-derived small silencing RNAs from a hypovirulent strain of *Sclerotinia sclerotiorum*. Front. Microbiol..

[25] Zhao Y., Zhang Y., Wan X., She Y., Li M., Xi H., Xie J., Wen C. (2020). A novel ourmia-like mycovirus confers hypovirulence-associated traits on *Fusarium oxysporum*. Front. Microbiol..

[26] Yu L., Sang W., Wu M.-D., Zhang J., Yang L., Zhou Y.-J., Chen W.-D., Li G.-Q. (2015). Novel hypovirulence-associated RNA mycovirus in the plant-pathogenic fungus *Botrytis cinerea*: Molecular and biological characterization. Appl. Environ. Microbiol..

[27] Shi M., Lin X.-D., Tian J.-H., Chen L.-J., Chen X., Li C.-X., Qin X.-C., Li J., Cao J.-P., Eden J.-S. (2016). Redefining the invertebrate RNA virosphere. Nature.

[28] Thekke-Veetil T., Lagos-Kutz D., McCoppin N.K., Hartman G.L., Ju H.-K., Lim H.-S., Domier L.L. (2020). Soybean thrips (*Thysanoptera*: *Thripidae*) harbor highly diverse populations of arthropod, fungal and plant viruses. Viruses.

[29] McHale M.T., Roberts I.N., Noble S.M., Beaumont C., Whitehead M.P., Seth D., Oliver R.P. (1992). *CfT-I*: An LTR-retrotransposon in *Cladosporium Fulvum*, a fungal pathogen of tomato. Mol. Genet. Genom..

[30] Suzuki N., Ghabrial S.A., Kim K.-H., Pearson M., Marzano S.-Y.L., Yaegashi H., Xie J., Guo L., Kondo H., Koloniuk I. (2018). ICTV virus taxonomy profile: *Hypoviridae*. J. Gen. Virol..

[31] Romon-Ochoa P., Gorton C., Lewis A., van der Linde S., Webber J., Pérez-Sierra A. (2020). Hypovirulent effect of the Cryphonectria hypovirus 1 in british isolates of *Cryphonectria parasitica*. Pest Manag. Sci..

[32] Bryner S.F., Rigling D., Brunner P.C. (2012). Invasion history and demographic pattern of Cryphonectria hypovirus 1 across European populations of the chestnut blight fungus. Ecol. Evol..

[33] Chiba S., Suzuki N. (2015). Highly activated RNA silencing via strong induction of dicer by one virus can interfere with the replication of an unrelated virus. Proc. Natl. Acad. Sci. USA.

[34] Eusebio-Cope A., Sun L., Tanaka T., Chiba S., Kasahara S., Suzuki N. (2015). The Chestnut blight fungus for studies on virus/host and virus/virus interactions: From a natural to a model host. Virology.

[35] García-Cuéllar M.P., Esteban R., Fujimura T. (1997). RNA-dependent RNA polymerase activity associated with the yeast viral P91/20S RNA ribonucleoprotein complex. RNA.

[36] Fujimura T., Esteban R. (2007). Interactions of the RNA polymerase with the viral genome at the 5′- and 3′-ends contribute to 20S RNA narnavirus persistence in yeast. J. Biol. Chem..

[37] Matsumoto Y., Fishel R., Wickner R.B. (1990). Circular single-stranded RNA replicon in *Saccharomyces Cerevisiae*. Proc. Natl. Acad. Sci. USA.

[38] Niu Y., Yuan Y., Mao J., Yang Z., Cao Q., Zhang T., Wang S., Liu D. (2018). Characterization of two novel mycoviruses from *Penicillium digitatum* and the related fungicide resistance analysis. Sci. Rep..

[39] Zoll J., Verweij P.E., Melchers W.J.G. (2018). Discovery and characterization of novel *Aspergillus fumigatus* mycoviruses. PLoS One.

[40] Cai G., Myers K., Fry W.E., Hillman B.I. (2012). A member of the virus family *Narnaviridae* from the plant pathogenic oomycete *Phytophthora infestans*. Arch. Virol..

[41] Chiba Y., Oiki S., Yaguchi T., Urayama S.-I., Hagiwara D. (2021). Discovery of Divided RdRp Sequences and a Hitherto Unknown Genomic Complexity in Fungal Viruses. Virus Evol..

[42] Sutela S., Forgia M., Vainio E.J., Chiapello M., Daghino S., Vallino M., Martino E., Girlanda M., Perotto S., Turina M. (2020). The virome from a collection of endomycorrhizal fungi reveals new viral taxa with unprecedented genome organization. Virus Evol..

[43] Jia J., Fu Y., Jiang D., Mu F., Cheng J., Lin Y., Li B., Marzano S.-Y.L., Xie J. (2021). Interannual dynamics, diversity and evolution of the virome in *Sclerotinia sclerotiorum* from a single crop field. Virus Evol..

[44] Ruiz-Padilla A., Rodríguez-Romero J., Gómez-Cid I., Pacifico D., Ayllón M.A. (2021). Novel mycoviruses discovered in the mycovirome of a necrotrophic fungus. mBio.

[45] Ayllón M.A., Turina M., Xie J., Nerva L., Marzano S.-Y.L., Donaire L., Jiang D., ICTV Report Consortium (2020). ICTV virus taxonomy profile: *Botourmiaviridae*. J. Gen. Virol..

[46] Nerva L., Turina M., Zanzotto A., Gardiman M., Gaiotti F., Gambino G., Chitarra W. (2019). Isolation, molecular characterization and virome analysis of culturable wood fungal endophytes in Esca symptomatic and asymptomatic grapevine plants. Environ. Microbiol..

[47] Wang Q., Mu F., Xie J., Cheng J., Fu Y., Jiang D. (2020). A single ssRNA segment encoding RdRp is sufficient for replication, infection, and transmission of ourmia-like virus in fungi. Front. Microbiol..

[48] Marzano S.-Y.L., Nelson B.D., Ajayi-Oyetunde O., Bradley C.A., Hughes T.J., Hartman G.L., Eastburn D.M., Domier L.L. (2016). Identification of diverse mycoviruses through metatranscriptomics characterization of the viromes of five major fungal plant pathogens. J. Virol..

[49] Liu Y., Zhang L., Esmael A., Duan J., Bian X., Jia J., Xie J., Cheng J., Fu Y., Jiang D. (2020). Four novel botourmiaviruses co-infecting an isolate of the rice blast fungus *Magnaporthe oryzae*. Viruses.

[50] Donaire L., Rozas J., Ayllón M.A. (2016). Molecular characterization of Botrytis ourmia-like virus, a mycovirus close to the plant pathogenic genus *Ourmiavirus*. Virology.

[51] Kondo H., Chiba S., Toyoda K., Suzuki N. (2013). Evidence for negative-strand RNA virus infection in fungi. Virology.

[52] Hao F., Wu M., Li G. (2018). Molecular Characterization and geographic distribution of a mymonavirus in the population of *Botrytis cinerea*. Viruses.

[53] Liu L., Xie J., Cheng J., Fu Y., Li G., Yi X., Jiang D. (2014). Fungal negative-stranded RNA virus that is related to bornaviruses and nyaviruses. Proc. Natl. Acad. Sci. USA.

[54] Wang L., He H., Wang S., Chen X., Qiu D., Kondo H., Guo L. (2018). Evidence for a novel negative-stranded RNA mycovirus isolated from the plant pathogenic fungus *Fusarium graminearum*. Virology.

[55] Lin Y.-H., Fujita M., Chiba S., Hyodo K., Andika I.B., Suzuki N., Kondo H. (2019). Two novel fungal negative-strand RNA viruses related to mymonaviruses and phenuiviruses in the shiitake mushroom (*Lentinula edodes*). Virology.

[56] King A.M.Q., Adams M.J., Carstens E.B., Lefkowitz E.J. (2012). Family-Bunyaviridae. Virus Taxonomy.

[57] Afonso C.L., Amarasinghe G.K., Bányai K., Bào Y., Basler C.F., Bavari S., Bejerman N., Blasdell K.R., Briand F.-X., Briese T. (2016). Taxonomy of the order *Mononegavirales*: Update 2016. Arch. Virol..

[58] Jiāng D.-H., Ayllón M.A., Marzano S.-Y.L., ICTV Report Consortium (2019). ICTV virus taxonomy profile: *Mymonaviridae*. J. Gen. Virol..

[59] Gao Z., Wu J., Jiang D., Xie J., Cheng J., Lin Y. (2020). ORF Ι of mycovirus SsNSRV-1 is associated with debilitating symptoms of *Sclerotinia sclerotiorum*. Viruses.

[60] Llorens C., Soriano B., Krupovic M., Ictv Report Consortium (2020). ICTV virus taxonomy profile: *Metaviridae*. J. Gen. Virol..

[61] Sandmeyer S., Patterson K., Bilanchone V. (2015). *Ty3*, a position-specific retrotransposon in budding yeast. Microbiol. Spectr..

[62] Abdoulaye A.H., Foda M.F., Kotta-Loizou I. (2019). Viruses infecting the plant pathogenic fungus *Rhizoctonia solani*. Viruses.

[63] Barba M., Czosnek H., Hadidi A. (2014). Historical perspective, development and applications of next-generation sequencing in plant virology. Viruses.

[64] Kwok K.T.T., Nieuwenhuijse D.F., Phan M.V.T., Koopmans M.P.G. (2020). Virus metagenomics in farm animals: A systematic review. Viruses.

