# Supplementary material for: Characterization of the Mycovirome from the Plant-Pathogenic Fungus Cercospora beticola
Source: Viruses. 2021 Sep 24;13(10):1915. doi: 10.3390/v13101915 (PMC8537984; doi:10.3390/v13101915)
Supplement: Supplementary file 1 [file viruses-13-01915-s001.zip › viruses-1368835-supplementary Figure S1.pdf]

Supplementary Figure S1

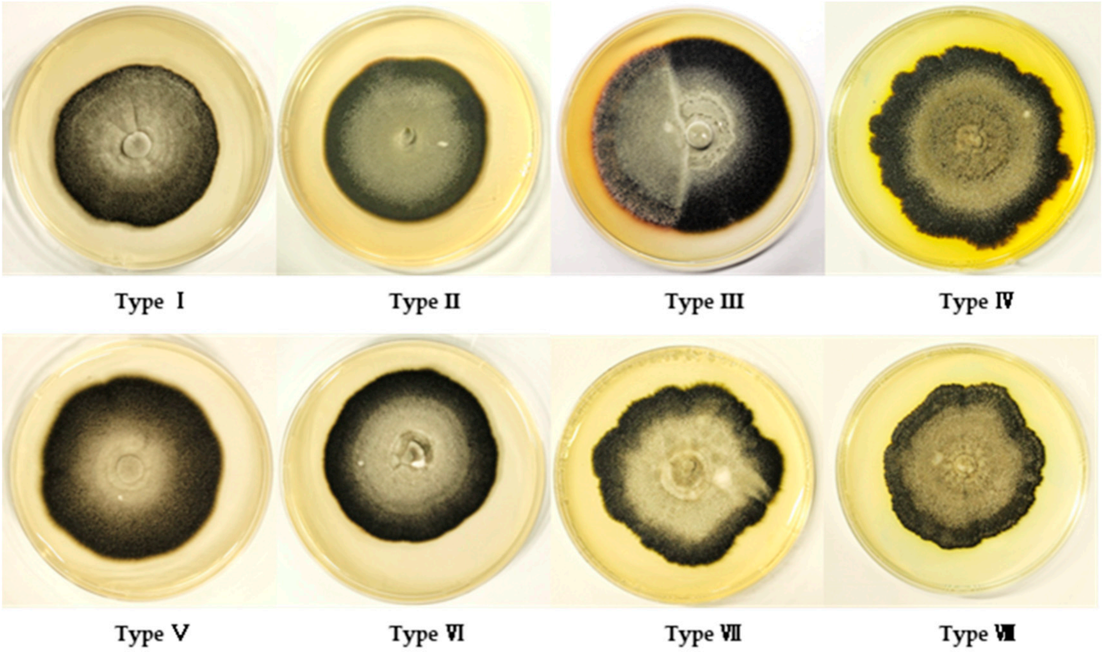

Figure S1. Phenotypes of *Cercospora beticola* isolates from different groups used for high-throughput sequencing. The isolates were grown on PAD for two weeks. Type I were isolates with normal phenotype collected from Inner Mongolia Autonomous Region in 2019. Type II were isolates with normal phenotype collected from Inner Mongolia Autonomous Region, Heilongjiang Province and Xinjiang Uygur Autonomous Region in 2020. Type III were isolates fanned-out edge collected from Inner Mongolia Autonomous Region, Heilongjiang Province and Xinjiang Uygur Autonomous Region of China in 2020. Type IV were isolates with red or yellow pigmentation collected from Xinjiang Uygur Autonomous Region, Beijing and Inner Mongolia Autonomous Region in 2020. Type V were difenoconazole -resistant isolates from Xinjiang Uygur Autonomous Region and Inner Mongolia Autonomous Region in 2020. Type VI were pyraclostrobin-resistant isolates from Inner Mongolia Autonomous Region in 2020. Type VII and Type VIII were isolates with irregular edge collected from Inner Mongolia Autonomous Region, Heilongjiang Province and Xinjiang Uygur Autonomous Region of China in 2020.
