# Supplementary material for: Characterization of the Mycovirome from the Plant-Pathogenic Fungus Cercospora beticola
Source: Viruses. 2021 Sep 24;13(10):1915. doi: 10.3390/v13101915 (PMC8537984; doi:10.3390/v13101915)
Supplement: Supplementary file 1 [file viruses-13-01915-s001.zip › viruses-1368835-supplementary Table S1.pdf]

Table S1. ***Cercospora beticola*** **isolates from China used in this study**

Sample	Code	Host	Date	Location	Description
1	66-ABT	*B. vulgaris*	2019	Inner Mongolia Autonomous Region	Isolates with normal phenotype collected from Inner Mongolia Autonomous Region in 2019 (Supplementary Materials Figure S1 Type I)
	65-ABT	*B. vulgaris*	2019		
	67-ABT	*B. vulgaris*	2019		
	YBS-7	*B. vulgaris*	2019		
	YBS-5	*B. vulgaris*	2019		
	61-SDY22	*B. vulgaris*	2019		
	43-H809	*B. vulgaris*	2019		
	40-H809	*B. vulgaris*	2019		
	55-H004	*B. vulgaris*	2019		
2	CF0901-7	*B. vulgaris*	2020	Inner Mongolia Autonomous Region	Colonies with normal phenotype. (Supplementary Materials Figure S1 Type II)
	CF0901-6	*B. vulgaris*	2020		
	CF0901-35	*B. vulgaris*	2020		
	CF0901-38	*B. vulgaris*	2020		
	CF0901-61	*B. vulgaris*	2020		
	CF0901-57	*B. vulgaris*	2020		
	CF0901-49	*B. vulgaris*	2020		
	CF0901-51	*B. vulgaris*	2020		
	CF0901-52	*B. vulgaris*	2020		
	XAM0819-1	*B. vulgaris*	2020		
	WLCB0811-19	*B. vulgaris*	2020		
	WLCB0807-1	*B. vulgaris*	2020		
	WLCB0807-13	*B. vulgaris*	2020		
	YA0807-18	*B. vulgaris*	2020	Heilongjiang Province	
	YA0807-16	*B. vulgaris*	2020		
	HEB-14	*B. vulgaris*	2020		
	HEB-39	*B. vulgaris*	2020		
	HEB-22	*B. vulgaris*	2020		
	YL1002-100	*B. vulgaris*	2020	Xinjiang Uygur Autonomous Region of China	
	YL1002-15	*B. vulgaris*	2020		
	YL1002-143	*B. vulgaris*	2020		
	YL1002-191	*B. vulgaris*	2020		
	XJYL-28	*B. vulgaris*	2020		
3	JN-38	*B. vulgaris*	2020	Inner Mongolia Autonomous Region	The edge of colonies is fanned out.<br>(Supplementary Materials Figure S1 Type III)
	CF0901-31	*B. vulgaris*	2020		
	CF1019SY-69	*B. vulgaris*	2020		
	CF1017SY-75	*B. vulgaris*	2020		
	CF1016SC-85	*B. vulgaris*	2020		
	CF1017SC-84	*B. vulgaris*	2020		
	CF1008-44	*B. vulgaris*	2020		

	WLCB0811-18	*B. vulgaris*	2020		
	WLCB0811-5	*B. vulgaris*	2020		
	YL1002-39	*B. vulgaris*	2020	Xinjiang Uygur Autonomous Region of China	
	YL1002-141	*B. vulgaris*	2020		
	YL1002-105	*B. vulgaris*	2020		
	YL1002-62	*B. vulgaris*	2020		
	XJYL-38	*B. vulgaris*	2020		
	YL1002-172	*B. vulgaris*	2020		
	YL1002-38	*B. vulgaris*	2020		
	YL1002-159	*B. vulgaris*	2020		
	YL1002-187	*B. vulgaris*	2020		
	HEB-12	*B. vulgaris*	2020	Heilongjiang Province	
	HEB-52	*B. vulgaris*	2020		
4	YL1002-77	*B. vulgaris*	2020	Xinjiang Uygur Autonomous Region of China	pigmentation (red) at the edge of colonies. (Supplementary Materials Figure S1 Type IV)
	XJYL-41	*B. vulgaris*	2020		
	SZDB-21	*B. vulgaris*	2020	Beijing	
	SZDB-3	*B. vulgaris*	2020		
	SZDB-13	*B. vulgaris*	2020		
	SZDB-10	*B. vulgaris*	2020		
	SZDB-24	*B. vulgaris*	2020		
	SZDB-1	*B. vulgaris*	2020		
	SZDB-29	*B. vulgaris*	2020		
	SZDB-26	*B. vulgaris*	2020		
	SZDB-19	*B. vulgaris*	2020		
	SZDB-7	*B. vulgaris*	2020		
	SZDB-18	*B. vulgaris*	2020		
	SZDB-6	*B. vulgaris*	2020		
	SZDB-17	*B. vulgaris*	2020		
	CF1018SY-74	*B. vulgaris*	2020	Inner Mongolia Autonomous Region	
	CF1019SY-99	*B. vulgaris*	2020		
	CF1020SY-98	*B. vulgaris*	2020		
	CF1022SY-32	*B. vulgaris*	2020		
5	CFNKY1006-BM2.5-15	*B. vulgaris*	2020	Inner Mongolia Autonomous Region	Difenoconazole-resistant isolates. (Supplementary Materials Figure S1 Type V)
	CFNKY1006-BM2.5-30	*B. vulgaris*	2020		
	CFNKY1006-BM2.5-31	*B. vulgaris*	2020		
	CFNKY1006-BM2.5-17	*B. vulgaris*	2020		
	CFNKY1006-BM2.5-56	*B. vulgaris*	2020		
	CFNKY1006-BM2.5-57	*B. vulgaris*	2020		
	CFNKY1006-BM2.5-58	*B. vulgaris*	2020		
	CFNKY1006-BM2.5-59	*B. vulgaris*	2020		

	CFNKY1006-BM2.5-60	*B. vulgaris*	2020		
	CFNKY1006-BM2.5-6	*B. vulgaris*	2020		
	CFNKY1006-BM2.5-4	*B. vulgaris*	2020		
	CFNKY1006-BM2.5-16	*B. vulgaris*	2020		
	CFNKY1006-BM2.5-18	*B. vulgaris*	2020		
	CFNKY1006-BM2.5-24	*B. vulgaris*	2020		
	CFNKY1006-BM2.5-25	*B. vulgaris*	2020		
	CFNKY1006-BM2.5-26	*B. vulgaris*	2020		
	CFNKY1006-BM2.5-43	*B. vulgaris*	2020		
	CFNKY-BM-36	*B. vulgaris*	2020		
	CFNKY-BM-8	*B. vulgaris*	2020		
	CFNKY-BM-35	*B. vulgaris*	2020		
	XJHC0925-BM2-11	*B. vulgaris*	2020	Xinjiang Uygur Autonomous Region of China	
	XJHC0925-BM2-13	*B. vulgaris*	2020		
	XJHC0925-BM2-20	*B. vulgaris*	2020		
	XJHC-BM2-18	*B. vulgaris*	2020		
6	CF0901BZ-38	*B. vulgaris*	2020	Inner Mongolia Autonomous Region	Pyraclostrobin-resistant isolates. (Supplementary Materials Figure S1 Type VI)
	CF0901BZ-25	*B. vulgaris*	2020		
	CF0901BZ-23	*B. vulgaris*	2020		
	CF2-4	*B. vulgaris*	2020		
	CFNKY-BZ2-4	*B. vulgaris*	2020		
	CFNKY-BZ2-23	*B. vulgaris*	2020		
	CFNKY-BZ2-22	*B. vulgaris*	2020		
	CFNKY-BZ2-17	*B. vulgaris*	2020		
	CFNKY-BZ2-20	*B. vulgaris*	2020		
	CFNKY-BZ2-21	*B. vulgaris*	2020		
	CFNKY-BZ2-8	*B. vulgaris*	2020		
	CFNKY-BZ2-14	*B. vulgaris*	2020		
	CFNKY-BZ2-13	*B. vulgaris*	2020		
	CFBZ2-2	*B. vulgaris*	2020		
	CFBZ2-14	*B. vulgaris*	2020		
	CFBZ2-9	*B. vulgaris*	2020		
	CFBZ2-1	*B. vulgaris*	2020		
7	YA0807-19	*B. vulgaris*	2020	Heilongjiang Province	Colonies with irregular edge. (Supplementary Materials Figure S1 Type VII)
	CF1016SY-62	*B. vulgaris*	2020	Inner Mongolia Autonomous Region	
	CF1016SY-66	*B. vulgaris*	2020		
	CF1016SY-71	*B. vulgaris*	2020		
	CF1016SY-48	*B. vulgaris*	2020		
	CF1017SY-91	*B. vulgaris*	2020		
	CF1019SY-78	*B. vulgaris*	2020		
	XJYL-34	*B. vulgaris*	2020	Xinjiang	

	XJYL-12	*B. vulgaris*	2020	Uygur Autonomous Region of China	
	XJYL-49	*B. vulgaris*	2020		
	XJYL-15	*B. vulgaris*	2020		
	XJYL-47	*B. vulgaris*	2020		
	XJYL-11	*B. vulgaris*	2020		
	XJYL-44	*B. vulgaris*	2020		
	XJHC-32	*B. vulgaris*	2020		
8	YL1002-22	*B. vulgaris*	2020	Xinjiang Uygur Autonomous Region of China	Colonies with irregular edge. (Supplementary Materials Figure S1 Type VIII)
	YL1002-13	*B. vulgaris*	2020		
	YL1002-90	*B. vulgaris*	2020		
	CF1016SC-51	*B. vulgaris*	2020	Inner Mongolia Autonomous Region	
	CF1016SY-4	*B. vulgaris*	2020		
	CF1016SY-3	*B. vulgaris*	2020		
	CF1016SY-46	*B. vulgaris*	2020		
	CF1016SY-13	*B. vulgaris*	2020		
	CF1016SY-35	*B. vulgaris*	2020		
	CF1016SY-47	*B. vulgaris*	2020		
	CF1016SY-24	*B. vulgaris*	2020		
	TCZF-1008	*B. vulgaris*	2020		
