# Supplementary material for: Characterization of the Mycovirome from the Plant-Pathogenic Fungus Cercospora beticola
Source: Viruses. 2021 Sep 24;13(10):1915. doi: 10.3390/v13101915 (PMC8537984; doi:10.3390/v13101915)
Supplement: Supplementary file 1 [file viruses-13-01915-s001.zip › viruses-1368835-supplementary Table S2.pdf]

Table S2. Primer pairs used to confirm the exitance of viral sequences in *Cercospora beticola*

Contig number	Putative name of viruses	Primer name	Primer sequence	Primer length	Amplication size (bp)
contig1357	CbHV1	1357-F	TGCTTGCTAGATTCGAAAGTC	21	741
		1357-R	GGGTATCCTGAGTTGTTCCAC	21	
contig965	CbOV1	965-F	CGAGAGAGAAGGCACAATCC	20	563
		965-R	TCCGACGAACAAAACCCACA	20	
contig1024	CbOV2	1024-F	TGAGTTGGTTCCTGGTCGTG	20	685
		1024-R	ATCCCTGGCGGTCTGAAAAG	20	
k141_5161	CbOV3	5161-F	CGGGAGGTATCGAACGTT	18	648
		5161-R	GAGAACCGAAACTGGGGGAG	20	
k141_72534	CbNV1	72534-F	CGAGACCGTTTCGCTGGA	18	765
		72534-R	ACAATACCCTGCAGTACCTT	20	
contig154	CbNSV1	154-F	CTAGAACAGTGCGCATCA	18	585
		154-R	CTGCCTGACCATCCCTTGA	19	
k141_3378	CbNSV3	3378-F	GATAGCCTCTCGGACATTGG	20	677
		3378-R	TTTTCTCTCGGGTGGCAGTC	20	
k141_6617	CbBYV1	6617-F	CCAAGATCTTTTGAGCCTGA	20	679
		6617-R	TTGACTCGGTTCGCAGTTGA	20	
k141_47347	CbBYV1	47347-F	CCGACGTATGACATGACAGGA	21	540
		47347-R	AACCCCAAGCACAGAAGC	18	
k141_67601	CbBYV1	67601-F	CCTCACTACGTCTACAAC	18	553
		67601-R	GTCGCCCCTATGTGTCTACC	20	
k141_63059	CbBYV2	63059-F	AGATGCGACAGAGGAGGACA	20	628

		63059-R	ACCGGACCATTTGACTGACC	20	
k141_13474	CbNSV5-B	13474-F	TGGTAGTGTTCTGGGCTTTGG	21	513
		13474-R	GGGCAGGTGGTGTTCGTAA	19	
k141_54286	-	54286-F	TCCGAGTCCTGATGCTTT	18	457
		54286-R	TCCATTACGCCAGACAAACA	20	
k141_31340	-	31340-F	GTTGACTACCGACTGAAGCTAC	22	649
		31340-R	GAAGAGATGGCTAAAGAGATAG	22	
Universal primers for Internal Transcribed Spacer (ITS) Region of fungi		ITS1	CTTGGCATTTAGAGGAAGTAA	21	-
		ITS4	TCCTCCGCTTATTGATATGC	20	
