# Supplementary material for: Characterization of the Mycovirome from the Plant-Pathogenic Fungus Cercospora beticola
Source: Viruses. 2021 Sep 24;13(10):1915. doi: 10.3390/v13101915 (PMC8537984; doi:10.3390/v13101915)
Supplement: Supplementary file 1 [file viruses-13-01915-s001.zip › viruses-1368835-supplementary Table S3.pdf]

Table S3. Viruses selected for phylogenetic analysis

Family	Virus name	Protein description	Accession number	Reference
Hypoviridae	Fusarium sacchari hypovirus 1	Polyprotein	QIQ28422.1	[1]
	Erysiphe necator associated hypovirus 2	RdRp partial	QHD64832.1	Unpublished
	Wuhan insect virus 14	Polyprotein	YP_009342443.1	[2]
	Alternaria alternata hypovirus 1	Polyprotein	QFR36339.1	[3]
	Trichoderma asperellum hypovirus 1	Polyprotein	AZT88614.1	[4]
	Fusarium sambucinum hypovirus 1	Polyprotein	BCP96869.1	[5]
	Fusarium graminearum hypovirus 1	Polyprotein	AZT88611.1	[4]
	Botrytis cinerea hypovirus 2	Polyprotein	QJT73706.1	Unpublished
	Macrophomina phaseolina hypovirus 2	RdRp	QOE55583.1	[6]
	Macrophomina phaseolina hypovirus 1	RdRp partial	ALD89099.1	[7]
	Cryphonectria hypovirus 1	ORF B	ATZ76095.1	[8]
	Cryphonectria hypovirus 2	Polyprotein	NP_613266.1	[9]
	Fusarium langsethiae hypovirus 1	Polyprotein	YP_009330037.1	Unpublished
	Fusarium poae hypovirus 1	Polyprotein	BAV56305.1	[10]
	Sclerotinia homoeocarpa hypovirus 1	Polyprotein	AZT88612.1	[4]
	Rosellinia necatrix hypovirus 2	Polyprotein	BBB86794.1	[11]
	Cryphonectria hypovirus 3	Polyprotein	NP_051710.1	[12]
	Cryphonectria hypovirus 4	Polyprotein	YP_138519.1	[13]
Botourmiaviridae	Magnaporthe oryzae ourmia-like virus 4	RdRp	QDW80874.1	[14]
	Neofusicoccum parvum ourmia-like virus 1	RdRp	QDB74998.1	[15]
	Pyricularia oryzae ourmia-like virus 1	RdRp	BBF90576.1	Unpublished
	Sclerotinia sclerotiorum ourmia-like virus 4	RdRp	QOE77939.1	Unpublished
	Epirus cherry virus	RdRp	YP_002019754.1	[16]
	Cassava virus C	RdRp	YP_003104770.1	[16]
	Entoleuca ourmia-like virus 1	Replicase	AVD68674.2	[17]

	Botrytis ourmia-like virus	RdRp	QLF49182.1	[18]
	Sclerotinia sclerotiorum ourmia-like virus 2	RdRp partial	YP_010084712.1	[7]
	Plasmopara viticola lesion associated ourmia-like virus 42	RdRp	QGY72572.1	[19]
	Cladosporium cladosporioides ourmia-like virus 1	RdRp	QDB74999.1	[15]
	Erysiphe necator associated ourmia-like virus 10	RdRp	QGZ98430.1	Unpublished
	Cladosporium uredinicola ourmiavirus 1	RdRp	QDB75001.1	[15]
	Pyricularia oryzae ourmia-like virus 3	RdRp	BBF90578.1	Unpublished
	Sclerotinia sclerotiorum ourmia-like virus 1	RdRp	YP_010084711.1	[7]
	Soybean thrips ourmia-like virus 1	RdRp partial	QQO81432.1	[20]
	Soybean leaf-associated ourmiavirus 2	RdRp partial	YP_009666498.1	[21]
	Soybean leaf-associated ourmiavirus 1	RdRp	YP_009666497.1	[21]
	Rhizoctonia solani ourmia-like virus 1	RdRp partial	YP_010084710.1	[7]
	Magnaporthe oryzae ourmia-like virus	RdRp	YP_009667033.1	Unpublished
Narnaviridae	Plasmopara viticola lesion associated narnavirus 4	RdRp	QIR30283.1	[19]
	Plasmopara viticola lesion associated narnavirus 3	RdRp	QIR30282.1	[19]
	Erysiphe necator associated narnavirus 18	RdRp	QJT93750.1	Unpublished
	Erysiphe necator associated narnavirus 35	RdRp	QJT93767.1	Unpublished
	Alternaria tenuissima narnavirus 1	RdRp	QDB74997.1	[15]
	Erysiphe necator associated narnavirus 13	RdRp	QJT93745.1	Unpublished
	Plasmopara viticola lesion associated narnavirus 2	RdRp	QIR30281.1	[19]
	Sclerotinia sclerotiorum narnavirus 1	RdRp	QUE49165.1	[22]
	Erysiphe necator associated narnavirus 12	RdRp	QJT93744.1	Unpublished
	Cladosporium tenuissimum narnavirus 1	RdRp	QDB74996.1	[15]
	Sclerotinia sclerotiorum narnavirus 2	RdRp partial	QUE49167.1	[22]
	Aspergillus fumigatus narnavirus 1	RdRp	AXE72933.1	[23]
	Neofusicoccum parvum narnavirus 3	RdRp	QTE76053.1	[24]
	Fusarium poae narnavirus 2	RdRp	YP_009272903.1	[10]
	Saccharomyces 20S RNA narnavirus	RdRp	NP_660178.1	[25]

	Saccharomyces 23S RNA narnavirus	RdRp	NP_660177.1	[25]
unclassfied (-)ssRNA virus	Hemipteran phenui-related virus OKIAV285	RdRp	QMP82212.1	[26]
	Mantodean phenui-related virus OKIAV283	RdRp partial	QMP82145.1	[26]
	Alternaria tenuissima negative-stranded RNA virus 2	RdRp	QDB75016.1	[15]
	Erysiphe necator associated negative-stranded RNA virus 10	RdRp	QJW70360.1	Unpublished
	Sclerotinia sclerotiorum negative-stranded RNA virus 5	RdRp	AHF48633.1	[7]
	Rhizoctonia solani negative-stranded virus 4	RdRp	ALD89133.1	[7]
	Cladosporium cladosporioides negative-stranded RNA virus 2	RdRp	QDB75018.1	[15]
	Coniothyrium diplodiella negative-stranded RNA virus 1	RdRp	QDB75015.1	[15]
	Fusarium poae negative-stranded virus 2	RdRp	YP_009272912.1	[10]
	Ixodes scapularis associated virus-6	Polymerase	AUW34408.1	Unpublished
	Fusarium graminearum negative-stranded RNA virus 1	RdRp	ATP75709.1	[27]
	Soybean leaf-associated negative-stranded RNA virus 1	RdRp	ALM62220.1	[21]
	Aspergillus fumigatus negative-stranded RNA virus 1	RdRp	BCH36617.1	Unpublished
	Sclerotinia sclerotiorum negative-stranded RNA virus 1	Large polymerase	AHW76811.1	[28]
	Sclerotinia sclerotiorum negative-stranded RNA virus 3	Gp5	YP_009129259.1	[7]
	Botrytis cinerea mymonavirus 1	RdRp	AXS76906.1	[29]
	Plasmopara viticola lesion associated mononegaambi virus 5	RdRp	QHD64776.1	[19]
	Penicillium cairnsense negative-stranded RNA virus 1	RdRp	QDB75012.1	[15]
	Sclerotinia sclerotiorum negative-stranded RNA virus 2	RdRp	ALD89145.1	[7]
	Sclerotinia sclerotiorum negative-stranded RNA virus 4	RdRp	YP_009666274.1	[7]
	Kiln Barn virus	Hypothetical protein partial	AWA82236.1	[30]
	Penicillium adametzioides negative-stranded RNA virus 1	RdRp	QDB75019.1	[15]
	Penicillium glabrum negative-stranded RNA virus 1	RdRp	QDB75014.1	[15]

Reference

1. Yao, Z.; Zou, C.; Peng, N.; Zhu, Y.; Bao, Y.; Zhou, Q.; Wu, Q.; Chen, B.; Zhang, M. Virome Identification and Characterization of *Fusarium Sacchari* and *F. Andiyazi*: Causative Agents of Pokkah Boeng Disease in Sugarcane. *Front Microbiol* **2020**, *11*, doi:10.3389/fmicb.2020.00240.
2. Shi, M.; Lin, X.-D.; Tian, J.-H.; Chen, L.-J.; Chen, X.; Li, C.-X.; Qin, X.-C.; Li, J.; Cao, J.-P.; Eden, J.-S.; et al. Redefining the Invertebrate RNA Virosphere. *Nature*

2016, *540*, 539–543, doi:10.1038/nature20167.
3. Li, H.; Bian, R.; Liu, Q.; Yang, L.; Pang, T.; Salaipeth, L.; Andika, I.B.; Kondo, H.; Sun, L. Identification of a Novel Hypovirulence-Inducing Hypovirus From *Alternaria Alternata*. *Front Microbiol* **2019**, *10*, 1076, doi:10.3389/fmicb.2019.01076.
4. Gilbert, K.B.; Holcomb, E.E.; Allscheid, R.L.; Carrington, J.C. Hiding in Plain Sight: New Virus Genomes Discovered via a Systematic Analysis of Fungal Public Transcriptomes. *PLoS One* **2019**, *14*, e0219207, doi:10.1371/journal.pone.0219207.
5. Mizutani, Y.; Uesaka, K.; Ota, A.; Calassanzio, M.; Ratti, C.; Suzuki, T.; Fujimori, F.; Chiba, S. De Novo Sequencing of Novel Mycoviruses From *Fusarium Sambucinum*: An Attempt on Direct RNA Sequencing of Viral DsRNAs. *Front Microbiol* **2021**, *12*, 641484, doi:10.3389/fmicb.2021.641484.
6. Wang, J.; Ni, Y.; Liu, X.; Zhao, H.; Xiao, Y.; Xiao, X.; Li, S.; Liu, H. Divergent RNA Viruses in *Macrophomina Phaseolina* Exhibit Potential as Virocontrol Agents. *Virus Evol* **2021**, *7*, veaa095, doi:10.1093/ve/veaa095.
7. Marzano, S.-Y.L.; Nelson, B.D.; Ajayi-Oyetunde, O.; Bradley, C.A.; Hughes, T.J.; Hartman, G.L.; Eastburn, D.M.; Domier, L.L. Identification of Diverse Mycoviruses through Metatranscriptomics Characterization of the Viromes of Five Major Fungal Plant Pathogens. *J Virol* **2016**, *90*, 6846–6863, doi:10.1128/JVI.00357-16.
8. Mlinarec, J.; Nuskern, L.; Ježić, M.; Rigling, D.; Ćurković-Perica, M. Molecular Evolution and Invasion Pattern of Cryphonectria Hypovirus 1 in Europe: Mutation Rate, and Selection Pressure Differ between Genome Domains. *Virology* **2018**, *514*, 156–164, doi:10.1016/j.virol.2017.11.011.
9. Hillman, B.I.; Halpern, B.T.; Brown, M.P. A Viral DsRNA Element of the Chestnut Blight Fungus with a Distinct Genetic Organization. *Virology* **1994**, *201*, 241–250, doi:10.1006/viro.1994.1289.
10. Osaki, H.; Sasaki, A.; Nomiyama, K.; Tomioka, K. Multiple Virus Infection in a Single Strain of *Fusarium Poae* Shown by Deep Sequencing. *Virus Genes* **2016**, *52*, 835–847, doi:10.1007/s11262-016-1379-x.
11. Arjona-Lopez, J.M.; Telengech, P.; Jamal, A.; Hisano, S.; Kondo, H.; Yelin, M.D.; Arjona-Girona, I.; Kanematsu, S.; Lopez-Herrera, C.J.; Suzuki, N. Novel, Diverse RNA Viruses from Mediterranean Isolates of the Phytopathogenic Fungus, *Rosellinia Necatrix*: Insights into Evolutionary Biology of Fungal Viruses. *Environ Microbiol* **2018**, *20*, 1464–1483, doi:10.1111/1462-2920.14065.
12. Smart, C.D.; Yuan, W.; Foglia, R.; Nuss, D.L.; Fulbright, D.W.; Hillman, B.I. Cryphonectria Hypovirus 3, a Virus Species in the Family Hypoviridae with a Single Open Reading Frame. *Virology* **1999**, *265*, 66–73, doi:10.1006/viro.1999.0039.
13. Linder-Basso, D.; Dynek, J.N.; Hillman, B.I. Genome Analysis of Cryphonectria Hypovirus 4, the Most Common Hypovirus Species in North America. *Virology* **2005**, *337*, 192–203, doi:10.1016/j.virol.2005.03.038.
14. Li, C.X.; Zhu, J.Z.; Gao, B.D.; Zhu, H.J.; Zhou, Q.; Zhong, J. Characterization of a Novel Ourmia-Like Mycovirus Infecting *Magnaporthe Oryzae* and Implications for Viral Diversity and Evolution. *Viruses* **2019**, *11*, E223, doi:10.3390/v11030223.
15. Nerva, L.; Turina, M.; Zanzotto, A.; Gardiman, M.; Gaiotti, F.; Gambino, G.; Chitarra, W. Isolation, Molecular Characterization and Virome Analysis of Culturable Wood Fungal Endophytes in Esca Symptomatic and Asymptomatic Grapevine Plants. *Environ Microbiol* **2019**, *21*, 2886–2904, doi:10.1111/1462-2920.14651.
16. Rastgou, M.; Habibi, M.K.; Izadpanah, K.; Masenga, V.; Milne, R.G.; Wolf, Y.I.; Koonin, E.V.; Turina, M. Molecular Characterization of the Plant Virus Genus *Ourmiavirus* and Evidence of Inter-Kingdom Reassortment of Viral Genome Segments as Its Possible Route of Origin. *J Gen Virol* **2009**, *90*, 2525–2535, doi:10.1099/vir.0.013086-0.
17. Velasco, L.; Arjona-Girona, I.; Cretazzo, E.; López-Herrera, C. Viromes in Xylariaceae Fungi Infecting Avocado in Spain. *Virology* **2019**, *532*, 11–21, doi:10.1016/j.virol.2019.03.021.
18. Ruiz-Padilla, A.; Rodríguez-Romero, J.; Gómez-Cid, I.; Pacifico, D.; Ayllón, M.A. Novel Mycoviruses Discovered in the Mycovirome of a Necrotrophic Fungus. *mBio* **2021**, *12*, e03705-20, doi:10.1128/mBio.03705-20.
19. Chiapello, M.; Rodríguez-Romero, J.; Ayllón, M.A.; Turina, M. Analysis of the Virome Associated to Grapevine Downy Mildew Lesions Reveals New Mycovirus Lineages. *Virus Evol* **2020**, *6*, doi:10.1093/ve/veaa058.

20. Thekke-Veetil, T.; Lagos-Kutz, D.; McCoppin, N.K.; Hartman, G.L.; Ju, H.-K.; Lim, H.-S.; Domier, L.L. Soybean Thrips (*Thysanoptera: Thripidae*) Harbor Highly Diverse Populations of Arthropod, Fungal and Plant Viruses. *Viruses* **2020**, *12*, E1376, doi:10.3390/v12121376.
21. Marzano, S.-Y.L.; Domier, L.L. Novel Mycoviruses Discovered from Metatranscriptomics Survey of Soybean Phyllosphere Phytobiomes. *Virus Res* **2016**, *213*, 332–342, doi:10.1016/j.virusres.2015.11.002.
22. Jia, J.; Fu, Y.; Jiang, D.; Mu, F.; Cheng, J.; Lin, Y.; Li, B.; Marzano, S.-Y.L.; Xie, J. Interannual Dynamics, Diversity and Evolution of the Virome in *Sclerotinia Sclerotiorum* from a Single Crop Field. *Virus Evol* **2021**, *7*, veab032, doi:10.1093/ve/veab032.
23. Zoll, J.; Verweij, P.E.; Melchers, W.J.G. Discovery and Characterization of Novel *Aspergillus Fumigatus* Mycoviruses. *PLoS One* **2018**, *13*, e0200511, doi:10.1371/journal.pone.0200511.
24. Marais, A.; Faure, C.; Comont, G.; Candresse, T.; Stempien, E.; Corio-Costet, M.-F. Characterization of the Mycovirome of the Phytopathogenic Fungus, *Neofusicoccum Parvum*. *Viruses* **2021**, *13*, 375, doi:10.3390/v13030375.
25. Rodríguez-Cousiño, N.; Solórzano, A.; Fujimura, T.; Esteban, R. Yeast Positive-Stranded Virus-like RNA Replicons. 20 S and 23 S RNA Terminal Nucleotide Sequences and 3′ End Secondary Structures Resemble Those of RNA Coliphages. *J Biol Chem* **1998**, *273*, 20363–20371, doi:10.1074/jbc.273.32.20363.
26. Käfer, S.; Paraskevopoulou, S.; Zirkel, F.; Wieseke, N.; Donath, A.; Petersen, M.; Jones, T.C.; Liu, S.; Zhou, X.; Middendorf, M.; et al. Re-Assessing the Diversity of Negative Strand RNA Viruses in Insects. *PLoS Pathog* **2019**, *15*, e1008224, doi:10.1371/journal.ppat.1008224.
27. Wang, L.; He, H.; Wang, S.; Chen, X.; Qiu, D.; Kondo, H.; Guo, L. Evidence for a Novel Negative-Stranded RNA Mycovirus Isolated from the Plant Pathogenic Fungus *Fusarium Graminearum*. *Virology* **2018**, *518*, 232–240, doi:10.1016/j.virol.2018.03.008.
28. Liu, L.; Xie, J.; Cheng, J.; Fu, Y.; Li, G.; Yi, X.; Jiang, D. Fungal Negative-Stranded RNA Virus That Is Related to *Bornaviruses* and *Nyaviruses*. *Proc Natl Acad Sci USA* **2014**, *111*, 12205, doi:10.1073/pnas.1401786111.
29. Hao, F.; Wu, M.; Li, G. Molecular Characterization and Geographic Distribution of a Mymonavirus in the Population of *Botrytis Cinerea*. *Viruses* **2018**, *10*, doi:10.3390/v10080432.
30. Medd, N.C.; Fellous, S.; Waldron, F.M.; Xuéreb, A.; Nakai, M.; Cross, J.V.; Obbard, D.J. The Virome of *Drosophila Suzukii*, an Invasive Pest of Soft Fruit. *Virus Evol* **2018**, *4*, vey009, doi:10.1093/ve/vey009.
