# Supplementary material for: Characterization of the Mycovirome from the Plant-Pathogenic Fungus Cercospora beticola
Source: Viruses. 2021 Sep 24;13(10):1915. doi: 10.3390/v13101915 (PMC8537984; doi:10.3390/v13101915)
Supplement: Supplementary file 1 [file viruses-13-01915-s001.zip › viruses-1368835-supplementary Table S4.pdf]

Table S4 The sequences of 99 primary contigs assembled from clean data of 139 strains of *Cercospora beticola*

>Cb _clean_k141_7467
CGCGGCATTAGCCGCTGCGTCTGTGGTGCTCTTACACAGGCCCCTCAGGGGACATATATAAGAGATGATTGCTCCACGCTTTCAGTGGATTCCATTTG
TCAAAGGTGGTTTTCCTTTGTCTGGAAGCAGATAATTTCTGCAATCGGATCCTTGCGGATCGCGGCACGTGCCATGCCGAGGCCTCTGTGAGGCCGT
TGCCCTTCCCGGGCGTTGTCAATTGTGACGAGGTGCGGAGCACCGAGCTATCGCGTCTACGAGTCCGGAATGACTGTAAACTATTGCAACAGGGCG
GTGTCACACCCAAATGTGTATAAACTTGCGCGTTTCGGAGCGTTCTCAAACGCTGACCTACACTGGGTAAACGGATGGTTATCAGAACGGCGATGAG
GTAACTGTGGACTAGTGACCCTAGAAGTTATCCACCTCATTGTGAACCATCTTCCCTTTAAGATTTAGAAATGAGCACCGCTCTAAACGGTTAACACC
ATTTTGTTTTCGGCTTCCCTCTGGACGGGACGATTCAAAATGTCTTCTATCAATTTTCTTCGTTTGGATCAACGCAAAGATCCCGGGAGGCTCCGAGT
GCCCAAAGAACGGTCTGCTATCCGTTCATCCTATTCAAGTTCAGCCGGGACTTGGAGGCTCCGCAGGGAGTCGCTGCCGGCATGGGCCCAGCCCAA
GTCTTCTCCTGTTGGTCTTCTCGCTTGTGGTGAGCTGACCAGTACTTCTTCTTTCGGCGAGGTGGTTCAGTCCACGCCTCGCCCAGCCACCATGGCT
GCCAAAGTGGACGTCTGTCAAGTGAGGTACTTGACATTCTCCGACTCATCTCAAGGAATAAAACACTGGTTGATTAAAAACCTGCCTGTCTCTCACC
CTGATGTGTTAGAGATGAGATTACATGGCTTTGTCGATCGCCTGGCAACAGTGAGCTCTCAACATTCTGCTTGCAGAGTTGAAGCTGAAAGTGCACT
GTTTTCCCCATCCCGGACTCCTGGTCACATGCAGAAGTTGATGGATGGAGTGTTGGCCCTTCGTCGCTCTGGTCAGATGACCTGTGTCAAGAAGGAA
TCTCCGGCTGAGTTCGCGCGCCGTAAGCGTGAACAAAAGTGGTCTTGGCTCATCAGGCGTCTGCAGAGGCGTTCAGCTGCTGAGCAAGCTGTGATC
GCTGAACAGCGGTTGACAAAGGCCCTGCATTTGCAGGCCAATAGTTTTTCCTTCGTCCCCACTGGGCCGAAGGGTTTCAAAGGTCTGGGAGAGCTG
GTACTCCCTACCCCTTCTGCTGAAGAGATCGCTTTTGGTTCCTCCTTCCTAGATGGCAAACGTGTTCGTGTTTTGCCAAGGAGAGAGAAGAATACTC
GGTTTTCCGCTTGGTGCCAGTCAAATGCCGAACGTAAGAAGGCATTAAAACTAGCCGAGTTGGAACGTCAAAGGTTTAGATTGTCTTCGTTGGTCGC
TGACCCTCTTCGTGTGCTCGGATGTCAAATTCCTGAGACGCTCAGTGGTGTCATTTCTTTCGCCCGTGGTCTTTTTGGCACTTCTTCACGTCGCTCTG
TCGTCCGAAGGGATTTCATCCATCGGGCTAGATACGCTGAGAAGTTCTTCTCTTGTCCTGTCAATGGTTACATTGTTGATTTGGTCGGGAACAAGATA
AGGGTGTCCAGACATAGGACAGGATCTGGTGTCTCTTTTCGCGTTGATGGTGGTCAGGTCGCCCACTGCGGCCTAAAGGACGAGTATCTTTGTGCTG
TTCTTCAGTCTAGGCTTGATCTAGTACCAAACCCCGGCGAGCCAGAGATCCTGACTATCGAGTTATTCCGTTCGAGATACGAAGGTTATTTCGGGAGG
TTTTTAACTGAGATGGACGGAACTAACCTCGACCTATGGTCTTTAATGACCATGGCCAAGAGATGGAAAAGAGATTTTGGTTGCCCTAGCAATACTT
GCATTTTGTCTTTTGCGGGTGACTCCGTTTCGTTTACTGTTGTCAACGAACAAG

> Cb_clean_k141_55778

GGAATACTCGTGTGCACCCCTTACGTCCAAACGGGTCTGGATATAAAACCACCACCAGATCTTTTGATCGATTGTGGCAAAGACTTCGTTTTGGACA
AAGGGCGTCCAGTATTTCCACTTCCGTGGACAGACAAGGACATACACCAGCAGAGAAGATGGAGGCGAGTGTCTGGAGTGCCAACTCAGGGGCCA
ATACGAGCCCGGTGAACACGACAGCGACATCGAGAATGAGCCGAGGCGCAGTGACTCGTCGGATGAAGACGACGAGTAGCCTTTCCGACGGCGAA
GAGATCACTCGACTAGAGCAGTAGCGGCCATGGTAGGACCACTTTGCCTTCTCAATGTGTTGAGCTCCGCTAACCAACTCGTAGCCAACGCGTGGCT
TGCGAAAGGCCGACTGCAGACCGGCAGAGGCTCCCGCATCA

>Cb_clean_contig_2817
GTGGGTTGCCGCTGCCTCTCTGTCCGAGCTTCAAAAATCACTTGACTGTGTGGTAAAGGCTTCACATCCTTTCAACACCGCGCTTGTGAGTAAGCTG
CGTGATGAGACGAACTCATTCTTTCAGAAGATCTCCGATTCTGAGCCAATAGGTGGTACAACCGTATCTGCCTTAGCTCATACATTCAAGACTCTATA
CGCCTGCCCTAACAATGGGTGCGGTTACGCACTAGAGATAAAACCAGAAGATGGAATGGTACTATTTCATTTGACTGAGTCTCCAGACGAAACCGTT
AAAAACAGGTTGTGGTGGGTTTTGAATGGGAATGAGATTTTGCATGGAGGGGACAAAACCCCTGTTTCCCCAAGCACCGTCTCATTCGGTTGGCTA
GCAAAGGAATATTCGACACATGAGGTGATCATCGAGCCCAAACCGGACTCTATGCTATCACAAGAGAAACCAGGGCCGTTTGCAATGCCCTCGCTTT
CTCAGTTTCAGAAATGTGTCGACAAGATCATCCCTTCTGGTAGTCATTTCTCTCGTTCTAAAGTCGGTGAACTCAAAGATCACATCAACCAGTA

> Cb_clean_k141_41826
GCGAACACCACTTAAAGCAAGTGAATGTATGAAAACAACTGACTTTTCGACGGGTAGAGAATTTAGCACTTTCTTGTTCGGTCGCAAGAAAGGCAA
ATCTGCCGTGAAAGCACCCGAGATTGGTGCGAGCTGTGGGACCTTGAAGTGTTCCGCGACAGCTTTATGTTGGGGGGTGATTGGTGTGCGTGAAGG
TTGTCACGTGTTGAAGAGTGATTGCCCACTGCCAACGCGGCGCACGGTTGCTATGCCAAGAGGTGAGACGTCGACTTCACCTTCAACTTTACTTCTG
TACCTCGTCTTTGCCATTGGCGTTACATCAACAAAGACGACGCCCATCATTTCTTGATATCTCAATGAGACCCGAAGATGCATTTATGCCCTTGAATAC
AGTCATCCTTCCCTGACGCGAGTACAAATATGAATAATTGGACAGCAGTACCAGACCAACGCGGGCGTGGACGCCGGGCACTTTGCGGGCCCGAGT
ATCAGAGCCACCAGTGATGAGGAGTTGGGCGCTGATAGCTTATAATACTGCGAGGCACCATGATACGA

> Cb_clean_contig_1712
GAACACTTCAAGGTCCCACAGCTCGCACCAATCTCGGGTGCTTTCACGGCAGATTTGCCTTTCTTGCGACCGAACAAGAAAGTGCTAAATTCTCTAC
CCGTCGAAAAGTCAGTTGTTTTCATACATTCACTTGCTTTAAGTGGTGTTCGCCAAGACAAT

>Cb_clean_k141_13671
GTTCATGTGGATGTCAAGTACCCTATAGTAACTAGGGAACATGTTTCCTCTCAACCAGGAGAGGAAAGCAACTTGACGCGGCGAGAGCTGTTGTTGT
GGGGAGTGCGCAATACTTTGCATAGATTATGGCCAGCGATGTGAATCGACATACACGTGCGTGTCAGATCTTACAGCCAAGTTCCCGATCCTGGGCA
ACATTGGTAGAAGCCTCTGACGAGACCTCATTCGCCGCCTTTGCCTGCTTGTTTATTTGGAAAGCTGCGTGGCCGTCGGTTGCTTCCTGGAGAGGAG
CCCGCTTTTGGTCTTTGAAAGCGTGATTTACTTTGTGATCTTTCTTTTGCGTTTTTGAAATTGCAGTC

> Cb_clean_k141_13420
CGCTGTTGCCACAACGGAGCGATCACCCTCTAGAACACGTAGGTTCTTTGGAAAGATCGACATTCACGGCAAGAGCGCGACGGAAGAAGTTAAGG
ACATTTTTGGATTCAAAGAACATGACGAGGTGTATCCTTACACTGGTAGACCGGAAAAGCCTCACGCCTATGAGGTTGAAACTCCGTTGCCTGTCAG
CAATCGGTTTTCCTTTCGTGCAACTCATCGTCAGGCCAAGGTGATGGCCAACGACTTTGAAGAGAAGTGGAGAGAAGACTCTAAGTGCAGAGCAC
GGACGTGGCACATCGAAGGTGATGACACACCGAAGCAAAAGCTTGCTCGGTCCCATCACCCTTTGTGGTGTCCGGACACGTTCAACGAACGGTTC
GACGAGATTTTCGTTGCCGCGTACATTTGGTTTTTCTTTCAACTCATTTGCACGTTTTACTTCTCGTTTTCTCGGCCTAGTCTTCGGCGGAAACCTGAC
AATCACACGGAAGGAGGAGAGCATCCTCTTGGAGATGAGGGTTTTTCTGCATCATCCTCCTTACTCGTGTGCAAGTTAGGAACCAGAGGCGACCAG
GTCCCTGTCGATTATTACGCGAACGTTGCTGCACACTATGGTGTCCGCACACATTCGCACGTGTACAAAGATGTGACGACAGACGATCTTGAAAAGA
TGAAAGTCGGAGACTTTTCATCTAGTTCCGCTGGTTATTTGAATCTTGCGTACGCCTCACAACTTGGTTATAAAAGAGTGTTCACCCCGCATGTGGAG
GTTGAAAGGTCACAGGGTGTGAGCTACACCTTGTCACCGAGTTCAAGATGGGTAGAATCGACAAGATTTATTGCTGACTGGTCAAAGGTTTCTTGGT
CCAATTACCTCCCCGCGTGGATAGCCACGCGGGTCACTGCTGTGTATGTGCCAACTTTCAGAATTGGTGCACTGTCTGGAAGCGACATCCCGCGTTC
TCATGACGGGCTGAACCTACTGGAGAAAGTAAAGAACACAGCGAAGTTCGATGAGGGTTGGACATCTGGCAGCTCATCAGTTTCAGTGATCCCTGA
TGAGATCAAGTCAAGATGCCCCGAAATACCATCGGGTAACCATCTTGAGATATTAAAGGATTACAAAACGGTGCACATCCATGGTGGAGCTGGTACC
GTGCAAACCGCCATCGCTTGCGGTGCACGACCGGTCATCCACGACAAGACACTCGATAGAGTGTATCATAGGATGCCTGAGCCAGAGGATTTCAAA
CAACCTTCCATTGGGAAATTCATGGGTTGGTTGGTATCCGCTGGTTTCAACGTTAATGCACCAATAGAGGTAAGAGCATGGTGGTTGTTACAATGGTA
TTGGTCCCAAAAGTGGAACTATGCGTACGATGCATTAGTTACACTTGTTAAAGCTTACACCATATCATCGTATGTATACAACCATTGGGAGCTTTTCCT
CATTCTTTTCTTCACCGTTCCGGCAGTGGTCTGGAGAATGTTGTTTTCTTCCAAATCATTATTCTCCATAGTCAAAATTTGTGCGCTTGGTTTCTGGCG
CTACCCTGTGTTGTGTTTGTTAGATTCGAAAGTCACTTTCGTGGCAGGAATCTGGTCAATGCAAAACTTGTGGATGGGCGCGGCGCAAGATTTGTCT
GCACTCATGGAGACACGATCTTCACTGGTTTGGGAGCCAGTGCAAAAGACCGTAGGAACGAAGAGATTGACGTTCCCTTTTCCCCTTGGGCATTGG
AGCATGAAAGATAACCAAACTGGTAAGATCTACGAAGGTAGATTTATTGGTCCCTCCGAGACTATCGGAGGGAACTTTCGCCTTGTGAGCACTACTC

GTGAATACAAGCGTGGATTT

>Cb_clean_k141_9569
TTTCTGCAATCGGATCCTTGCGGATCGCGGCACGTGCTATGCCGAGGCTCTTCCGGGCCGTTGCCCTTTCGGGCGATGTCAATTGTGACGAGGTGCA
CCGCACCGAGCTATCGCGTCTGTGAGTCTGGAATGACTATAAACTATTGTGACAGGACGGTACTACACCCAAATGTGTATAAACTTGCGTGTTTCGGA
GCGTTGTTCAAACGCTGGCCTACACTGGGTAAATCGATGGTGATCAGAACGGCGATGAGGTAACTGTGGACTAGTGACCCTAGAAGTTATCCACCTC
ATTGTGAACCATCTTCCCTTTAGAATTTAGAAACGAGCACCGCTCTAAACGGTTAAACCCATTTTGTCTTCGGCTTCCCTCTAGACGGGACGATTCAA
AATGTCTTCCATCAACTTTCTTCGTTTGGATCAACGTAAAGATCCCGGGAGGCTCCGAGTGCCTAAAGAGCGGTCTGCTATCCGTTCATTCTATTCAA
GTTCATCCGGGACTTGGAGGCTTCGTGAGGAGCTGCTACCGGCGTGGGCTCAGCCCAAACCTTCCCCTGTTGGTCTCCTTGCTTGTGGTGAGTTGAC
CGAAGGTTCTTCTTTCGGCGATGTGGTTCAGTCCACGCCTCGTCCAGCCGCTACGGCTGCCAAGGTGGACGTTTGTCAAGTGAGGTACTTGACATTC
TCTGACTCTTCTCGAGGAATAAAGCGCTGGCTGATAAAGAGCTTACCTGTCTCTCACCCTGATGTGGTGGAGATGGCGTTGCACGGATTTGTCGACC
GCCTGGCAACGGTAAGCGCTCAACACTCTGCTTGCAGAGTTGAGGCTGAAAATTCCTTGTTTTCCCCATCTAAAGCTCCCGGCTGCGTGCAGGGATT
GATGGATGGTGTTTTGGCCCTTCGCCGATCCGGTCAGATGACCAGTGTCAAGAAGGAATCTCCGGCAGAGTTCGCGCGCCGTAAGCGTGAACAGAA
GTGGTCCTGGCTCGTTAGGCGCTTGCAGAGGCGTTCGGCTGCCGAGCAGTCCGTGATCGCTGAACAGCGGTTGACGAAAGCTCTGCACCTACAGGC
CGACATTTCTTCCTTTGTCCCGAGCGGACCAAAGGGTTTTCATGGTTTGGGAGAGTTGGTACTCCCTACCCCTTCCGCCGAAGAAGTTGCTTTCGGT
TCTTCTTTTCTTGGTGGCAAGCGCATTCGCACCTTGCCAAAGAGGGAGAAGAACAGCCGGTTTTCTGCTTGGTGCCAGTCAAATGCCGAACGAAAG
AAGGCATTGAGACTGGCCGAGGTGAAACGCCAGAAATTCCGATTGTCTTCGTTGGTGGCAGACCCTCTTCGTGTACTCGGATGTCAAATTCCTGAG
ACATTGGATGGTGTCGTCTCTTTTGCTCGCGGCCTCTTCGGTTCCTCTTCGCGTCGCTCTGTGGTCCGTAGGGATTTCATTCATCGGGCCAGGTACGC
TGGGAAGATTTTCTCTTGCCCTACCAGTGGTTTTATCATCAACCTGGTTGATAATAGGGTGAAAGTGTCGAGGAACAGGACCGGGTCCGGTGTTTCT
TTCCGTGTTGATGGTAATCAGATTGCTCACTGTGGCTTAAAGGATGAGTACCTCTGTGCCGTCCTACAGTCCAGACTCAATCTGATACCAAATCCTGG
TGAGGCAGAGTTCCTGACCGTCGACTTGTTCCGTTCGAGATACGAAGAATACTTCGGAAGGTTCCTCGCCGAGATGGATGGAACCAACTTGGACTT
GTGGTCCTTAATGACCATGGCTAAAAGATGGAAGCGGGATTTCGGATGTCCTGGCAATACTTGCGTCTTATCTTTTGCGGGTGACTCAGTTTCATTCA
CCGTTATCAATGACCAAGTCGAGTTACCGCCGGCATTGTGGTTTCGTCTTGATGGAAAGGATCTATTCCATTGCGGGGTTGACCAAGTTAAAAGACT
GCAATTCCAAAAGAACATTTGGAAGGCGGTTGCCAAACTGGTCATTCAGGATGGTAGTCCGGCCGAACAGCGCTTGGCCGAGAAAGTCTCTTCTTC
TGCGCATGTGAGTGTCCCTTGTCTGAAACCTAACCTGTTCCATCAGCTGACGAACGGTTTCAGGGTTTGTGCCAAGTTTTCCGGTCTGAGCGTCTTC
GAACGCGGACTTGATTTCACACGACCTGACCCTCGGGTTGAGGGTGGTTTCATCACCACCAGGCAGGAGAGATTGTTTTGGGATACAGGAGCAAGT

AGGTTGGTGTTCACACCAAAGGGCCTGGCCAAAATATTCTTCCGTAAACACAATCAAGAAAAATGGAGAATAGCTCTGGCTGCCGCGATTGGTTTG
ACCCATGATGAAGGTCTGCTCTTCTTCTCGAAGTTTCACAATGATAGTACGAAACCAGATGTCGTGTTGGAGGTTTTGGGGTCATCACCCTACCACA
AGCCGGACAAGGAAACGGTAACAAGAGTAGCTTTCATTGACGTGGAGAAACCGGTTTCATCCAGGCCTATACCTCGGCTTCCCACAGTTGTTCCGC
CTCCTCCGCCTGTACCACCAGTGCAGGGAGTTAAGCAGAAGGGACCCAAGGTGGAACTGGCTGATGGTGACACGTGGCTGCTTGCTGAAGGTTCT
GAAGGTAAGTCAAATAGAGCTCGCTTCAGATCTCTCTTCCCACATGATTGGTGGTTGAGAGTGTCCGAAAGGGTCATGCCCTTTATGAATCAAGACG
CATCCCGTGACGTTGGATACAAGTCCCGATCTTGGTCCCAATCTTCCCTTGTCTCAGATCCCTCAGAGAAGTTTGATGTCAACGCCCAAAAATACAC
ACCTTCTAGCTCTTCCAAAGCATCAGGTTCAACACCTTTACGTGTTGATCCTGAAGCCTTTAGGAGGCAGCTGCAAGCCAAGGCTAAGAAAGTGGG
TGCGACAGCGTCTTCCCGACCTGATGCGAGGGAGGCGTATCTGAAGGGTGCCAACGAGTTCAAACCTGAGAAGGTTTCTCATAGAAAAGCCCTTTC
TGAGCCCACTCCGTCGAAGAAACCTTCTTCTGAATGGATGTCTATTTCGCGAGCATCCTCTGATTATCTCTTTGATGATGATCATGAGGACGCGAGAG
ATTCGACGAAATCGGACATCGAAACTAATCCCGGACCTTCTATCAGGCATTTCCGTTCTAAATCGACTTCAGATTTGGAGGGTGAAACACCGTCATTG
AATTTCACACCCCCGACCCCTGGTGATGTCTTGACTGTGCACCATCCGACACCACCTCGTTTGAGGGTCAATCACAGGATGAACAGACATACCGTAA
TTGTACCTTCACCGGGTGAGAACACGTCGAATTGGAATGTAGGTTACTCGTCATTTGCATGGCCTCCAACGGAGGAACATCTGGAACCTCGAGACTC
CAGAACCGCGGATATAGAAGAAAATCCCGGACCATGTGAAACCCTTGACCCCACTTTGCCTCAGTTTCGAGGTCGGAGTGGTTCTCAGAGTTCAGT
TGCCGCTGTCACTTCGGAGCGATCACCTTCAAGAACACGTAGGTTTCTTGGGAAGATCGGCATCCATGGCAAAAGCGCAACGGAAGAAGTTAAGG
ACATTTTTGGATTTAAAGAACATGATGAGGTGTATCCGTACACTGGTAAAAACTCATCTTTTGTTGAAAAGCATTATGAATTCCACTATGTTCTTTCTC
ATTTACCTAAAGACAAGAAGTGGCGGTCATCAACCTTTATCAGGTGGGTTGCCGCTGCTTCTTTGTCCGAGCTTCAAAAATCACTTGACTGTGTCGT
TAAGGCTTCCCACCCTTTCAACACCGCGCTAGTGAGTAAGTTGCGTGATGAGACAAACTCCTTCTTCCAGAAGATTTCTGATTCTGAGCCAGTAGGC
GGTACAACCATGTCTGCCTTAGCTCACACGTTCAAGACCCTATACGCCTGTCCAAACAATGGATGCGGTTATGCATTGGAGGTGAAACCGGAAGATG
GAATTGTACTGTTCCATCTGACAGAGTCCCCAGACGAAATCGCTAAGAATAGGTTGTGGTGGGTCTTGAATGGGAATGAGATTTTGCACGGAGGGG
ACAAGGCTCCTGCCTCTCCAAGCACAATCTCATTCGGTTGGCTGGCAAAGGAGTATTCAACACATGAGGTGATAATTGAGCCCAAACCGGACTCTGT
GTTATCACAAGAGAAGCCAGGGCCGTTCGCGATGCCCTCACTTTCTCAGTTTCAGAAATGTGTTGACAGGATTATTCCTACTGGTGGTCATTTCTCCC
GCTCTGAGGTCGACGACCTCAAAAATCACATCAACCAATACCTGGAGTCTACAGATGTCAAGATAGTGGATCCAGTGTTGGTTGCAGCGATGGCAAT
GAGCTTCAAGTCAAAGTTCAATTGTCCAAAAGATGTCAGCTCTTTTGCAATTGATTTTAGACTTGACCGGAGGATGGTTGTTTTCTGCGCCACAGATT
CCCCTGAAGACAAAAATAAGGGGAAGCTTTATTGGTGCATTGGTGGTAGTGTCATTGTCCATGGCGGGCTGCTAGGTATCGAAGATGGGAAGTCTTG
TTCCCCTTCACTGGATCACTATAGCAAGTTGTGGACGATGCCGCTTCCAAAAACGGGTTGGGTGGACAGGTCATTCGACGCTGAATACTTCACAAAG
ATCTCAACCTGGCAAAAGGCCCTCGACCGGTGTGAACCGATCGTTGTATTCTCTGAACACACATTGCAACGCACACTTTACCAATACTTTGATGGTG

TCACTGACAAGGTGTTATCACCATCACATACTGCAATCGTTGCTTGTGTGGTAAAATCCCTGATGAAATGCCCTGAGAGCGGGGTAGGTTATGCTATA
CAGTTCGATAGTCATGGGGCTTCGCTGTATGTGTGTCGACAACCGAACTCCGCAGAGGTGAACCGGTTGTGGTACTTTCACTGGGATTTTCCTTTCG
TGCATGGGGGAAGAAAATCTTATGCTCCGGAACCCAGGCAGTTCTTCTACACTGAGCATCTCAACATAGATTATTCGATACCCATGCGCGAAGTTTTT
GCTGGGGAAGGTCTGCCCAAATCCAATTCCTCACATTTGACTTTCAGGAGTCAACAGGAATTGTTTTTCGGTTTCCAGCAGCAAATAGATTTGTATG
ATGTGCCGACACCGGCATTCATGCATTCGAGAGCACATGGTGTGGCTGAACAAGCCATTCGTGACACAATACCCAAAATGAGAACTCTCCAGTTCA
ATTTTGAGCAAGTTGCCGCGATAGCCATGTGTTATAGATCAAGGATGCAATGCCCATGTCCGGAGTCAGCCATGGCTGTTGACTTTGACTTCGCCAAT
GAGCGCATTGTGTTCTTTTCGAACTTAGGCAGTGGAAGTTTCCGCTGTCGAGGTCAAATGTGGTGGTGTATAAATGGAGTCGGACTAGTGCACACTG
GTTCTACATTCCACACCTATCACAACCAGTCCAACCAATTTCGACCCGGTGCCCGAGTATGTAGGTACTGGGACGAGATTGTTTCTTTTGACTTGACT
GAGTTCTGCCAGCCTACGTTGGATTATTGGACATCAGATTTCCATTTGCAGCTTGACAAAGACGAGCAGCTACAGAGGGCTCAGATTGCATTGGATG
CGCATAGTGACCCTGTTGAGCCTTTCAGCAGGGACATGGCCATATTTCATGCATTTGAATCGATCAGACAAACAGCCCAAGCGGTCGATATAACTGTC
ATGAAGATTGAACATTTGATCATCATGGCATGCATATATCGTTCGAAGATGGCATGCCCTACTGTAGGCACCTCAATGATTGTCAAGTTCGATTTGTCA
AATCAGAGAGTGAGGTTTGGCGTTGCAAGAGGAACGTATCCTGAGTCAGATGGCAGGATGTGGTGGTATCTGCACGATGATTGCATGATCCACACG
GGTAGGCCGGAAAAGCTTCACACCGCTGGGGTGGAAGCGCCGTTGCCTGTCAGCAGTCGATTTTCCTTTCGTGCAACTCACCGCCAGGCCTGTGAA
ATGGCCGACGGTTTTGAGAAAACTTGGAGAAAAGACCAAAGATCCGTTTGCAAAGACAAGGGTGAAAACCACAATCATCATCCCCTTTTCTGCGGA
TCAACTTTTGACGAGAGGCACGATGAGGTATTCGTGTCCGCTTACATATGGTTCTACATCCAACTCATTTGCACGTTTTATTTCTCCTTTTCCCGGCCT
AATCTGCGGCGAAGACCTGACGGTCACACAGAAGGGGGAGAGTATCCCGTTGCGGATGAGAGTTTTTCAGCCTCATCTTCCCTCCTAGTGTGCAAG
TTAGGGACTAGAGGTGACCAAGTTCCTGTTGATTATTACGCGAACGTGGCGGCACACTATGGTGTCCGTACACATTCCCATGTGTATAAAGACGTAAC
AACAGACGATCTTGAAAAGATGAAAGTCGGAGACTTTTCATCTAGTTCCGCTGGTTACCTGAATCTTGCGTATGCTTCTCAACTTGGTTACAAGAGA
CTGTTCACTCCGCATGTGGAGGTGGAAAGGTCTCAAGGTGTAAGTTATACTTTATCACCAAGCTCAAAGTGGGTGGAAGCAACGAGGTTCATTGCC
GATTGGTCGAAGGTTTCCTGGTCCAACTTTCTCCCTGCGTGGATAGCCACGAGGGTCACTGCTGTGTATGTGCCAACTTTCAGAATTGGTGCGCTGT
CCGGAAGCGACATCCCGCGTTCTCACGATGGGTTAAACTTATTGGAGAAAGTTAAAAACACAGCAGAGTATGAGGAAGGTTGGACATCCGGCAGCT
CGTCAGTTTCAGTGATCCCTGACGAGATCAAGTCGAGATGCCCCGAAATACCATCGGGTAATCATCTTGAAATATTGAAGGATTACAAAACGGTGCA
CATCCACGGGGGGGCCGGCACTGTGCAGACCGCCATTGCCTGCGGTGCACGACCGGTTATCCACGACAAGACACTCGATAGAGTGTATCATAGGAT
GCCTGAACCAAAGGATTTCAAACAACCCTCCATTAGCAAGTTTATGGGTTGGTTGGTGTCTGCTGGTTTCAAAGTTGACGCGCCAATTGAGGTGAG
AGCGTGGTGGTTGTTACAATGGTATTGGTCCCAAAAATGGAACTATGCGTACGACGCATTAGTTACACTTGCGAAAGCCTATACTATATGTTCATATGT
ATATAACCATTGGGAACTTTTCCTCATTCTTTTCTTTACCGTTCCAGCAGTGATCTGGAGGATGCTTTTCTCTTCCAAGTCATTGTTCGCCATAGTCAA

AATCTGTGCGCTTGGTTTTTGGCGCTACCCTGTGTTATGCTTGCTAGATTCGAAAGTCACGTTTGTGGCGGGCATCTGGTCAATGCAAAACTTGTGGA
TGGGCGCAGCGCAAGATTTCGCGGCACTTATGGAGACACGTTCCTCACTGGTTTGGGAGCCAGTGCACAAGATCGTGGGAACGAAGAAGCTGACA
TTCCCTTTTCCCCTTGGGCATTGGAGCATGAAAGATAACCAAACTGGTAAGATCTACGAAGGTAGATTTATTGGTCCCTCCGAGACTATCGGAGGGA
ACTTTCGCCTTGTGAGCACTACTCGTGAATACAAGCGTGGATTT

>Cb_clean_contig_2550
GTGAACTCAAAGATCACATCAACCAGTACTTGGAATCTACAGATGTCAAGACAGTAGATCCTGTGTTGGTCGCGGCGATCGCAATGAGTTTTAAGTC
GAAATTCAATTGTCCAAAAGAAACCAGCTCTTTCGCAATTGATTTCAGACTCGACCGAAGAATGGTTGTTTTCTGTGCCACAGATTCCCCTGAAGAT
AAGAACAAGGGGAAACTTTACTGGTGCATCGGTGGTAGTGTTATCGTCCATGGCGGGTTGTTAGGTATTGAAGATGGGAAGTCTTGTTCCCCATCGT
TAGATCACTATAGCAAGTTATGGACGATGCCACTTCCGAAATCAGGTTGGGTGGACAGATCTTTTGAAGCTGAATACTTCACAAAGATTTCAACCTG
GCAAAAGGCCCTCGATCGGTGTGAACCGATCGTTGTATTCTCTGAACACACATTGCAACGCACACTCTACCAGTATTTTGATGGTGTGACAGACAAA
GTTTTGTCCCCATCACATACTGCAATCGTTGCTTGTGTGGTAAAATCCTTGATGAAATGCCCGGAGAACGGGGTAGGATATGCAGTACAGTTTGATAG
CCATGGGGCTTCACTGTACGTATGCAGGCAACCGAATTCCGCAGAGGCGAACCGGTTGTGGTACTTTCATTGGGATTTCCCTTTCGTGCACGGGGGA
AGAAAATCTTATGCTCCAGAGCCCAGGCGGTTCTTTTATACTGAGCATCTCAACATAGATTATTCCATACCCATGCATGAGGTTTTTGTTGGAAAGGAT
CTACCTAAATCCAATTCTTCTCATTTGACTGTCAAGAGTCAACAAGAATTGTATTTCGGTTTTCAACAGCAAATTGATATGTATGATGCGCCAACACCG
GCCTTCATGCATTCAAGAGCACATGGCACAGCTGAGCAAGCCATACGTGACACAGTGCCCAAGATGAGAACCTCTCAGTTCAATTTTGAGCACGTT
GCAGCGATAGCCATGTGTTATAGATCCAGAATGCAATGTCCATGCCCGGAATCAGCCATGGCTGTTGATTTTGATTTCGCAAATGAGCGCATTGTGTT
CTTTTCAAACCTAGGCAGTGGGTATTTCCGCTGTCGAGGCCAAATGTGGTGGTGTATAAACGGAGTCGGTCTGATCCACACTGGATCAATATTCCACA
CCTACCACAACCAATCAAACCAATCTCGACCTAGTCCCCGAGTGTGTAGGTACTGGGACGAGATTGTTTCTTTTCATTTGACCGAATTCTGCCAACCC
ACGTTGGGTTATTGGACATCGGATTTTCACTTGCAACCTGACAAAGACGAACAGTTGCAGAGGGCTCAGATTGCTCTGGATGCACATAGTGACCCTG
TTGAGCCTTTCAGCAGGGACGTTGCAGTATTTCATGCTTTTGAGGCGATCAGACAAACGGCCCATGCGGTCGATATAACTGTCATGAGGATTGAACA
TTTGATCATCATGGCGTGCATATATCGTTCGAAAATGGCGTGCCCTACAGCCGGCACTTCAATGATTGTCCAGTTCGATTTGTCAAACCAAAGAGTGA
GGTTCGGCGTTGCAAGGGGAACACATCCGGAGCTGGATGGCAGAATGTGGTGGTATCTGCACGACGATTGCATGATCCACACTGGTAGGCCAGAAA
AACCTCACGCCAATGAGGTTGAAACTCCATTGCCTGTCAGCAGTCGGTTTTCTTTTCGTGCAACTCATCGTCAGGCCAAGGTAATGGCCAACGATTT
TGAAGAGAAGTGGAGAGAAGACTCCAAGTGCAGAGCACGGACATGGCACATCGAAGGTGATGACACACCGAAGCAAAAGCTTGCTCGGTCCCAT
CACCCTTTGTGGTGTCCGGACACGTTCAACGAGCGGTTCGACGAGATCTTCGTTGCCGCTTACATTTGGTTTTTCTTTCAACTCATTTGCACGTTTTA

TTTTTCATTTTCTCGACCAAATCTTCGGCGAAAACCTGACAAGCACACGGAAGGAGGAGAGCACCCTCTTGGAGATGAGGGTTTTTCTGCATCATCC
TCCTTGCTCGTGTGCAAGTTAGGAACCAGAGGTGACCAGGTCCCTGTCGATTATTACGCGAACGTTGCTGCACACTATGGTGTCCGCACACATTCGC
ACGTGTACAAAGATGTGACGACAGACGATCTTGGAAAGATGAAAGTTGGAGACTTTTCATCTAGTTCCGCTGGTTATTTGAATCTTGCATACGCTTC
ACAACTTGGTTATAAAAGAGTGTTCACCCCGCATGTGGAGGTAGAGCGGTCCCAAGGTGTGAGCTATACTTTGTCACCGAGTTCGAAGTGGGTGGA
AGCAACAAGGTTCATTGCTGATTGGTCAAAGGTTTCTTGGTCCAATTTCCTCCCCGCGTGGATAGCCACGAGGGTCACTGCTGTGTATGTGCCAACT
TTTAGGATTGGTGCGTTGTCTGGAAGTGACATCCCGCGTTCTCATGACGGGCTGAACCTGCTGGAAAAGGTAAAGAACACAGCGGAGTTCGATGAG
GGTTGGACATCTGGCAGCTCGTCAGTTTCAGTAATCCCTGACGAGATCAAGTCAAGATGTCCCGAAATACCATCGGGTAACCATCTTGAAATATTAAA
GGATTACAAAACGGTGCACATTCATGGTGGAGCCGGTACTGTGCAAACTGCTATTGCTTGCGGCGCACGACCGGTCATCCACGACAAAACACTCGA
TCGAGTGTATCATAGGATGCCTGAGCCAAAGGATTTCAAACAACCTTCCATCGGGAAGTTCATGGGTTGGTTGGTATCCGCCGGTTTTAACGTTAATG
CACCAATAGAGGTAAGGGCGTGGTGGTTGTTACAATGGTATTGGTCCCAAAAGTGGAACTATGCGTACGACGCATTAGTTACACTTGCCAAAGCTTA
CACCATATCATCGTATGTGTACAATCATTGGGAGCTCTTCCTCATTCTTTTCTTCACCGTTCCGGCAGTGGTCTGGAGAGTGTTATTCTCTTCCAAATC
ATTGTTCTCCATAGTAAAAATTTGTGCGCTTGGTTTCTGGCGCTATCCTGTGTTGTGTTTGTTAGATTCGAAAGTCACGTTCGTGGCAGGAATCTGGTC
AATGCAAAACTTGTGGATGGGCGCGGCGCAAGATTTCTCTGCACTTATGGAGACACGATCTTCACTGGTTTGGGAGCCAGTGCAAAAGACCGTAGG
AACGAAGAAGTTGACGTTCCCTTTTCCCCTTGGGCATTGGAGCATGAAGGACAACCAAACTGGTAAAATCTACGAAGGTAGGTTTGTCGGTCCCTC
CGAGACTATTGGAGGGAATTTTCGCCTTGTAAGCACTACTCGTGAGTACAAGCGTGGGTTTAAGTCTTTTCCTGCCCCTTTCAATGTTGGCCATGCCG
AGTCTCTTGTGAGGGCTCAGCACGTGGCACCATATTCATCTTCGCATAATTGTGTGACTATGATGGTGCAGCTTTGTGCCAAGAGATCGACTGCCTGG
ACAGCATGTATGGTAATGGTAGGTGCCTTAACGGGGCTTGCCCTGGCGCCGCCAAAACTGCTCAAGGACATTCTTTCCTTTCTTTTCCCTGGCAAGG
ATTTGCCAGTACTCCTTTTTATCGTTCGCTTGGCTTTGCCGCATCGTTAGAAAACATCCCGTTTGAAATAGAAGGGATGGTCGAAACGACAGAGCCA
ATGCCAGAGACAGCTACACCTGAACCAACAGATCTGACGGATCCAGATTCTTTCAATATGTTGGTCGACGAGATAGCGTTCATCCAGGCCTGCGTGG
AACAACTCAAGATACCTACCTTGGACAAGGAGGCGTTTGATGAGGTTTCACAGCGAACTTTAGAAAAGGAGTTGGACAAGGTCGAGATCCCTGAA
GATTCGTTAATCAGGGTTTCCAACATACCACCGAGGGTGCACCATGAGTGGGCACAATTGGTGGATGGTCTCCACCATGCGATCTCTTTCGTGCGCA
CAAACCGCGTTGCTGACGCATTTGTATTGTACGTTAAGAACGCTGGTGAAAATATATATGACTTTGTCCAGCCGATACTGGAAATCCTTTCCTTCTTTC
TGCACCTTGCAGCTGAGCACTCTAAGCATGCGTTCGATCGCCTGTTTAAATGGCTTTGCGATTTCTTAGACCATGTGTGGGGTCTAGAAGCGTCAAA
GAGAGTTAAAACAGTGTGGGGCCTAACTGGCCTTCATCGAACGGGATTGATTGGTGTACAAGCCAAACTTGCAGCTAATGTTGCTTATTCTGAGTTC
GTAGGTAGACAAGATATCGAATCAGATTCAAACAAG

>Cb_clean_contig_3446
ATGACGAAGGCTTGCTTTTCTTTTCAAAGTTCCACAATGATAACACGAAACCAGATGTTGTGTTGGAGACTTTGGGTTCGTCACCCTATCACAAGCC
AGATAGGGAGACAGTGACGAGAGTGGCTTTTGTTGATGTAGAGAGACCTGTCGCATCCAACCCTACGGCTCAGTCTTCCACAGTTGTCCCGCCTCCT
CCGCCTCCACCGCCGGTGCAGGAGGTCAAGCAGAAGAGGAAGAAGACAGAGTTGGCTGATGGTGACACGTGGCTTCTTGCTGAAGGTTCTGAAG
GCAAGTCAAACAGAGCTCGTTTTAGATCTCTCTTCCCACATGATTGGTGGTTGAGGGTGTCTGAGAGGGTTATGCCTTTCATGAATCAGGATGCGTCT
CGTGACGTCGGGTACAGATCCAGATCTTGGTCTCAATCTTCCCTGGTTTCAAATCCTTCAGAGAAGTTTGATATAAACACGGTGAGGTATACACCTTC
TAGTTCTTCCAAGGCATCAGGTTCGACGCCTTTGCGGGTTGATCCTGAGGCTTTCAAGAGACAGCTTCAGGCTAAGGCTAGGAAGGTGAGTGCAAC
GTCGTCCTCCCGTCCCGATGCGAGGGAGGTGTATCTGAAGGGTGTTGGTGAGTTCAAACCGGATAAGGTTTCTCACAGAAAAGCCCTTTCTGAACC
TGCTCCGTCGAGGAAACCTTCTTCTGAGTGGATGTCTATTTCTCGAGCGTCTTCTGATTATCTTTTCGATGATGATCATGAAGACGCAAGGGATTCGA
CTAAGTCAGACATTGAGACCAACCCCGGGCCTTCAATCAGACATTCCCGTTCCAAGTCGACCTCGGATTTGGAGGGTGAAACACCGTCGTTAAATTT
TACACCACCCACCCCTGGTGATGTCTTGACTGTGCACCATCCAACACCGCCTCGTTTGAGGGTCAATCACAGGATGAATAGACACACTGTGATTGTA
CCTTCGCCGGGTGAAAACACGTCGAATTGGAATGTGGGTTACTCGTCATTTGCGTGGCCTCCAACAGAGGAGCATCTGGAACCTCGAGACTCCAGA
ACCGCGGATATAGAGGAAAATCCCGGACCATGCGAAACCCTTGACCCC

>Cb_clean_k141_29346
ATCGTTGCTTGTGTGGTGAAATCCTTGATGAAATGCCCAGATAACGGGACAGGATATGCAGTACAATTTGACAGCCATGGGGCTTCATTATATGTATGT
CGTCAACCAAATTCCGCAGAGGCAAACCGACTGTGGTACTTTCACTGGGATTTTCCTTTCGTGCACGGGGGAAGAAAATCTTATGCTCCAGAACCC
AGACGGTTCTTTTACACTGAGCATCTCAATATAGATTATTCAATACCCATGCGTGAAGTTTTTGCTGGAAAAGATCTGCCCAAATCCAGTTCTTCGCAT
CTGACTGTCAGGAGTCAACAGGAATTGTTTTTTGGTTTTCAGCAACAAATAGATTTGTATGATGTGCCGACACCGGCCTTCATGCACTCAAGAGCAC
ATGGCGTGGCTGAACAAGCCATTCGAGACACAGTGCCCAAAATGAGAACTACTCAGTTCAGTTTCGAGCAGGTTGCCGCGATAGCCATGTGTTATA
GATCTAGGATGCAGTGCCCATGTCCGGAGTCAGCTTTGGCTGTCGACTTTGACTTCGCCAATGAGCGCATTGTTTTCTTTTCAAACCTGGGCAGTGG
AAGTTTCCGCTGCCGCGGTCAAATGTGGTGGTGTATAAATGGAGTCGGACTGATACACACTGGGTCCACATTCCACACCTACCACAACCGGTCCAAC
CAATCTCGATCCAGTCCCCGGGTGTGTAGATACTGGGACGAGGTTGTTTCTTTTGACCTAACCGAGTTCTGCCAGCCTACGTTGGATCATTGGACGTC
GGATTTCCATTTGCAACTCGACAAAGACGAACAGCTACGCAGGGCTCAAGTTGCATTGGATGCGCATGATGACCCTGTTGAACCTTTCAGCAGGGA
CATTGCCATATTTCATGCGTTTGAATCGATCAGACAAACAGCCCAAGCTGTCGATATAACTGTCATGAAAATCGAACATTTGATTATCATGGCGTGCAT
GTATCGTTCAAAGATGGCGTGCCCTACAGCAGGCACTTCAATGATCGTCCAATTCGACTTGCCAAACCAGAGAGTGAGATTTGGCGTTGCAAGAGG

AACACATCCCGAGCTAGATGGCAGAATGTGGTGGTATCTGCACGACGATTGCATGATCCACACAGGTAGACCGGAAACGCTTCACACCGATAAGGT
GGAAGTGCCGTTGCCCGTTGGCAGTCGGTTTTCTTTTCGTGCAACCCACCGTCAAGCCTGTGAGATGGCTGACGGTTTTGAGAAAACTTGGAGAAA
AGACCAAAGGTCCGTTTGCAAGAACAAGGGTAGAGATCACAAGCATCATCCCCTTTTCTGTGGGTCAACTTTTGACGAGAGGCACGATGAAGTGTT
CGTGTCCGCGTACATATGGTTCTATATCCAACTCATTTGCACGTTTTATTTCTCTTTTTCTCGTCCTAATCTGCGTAAAAAACCTAATGATCACACGGAA
GGGGGAGAGTATCCCGCAGTAGACGAGGGCTTTTCTGCCTCATCTTCCCTTCTTGTGTGTAAGCTGGGAACCAGAGGTGACCAGGTCCCTGTTGATT
ACTATGCAAATGTGGCGGCACATTATGGCGTCCGCACACATTCACATGTGTACAAGAGCGTAACAACAGATGATCTTGAAAAGATGAAGGTCGGAG
ACTTTTCATCTAGTTCCGCTGGCTATTTGAATCTAGCATACGCTTCTCAACTTGGTTACAAAAGAGTTTTCACCCCGCACGTGGAGGTGGAGAGATCT
CAAGGTGTGAGTTATACTTTGTCACCAAGTTCAAAGTGGGTGGAAGCAACGAGATTCATCGCTGATTGGTCGAAGGTTTCCTGGTCCAACTTCCTCC
CCGCGTGGATAGCCACGAGGGTTACCGCTGTGTACGTACCAACTTTCAGAATTGGTGCATTGTCCGGAAGTGACATCCCGCGTTCTCATGACGGGTT
GAACTTGTTAGAGAAGGTTAAAAACACAGCCAAGTATAATGAGGGTTGGACATCTGGCAGCTCGTCAGTTTCCGTGATCCCTGACGAGATCAAGTC
AAGATGCCCTGAAATACCATCAGGTAACCATCTTGAAATATTAAAGGATTACAAAACGGTGCACATCCATGGCGGAGCTGGTACCGTGCAAACCGCC
ATTGCTTGCGGTGCACAACCAGTCATCCACGACAAAACACTCGATAGGGTGTATCACAGGATGCCTGGGCCAAAAGATTTCAAGCAACCTACCATC
GGGAAGTTCATGGGTTGGTTAATATCTGCAGGTTTCAAGGTCAAAGCACCAATTGAGGTAAGAGCATGGTGGTTGTTACAGTGGTACTGGTCCCAGA
AGTGGAACTATGCGTATGATGCATTAGTTACGCTTGCGAAAGCCTACACCATATCCTCATATGTATACAACCATTGGGAGCTCTTCCTCATCCTTTTCTT
CACTGTTCCAGCAGTGGTTTGGAGAATGTTGTTTTCATCAAAATCACTGTTTTCCATAGTCAAGATCTGCGCATTAGGTTTCTG

>Cb_clean_k141-6204
GGTGCTCTTACACAGGCCCCTCAGGGGACATATATAAGAGATGATTGCTCCACGCTTTCAGTGGATTCCATTTGTCAAAGGTGGTTTTCCTTTGTCTG
GAAGCAGATAATTTCTGCAATCGGATCCTTGCGGATCGCGGCACGTGCTATGCCGAGGCCTCTGTGAGGCCGTTGCCCTTTCCGGGCATTGTCAATT
GTGACGAGGTGCGGTGCACCGAGCTATCGCGTCTACGAGTCCGTAATGACTGTAAACTATTACAACAGGGCGGTGTCACACCCAAATGTGTATAAAC
TTGCGCGTTTCGGAGCGTTCTCAAACGCTGGCCTACACTGGGTAAACGGATGGTGATCAGAACGGCGATGAGGTAACTGTGGGCTAGTGACCCTAG
AAGTTATCCACCTCATTGTGAACCATCTTCCCTTTAAGATTTAGAAATGAGCACCGCTCTAAACGGTTAACACCATTTTGTTTTCGGCTTCCCTCTGGA
CGGGACGATTCAAAATGTCTTCTATCAATTTTCTTCGTTTGGATCAACGCAAAGATCCCGGGAGGCTCCGAGTGCCTAAAGAACGGTCTGCTATCCG
TTCATCCTATTCAAGTTCAGCCGGGACTTGGAGGCTCCGCAGGGAGTCGCTGCCGGCATGGGCCCAGCCCAAATCTTCTCCTGTCGGTCTTCTCGCT
TGTGGTGAGCTGACCAGTACTTCTTCTTTCGGCGAGGTGGTTCAGTCCACGCCTCGCCCAGCCACCATGGCTGCCAAGGTGGACGTTTGTCAAGTG
AGGTACTTGACATTCTCCGACTCGTCTCAAGGAATAAAACACTGGTTGATTAAAAGCCTGCCTGTCTCTCACCCTGATGTGTTAGAGATGAGATTACA

TGGCTTTGTCGATCGCCTGGCAACAGTGAGCTCTCAACATTCTGCTTGCAGAGTTGAAACTGAAAGTGCACTGTTTTCCCCATCCCGGACTTCTGGT
TACATGCAGAAGTTGATGGATGGAGTGTTGGCCCTTCGTCGCTCTGGTCAGATGACCTGTGTCAAGAAGGAATCTCCGGCTGAGTTCGCGCGCCGTA
AGCGTGAACAGAAGTGGTCTTGGCTCATCAGGCGTCTGCAGAGGCGTTCAGCTGCTGAGCAAGCTGTGATCGCTGAACAGCGGTTGACAAAGGCC
CTGCATTTGCAGGCCAATACTTCTTCCTTCGTCCCCACTGGGCCGAAGGGTTTCAAAGGTCTGGGAGAGTTGGTACTCCCTACCCCTTCTGCTGAAG
AGATCGCTTTTGGTTCCTCCTTCCTAGATGGCAAACGTGTTCGTGTTTTGCCAAGGAGAGAGAAGAACACTCGGTTTTCCGCTTGGTGCCAGTCAAA
TGCCGAACGTAAGAAGGCATTAAAACTAGCCGAGCTGGAACGTCAAAGGTTTAGATTGTCTTCGTTGGTCGCTGATCCTCTTCGTGTGCTCGGATGT
CAAATTCCTGAGACGTTTAGTGGTGTCATTTCTTTCGCCCGTGGTCTTTTTGGCACTTCTTCACGTCGCTCTGTCGTCCGAAGGGATTTCATCCATCG
GGCTAGACACGCTGAGAGGTTCTTCTTCTGTCCTGTCAATGGTTACATTATTGATCTGGTCGGGAACAAGATAAGGGTGTCCAGAAATAGGGCGGGA
TCTGGTGTCTCTTTTCGCATTGATGGTGGTCAGGTCGCCCACTGCGGCCTAAAGGACGAGTATCTTTGTGCCGTTCTTCAGTCCAGGCTAAATCTCAT
ACCAAACCCTGGCGAGCCGGAGCTCCTGACTATCGAACTGTTTCGTTCGAGGTACGAGGACTATTTCGGGAGGTTTTTGACTGAAATGGACGGAAC
CAACCTCGACTTGTGGTCTTTAATGACCATGGCCAAAAGGTGGAAAAGAGATTTTGGTTGCCCTGGCAACACTTGCATCTTGTCTTTTGCGGGTGGT
TCGGTCTCGTTTACTGTTGTTAACGAACAGGACGATCTGCCGCCGGCTTTGTGGTTTCGCCTTGATGGCAAAGATTTGTTCCACTGCGGCGTTGACC
AGGTCAAGAGACTGCAGTTCCAGAAGAACATCTGGAAAGCAGTTGCCAAGATGGTCATCCAGGACGGTAGCCCTGCTGAACAGCGGTTGGCAGAG
AAGGTCTCCTCCTCCGCACACGTGAGTGTCCCTTGCCTGAAACCTAACTTGTTCCACCAGTTAACAAACGGTTTCAGGGTCTGCGCCAAGTTCTCCG
GGCTTAGCGTTTTTGAGCGCGGGCTCGACTTTACACGCCCTGATCCTCGTGTCGAAGGCGGCTTCATCACAACCAGACAGGAGAGGTTGTTTTGGG
ACGTAGGGGCAAGCAGGTTGGTGTTCACACCAAAAGGCCTGGCAAAGATATTCTTCCGCAAGCACAATCAAGAAAAATGGAGGATAGCTTTGGCTG
CTGCGATTGGTTTGACCTATGACGAAGGTCTGCTTTTCTTTTCAAAGTTTCACAATGATAACACGAAACCAGATGTTGTGTTGGAGACTTTGGGTTCG
TCACCCTATCACAAGCCAGATAGGGAAACAGTGACAAGAGTGGCTTTTGTTGACGTGGAGAAACCTGTCGCACCCAACACTACGCCTCAGTCTTCC
ACAGTTGTTCCGCCTCCGCCGCCTCCACCGCCGGTGCAGGAAGTCAAGCAGAAGGGAAAGAAGACAGAGTTGGCTGATGGTGACACGTGGCTTCT
TGCTGAAGGTTCTGAAGGCAAGTCAAACAGAGCTCGTTTTAGGTCTCTCTTCCCAAATGATTGGTGGTTGAGGGTATCTGAAAGGGTCATGCCTTTC
ATGAATCAAGACGCATCTCGTGACGTCGGGTACAGATCCAGATCTTGGTCTCAATCTTCCCTGGTTTCAAACCCTTCAGAGAAGTTTGATATAAACGC
GGTGAGGTACACACCTTCTAGTTCTTCTAAGGCATCAGGTTCAACACCTTTGCGGGTTGATCCTGAGGCTTTCAAGAGACAGCTTCAGGCTAAAGCT
AGGAAAGTGAGTGCGACATCGTCTTCCCGTCCCGATGCAAGGGAAGTGTATCTAAAGGGTGTCGGTGAGTTCAAACCGGACAAGGTTCATCACAG
AAAAGCCCTTTCTGAACCTACTCCGTCGAGGAAACCTTCTTCCGAGTGGATGTCTATATCTCGAGCATCTTCTGATTATCTTTTCGACGACGATCATG
AAGACGCAAGGGATTCGACTAAATCAGATATTGAGACCAATCCTGGACCTTCGATCAGGCATTTCCGTTCCAAGTCGACTTCAGATTTGGAGGGTGA
AACACCGTCTTTAAATTTTACACCACCTACCCCTGGTGATGTCTTGACTGTGCACCATCCGACACCACCTCGTTTGAGGGTTAACCACAGGATGAATA

GACACACTGTGATTGTACCTTCGCCGGGTGAGAACACGTCGAATTGGAATGTAGGTTACTCGTCATTTGCTTGGCCTCCAACAGAGGAGCATCTGGA
ACCTCGAGACTCCAGAACCGCGGATATAGAAGAAAATCCCGGGCCAAGTGAAACCCTTGACCCCACTTTGCCCCAGTTCCGGGGTCGAAGTGGTTC
ACGGAGTTCAATCGCTGTTGTCACAACGGAGCGATCACCCTCTAGAACGCGTAGGTTCTTTGGAAAGATCGGCATTCACGGCAAGAGCGCGACGGA
AGAAGTCAAGGACATTTTTGGATTCAAAGAACATGACGAGGTGTATCCTTACACTGGCAAGAACTCATCGTTCGTGGAGAAGCATTATGAATTCCAC
TATGTCCTTTCTCATTTACCTAAGGACAAGAAGTGGCGGTCGTCAACCTTCATCAGGTGGGTTGCCGCTGCTTCTCTGTCCGAGCTGCAAAAATCAC
TCGACTGTGTAGTAAAGGCTTCACATCCTTTCAACACCGCACTTGTGAGTAAGCTGCGTGATGAGACGAATTCCTTCTTTCAGAAGATCTCCGATTCT
GAGCCGATAGGTGGTACGACCGTATCCGCCTTAGCTCACACATTCAAAACCCTGTACGCCTGCCCTAACAATGGGTGCGGTTATGCACTAGAGATAA
AGCCAGAAGATGGAATGGTATTATTCCATTTGACTGAGTCTCCAGACGAAACCGTAAAAAACAGGTTGTGGTGGGTTTTGAATGGGAACGAGATTTT
GCATGGAGGGGATAAAACCCCTGTTTCCCCAAGCACCGTCTCATTCGGTTGGCTAGCAAAGGAATACTCGACACATGAGGTGATCATCGAGCCCAA
ACCGGACTCTATGATATCACAAGAGAAACCAGGGCCGTTTGCAATGCCCTCGCTTTCTCAGTTTCAGAAATGTGTCGACAAGATTATTCCTTCTGGTG
GTCATTTCTCTCGTTCTAAAGTCGGCGAACTCAAAGATCACATCAACCAGTATCTGGAATCTACAGATGTCAAGACAGTAGATCCTGTGTTGGTCGC
GGCGATCGCGATGAGTTTTAAGTCGAAATTCAATTGTCCAAAAGAAACCAGCTCTTTCGCAATTGATTTCAGGCTCGACCGAAGAATGGTTGTTTTC
TGTGCCACAGATTCCCCTGAAGATAAGAACAAGGGGAAACTTTACTGGTGCATCGGTGGTAGTGTTATCGTCCATGGCGGGTTGCTAGGTATTGAAG
ATGGGAAGTCCTGTTCCCCATCGTTAGATCACTATAGCAAGTTATGGACGATGCCACTTCCGAAATCAGGTTGGGTGGACAGATCTTTTGAAGCTGA
ATACTTCACAAAGATTTCAACCTGGCAAAAGGCCCTCGATCGGTGTGAACCGATCGTTGTATTCTCTGAACACACATTGCAACGCACACTCTACCAG
TATTTTGACGGTGTGACAGACAAAGTTTTGTCCCCATCACATACTGCAATCGTTGCTTGTGTGGTGAAATCCTTGATGAAATGCCCGGAGGACGGGA
TGGGATATGCAGTACAGTTTGATAGCCATGGGGCTTCACTGTACATATGCAGGCAACCGAATTCCGCAGAGGCGAACCGGTTGTGGTACTTTCATTG
GGATTTCCCTTTCGTGCACGGGGGAAGAAAATCTTATGCTCCAGAGCCTAGGCGGTTCTTTTATACTGAGCATCTTAACATAGATTATTCCATACCCAT
GCATGAGGTTTTTGTTGGAAAGGATCTACCCAAGTCCAGTTCTTCGCATTTGACTGTCAAGAGTCAACAAGAGTTGTATTTCGGTTTCCAACAACAA
ATCGATCTGTATGATGCGCCAACACCGGCCTTCATGCATTCAAGAGCACATGGCACAGCTGAGCAAGCCATACGTGACACAGTGCCCAAGATGAGA
ACTTCTCAGTTCAATTTTGAGCAAGTTGCTGCGATAGCCATGTGTTATAGATCCAGAATGCAATGTCCATCTCCG

>Cb_clean_k141_45450
TCGTGGTTGAGGGGGAACATGTTCCCTAGCTACTACAGGGTACTTGACATCCACATGAACGTCGCAAAAGTAGATCCAGAACGACACGCTAAATTCC
TTCGTAAACTTGAGAAAGGTTGCCATCCAGTGTATGTCGTACGCTGTGCTTGTGCTGCGCCAATGGCGAGCGAGAGGAGAAGTGCTTTGGAATACAT
CGTCGTTCTTCGTCTTCACAACCACCACTGGACACTCTCCAGTGTTGAAGACCGCAGTTTCATTTCGGCGCTTTATATCTGCTGACGAGGCTTATCAT

CCTGTCACCAGATTACTTCTTTTTTGTTTAGTTCATTCTTATCAACCAGAGAAGAACTAGTACGCGTCATCACCATCTAGGACAGAGATTGGCAGTTC
AAAAAACTCATCATCACAACAAACATCTCAGGCTGTGACCAAATTTCTTCTCACCCACTCACCAAAAACCACGATGTCAAGCATCTCCGAGGACAG
CATCTACACGAACGGCGACAGCGCCAGCGTGAGCAATGGCGAGAATCCCGCCGATGATGAGCTCTTTCTCTTCACCTCAGAGTCCGT

> Cb_clean_contig_340
GTTCACGAACCGGAACGGCCGACGAGCACATGACCACAGAGAAGCACTGAACAAATAGCCACGCGTCCCGCGCCCAACACCGTCTTTCACCATCG
TTCTCTCGCGAATTCCCCGAACCATCCTCACCCTATGCAATGAGGCATGACCACGCCGAACGGATACCACAAGCCTCACGAACAGGACGGCCTCCAT
GATGACGATCGCGATTCGGATTTGGACCTCGACCTGAATGAGCTTGATCCGCATCCTGTCACCGACCGTACACCATCTCATTCGCGCCAGCCGAGTTC
TGGTCGCATCAGCCATGACTTTGCTGCGCGAATACCTTTGCGAAATTTGCGCTTTGGAGGGCGACGGAGACCGCGCAACGAAGATACAGAAGATCT
GGAAGCGCTGGTAGGGGACGATGAAGATGGACCGAAGCCGTCGACCGCCAGCTCAGGAGCGTCTGCATACGATGATGCGCCGCTGCTTTCACAGC
AGAATGGAGGCACGCGGAAGTTCTCGCAGCTCGGATCCAACCGTTTTGGCGACAATCGCCGACCAAAAGGCATCTGGCAATATGTGCCTTTCATGA
ACCGATCAACCTCCATACAGCTGCCGGCAATAAGTGAAGACGAGGCCGAAGATGATCATGATCCAGCGAGCGCCAGAAATATTGCTGTGGGGCAGA
AGCAACCGGCGCGGTTCCCAGCGAATGCCATATCGAATGCAAAATACACGCCCTGGTCGTTCTTGCCGAGAACACTCTTCAATGAGTTCAAGTTCTT
CTTCAACATGTACTTCCTACTCGTCGCATTATCGCAAATCATTCCCGCACTCCGAATCGGCTACCTTTCGACGTACATCGCGCCTCTTGCCTTTGTACT
CATGATCACCCTTAGTAAAGAGGCGTTCGACGATCTGAGTAGGAGGAAACGGGACAGAGAAGCCAACAGTGAGCCTTACAGAGTGCTGCGATTCG
ACAGTCCTGGCTTCTCCGATACGGACGCTAGCAGCAAGAAAAGGAAGATCAAGAGCATAACAGAGAAAAAGAAGAAAGGGAAGCGCAGAAGAGT
GTCAGCAGAAGATGCAAGGCTACAGCAGGCCGAGGACGAAGAGTACAGCACACAGAATGTAGACGCCCCTTCGTCAAACGTTGCTGAAATCATCA
AACCATCGGCCCAGCTCAAGGTTGGTGATGTGATTGTGCTCGAGAAGGATCAAAGAGTGCCTGCAGATGTGGTCATCCTGAAGAGCTTCTCGGCAG
AAACCGTTCAGACAGAGGCAGAACCTCGACAAGAGACGGTCGAAGAAGCGCTTGTGGATATGGGCGAGAACGCTGCACCTGTCAGCCCACGTCTA
CAACGTCAGTCTATTCAAGGTGTACAATTGAGCAACGAGACCACTTCATCCGATCCGAGTGGCGGAGGTGAAGCTTTCATTCGTACTGATCAGCTGG
ATGGAGAGACCGACTGGAAGCTGCGCCTGGCCACACCACTTGCGCAGACATTGCCCGCATCCGAGTATCTCAGGTTGCGAGTGACCGCCGGCAAA
CCAGACAAACGAGTCAATGACTTGGATTGCTCCACGCTTTCAGTGGATTCCATTTGTCAAAGGTGGTTTTCCTTTGTCTGGAAGCAGGTAATTTCTG
CAATCGGATCCTTGCGGATCGCGGCACGTGCTATGCCGAGGCCTTTGTGAGGCCGTTGCCCTTCTCGGGCGTTGTCAATTGTGACGAGGTGCGGTTC
ACCGAGTTATCGCGTCTACGAGTCCGGAATGACTGTAAACTATTGCAACAGGGCGGTGTCACACCCAAATGTGTATAAACTTGCGCGTTTCGGAGCG
TTCTCAAACGCTGACCTACACTGGGTAAACGGATGGTGATCAGAACGGCGATGAGGTAACTGTGGAGTAGTGACCCTAGAAGTTATCCACCTCATTG
TGAACCATCTTCCCTTTAATATTTAGAAATGAGCACCGCTCTAAACGGTTAACACCATTTTGTTTTCGGCTTCCCTCTGGACGGGACGATTCAAAATG

TCTTCTATCAATTTTCTTCGTTTGGATCAGCGCAAAGATCCCGGGAGGCTCCGAGTGCCTAAAGAACGGTCTGCTATCCGTTCGTTCTATTCAAGTTC
AGCCGGGACTTGGAGGCTCCGCAGGGAGTCGCTGCCGGCATGGGCCCAGCCCAAGTCTTCTCCTGTTGGTCTTCTCGCTTGTGGTGAGCTGACCAG
TACTTCTTCTTTCGGTGAGGTGGTTCAGTCCACGCCTCGTCCAGCTGTCATGGCTGCCAAGGTGGACGTTTGTCAAGTGAGGTACTTGACATTCTCC
GACTCGTCTCGAGGAATAAAGCGCTGGTTGGTTAAGAGCTTGCCTGTTTCTCATCCTGATGTATTAGAGATGAGCTTGCATGGCTTTGTTGATCGCCT
GGCAACGGTGAGTTCTCAGCATTCTACTTGCAGAGTTGAGGCTGAAAGTGTCTTGTTTTCTCCATCCCGGGCTTCTGGTCACATGCAGAAGTTGATG
GATGGCGTGTTGGCTCTTCGTCGCTCTGGTCAGATGACCTGTGTCAAGAAGGAATCTCCGGCTGAGTTTGCGCGCCGTAAGTGTGAACAGAAGTGG
TCTTGGCTCATCAGGCGTCTGCAGAGGCGTTCAGCTGCTGAGCAAGCTGTGATCGCTGAACAGCGGTTGACAAAGGCCCTGCATTTGCAGGCCGAT
ACTTCTTTCTTCGTCCCTACTGGGCCGAAGGGTTTCAAAGGTCTGGGAGAGTTGGTACTCCCTGCCCCTTCTGCTGAAGAGATCGCTTTTGGTTCTT
CTTTCCTTGATGGTGAACGTGTTCGTGTTTTGCCAAAGAGAGAGAAGAACATTCGGTTTTCCGCTTGGTGCCAGTCAAATGCTGAACGTAAGAAGG
CATTGAAACTAGCCGAGCTGGAACGTCAAAGGTTTCGATTGTCTTCGTTGGTTGCTGATCCTCTTCGTGTTCTCGGATGTCAAATTCCTGAGACATTC
AGTGGTGTCATTTCTTTCGCCCGTGGTCTTTTTGGCACTTCTTCACGTCGCTCTGTCGTCCGAAGGGATTTCATCCATCGGGCTAGATACGCTGAGAG
GTTCTTCTTTTGTCCTGTCAGTGGTTACATTATTGATTTGGTCGGGAACAAGATAAGGGTGTCCAGAGATAGGGCGGGGTCTGGTGTCTCTTTTCGCG
TTGATGGTGGTCAGGTCGCCCATTGCGGCCTAAAGGACGAGTATCTTTGTGCCGTTCTTCAGTCTAGGTTCGATCTCATACCAAACTCTGGCGAGCC
GGAGTTCTTGACTATTGAGCTGTTCCGTTCGAGATATGAAGACTATTTCGGGAGGTTTTTGACTGAAATGGACGGAACCAATCTCGACTTGTGGTCTC
TAATGACCATGGCCAAAAGGTGGAAAAGGGATTTTGGTTGCCCTGGCAACACTTGTATTTTGTCTTTTGCGGGTGATTCGGTCTCGTTTACTGTTGTT
AACGAACAGGACGATCTGCCGCCGGCTTTGTGGTTTCGCCTTGATGGTAAAGATTTGTTCCACTGCGGCGTTGACCAGGTCAAGAGACTGCAGTTC
CAGAAGAACATCTGGAAAGCAGTCGCCAAGATGGTCATCCAGGATGGCAGCCCTGCTGAACATCGGTTGGCAGAGAAGGTTTCCTCCTCTGCACAC
GTGAGTGTCCCTTGTCTAAAACCTAACCTGTTCCACCAGTTAACAAACGGTTTCAGGGTCTGCGCCAAGTTCTCCGGGCTCAGCGTTTTTGAGCGCG
GGCTCGACTTTACACGTCCTGATCCTCGGGTTGAAGGTGGTTTCATCACGACCAGGCAGGAGAGGTTGTTTTGGGACGTAGGGGCAAGCAGGTTGG
TGTTCACACCAAAAGGCCTGGCAAAGATATTCTTCCGCAAGCACAATCAAGAAAAATGGAGGATAGCTTTGGCTGCTGCGATTGGTTTGACCTATGA
CGAAGGTCTGCTTTTCTTTTCAAAGTTTCATAATGATAACACGAAACCAGATGTTGTGTTGGAGACTTTGGGTTCGTCACCCTATCACAAGCCAGATA
GGGAGACAGTGACGAGAGTGGCTTTTGTTGACGTGGAGAAACCTGTTGCGTCCAACCCTATGGCTCAGTTTTCCACAGTTGTTCCGCCTCCTCCGC
CTCCACCGCCAGTGCAGGAAGTCAAGCAGAAGGGGAAGAAGACAGAGTTGGCTGATGGTGACACGTGGCTTCTTGCTGAAGGTTCTGAAGGCAA
GTCAAACAGAGCTCGTTTTAGATCTCTCTTCCCACATGATTGGTGGTTGAGGGTGTCTGAGCGGGTTATGCCTTTCATGAATCAGGATGCGTCTCGTG
ATGTCGGGTACAGATCCAGGTCTTGGTCTCAATCTTCCCTGGTTTCAGATCCTTCAGAGAAGTTTGATATAAACACGGTGAGGTATACACCTTCTAGT
TCTTCCAAGGCATCAGGTTCAGTGCCTTTGCGGGTTGATCCTGAGGCTTTCAAGAGGCAGCTTCAGGCTAAGGCTAGGAAGGTGAGTGCAACGTCA

TCCTTCCGTCCCGATGCGAGGGAGGTGTATTTGAAGGGTGTTGGTGAGTTCAAACCGGATAAGGTTTCTCACAGAAAAGCCCTTTCTGAACCTACTC
CGTCGAGGAAACCTTCTTCTGAGTGGATGTCTATTTCTCGAGCGTCTTCTGACTATCTTTTTGATGATGATCATGAAGACGCAAGAGATTCGACTAAG
TCAGACGTTGAGACTAACCCTGGGCCTTCGATCAGGCATTTCCGTTCCAAGTCGACTTCGGATTTGGAGGGTGAAACACCGTCTTTAAATTTTACAC
CACCTACCCCTGGTGATGTTTTGACTGTGCACCATCCGACTCCACCTCGTTTGAGGGTTGATCACAGGCTGAATAGACACACTGTGATTGTGCCTTC
ACCGGGTGAAAACACGTCGAATTGGAATGTTGGTTACTCGTCATTTGCTTGGCCTCCAACAGAGGAGCATCTGGAACCTCGAGACTCCAGAACCGC
GGATATAGAAGAAAATCCCGGGCCAGGTGAAACCCTTGACCCTACTTTGCCCCAGTTCCGGGGTCGAAGTGGTTCACGGAGTTCAATCGCTGTTGC
CACAACGGAGCGATCACCCTCTAGAACACGTAGGTTCTTTGGAAAGATCGACATTCACGGCAAGAGCGCGACGGAAGAAGTTAAGGACATTTTTGG
ATTCAAAGAACATGACGAGGTGTATCCTTACACTGGCAAGAACTCATCGTTCGTGGAGAAGCATTATGAATTCCATTATGTCCTTTCTCATTTACCTAA
GGACAAGAAGTGGCGGTCTTCAACCTTCATCAGGTGGGTTGCCGCTGCTTCTCTGTCCGAGCTTCAAAAATCACTTGACTGTGTAGTAAAGGCTTC
ACATCCTTTCAACACCGCGCTTGTGAGTAAGCTGCGTGATGAGACGAATTCCTTCTTTCAGAAGATCTCCGATTCTGAGCCGATAGGTGGTACGACC
GTATCTGCCTTAGCTCACACATTCAAGACTCTGTACGCCTGCCCTAACAATGGGTGCGGTTATGCACTGGAGATAAAGCCAGAAGATGGAATGGTGT
TATTTCATTTGACTGAGTCTCCAGACGAAACCGTTAAAAACAGGTTGTGGTGGGTTTTGAATGGGAATGAGATTTTGCATGGAGGGGATAAAACCCC
TGTTTCCCCAAGCACCGTCTCATTCGGTTGGCTAGCAAAGGAATATTTGACACATGAGGTGATCATCGAGCCCAAACCGGACTCTATGCTATCACAA
GAGAAACCAGGGCCGTTTGCAATGCCCTCGCTTTCTCAGTTTCAGAAATGTGTCGACAGGATTATTCCTTCTGGTGGTCATTTCTCTCGTTCTAAAGT
CGGTGAACTCAAAGATCACATCAACCAGTATCTGGAATCTACAGATGTCAAGACAGTAGATCCTGTGTTGGTCGCGGCGATCGCGATGAGTTTTAAG
TCGAAATTCAATTGTCCAAAAGAAACCAGCTCTTTCGCAATTGATTTCAGGCTCGACCGGAGAATGGTTGTTTTTTGTGCCACAGATTCCCCTGAAG
ATAAGAACAAGGGGAAACTTTACTGGTGCATCGGTGGTAGTGTTATCGTCCATGGCGGGTTGCTATGTATTGAAGATGGGAAGTCTTGTTCCCCATCG
TTAGATCACTATAGCAAGTTGTGGACGATGCCACTTCCGAAATCAGGTTGGGTGGACAGGTCTTTTGACGCTGAATACTTCACGAAGATTTCAACCT
GGCAAAAGGCCCTCGATCGGTGTGAACCGATCGTTGTATTCTCTGAACACACATTGCAACGCACACTTTACCAGTATTTTGATGGTGTGACAGACAA
AGTTTTGTCCCCATCGCATACTGCGATTGTTGCTTGTGTGGTAAAATCCTTGATGAAATGCCCGGAGGACGGGGTGGGATATGCAGTGCAGTTTGATA
GCCATGGGGCTTCACTGTACGTGTGTCGGCAACCGAATTCCGCAGAGGCGAACCGGTTGTGGTACTTTCATTGGGATTTCCCTTTCGTGCACGGGGG
AAGGAAATCTTATGCTCCAGAGCCTAGGCGGTTCTTTTATACTGAGCATCTTAACATAGATTATTCCATACCCATGCATGAGGTTTTTGTTGGAAAGGA
TCTACCCAAGTCCAATTCTTCGCATTTGACTGTCAGGAGTCAACAAGAGTTGTATTTCGGTTTCCAACAACAAATCGATTTGTATGATGCGCCAACAC
CGGCCTTCATGCATTCAAGAGCACATGGCGTAGCTGAGCAAGCCATACGTGACACAGTGCCTAAGATGAGGACTTCTCAGTTCAATTTTGAGCACAT
TGCTGCGATAGCCATGTGTTATAGGTCCAGGATGCAATGTCCATGTCCGGAGTCTGCTATGGCTATCGATTTTGACTTCGCCAATGAGCGCATTGTGTT
CTTTTCGAACCTAGGCAGTGGGTATTTCCGCTGTCGGGGCCAAATGTGGTGGTGTGTAAATGGAGTTGGTCTGATTCATACTGGATCAGTGTTTCACA

CCTACCACAACCAGTCAAACCAATCTCGACCTATTCCCCGAGTGTGTAGGTACTGGGACGAGATTGTTTCTTTTCATTTGACCGAATTCTGCCAACCC
ACGTTGGGTTATTGGACGTCGGATTTTCACCTGCAACCTGACAAAGACGAACGGTTGCAGAGGGCTCAGATTGCGCTGGATGCACATAATGATCCTG
TTGAGCCTTTCAGCAGGGACATTGCCGTGTTTCATGCGTTTGAGGCGATCAGACAAACGGCCCAAGCGGTCGATATAACTGTCATGAAGATTGAACA
TTTGATCATCATGGCGTGCATATATCGTTCGAAAATGGCGTGCCCTACAGCAGGCACTTCAATGATTGTCCAGTTCGATTTGTCAAACCAAAGAGTGA
GGTTCGGCGTTGCAAGAGGAACCCATCCGGAGTTGGATGGCAGAATGTGGTGGTATCTGCACGACGATTGCATGATCCACACTGGTAGGCCGGAAA
AGCCTCACTCCGGTGAGGTTGGAACTCCGTTGCCTGTCAGCAGTCGGTTTTCTTTTCGTGCAACTCATCGTCAGGCCAAGGTAATGGCTAACGATTT
TGAAGAGAAGTGGAGAGAAGACTCTAAATGTAGGGCACGGACATGGCACATTGAAGGTGATGATACACCGAGGCAAAAGCTTGCTCGGTCCCATC
ACCCTTTGTGGTGTCCGAATACGTTCGACGAACGGTTCGACGAGATCTTCGTTGCCGCGTACATTTGGTTTTTCTTTCAACTCATTTGCACGTTCTAC
TTCTCGTTCTCTCGGCCTAATCTTCGGCGAAAACCTGACAGACACACGGAAGGGGGAGAGCATCCTCTTGGAGATGAGGATTTTTCTGCATCATCTT
CCTTACTCGTGTGTAAGTTAGGAACCAGAGGTGATCAAGTCCCTGTCGACTATTACGCGAACGTGGCGGCACACTATGGTGTCCGCACACATTCTCA
CGTATACAAAGATGTGACGACAGATGATCTTGAAAAGATGAAAGTCGGAGACTTTTCATCTAGTTCCGCTGGTTATTTGAATCTCGCATACGCTTCTC
AACTTGGTTACAAGAGAGTTTTCACCCCGCACGTGGAGGTGGAGAGATCTCAAGGTGTGAGTTACACCTTGTCACCAAGTTCAAAGTGGGTAGAA
GCAACGAGATTCATCGCTGATTGGTCGAGGGTTTCCTGGTCCAACTTTCTCCCCGCGTGGATAGCCACGAGGGTTACCGCTGTGTACGTACCAACTT
TCAGAATTGGTGCGTTATCAGGAAGTGACATCCCGCGTTCTCATGACGGGTTGAACTTGCTGGAGAAAGTTAAGAACACAGCAAAGTTCGATGAGG
GTTGGACATCTGGCAGCTCATCAGTTTCAGTAATCCCTGACGAGATCAAGTTAAGATGCCCCGAAGTACCATCGGGTAACCATCTTGAAATATTAAAG
GATTACAAAACGGTGCACATTCATGGTGGAGCCGGTACTGTGCAAACTGCCATTGCTTGCGGCGCACGACCGGTCATCCACGACAAGACACTCGAT
AGAGTGTATCATAAGATGCCTGGGCCAGAGGATTTCAAACAACCTTCTATTGGGAAGTTCATGGGTTGGTTGGTATCTGCCGGTTTTAAAGTTAATGC
ACCAATAGAGGTAAGAGCTTGGTGGTTGTTACAATGGTATTGGTCGCAAAAGTGGAACTATGCGTACGACGCATTAGTTACACTTGCTAAAGCTTAC
ACCATATCATCGTATGTGTACAATCATTGGGAGCTTTTCCTCATTCTTTTCTTCACCGTTCCGGCAGTGGTCTGGAGAATGTTGTTTTCTTCCAAATCAT
TGTTCTCCATAGTCAAAATTTGTGCGCTTGGTTTTTGGCGCTACCCTGTGTTGTGCTTGTTAGATTCGAAAGTCACTTTCGTGGCAGGAATCTGGTCA
ATGCAAAACTTGTGGATGGGCGCGGCGCAAGATTTGTCTGCACTCATGGAGACACGATCTTCACTGGTTTGGGAGCCAGTGCAAAAGATCGTAGGA
ACGAAGAAGTTGACGTTCCCTTTTCCCCTTGGGCATTGGAGCATGAAAGATAACCAAACTGGTAAGATTTACGAAGGTAGATTTGTTGGTCCCTCCG
AGACTATCGGAGGGAACTTTCGCCTTGTGAGCACTACTCGTGAATACAAGCGTGGATTTAAGTCTTTTCCTGCTCCTTTTAATGTTGGTCATGCGGAG
TCTCTTGTGAAGGCTCAACACGTAGCACCGTACTCATCTTCACATAATTGTGTGACTATGATGGTGCAACTTTGTGCCAAGAGATCGACTGCATGGAC
AGCATGTATGGTGATGGTTGGTGCCTTAACGGGACTTGCCCTGGCGCCGCCGAAGTTGCTCAAGGACATTCTTTCTTTTCTTTTCCCTGGCAAGGATT
TTGCCAGTACTCCTTTTTATCGATCGCTTGGCTTTGCCGCGTCGTTAGAAAACATCCCGTTTGAGATAGAAGGGATGGTCGAAACGACAGAGCCAAT

GCCAGAGACAGCTACACCTGAACCAACAGATCTGGCGGATCCAGATTCTTTCAATATGTTGGTCGATGAGATAGCGTTCATCCAGGCATGCGTGGAA
CAACTCAAGATACCTACCTTGGATAAGGAGGCATTTGATGAGGTTTCACAGCGAACTTTAGAAAAGGAGTTGGACAAGGTCGAAATCCCTGAGGAT
TCGTTAATCAGGGTTTCCAACATTCCTCCAAGAGTGCATCATGAGTGGGCGCAATTGGTGGATGGTCTCCACCATGCTATTTCTTTCGTTCGTACGAA
TCGCGTCGCTGACGCGTTTGTGTTGTACGTAAAGAATGCTGGTGAAAATATATATGACTTTGTCCAGCCGATACTGGAAATTCTTTCCTTCTTTCTGCA
CCTTGCAGCTGAGCATTCCAAGCATGCGTTTGATCGCTTGTTCAGATGGCTTTGCGGTTTCTTGGACCATGTGTGGGGTCTAGAAGCGTCAAAGAGA
GTCAAAACAGTGTGGGGCCTGACTGGCCTTCATCGAACGGGATTGCTTGGTGTAAAAGCCAAACTTGCAGCTAATGTTGCTTATTCTGATTTCGTAG
GTAGACAGGATTTCGAATCAGATTACAACAAGTTGGTGCAAGAAGGCAAGCACCTTGCTCAGGTGTATGGTGCCGTTAAACGATCAAACCTTGGAG
GGCCTCAGAGGAGGCCTATAGGTTATGGAAAACCTCTAATGTCGCAATCTGAAGCCAGTCTGCTCGGTTTCGAGTCAGGTGAGTTTGTGACTGATCG
TGAGTATGAGGCTCGTGTGCAATCGTACCTTGAACAGGGTGTTGCACAAGGTGCTGACGGGGTGTTTTTGGCCGATAAGAGACCGGAGCTGATTGC
TAAGTCACAGCACCGTTATGAGCCAAAGTATCCCGCACTCACTTCTGAGGATCGTTCCATGGCTAAGGAAATAGCCCAGGCCATGTTTGATCAATATC
CGGAAGTCTTCGCTAATTGTGATGTTTTGCCGCCGAGGGCAGTTCACAATTATGTGAAGCAAAAATATTCTCCAGGAACCCCGTTCATCAAACCTGG
GGGCTTCAAATCTCGTCAAGCGATGTTCGACGCTGGTTATGACCGCGTCATGCAACGACGAGCAATGCATTACTTGAAAACTGGTGAATATCCAGTG
CAGTTCTATCACGCTTTTGTGAAATCACAGGTGGTAGATATCAATAAATGCTTGAGCCCCGAGGAAGGTGGAAAAGGGAAAGATGTTCGCACTGTG
GTCAGCCAAGATCTGTTCTCCTATTACATTGACCAATGTCTCCAAATTGAGCGCAACAAGCGCATAAACTGGGATACATATGGTGCAGGTATAGGTAT
GCCTCTTAATCAGTCGATGGAGGCAATATACAGTAGAATGGCAGCTGCACAGAAATCGAGAGGAGGCAGGTACATCATTGCTGATGCAACCGCCTTC
GACTCATTTTGCAAACCTTTTCTCTTTGAAGTGAACGCAAATCTTTGGGAACTCGGCTACAAAGATCATCCAAGCGGCAACGGCAAGAACCTCGCAT
CTGTTCTAAATGCGTCGTATCAAGCCAGACAAAATGCGTGGATAATCGGTGTGACCGAGCCGGAATATGACTCGTTGACTGTTTGCGTCCAAGACAA
AAACAGTCGCGAGCTCATACGGCAACGATCCCCAAAGACAGTAATCTGCTTGGAAGATCTCATAGACTGGGACAAGTACAATAGATTGCCTGCTGA
G

> Cb_clean_k141_5852
ACCGAGCCGGAATATGACTCGTTGACTGTTTGCGTCCAAGACAAAAACAGTCGCGAGCTCATACGGCAACGATCCCCAAAGACAGTAATCTGCTTG
GAAGATCTCATAGACTGGGACAAGTACAATAGATTGCCTGCTGAGCAACGTTCGAACTACGTTGCAAAACTTCAACCACCTGAAGGGAAGACGGTG
GTAACCTGGGATCCTTCTCTCCGCCCATCCAGATCAAACTGGATGGGTAAATATGAATATGGTGTCTTATCTGATGTTGACAAGAAGTTCTTTGAACAT
CAGACTTTCACCTACGATCCTTCGAATTTTGAAGACATGTATCAAGACATATTATCAGTTTCACGATCAAACTATAAACTGATGTCAAACGTACATGCG
AAGAATCGTGGTGGAAGCACTGGAGGGTCAGATACATCAAACGTTAACACACACGCCTTCAAGGCAGGTGTGATTTACGCTTGGTGTCAGACTACT

GGCAGGGCTCCAAAGGAGTTCTTCCAATACAATGACCTTGCCAATACATCAGATGACACCATCTGGCAATCGGGTGGCAAACATGGCCTAAACACTG
TAGAAGATGTCGAAGTGTTTCGACATCATTGTCAATCAGTGGGCATTCATCTAGAGATAGAGACAACGAAGGACATATCACAAGTGGAATACTTGTC
AAAATTCGTTCGTGTCCCTACTGCAGAAGATTCAGCAGCATTGAAAGTCTGGAGATCCCAGAAAATCAAGGCTGCTTCAAATGCCCATAAACAATTA
GGCCTGACTGTTCCCAATTCTTGGGAGCAGTTCAACAACCCGCAATTTGTCGTCGTTCAAAACCCGTCTGCGATCTTGCTCCGAAGGTCTGCGTTTA
GGTATTACCAATCAAACGCAAATCATTGGAAATACGTTTCAGTGGAAAGAGGTGCTGGTCATGCTCAGAGCACCGCCTTTCTACCTGAATTGTATGG
CAAGTTCGCACAAGAGTGGTGCGACGACATCAACGATCTTCTGGTACAACAGAAGATCCATCGGAAGTTCAAGGTCACAAACGGCCAATTTGGACT
ACAGTGCGTCGAACAGGTGGACCCGAGAGCGTCGCAACAACAGCTCTCACCACGTCAAGTCGCTTTCCTCTCATGGCTGAGAGGAAATATGTTCCC
TAGTTACTACAGGGTACTTGACATCCACATGAACGTCGCAAAAGTAGATCCAGAACGACACGCCAAGTTCCTTCGTAAGCTTGAGAAAGGCTGGCG
AGGTTGGGAAGAACTGTTACGTGAAGGGGTAGATAACCTCTACGCCTTAACTGATTCCATCCCAGATGAAATCAGCAAAAAGTTCCAACCCGGCCT
CGACATGCTCTATGCAGAGCAACCATTCTACACGAAGAACAAGGTCGTTGAAAAGTTCGTTTACTTGAAACTTCTGGAAGAAAGCCCAGAGACAGA
GGTCACATTTTCAGACTTTTCCGCTCGGATTCAAGAGTCACCGTATGCGTCGGTGTGCGACCCCTATCACTTCTGGGAGAAGTTAGGGGAGCCTGAG
TTCAAAGAACAGTTGATGGCTGAAGATCCTCTGAAGATACAAGGTCTCGTTTTGCTTATAAGCACCATGTACCTATTAACAAACACGGCTGAGTCAG
TAGTGTTGGCCATTCCTTTTGTTGGTCTATTCTACAAACTCTTCCTGTGGTCCTTTATCGGACTCAATAAGGTATATGGACTGTTGAACACTATGTACTG
GCATTCCGAAGGTAAGTCTTCGAGGGAGATATCCCGAATTATGCCTCGAGATCCGTACATGGTGACGAAACAGTTCGCTGCGTTTTGCATCGACTTTG
TACCATCACAACTGGGTTACTTGGCCCTTATCCCATTGCACATATTGTCCTTACTTCCAGTGCTGTTTGAAGCACTTGGAAAAGCATGGTATGTGGCA
GGGGAGGTCAAGAATGTGATGGAACCCCCTGGCAGCACCGTAAACAAGTGGGATCAATACGCAGACGAGTACATACAAGCTCTGCGTTCGTCTCCC
ACGAACAGAAAATACGTTGCTGCCAAGACTTCAACTGGCAAATCGACCTGGTTTATAGCTGCCCTGTGGAAAGCTAGACACCGCCAGAAGGTCAGG
AAATTTTGGCTTGTCGAACCTCGATTGGTGTTGCGCGACAATACGCATGTACCATTCGATGCACCTTATCAAGTGCTCAAGGACAACATCCAAGTTTC
AAACACCCACGATATCCACATCTTGACGTATGGACATTTACAGTCAAGATTGTTGGATATAGATAATGAACGTGATATCGTCATTTTTGACGAATTTCA
CGAACAACAAGGTGAAATGATTCTTGGGCTTGACAAGTGCAAGGCGCCAATCTTTCTTCTCTCGGCCACGCCAGCCGAAATACCATCTCTGCACGG
TTCCGGAACTCTGGTTCCAAACATTGACAGACGACATCCAATCCAGATCCACCAAGTGGATGACGGAATGGGTGTTGTTGACATGTTCATGGAAGCC
AGAAACCGTTACCCTGATTTATGCAAGAGAGCGATGATCATTGTACCAACTCACAAGGAGGTTGCAAAAGTTATCGCTTCCCTTGATTATCTCAAGGT
AGGTAAGGTGTCCCCTCTGTCCGCACGTGATCGTGTGGTACAGGAGGAGGGAATACTGGTGTGCACCCCTTACGTCCAAACGGGTCTGGATATAAA
ACCACCACCAGATCTTCTGATCGATTGTGGCAAAGACTTCGTTTTGGATAAAGGGCGTCCAGTATTTCCACTCCCGTGGACAGATAAGGACATAAAC
CAGCAGAGAGTTGGTCGTGTTGGAAGATTAAGACCTGGAATCGTCTTTCAACCGAAATCAGCAGGTTCCGGAAGGAAAGGTGTGGTTTACCCGAG
CCCACATCTATTCCAACATAAAGCTGTCGCGGAACATTTTAAGGTTCCACAGCTTGCACCAATCTCGGGTGCTTTCACGGCAGATTTGCCTTTCTTGC

GACCGAACAAGAAAGTGTTAAATTCTCTACCCGTCGAAAAGTCAGTTGTTTTTATACATTCACTTGCTTTAAGTGGTGTTCGCCAGGAGCAATGGCA
ACACATGTATACGAGATCCGTGCAGGGGCACACTCTCGGCAACGACTACAAAAACGTAGACAGACTGCTTAATCATTCAAGGTACAAACGATCAAA
GCTAGTCTCGTATGGAGACGCACTTTATCATCTTTACCGAGAACATGCAGTCCAGTACGGATTTGTCAAGGAAGACGGAAGTACAGAATACAAGTCT
GCCAGGCCAATGTCGGTCATCAATGGAAAGTGGATAGAGTTAGAACAATCGCCGACGACTAAGATTGATATTCAAGAGTTACCACAACAGGAACTTT
CAAACAAATATCAATCTTTCGAATCATCAATGAGGCGATGTCGAGACTCCATTATTTTCAAAGCCAAGGACATGCCTGGGCATGACTTGGAACGTCTC
AACTCCATCTTAGCGTTTTGAGCACTAAAAGGCGGCACTTGTAGCTGAAATGCTACATTTAAAGACAACCGCAGTTTTGTGTTACCCAATTGACGTTT
GACTTTTGTCAGACACGCTATTTCTGTTGTTGCCTCCCGACCTCTTTCGTGACATTTGTCACATCCGTTCGTTACCTTTCTTTCTTTGTCCGTAAAACT
ACAAACAAACGCAGCGGTTAATGCCGCGGTAATAAACCGGATTCACATGCTAACGCATGGTGTGGTTGGCCGTTTGCCAACGTAATCACTAGGGTGG
TTAATTCCACCCTATGAGAAGGGAAAAAAAAAGAAAGGTAACGAACGGATGTGACAAATGTCACGAAAGAGGTCGGGAGGCAACAACAGAAATA
GCGTG

> Cb_clean_k141_14799
CAGACTACTGGCAGGGCTCCAAAGGAGTTCTTCCAATACAATGACCTTGCCAATACATCAGATGACACCATCTGGCAATCGGGTGGCAAACATGGCC
TAAACACTGTAGAAGATGTCGAAGTGTTTCGACATCATTGTCAGTCAGTGGGAATTCATCTAGAGATAGAGACAACGAAAAACATCTCACAAGTAG
AGTACTTGTCGAAATTCGTTCGCGTCCCTACTGCAGAAGATTCAGCAGCATTGAAAGTCTGGAGATCCCAGAAAATCAAGGCTGCTTCAAATGCCCA
TAAACAACTAGGCCTAACTGTTCCTACTTCATGGGAACAGTTCAACAACCCGCAATTCGTTGTCGTTCAAAACCCTTCCGCGATCTTGCTCCGAAGA
TCTGCGTTTAGATATTATCAATCTAACGCAAACCATTGGAAGTATGTCTCAGTAGAAAGAGGTGCCGGTCATGCTCAAAGCACCGCCTTTCTACCTGA
ATTATATGGCAAGTTCGCACAAGAATGGTGCGACGACATCAACGATCTTCTGGTGCAACAGAAGATCCATCGGAAGTTTAAGGTCACAAACGGTCA
GTTTGGACTACAATGCGTCGAACAAGTGGATCCGAGAGCGTCGCAACAACAGCTCTCACCACGTCAAGTCGCTTTCCTCTCATGGTTGAGAGGAAA
TATGTTCCCTAGTTACTACAGGGTACTTGACATCCACATGAACGTCGCAAAAGTAGATCCAGAACGACACGCCAAGTTTCTTCGTAAACTTGAGAAA
GGTTGGCGAGGTTGGGAAGAACTGTTACGTGAAGGGGTAGACAACCTCTACGCCTTAACTGATTCCATTCCAGATGAAATCAGCAAAAAGTTCCAA
CCCGGCCTCGATATGCTCTATGCGGAGCAACCGTTCTACACGAAGAACAAGGTAGTCGAAAAGTTTGTTTACCTGAAACTTCTGGAGGAAAGCCCG
GAGACAGAGGTCACATTTTCAGATTTTTCTGCTCGAATTCAGGAATCACCGTACGCGTCGGTGTGTGACCCCTATCACTTCTGGGAGAAGTTAGGGG
AACCGGAATTCAAAGAACAGTTAATGACTGAGGATCCTCTGAAAATACAAGGTCTCGTCTTGCTCATTAGCACCATGTACCTGTTAACGAACACAGC
TGAGTCAGTGGTGTTGGCCATTCCTTTTATTGGCCTGTTCTACAAACTCTTTCTGTGGTCCTTTATTGGACTCAACAAGGTGTATGGACTGTTGAACA
CGATGTACTGGCATTCCGAGGGTAAATCTTCGAGAGAGATATCTCGAATCATGCCTCGAGACCCGTACATGGTGACAAAACAGTTTGCTGCATTTTGC

ATCGACTTTGTACCATCACAACTGGGTTACTTGGCCCTTATCCCACTGCACATATTGTCCTTACTTCCAGTGCTTTTCGAGGCACTTGGAAAAGCGTG
GTATGTAGCAGGGGAGGTCAAGAATGTGATGGAACCTCCTGGCAGCACCGTAAACAAGTGGGACCAGTACGCAGACGAGTACATACAAGCTCTGC
GTTCGTCTCCCACAAGCAGAAAATACGTTGCTGCCAAGACTTCAACTGGCAAATCGACCTGGTTTATAGCTGCCCTGTGGAAAGCTAGACACCGCC
AGAAGGTCAGGAAATTTTGGCTTGTCGAACCTCGATTGGTGTTGCGCGACAACACACATATACCATTCGACGCACCCTATCAAGTACTCAAGGACAA
CATCCAAGTTTCAAACATCCACGATATCCACATATTGACGTATGGACATTTACAGTCAAGGTTGTTGGATATAGATAATGAGCGTGATATCGTCATTTTT
GACGAATTTCACGAACAACAAGGTGAAATGATTCTTGGGCTTGACAAGTGCAAAGCGCCAATCTTTCTTCTC

> Cb_clean_contig_2265
CCTTGCCTGAAACCTAACTTATTCCACCAGTTAACAAACGGTTTCAGGGTCTGCGCCAAGTTCTCCGGGCTCAGCGTTTTTGAGCGCGGGCTCGACT
TTACACGCCCTGATCCTCGGGTCGAAGGCGGCTTCATCACAACCAGACAGGAGAGGTTGTTTTGGGACGTAGGGGCAAGCAGGTTGGTGTTCACAC
CAAAAGGCCTGGCAAAGATATTCTTCCGCAAGCACAACCAAGAAAAATGGAGGATAGCTTTGGCTGCTGCGATTGGTTTGACCTATGACGAAGGTC
TGCTTTTCTTTTCAAAGTTTCACAATGATAACACGAAACCAGATGTTGTGTTGGAGACTTTGGGTTCGTCACCCTATCACAAGCCAGATAGGGAAAC
AGTGACAAGAGTGGCTTTTGTTGACGTGGAGAAACCTGTCGCACCCAACACTACGCCTCAGTCTTCCACAGTTGTTCCGCCTCCGCCGCCTCCACC
GCCGGTGCAGGAAGTCAAGCAAAAGAGGAAGAAGACAGAGTTAGCTGACGGTGACACGTGGCTTCTTGCTGAAGGTTCTGAAGGCAAGTCAAAC
AGAGCTCGTTTTAGATCTCTCTTCCCACATGATTGGTGGTTGAGAGTGTCTGAGAGGGTTATGCCTTTCATGAATCAGGATGCATCTCGTGACGTCGG
GTACAGATCCAGATCCTGGTCTCAATCTTCCCTGGTTTCAAATCCTTCAGAGAAGTTTGATATAAACACGGTGAAGTACACACCTTCTAGTTCTTCCA
AGGCATCAGGTTCAACACCTTTGCGGGTTGATCCTGAAGCTTTCAAGAGACAGCTTCAGGCTAAAGCTAAGAAAGTGAGTGCAACGTCGTCCTCCC
GTCCCGATGCGAGGGAAGTGTATCTGAAGGGTGTCGGTGAGTTCAAACCAGACAAGGTTTCTCACAGAAAAGCCCTTTCTGAACCTACTCCGTCGA
GGAAACCTTCCTCTGAGTGGATGTCTATATCTCGAGCATCATCTGATTATCTGTTCGATGATGATCATGAAGACGCAAGAGATTCGACTAAATCAGAC
ATTGAGACCAACCCTGGACCTTCAATCAGGCATTTCCGTTCCAAATCGACTTCACATTTGGAGGGTGAAACACCGTCTCTAAATTTTACACCACCTAC
CCCTGGTGATGTCTTGACTGTGCACCACCCGACACCACCTCGTTTGAGGGTCAATCACAGGATGAATAGACACACTGTGATTGTACCTTCACCGGGT
GAAAACACGTCGAACTGGAATGTAGGTTACTCGTCATTTGCATGGCCTCCAACAGAGGAACATCTGGAACCTCGAGACTCCAGAACCGCGGATATA
GAAGAAAATCCCGGACCATGTGAAACCCTTGACCCCACTTTGCCTCAGTTTCGAGGTCGGAGTGGTTCTCAGAGTTCAGTTGCCGCTGTCACTTCG
GAGCGATCACCTTCAAGAACACGTAAGTTTCTTGGAAAGATCGGCATCCATGGCAAAAGCGCAACGGAAGAAGTTAAGGACATTTTTGGATTTAAA
GAACATGATGAGGTGTATCCGTACACTGGTAAAAACTCATCTTTTGTTGAAAAGCATTATGAATTCCACTATGTTCTTTCTCATTTACCTAAAGACAAG
AAGTGGCGTTCATCAACCTTTATCAGGTGGGTTGCCGCTGCTTCTTTGTCCGAGCTTCAAAAATCACTTGACTGTGTCGTTAAGGCTTCCCACCCTTT

CAACACCGCGCTAGTGAGTAAGTTGCGTGATGAGACAAACTCCTTCTTCCAGAAGATCTCTGATTCTGAGCCAGTAGGCGGTACAACCATGTCTGCC
TTAGCTCACACGTTCAAGACCCTATACGCCTGTCCAAACAATGGATGCGGTTATGCACTGGAGGTGAAACCGGAAGATGGAATTGTACTGTTCCATC
TGACAGAGTCCCCAGACGAAATCGCCAAGAATAGGTTGTGGTGGGTCTTGAATGGGAATGAGATTTTGCACGGAGGGGACAAGGCTCCTGCCTCTC
CAAGCACAATCTCATTCGGTTGGCTGGCAAAGGAGTATTCAACACATGAGGTGATAATTGAGCCCAAACCGGACTCTGTGTTATCACAAGAGAAGC
CAGGGCCGTTCGCGATGCCCTCACTTTCTCAGTTTCAGAAATGTGTTGACAGGATTATTCCTACTGGTGGTCATTTCTCCCGTTCTGAGGTCGACGAT
CTCAAAAATCACATCAACCAATACCTGGAGTCTACAGATGTCAAGATAGTAGATCCAGTGTTGGTTGCAGCGATGGCAATGAGCTTCAAGTCAAAGT
TCAATTGTCCAAAAGATGTCAGCTCTTTCGCAATTGATTTTAGACTTGACCGGAGGATGGTTGTTTTCTGCGCCACAGATTCCCCTGAAGACAAAAA
TAAGGGGAAGCTTTATTGGTGCATTGGTGGTAGTGTCATTGTCCATGGCGGGCTGCTAGGTATCGAAGATGGGAAGTCTTGTTCCCCTTCACTGGATC
ACTATAGCAAGTTGTGGACGATGCCGCTTCCAAAATCGGGTTGGGTGGACAGGTCATTCGACGCTGAATACTTCACAAAGATCTCAACCTGGCAAA
AGGCCCTCGACCGGTGTGAACCGATCGTTGTATTCTCTGAACACACATTGCAACGCACACTTTACCAATACTTTGATGGTGTCACTGACAAGGTGTT
ATCACCATCACATACTGCAATCGTTGCTTGTGTGGTAAAATCCCTGATGAAATGCCCTGAGAGCGGGGTAGGTTATGCTGTACAGTTCGATAGTCATG
GGGCTTCGTTGTATGTGTGTCGACAACCGAACTCCGCAGAGGTGGACCGGTTGTGGTACTTTCACTGGGATTTTCCTTTCGTGCATGGGGGAAGAA
AATCTTATGCTCCGGAACCCAGGCAGTTCTTCTACACTGAGCATCTAAACATAGATTATTCGATACCCATGCGCGAAGTTTTTGCTGGGGAAGGTCTG
CCCAAATCCAATTCCTCACATTTGACTTTCAGGAGTCAACAGGAATTGTTTTTCGGTTTCCAGCAGCAAATAGATTTGTATGATGTGCCGACACCGGC
ATTCATGCATTCGAGAGCACATGGTGTGGCTGAACAAGCCATTCGTGACACAATACCCAAAATGAGAACTCTCCAGTTCAATTTTGAGCAAGTTGCC
GCGATAGCCATGTGTTATAGATCAAGGATGCAATGCCCATGTCCGGAGTCAGCCATGGCTGTTGACTTTGACTTCGCCAATGAGCGCATTGTGTTCTT
TTCGAACTTAGGCAGTGGAAGTTTCCGCTGCCGAGGTCAAATGTGGTGGTGTATAAATGGAGTCGGACTAGTGCACACTGGTTCTACATTCCACACC
TATCACAACCAGTCCAACCAATTTCGACCCGGTGCCCGAGTATGTAGGTACTGGGACGAGATTGTTTCTTTTGACTTGACTGAGTTCTGCCAACCTAC
GTTGGATTATTGGACATCGGATTTCCATTTGCAGCTTGACAAAGACGAGCAGCTACAGAGGGCTCAGATTGCATTGGATGCGCATAGTGACCCTGTT
GAGCCTTTCAGCAGGGACATTGCCATATTTCATGCATTTGAATCAATCAGACAAACAGCCCAAGCGGTCGATATAACTGTCATGAAGATTGAACATTT
GGTCATCATGGCATGCATATATCGTTCGAAAATGGCATGCCCTACTGTAGGCACCTCAATGATTGTCAAGTTCGATTTGTCAAATCAGAGAGTGAGGT
TTGGCGTTGCAAGAGGAACGTATCCTGAGTCAGATGGCAGGATGTGGTGGTATCTGCACGATGATTGCATGATCCACACGGGTAGGCCGGAAAAGC
TTCACACCGCTGGGGTGGAAGCGCCGTTGCCTGTCAGCAGTCGATTTTCCTTTCGTGCAACTCACCGTCAGGCCTGTGAAATGGCCGACGGTTTTG
AGAAAACTTGGAGAAAAGACCAAAGATCCGTTTGCAAAGACAAGGGTGAAAACCACAATCATCATCCCCTTTTCTGCGGATCAACTTTTGACGAGA
GGCACGATGAGGTATTCGTGTCCGCTTACATATGGTTCTACATCCAACTCATTTGCACGTTTTATTTCTCCTTTTCCCGGCCTAATCTGCGGCGAAGAC
CTGACGGTCACACAGAAGGGGGAGAGTATCCCGTTGCGGATGAGAGTTTTTCAGCCTCATCTTCCCTCCTAGTGTGCAAGTTAGGGACTAGAGGTG

ACCAAGTTCCTGTTGATTATTACGCGAACGTGGCGGCACACTATGGTGTCCGTACACATTCCCATGTGTATAAAGACGTAACAACAGACGATCTTGAA
AAGATGAAAGTCGGAGACTTTTCATCTAGTTCCGCTGGTTACCTGAATCTTGCGTATGCTTCTCAACTTGGTTACAAGAGACTGTTCACTCCGCATGT
GGAGGTGGAAAGGTCTCAAGGTGTAAGTTATACTTTATCACCAAGCTCAAAGTGGGTGGAAGCAACAAGGTTCATTGCCGATTGGTCGAAGGTTTC
CTGGTCCAACTTTCTCCCTGCGTGGATAGCCACGAGGGTCACTGCTGTGTATGTGCCAACTTTCAGAATTGGTGCGCTGTCCGGAAGCGACATCCCG
CGTTCTCACGATGGGTTAAACTTATTGGAGAAAGTAAAAAACACAGCAGAGTATGAGGAAGGTTGGACATCCGGCAGCTCGTCAGTTTCAGTGATC
CCTGACGAGATCAAGTCGAGATGCCCCGAAATACCATCGGGTAATCATCTTGAAATATTGAAGGATTACAAAACGGTGCACATCCACGGGGGGGCCG
GCACTGTGCAGACCGCCATTGCCTGCGGTGCACGACCGGTTATCCACGACAAGACACTCGATAGAGTGTATCATAGGATGCCTGAACCAAAGGATTT
CAAACAACCCTCCATTGGCAAGTTTATGGGTTGGTTGGTGTCTGCTGGTTTCAAAGTTGACGCGCCAATTGAGGTGAGAGCATGGTGGTTGTTACAA
TGGTATTGGTCCCAAAAATGGAACTATGCGTACGACGCATTAGTTACACTTGCGAAAGCCTATACTATATGTTCATATGTATATAACCATTGGGAACTTT
TCCTCATTCTTTTCTTTACCGTTCCAGCAGTGATCTGGAGGATGCTTTTCTCTTCCAAGTCATTGTTCGCCATAGTCAAAATCTGTGCGCTTGGTTTTT
GGCGCTACCCTGTGTTATGCTTGCTAGATTCGAAAGTCACGTTTGTGGCGGGCATCTGGTCAATGCAAAACTTGTGGATGGGCGCAGCGCAAGATTT
CGCGGCACTTATGGAGACACGTTCCTCACTGGTTTGGGAGCCAGTGCACAAGATCGTGGGAACGAAGAAGCTGACATTCCCTTTTCCCCTTGGGCA
TTGGAGCATGAAAGACAATCAAACCGGCAAGATCTATGAAGGTAGATTTGTCGGCCCCTCTGAAACGATCGGAGGAAATTTTCGTCTTGTAAGTACC
ACTCGTGAGTACAAGCACGGATTTAAGTCTTTTCCGGCTCCTTTCAATGTTGGCCACGCGGAGTCACTCGTAAGAGCACAACACGTGGCACCATACT
CATCTTCACATAATTGTGTGACCATGATGGTGCAACTTTGTGCTAAGAGATCGACTGCATGGACAGCATGCATGATAATGGTTGGTGCTCTGACAGGG
TTGGCACTGGCACCACCAAAATTGCTCAAGGATGTTCTTTCTTTTCTTTTTCCTGGCAAGGATTTTGCCAACACCCCTTTTTATCGCTCGCTTGGCTTC
GCCGCGTCCCTAGAAAACATCCCGCTCGAAATAGAGGGGATGTTAGAAGCGGCAGAACCAATGCCACAGATAGCTACACCAGAACCAACTAATCTG
GCCCACCCAGATTCTTTCAATATGTTGGTTGATGAAATAGCGTTCATCCAGGCATGCGTGGAACAACTCAAGATACCCACCTTAGATAAGGAGGCATT
TGACGAAGTTTCACAGCGGACTTTAGAAAAGGAGCTGGACAAGGTCGAAATCCCTGAAGACTCACTGATCAGGGTATCAAACATACCGCCAAGAG
TGCACCATGAATGGGCACAACTGGTAGATGGTCTACACCATGCCATTTCTTTCGTTCGCACAAACCGCGTCGCAGACGCGTTTGTGTTATACGTCAA
AAATGCCGGTGAAAACATATATGACTTTGTCCAGCCAATACTGGAAATCCTTTCCTTCTTTCTGCACCTTGCAGCTGAGCACTCCAAGCATGCGTTCG
ATCGTCTGTTCAAATGGCTTTGCGATTTCTTGGACCACGTGTGGGGTCTAGAAGCGTCGAAGAGAGTCAAAACAGTGTGGGGCCTGACCGGCCTTC
ATCGAACGGGACTGCTTGGTGTAAAAGCCAAACTTGCAGCTAATGTTGCTTATTCTGATTTCGTAGGTAGACAAGATTTCGAATCAGATTACAACAA
GCTGGTGCAAGAAGGCAAGCACCTTGCTCAGGTGTATGGTGCCGTTAAACGATCAAACCTTGGAGGGCCTCAGAGGAGGCCTATAGGTTACGGAAA
ACCTCTAATGTCACAATCTGAAGCCAGTCTGCTCGGTTTCGGACCAGACGAGTTTGTGACTGATCGTGAGTATGAGGATCGTGTGCAGTCATACCTT
GAACAGGGTGTTGCACAAGGTGCTGACGGGGTATTTTTGGCAGACAAGAGACCGGAGCTGATTGCTAAATCACAGCACCGTTATGAACCAAAGTAT

CCCGCACTCACTTCAGAGGATCGTTCCATGGCCAAGGAGATAGCCCAAGCCATGTTTGATCAATATCCGGAAGTCTTCGCTAATTGTGACGTTTTGCC
ACCAAGGGCAGTCCACAATTATGTGAAGCAAAAATACTCTCCAGGAACGCCGTTCATCAAACCTGGGGGCTTCAAATCTCGTCAAGCAATGTTTGA
CGCCGGTTATGACCGCGTCATGCAACGACGAGCGATGCATTACTTGAAAACTGGTGAATATCCAGTGCAATTCTATCACGCTTTTGTAAAATCACAAG
TGGTGGATATCAAGAAATGTTTGAGCCCCGAGGAAGGTGGAAAAGGAAAAGATGTTCGCACGGTGGTCAGCCAAGATCTGTTCTCCTATTACATTG
ACCAATGTCTCCAAATTGAGCGCAACAAACGCATAAACTGGGATACATATGGTGCAGGTATAGGTATGCCTCTTAATCAGTCGATGGAGGCAATATAC
GGTAGAATGGCAGCTGCACAGAAATCGAGAGGAGGCAGGTACATCATTGCTGATGCAACCGCCTTCGACTCATTTTGCAAACCTTTCCTTTTTGAAG
TAAATGCAAACCTTTGGGAACTCGGCTACAAAGATCATCCAAGCGGTAACGGCAAGAACCTCGCATCTGTTCTAAATGCGTCGTATCAAGCCAGACA
AAATGCGTGGATAATCGGTGTGACCGAGCCAGAATATGACTCGTTGACTGTTTGCGTCCAAGACAAAAACAGCCGCGAGCTCATACGGCAACGATC
CCCAAAGACAGTAATCTGCTTAGAAGATCTCATAGACTGGGACAAGTACAACAGATTACCTGCTGAGCAACGTCCAAACTACGTTGCAAAACTTCA
ACCACCTGAAGGAAAGACGGTGGTAACCTGGGATCCTTCTCTCCGCCCATCCAGATCAAACTGGATGGGCAAATATGAATACGGTGTCTTATCTGAT
GTCGACAAGAAGTTCTTCGAACATCAGACATTCACATACGATCCTTCAAATTTTGAAGACATGTACCAAGACATATTATCAGTTTCACGATCAAATTA
CAAACTGATGTCAAACGTACATACGAAGAATCGTGGTGGAAGTACTGGAGGGTCAGACACATCAAACGTTAATACACATGCCTTCAAGGCAGGTGT
AATTTACGCTTGGTGCCAGACCACTGGCAGAGCTCCAAAGGAGTTCTTCCAGTACAATGACCTTGCCAATACATCAGATGACACCATCTGGCAATCG
GGTGGCAAACACGGCCTTAACACTGTTGAAGACGTCGAAGTCTTTCGGCATCACTGTCAATCAGTGGGCATCCATCTGGAGATAGAAACAACGAAG
AACATATCACAAGTAGAATACTTGTCAAAATTCGTTCGCGTCCCGACTGCAGAAGATTCAGCAGCATTGAAAGTCTGGAGATCCCAGAAAATCAAG
GCTGCTTCAAATGCCCACAAACAGTTAGGCCTAACTGTACCCACTTCGTGGGAACAGTTCAACAACCCACAATTTGTCGTCGTGCAAAACCCGTCT
GCGATCTTGCTCCGAAGATCTGCGTTTAGATATTACCAATCAAACGCAAACCATTGGAAGTACGTTTCAGTAGAAAGAGGTGCCGGTCATGCTCAAA
GCACCGCCTTCCTACCTGAACTATATGGCAAGTTCGCACAAGAGTGGTGCGACGACATCAACGATCTTCTGCTCCAACAGAAGATCCACCGAAAGT
TCAAGGTCACAAACGGTCAATTTGGACTACAATGCGTCGAACAAGTGGATCCAAGAGCGTCACAACAACAGCTCTCACCACGTCAAGTTGCTTTCC
TCTCATGGTTGAGAGGAAACATGTTCCCTAGCTACTACAGAGTACTTGACATCCACATGAACGTCGCAAAAGTAGATCCAGAACGACACGCCAAATT
CCTTCGTAAACTTGAGAAAGGTTGGCGAGGTTGGGAAGAACTGTTACGTGAAGGAGTAGACAACCTCTACGCCTTAACTGATTCCATTCCAGATGA
AATCAGCAAAAAGTTCCAACCCGGCCTCGATATGCTCTATGCGGAGCAACCGTTCTACACGAAGAACAAAGTGGTCGAAAAGTTTGTTTACCTAAA
ACTTCTGGAGGAAAGCCCAGAAACAGAGGTCACATTTTCAGATTTCTCTGCTCGAATTCAAGAGTCACCATACGCATCGGTGTGTGACCCCTATCAC
TTCTGGGAGAAGCTAGGGGAACCTGAATTCAAAGAGCAGCTAATGACTGAAGACCCTCTGAAAATACAAGGTCTCGTCTTGCTTATAAGCACCATG
TACCTATTGACAAACACAGCTGAGTCAGTGGTGTTGGCCATACCTTTTGTTGGCCTATTCTACAAACTCTTCCTGTGGTCCTTTATCGGACTCAATAAG
GTATATGGACTGTTGAACACCATGTACTGGCATTCCGAAGGTAAATCGTCGAGAGAAATATCTCGGATAATGCCTCGGGACCCGTACATGGTGACAA

AACAGTTTGCTGCGTTCTGCATCGACTTTGTACCATCACAACTGGGTTACTTGGCCCTTATCCCGCTACACATATTATCCTTACTTCCAGTGCTGTTCG
AAGCACTTGGAAAAGCATGGTATGTGGCAGGAGAGGTCAAGAATGTGATGGAACCCCCTGGCAGCACCGTAAACAAGTGGGACCAGTACGCAGAT
GAGTACATACAAGCTCTGCGTTCGTCTCCCACGAACAGAAAATACGTTGCTGCCAAGACATCAACTGGCAAATCGACCTGGTTTATAGCTGCCCTTT
GGAAAGCTAGACA

> Cb_clean_contig_2381
TTGTTGACATGTTTATGGAAGCCAGAAACCGTTACCCTGATTTATGCAAGAGAGCAATGATCATTGTACCCACTCATAAGGAGGTTGCAAAAGTCATA
GCTTCTCTTGACTACCTCAAGGTAGGCAAAGTGTCCCCTCTGTCCGCGCGTGACCGCGTGGTACAAGAAGAGGGAATTCTGGTGTGCACACCTTAT
GTCCAAACAGGTCTAGATATAAAACCACCACCTGATCTTTTGATCGATTGTGGCAAAGACTTTGTTTTGGATAAAGGACGTCCAGTTTTCCCACTTCC
ATGGACAGATAAGGACATAAACCAGCAGAGAGTTGGTCGTGTCGGAAGATTAAGACCCGGAATCGTCTTCCAACCGAAATCAGCAGGTTCCGGAA
AGAAAGGTGTGGTTTACCCGAGTCCACACCTGTTCCAACATAAAGCTGTCGCGGAACATTTCAAGGTTCCACAGCTAGCACCAATCTCGGGTGCTTT
CACGGCAGACTTGCCTTTCTTACGACCGAACAAGAAAGTACTAAATTCTTTACCCGTCGAAAAGTCAGTTGTTTTCATACACTCACTTGCTTTAAGT
GGTGTTCGCCAAGAACAATGGCAACACATGTATACGAGATCCGTGCAAGGGCACACTCTCGGCAACGACTACAAAAACGTAGACAGACTGCTCAAT
CATTCAAGGTACAAGCGATCAAAGCTAGTCTCCTATGGAGACGCGCTTTATCATCTCTACCGAGAACACGCAGTCCAGTACGGATTTGTCAAAGAAG
ACGGAAGCACAGAGTACAAATCTGTCAAGCCAATGTCGGTCATCAACGGAAAGTGGATAGAGTTAGAACAATCGCCGACGACTAAGATTGATGTTC
AAGAGTTGCCGCAACAGGAACTTTCAAACAAATATCAATCTTTCGAATCATCAATAAGGCGATGTCGAGACTCCATTCTTTTCAAAGCCAAGGACAT
GCCTGGGCATGACTTGGAACGTCTCAACTCCATCTTAGCGTTTTGAGCACTAAAAGGCGGCACTTGTAGCTGAGATGCTACATTTAAAGACAACCGC
AGTTTTGTGTTACCCAATTGACGTTTGACTTTTGTCAGACACGCTATTTCTGTTGTTGCCTCCCGACCTCTTTCGTGACATT

> Cb_clean_k141_1313
CAAGAAGACCGACGGTGTCCTCGGCGACGTCGCCCCCACTGTCCTCGCAGTCATGGGTCTCGACCAGCCCAAGGAGATGACCGGCACGCAGGCGC
ATTTGAGTGCTTTGGCGTATATGAGTGATTCGTACTTCATCGGAACCGTGCAGAGATGGTATTTCGGCTGGCGTGGCCGAGAGAAGAAAGATCGGCG
CCTTGCACTTGTCAAGCCCAAGAATCATTTCACCTTGTTGTTCGTGGAACTCGTCAAAAATGACGATATCACGTTCGTTGTCTATATCCAACAACCTT
GATTGTAAATGTCCATACGTCAAGATGTGGATATCGTGGGTGTTTGAGACTTGGATGTTGTCCTTGAGCACTTGATAAGGTGCGTCGAATGGTATGTG
CGTATTGTCGCGCAATACCAATCGAGG

> Cb_clean_k141_49228
GACAAGATTTCGAATCAGACTACAACAAGTTGGTGCAAGAGGGCAAACACCTTGCTCAGGTGTATGGTGCCGTCAAACGATCAAACCTTGGAGGG
CCTCAAAGGAGGCCTATAGGTTATGGAAAACCTCTGATGTCACAGTCTGAAGCCAGTCTGCTTGGGTTCGGGCCAGACGAGTTTGTGACTGATCGT
GAGTATGAAACCCGTGTGCAATCATACCTCGAACAAGGTGTTGCGCAAGGTGCTGATGGAGTGTTTCTAGCCGATAAGAGACCGGAGTTGATTGCC
AAGTCACAACACCGTTACGAGCCGAAATACCCCGCACTCACTTCAGAGGACCGTTCCATGGCCAAAGAGATAGCCCAAGCCATGTTTGATCAATATC
CAGAAGTCTTCGCTAATTGTGACGTTCTGCCACCAAGGGCAGTCCACAACTATGTGAAGCAAAAATATTCTCCAGGAACCCCGTTCATCAAACCTGG
GGGCTTCAAATCTCGTCAAGCAATGTTCGACGCTGGTTATGACCGCGTCATGCAACGACGGGCAATGCATTACTTGAAAACTGGTGAATATCCA

> Cb_clean_k141_63002
AAATCGACCTGGTTTATAGCTGCCCTGTGGAAAGCTAGACACCGCCAAAAGGTCAGGAAATTTTGGCTTGTCGAACCTCGATTGGTGTTGCGCGAC
AATACGCACATACCATTCGACGCACCTTATCAAGTGCTTAAGGATAACATCCAAGTTTCAAACACCCACGATATACACATCTTGACGTACGGACATTT
ACAGTCAAGATTGTTGGATATAGACAATGAACGTGATATCGTCGTCTTTGACGAGTTTCATGAACAACAGGGTGAAATGATCCTTGGGCTTGACAAG
TGCAAGGCGCCAATCTTTCTTCTCTCGGCCACGCCAGCCGAAATACCATCTCTGCACGGTTCTGGAACTCTGGTTCCAAACATAGACAGACGACATC
CAATCCAGATCCACCAAGTGGATGACGGAATGGGTGTTGTTGACATGTTTATGGAAGCCAGAAACCGTTACCCTGGTTTATGCAAGAGAGCGATGAT
TATTGTACCGACTCATAAGGAGGTTGCAAAAGTTATCGCTTCCCTTGATTATCTCAAGGTAGGTAAGGTGTCCCCTCTATCCGCACGCGATCGCGTGG
TACAGGAAGAAGGAATACTGGTGTGCACCCCTTACGTCCAAACTGGTCTAGATATAAAACCACCACCCGATCTCTTGATCGATTGTGGTAAAGACTT
CGTTTTGGACAAAGGGCGTCCAGTATTTCCACTCCCGTGGACAGACAAGGACATAAACCAGCAGAGAATTGGTCGTGTTGGAAGATTAAGACCCGG
AATCGTCTTCCAACCGAAATCAGCAGGTTCCGGAAAGAAAGGTGTGGTTTACCCGAGCCCACATTTATTCCAACATAAAGCTGTCGCCGCGTATTTG
TGGCTGGACGACGAGCTCACGAACGAGCCTCTGTTTTGGAC

> Cb_clean_contig_1397
ATGATCTTGATGATGTCGCTTCCGGTGAAAGGCATGGTGACGGTGGTTTGGTGTGGTAGTGGTGAAGGTACTGAGGACGAAGTGGGAAGGCTTCTA
GAGCTTGTCGTCGCAGGGTGTTTGCTGTGGTGTTTGTGGTGAATGGGGGAGAAAGAGAAACACAAGTCAGGCGCTGCTGGCGACAATATACTTGCT
CGTCACTTCTGACGTAACACCAGCGCACTTATTCACACTTCTTGGTGCACAAGAGCACAGGGCACTTGGCCAGCACCGGCACGTCTCACACACTGA
CCCACCCACAAGGCACACGCTCGGCAACGACTACAAAAACGTAGATAGACTGTTTAATCATTCAAGGTACAAGCGATCAAAGCTAGTCTCCTATGG
AGACGCGCTTTATCATCTCTACCGAGAACACGCAGTCCAGTACGGATTTGTCAAAGAAGACGGAAGCACAGAGTACAAATCTGTCAAGCCAATGTC

GGTCATCAACGGAAAGTGGATAGAGTTAGAACAATCGCCGACGACTAAGATTGATATTCAAGAGTTGCCGCAACAGGAACTTTCAAACAAATATCA
ATCTTTCGAATCATCAATAAGGCGATGTCGAGACTCCATTCTTTTCAAAGCCAAGGACATGCCTAGGCATGACTTGGAACGTCTCAACTCCATGCCA
GGCGCGCTAGGACTTGCACCCAAAGATCTCGATTCTCGCAGCATCCTCTTCGCAAGAAACGCGCTTCTTCGCTTTGCTGAGCAGTTCCCTGGCGCTT
CGATATGAGGACAAGGCTCCTCTGAAGTTCCCGTCTT

> Cb_clean_contig_1993
AGGAGGAGGAGATCCTATATTATACCAACATCTTTTCTGATTCTTCGGTCACCCAGAAGTTTATATTTTAATTATACCAGGTTTTGGTATAATCAGTACA
ACTATATCTGCAAGCTCAAATAAAAGCGTATTCGGACCTAGGTATGGTATATGCTATGATGTCTATAGGTGTTTTAGGGTTTGTAGTTTGAAGTCACCA
CATGTACAGTGTAGGTTTAGACGTAGATACACGTGCCTACTTCACTGCTGCTACATTAATAATAGCAGTACCAACTGGAATTAAAATATTTAGTTGATT
AGCTACTTGTTATGGAGCGTCAAGAATGTGATGGAACCCCCTGGCAGCACCGTAAACAAGTGGGATCAATACGCAGACGAGTACATACAAGCTCTG
CGTTCGTCTCCCACGAACAGAAAATACGTTGCTGCCAAGACTTCAACCGGCAAATCGACCTGGTTTATAGCTGCCTTGTGGAAAGCTAGACACCGC
CAAAAGGTCAGAAAGTTTTGGCTTGTTGAACCTCGATTGGTGTTGCGAGACAACACGCACATACCATTCGATGCACCTTATCAAGTGCTGAAGGAC
AACATACAAGTTTCAAACACCCATGATATCCACATATTGACGTATGGACATTTACAGTCAAGGTTGTTGGATATAGACAATGAACGTGATATCGTCATC
TTTGACGAGTTCCACGAACAACAGGGTGAAATGATCCTTGGACTCGATAAGTGCAAGGCGCCAATCTTTCTTCTCTCGGCCACGCCAGCCGAAATA
CCATCTCTGCACGGTTCCGGAACTTTGGTCCCAAACATTGACAGACGACATCCGATCCAAATCCACCAAGTGGACGATGGGATGGGTGTTGTTGAC
ATGTTCATGGAAGCCAGAAACCGTTACCCTGATTTATGCAAGAGAGCGATGATCATTGTACCAACTCACAAGGAGGTTGCAAAAGTTATCGCTTCCC
TTGATTATCTCAAGGTAGGTAAGGTGTCCCCTCTGTCCGCACGTGATCGTGTGGTACAGGAGGAGGGAATACTGGTGTGCACCCCTTACGTCCAAAC
GGGTCTGGATATAAAACCACCACCAGATCTTTTGATCGATTGTGGCAAAGACTTCGTTTTGGATAAAGGGCGTCCAGTATTTCCACTTCCGTGGACA
GACAAGGACATA

> Cb_clean_contig_3558
GTCATTTCTCTCGTTCTAAAGTCAGTGAACTCAAAGATCACATCAACCAGTACCTGGAGTCTACAGATGTCAAGATAGTAGATCCGGTGCTGGTCGC
GGCGATCGCAATGAGTTTTAAGTCGAAATTCAATTGTCCAAAAGAAACCAGCTCTTTCGCAATTGATTTTAGGCTCGACCGAAGAATGGTTGTTTTC
TGTGCCACAGATTCCCCTGAAGATAAGAACAAGGGGAAACTTTACTGGTGCATCGGTGGTAGTGTTATCGTCCATGGCGGGTTGCTAGGTATTGAAG
ATGGGAAGTCTTGTTCCCCATCATTAGATCACTATAGCAAGTTATGGACGATGCCACTTCCGAAATTAGGTTGGGTAGACAGATCTTTTGACGCTGAA
TACTTCACGAAGATCTCAACCTGGCAAAAGGCCCTCGATCGGTGTGAACCGATCGTTGTATTCTCTGAACACACATTGCAACGTATACTCTACCAGTA

TTTTGATGGTGTGACAGACAAAGTTTTGTCCCCATCACATACTGCAATCGTTGCTTGTGTGGTGAAATCCTTGATGAAATGCCCGGAGAACGGGGTA
GGGTATGCAGTACAGTTTGACAGTCATGGGGCTTCACTGTACGTGTGTCGGCAACCGAATTTCGCAGAGGTGAACCGGTTGTGGTACTTTCATTGGG
ATTTCCCTTTCGTGCACGGGGGAAGAAAATCTTATGCTCCGGAGCCTAGGCGGTTCTTTTATACTGAGCATCTTAACATAGATTATTCCATACCCATGC
ATGAGGTTTTTGTTGGAAAGGATCTACCTAAGTCCAATTCTTCTCATTTGACTGTCAGGAGTCAACAAGAGTTGTATTTCGGTTTTCAGCAACAAATT
GATCTGTATGATGCGCCAACGCCGGCCTTCATGCATTCAAGAGCACATGGCACAGCTGAGCAAGCCATACGTGACACAGTGCCCAAGATGAGAACT
TCTCAGTTCAATTTTGAGCAAGTTGCTGCGATAGCCATGTGTTATAGATCCAGAATGCAATGTCCATCTCCGGAGTCTGCTATGGCTATCGATTTTGAC
TTCGCCAATGAGCGCATTGTGTTCTTTTCGAACCTAGGCAGTGGGTGTTTCCGCTGTCGGGGCCAAATGTGGTGGTGTATAAACGGGGTTGGTCTGA
TTCATACTGGATCAGTGTTCCACACCTACCACAACCAATCAGGCCAATCTCGACCTAGTCCCCGAGTGTGTAGGTACTGGGACGAGATTGTTTCTTTT
CATTTGACCGAATTCTGCCAACCCACGTTGGGTTATTGGACATCGGATTTTCACCTGCAACCTGACAAAGATGAACAGTTGCAGAGGGCTCAGATTG
CGCTGGATGCACATAATGATCCTGTTGAGCCTTTCAGCAGGGACATTGCCGTATTTCATGCATTTGAGGCGATCAGACAAACGGCCCATGCGGTCGAT
ATAACTGTCATGAGGATTGAACATTTGATCATCATGGCGTGCATATATCGTTCGAAAATGGCGTGCCCTACAGCCGGCACTTCAATGATTGTCCAGTTC
GATTTGTCAAACCAAAGAGTGAGGTTCGGCGTTGCAAGGGGAACCCATCCGGAGTTGGATGGCAGAATGTGGTGGTATCTGCACGACGATTGCATG
ATCCACACTGGTAGGCCGGAAAAGCCTCACTCCGGTGAGGTTGGAACTCCGTTGCCTGTCAGCAGTCGGTTTTCTTTTCGTGCAACTCATCGTCAGG
CCAAGGTAATGGCTAACGATTTTGAAGAGAAGTGGAGAGAAGACTCTAAGTGTAGGGCCCGGACATGGCACATTGAAGGTGATGATACACCGAAGC
AAAAGCTTGCTCGGTCCCATCACCCTTTGTGGTGTCCGGATACGTTCGACGAGCGGTTCGACGAGATCTTCGTTGCCGCTTACATTTGGTTTTTCTTT
CAACTCATTTGCACGTTTTACTTTTCGTTTTCTCGGCCAAATCTTCGGCGAAAACCTGACAAACACACGGAAGGAGGAGAGCATCCTCTTGGAGATG
AGGGTTTTTCTGCATCATCCTCCTTACTCGTGTGCAAGTTAGGAACCAGAGGTGACCAAGTCCCTGTCGATTATTACGCGAACGTGGCTGCACACTAT
GGTGTCCGCACACATTCGCACGTGTACAAAGATGTGACGACAGATGATCTTGAAAAGATGAAAGTTGGAGACTTTTCATCTAGTTCCGCTGGTTATT
TGAATCTTGCGTACGCTTCTCAACTTGGTTATAAGAGAGTTTTCACCCCGCATGTGGAGGTGGAGCGGTCTCAAGGTGTGAGTTATACTTTGTCACCA
AGTTCGAAGTGGGTGGAAGCAACGAGGTTCATTGCTGATTGGTCGAGGGTTTCTTGGTCCAATTTTCTCCCCGCGTGGATAGCCACGAGGGTTACTG
CTGTGTATGTGCCAACTTTCAGAATTGGTGCGTTGTCTGGAAGTGACATCCCGCGTTCTCATGACGGGTTGAACTTGCTGGAGAAAGTTAAGAACAC
AGCAAAGTTCGATGAGGGTTGGACATCTGGCAGCTCGTCAGTTTCAGTAATCCCTGACGAGATCAAGTTAAGATGCCCCGAAATACCATCGGGTAAC
CATCTTGAAATATTAAAGGATTACAAAACGGTGCACATTCATGGTGGAGCCGGTACTGTGCAAACTGCTATTGCTTGCGGCGCACGACCGGTCATCC
ACGACAAGACACTCGATCGAGTGTATCATAGGATGCCTGGGCCAGAGGATTTCAAACAACCTTCTATTGGGAAGTTCATGGGTTGGTTGGTATCTGC
CGGTTTTAACGTTAATGCACCAATAGAGGTAAGAGCGTGGTGGTTGTTACAATGGTATTGGTCGCAAAAGTGGAACTATGCGTACGATGCATTAGTTA
CACTTGCTAAAGCTTACACCATATCATCGTATGTGTACAATCATTGGGAGCTTTTCCTCATTCTTTTCTTCACCGTTCCGGCAGTGGTCTGGAGAATGT

TGTTTTCTTCTAAATCATTGTTCTCCATAGTCAAAATTTGTGCGCTTGGTTTTTGGCGCTACCCTGTGTTGTGTTTGTTGGATTCGAAAGTCACTTTCG
TGGCAGGAATCTGGTCAATGCAAAACTTGTGGATGGGCGCGGCGCAAGATTTGTCTGCACTTATGGAGACACGATCTTCACTGGTTTGGGAGCCAG
TGCAAAAGACCGTAGGTACGAAGAAGTTGACGTTCCCTTTTCCCCTTGGGCATTGGAGCATGAAGGACAACCAAACTGGTAAAATCTACGAAGGTA
GGTTTGTCGGTCCCTCCGAAACTATTGGAGGGAACTTTCGCCTTGTAAGCACTACTCGTGAGTACAAGCGTGGGTTTAAGTCTTTTCCTGCCCCTTTC
AATGTTGGCCATGCAGAGTCTCTTGTGAAGGCTCAGCACGTAGCACCATATTCATCTTCGCATAATTGTGTGACTATGATGGTGCAGCTTTGTGCTAA
GAGATCGACTGCATGGACAGCATGTATGGTGATGGTAGGTGCCTTAACGGGACTTGCCCTGGCGCCGCCAAAACTGCTCAAGGACATTCTTTCTTTT
CTTTTCCCTGGCAAGGATTTTGCCAGTACTCCTTTTTATCGCTCGCTTGGCTTTGCCGCGTCGTTGGAAAACATCCCGTTTGAGATAGAAGGGATGGT
CGAGACGACAGAGCCAATGCCAGAGACAGCTACACCTGAACCAACAGATCTGGCGGATCCAGATTCTTTCAATATGTTGGTCGATGAGATAGCGTTC
ATCCAGGCATGCGTGGAACAGCTCAATATACCTACCTTGGACAAGGAGGCATTTGATGAGGTTTCACAGCGAACTTTGGAAAAGGAGTTGGACAAG
GTCGAAATCCCTGAGGGTTCGTTAATCAGGGTTTCCAACATACCACCGAGAGTGCATCATGAGTGGGCGCAGCTGGTGGATGGTCTCCACCATGCTA
TTTCTTTCGTTCGCACAAATCGCGTCGCAGACGCGTTTGTGTTATACGTTAAGAATGCTGGTGAAAATATATATGACTTTGTCCAGCCGATACTGGAA
ATCCTTTCCTTCTTTCTGCACCTTGCAGCTGAGCACTCCAAGCATGCATTCGATCGTTTGTTCAAATGGCTTTGTGATTTCCTGGACCATGTGTGGGGT
CTAGAAGCGTCAAGGAGAGTCAAAACAGTGTGGGGCCTGACTGGCCTTCATCGAACGGGATTGCTTGGTGTGAAAGCCAAACTTGCGGCTAATGTT
GCTTATTCTGATTTCGTAGGTAGACAGGATTTCGAATCAGATTACAACAAGCTGGTGCAAGAAGGCAAGCACCTTGCTCAGGTGTATGGTGCCGTTA
AACGATCAAACCTTGGAGGGCCTCAGAGGAGGCCTATAGGTTATGGAAAGCCTCTAATGTCACAATCTGAAGCTAGTCTGCTCGGTTTCGGACCAG
ACGAGTTTGTGACTGATCGTGAGTATGAGGGTCGTGTGCAATCGTACCTTGAACAGGGTGTTGCGCAGGGTGCTGACGGGGTATTTTTGGCAGACA
AGAGGCCGGAGCTGATTGCTAAATCTCAGCACCGTTATGAACCGAAGTATCCCGCACTCACTTCAGAGGATCGTTCCATGGCCAAGGAAATAGCCCA
AGCCATGTTTGATCAATATCCGGAAGTCTTCGCTAATTGTGACGTTTTGCCACCAAGGGCAGTCCACAATTATGTGAAGCAAAAATACTCTCCAGGA
ACCCCGTTCATCAAACCTGGGGGCTTCAAATCTCGTCAAGCAATGTTCGACGCTGGTTATGACCGCGTCATGCAACGACGAGCAATGCATTACTTGA
AAACTGGTGAATATCCAGTGCAATTCTATCACGCTTTTGTAAAATCGCAGGTGGTAGATATCAATAAATGCTTGAGTCCCGAGGAAGGTGGAAAAGG
GAAAGATGTTCGCACTGTGGTCAGCCAAGATCTGTTTTCCTATTACATTGACCAATGTCTCCAAATTGAGCGCAACAAGCGCATAAACTGGGATACG
TATGGTGCAGGTATAGGTATGCCTCTTAATCAGTCGATGGAGGCAATATACAGTAGAATGGCAGCTGCACAGAAATCGAGAGGAGGCAGGTACATCA
TTGCTGATGCAACCGCCTTCGACTCATTTTGCAAACCTTTTCTTTTTGAAGTGAATGCAAATCTTTGGGAACTCGGCTACAAAGATCATCCAAGCGGC
AACGGCAAGAACCTCGCATCTGTTCTAAATGCGTCGTATCAAGCCAGACAAAATGCGTGGATAATCGGTGTGACCGAGCCGGAATATGACTCGTTGA
CTGTTTGCGTCCAAGACAAAAACAGTCGCGAGCTCATACGGCAACGATCCCCAAAGACGGTAATCTGCTTGGAAGATCTCATAGACTGGGACAAGT
ACAATAGATTGCCTGCTGAGCAGCGTTCGAACTACGTTGCAAAACTTCAGCCACCTGAAGGGAAGACGGTGGTAACCTGGGATCCTTCTCTCCGCC

CATCCAGGTCAAACTGGATGGGTAAATATGAATATGGTGTCTTATCTGATGTTGACAAGAAGTTCTTCGAACATCAGACATTCACCTACGATCCTTCA
AATTTTGAAGACATGTACCAGGACATATTATCAGTTTCACGATCAAATTATAAACTGATGTCAAACGTGCATGCGAAGAATCGTGGTGGAAGTACTGG
AGGGTCAGATACATCAAACGTTAATACACATGCCTTTAAGGCAGGTGTAATTTACGCTTGGTGTCAGACTACTGGTAGAGCTCCAAAGGAGTTCTTC
CAGTATAATGACCTTGCCAATACATCAGATGACACCATCTGGCAATCGGGTGGCAAACATGGCCTTAACACTGTTGAGGACGTCGAAGTCTTTCGGC
ATCACTGTCAGTCAGTGGGCATCCATCTGGAGATAGAAACAACGAAGAACATATCACAAGTGGAATACTTGTCAAAATTCGTTCGTGTTCCCACTGC
AGAGGATTCAGCAGCATTGAAAGTCTGGAGATCCCAGAAAATCAAGGCTGCTTCAAATGCCCATAAACAGTTAGGCCTGACTGTTCCCACTTCTTG
GGAACAGTTCAACAACCCGCAATTTGTCGTCGTTCAAAACCCGTCTGCGATCTTGCTCCGAAGGTCTGCGTTTAGGTATTACCAATCAAACGCAAAT
CATTGGAAGTACGTTTCAGTTGAAAGAGGTGCTGGTCATGCTCAGAGCACCGCCTTTCTCCCTGAATTGTATGGCAAGTTCGCACAAGAGTGGTGC
GACGATATTAATGATCTTCTGGTCCAACAGAAGATCCATCGGAAGTTTAAGGTCACAAACGGTCAGTTTGGACTACAGTGTGTCGAACAGGTGGATC
CGAGGGCGTCGCAACAACAGCTTTCGCCACGTCAAGTTGCTTTCCTCTCGTGGTTGAGGGGGAATATGTTCCCTAGTTACTACAGGGTACTTGACAT
CCACATGAACGTCGCAAAAGTAGATCCAGAACGACACGCTAAGTTCCTTCGTAAGCTTGAGAAAGGTTGGCGTGGTTGGGAAGAACTGTTACGTG
AAGGGGTGGATAATCTCTACGCCTTAACTGATTCCATTCCGGATGAAATCAGCAAAAAGTTCCAACCCGGCCTCGATATGCTCTATGCGGAGCAACC
GTTCTACACGAAGAACAAGGTGGTTGAAAAGTTTGTTTACTTGAAGCTTCTGGAGGAAAGCCCAGAGACGGAGGTCACATTTTCAGATTTTTCTGC
TCGGATTCAAGAGTCACCGTATGCGTCGGTGTGTGACCCCTATCACTTCTGGGAGAAGTTAGGGGAGCCTGAGTTCAAGGAGCAGTTGATGGCTGA
AGATCCTCTTAAGATACAAGGTCTCGTTTTGCTTATAAGCACCATGTACCTGTTAACAAACACAGCTGAGTCAGTAGTGTTGGCCATTCCTTTTGTTG
GTCTATTCTACAAACTCTTCCTGTGGTCCTTTATCGGACTCAATAAGGTATATGGACTGTTGAACACTATGTACTGGCATTCCGAAGGTAAGTCTTCGA
GGGAGATATCCCGAATTATGCCTCGAGATCCGTACATGGTGACGAAACAGTTCGCTGCGTTTTGCATCGACTTTGTACCATCACAACTGGGTTACTTG
GCCCTTATCCCATTGCACATATTGTCCTTACTTCCAGTGCTGTTTGAAGCACTTGGAAAAGCGTGGTATGTGGCAGGGGAGGTCAAGAATGTGATGG
AACCTCCTGGCAGCACTGTAAACAAGTGGGATCAGTACGCAGATGAGTACATACAAGCTCTGCGTTCGTCTCCCACGAACAGAAAATACATTGCTG
CCAAGACTTCAACTGGCAAATCAACCTGGTTTATAGCTGCCCTTTGGAAAGCTAGACACCGCCAAAAGGTCAGGAAATTTTGGCTTGTCGAACCTC
GATTGGTGTTGCGCGACAATACGCACATACCATTCGACGCACCTTATCAAGTGCTTAAGGATAACATCCAAGTTTCAAACACCCACGATATCCACATC
TTGACGTATGGACATTTACAGTCAAGGTTGTTGGATATAGACAATGAACGTGATATCGTCGTCTTTGACGAGTTTCATGAACAACAGGGTGAAATGAT
TCTTGGGCTTGACAAGTGCAAGGCGCCAATCTTTCTTCTGTCGGCCACGCCAGCCGAAATACCATCTCTGCACGGTTCTGGAACTCTGGTTCCAAAC
ATTGACAGACGACATCCAATCCAGATCCACCAAGTGGATGACGGAATGGGTGTTGTTGACATGTTCATGGAAGCCAGAAACCGTTACCCTGATTTAT
GCAAGAGAGCGATGATCATTGTACCAACTCACAAGGAGGTTGCAAAAGTTATCGCTTCCCTTGATTATCTCAAGGTAGGTAAGGTGTCCCCTCTGTC
CGCACGCGATCGTGTGGTGCAGGAAGAGGGAATACTGGTGTGCACCCCTTATGTCCAAACTGGTCTAGATATAAAACCACCACCCGATCTCTTGATC

GATTGTGGCAAAGACTTCGTTTTGGACAAAGGGCGTCCAGTATTTCCACTCCCGTGGACAGACAAGGACATAAACCAACAGAGAATTGGTCGTGTT
GGAAGATTAAGACCCGGAATCGTCTTCCAACCGAAATCAGCAGGTTCCGGAAAGAAAGGTGTGGTTTACCCGAGCCCACATCTATTCCAACATAAA
GCTGTCGCGGAACACTTCAAGGTCCCACAGCTCGCACCCACTCGTCAACTGCTGGTATTTTCGACGCAGTGAACATTGCGCCTTCGTTGCGCAGCTC
ACTTGCAATGATCTTATCGGCGTCAGTTCCAGAGCTCGCAGCTTTGTCAAAGAGGCCCTTCCACGTCTCGTTCGGGGTAAGATGAGGATGTGTTCCA
TGAATGCCTCCAAGACCATCTCTTCCATGAAAGAAGTCCGCCATCAACATTTGATCAGTAAGTGGATGATCGCTGCCAACAGCAACGATGGGCTTCG
TTTTGCGCAGAGCGTCAAAACCCAGGGGCTTGCCTTGAGCTGCTCGCCACTCGATCTCTTTCTCAATATGGAAGAACATGGAAACGACATTTCTTAG
ACAGTTTTCCACGTCGATATTGCCATAGGTGATCGATAGAAGTAGTACTTCGAGTTCTTCCGGCAGGGCAGAGAGTGCCAGTAACATTGCCATAACAT
CGTCCACCCCTGGGTCGGTATCTATGATGACTTTTCGAGCCATGGCTGTGATTTTGCTGATGAAAATTTGTGTGTCAAACCCCTACGCGTAATGAACG
TGACCGGAGGTGGGAACCGCAAGGTGCACCATCGACCGATCCTGATGTCCTCGGATGGATTTGAGTAAGAGCATAGCTGTTGGGACCCGAAAGATG
GTGAACTATGCCTGAATAGGGTGAAGCCAGAGGAAACTCTGGTGGAGGCTCGCAGCGGACCCTGTATCAGCTTGGCAGGCAGCAAGTCGGTTGAA
GGCCAGCCTCTCAACAGATTGATAGCTTCTTTCGCAGACGACATCTTTAGCGCAGAGTTCGCTCGGGGCTCGATAGCGGCGGCGGCTGTTGAGTTTG
ACCAGAATAGATTCCGAACGAGGCGGAGAAGATCGTTTCTGGGCATTGACGTGCTCGGAGGAAGTCCTTCTCGTCCTACCTGTCCACTGCTTGCTGC
CGAACGTGACTCCTCAGCCTTGCGAGCAACGGAAGGAGCTGCCCCTAACTTTCGTTCACTGATTAATGAAAACATCCTTGGCAAATGCTTTCGCAGT
AGTTCGTCTTCAATAAATCCAAGAATTTCACCTCTGACAATTGAATACGGATGCCTCACGAAGAAGTTGACCAGCGAAGGAGTAGAGATGGTAGCC
GTTTTCCATGCGGTGTTTCCAAACTGATGCGCTGGTCGCGACAAATCGTCCACTGGGGTCCCATTCAATATCTTTGATACCGTAGTGGTCGGCTGTGT
TCATGAGTTGGAGGTTGGCAGTAAGATCCTTCTCCTTCTCGTCCTTCTCACCCTCGAAGTCGAAGTCCCAGAAGTCCAGATCGAAGCTCTGCTGGTT
CAACACTGTGGCAACAATACAGAATCGGCCATTCGGTGACCAGTGAATGGCATTGTTGTTCTTCTTGTCCACGGTGCGAATGAGCTTGAAGTTGCCA
ACGGCATTACCCTTGGCCTTCTCAGGAGCGAAGAAGGAGACGGAAGTCTTGGGGGGAATAGCAGCACCGGCGGGCACTTCGCCAGCAGTTATGAG
GACGAAGCGATCACCTTTTGGCTCCCACGCGAAGTTGATGACGGTGTCCTTCAAGCTGTCGACAACTTCGACAGGCACACCCTTTTCTTGTACGCG
GAAGATTTCGAGGTTCGTAGCGAGCGACTTCTTCGATTTGCTGTGTCGATCCACCTTGCAGCAGAGGAATTTCGAGTCGGATTGCCAGTGGAGCTTC
GCGTCGCTAACGTTGAAAAGGTTTCGTGTTCGGACAATCTCTTTTGACGGAATAGACATGATACCGACCTTGGCCGGGTTTGAGCCCATCTCAGGTG
TCCAGTATGCGAATAGTTGCTCGTAGTCCTTTTGCTCAATGCGCTTTGGATTCGCCGGGCACCACTCAAAGTCCTGTACTCCTTCGATCTTCACGCTTA
CCTTGTCCATCAGTCGCATGCTGGGAAGTTCGTAGACGGAGATGCTCTGGTTGGGTGTCATTCGTGCAACGTATTTCGAGTCTGCAGACCACTTGAA
AGCTGGCCATTGAATCTTTTGCTTAATCGGGTTGCCTTCGGGGTCGAGGCCAGGGCTAGGAAGGTCGAGCGTAACGAAGGATCGAAGAGGCTTGCC
GGTCGCGACGTCCCAGATGATGTAGTTCTTGCCATCCTCCTCGAGCGAGAGGATAGGGTTGCCTTCGTCGACAACCATGGGGCGGTGCGACCACGT
TGTGACGTACTTCTCATTGGGCGAAAAGTCGACCAGGTTGACGCCTGGGTGCATGAATCGCTTTTGCCTCGACCACTGCGGGCCTCCCCACAGCTG

AACACCTTGCGCATGCATCGATGTCAAGTATGTGCCTTTTGGTGACCACTGCACGAATGACTCTGTCCAGTGTTGACGATCCACAATCTGGTCAGGC
GCAGCTTCCCTCTCGTTCCAGAACACGCCCACATTATCACCGCGGTACATGACGAACTGGTCTCTGCCATCTGCATCTCCCAACCACCATCGCAAGT
GCTCTTTCTCTTCGAATGGCGCGATCTCTGGTGCCTTGTATGTCTCGTCGATTCTTCCCTCACGACCGAATCGCTCAATATCGGTGAGCTTGTTGACAG
CCATGGTGTGCTTCTTGTCGAGCGGCTGCGCGTGAAGTGCCTTGACTGCGGCAGCGGCCTGTGCTGGAGTCTCATACTCGACGAAAGCGAATCCCT
CTGTCTGGCCTGTCTCGCCGCTGAGGGGCATGAACACATTCTCCTCCTTCGTCTTCCCGACCTTGGTCAAGCTGCGCAGCAAGAACTTGATGAGCTT
GTCCTTGCTGGCTTCGGGAACCTTCGGCAGGCCGTCGATGACAATGAAGGTGTCGAGGCCCTCCTCCATCACTGTCTCAAATTTCTCGCGCAAGTCT
GAGAAGTCGATCTCCTCATCGTCAACCTCGTACTCCGTCTCCGGATCGAGGTTCTCGAAAGATGGCGCCATGGTTGCGGTCTTGATGTGCGTGCGCT
TTCCGATGAAAGCGCTGGACGAGCAGATGGCGGCCGTGTATTGTTCCAGCAGTCACAACCATTTGCCGCGCTGCTGCAAGCTTCGGAGATGGCGAG
CCAAGAGTCTGCACGAGCTGGCAGGAAGTCGCACCACCGCAACGCTCAGAGCATGTCGAGTTTGCCAAACACTCCTCAGCGTAATCAAGGCATGC
GCGGTCAGCCTCCATTTCAGACGCCTCAAGCTCAACGTGGAATTCCTCGCCAATCTCTGGCTTGTTGAGGAAGTATCTCGTTGGCAAGACTGAGAG
GTCACCGAACTGCGATGTCCACTTCTTGTACAGCTGCTGGTCTCCTCCTCGTGGGAGCCATCATGGCTGCTCTATAAATGTTCTAAACGAAGAACGTT
GTATAATTTTGTGATGACAATAAAATACAGGCATTTAATGGGAATCAGGACAGGACACAAAAATGCATATTCTGATGATGGGGGATAACCTACTTGTA
ACGGCTGTAGATTTAGCGATGTTGTTAAAACGGGTGGGTTTGAGAAGTCGAAGAGGGTGAGGCGAAAAGATCGCAAAGTGGCGAGAATTGTTAG
GTCAGCAAAGAAAACCTTTCCAATCGAGGCCTATACAAAAGAAGTTTACAACCCATAGGGCCGTCATCCTTCACGCGGGATGGCTGGATCAGGCGC
TAACCCATTGTCCAACCCTTGCTGCATCCGCCGCCAAGATTGCGGGTGCAACTATTGACGGCCACCCGCTCACCTTCTGATATCCTGTATCGCCTGCC
GACGATGTATGCTTTCGGCAAATAGTGCCTCGATGAGACCGACGACCTCCTCAGCAGTGTACTCCATGCTCAACTCGCTCTCCTTCCACATGTACTTG
ATGTCTGCAAGACTGCTTTCCATGATCTCAGCCATGTATTGGAACTTCAGCCAATTTGGACAATAGACGAAAGTCATGTCGCGGTCGTTCTGCACAA
ACAAGTCCTTGGCCGTCGAGGCCTGGTCGATCAAAAACATGAGCTGTTTGGAGTCCGCCACGCCAATGTCCGGCAGGTCTTCAAATTCACGAATAA
TCTTCTGCGCGATCGATGACAAGAGGCTTCCAACACT

> Cb_clean_contig_4473
TGCGCATTAGGTTTCTGGCGCTACCCAGTTTTGTGTTTGCTGGATTCAAAAGTTGCCTTCGTGGCAGGAATCTGGTCAATGCAAAATTTGTGGATGGG
CGCAGCGCAGGATTTTGCGGCAATTATGGAAACACGGTCCTCGCTGGTTTGGGAGCCAGTGCAAAAGACCGTAGGAACAAAGAAGTTGACGTTCC
CTTTTCCCCTTGGGCACTGGAGCATGAAAGACAACCAAACTGGCAAAATCTATGAAGGTAGGTTTGTCGGTTCCTCCGAGACTATAGGAGGAAACT
TCCGCCTTGTGAGTACCACTCGTGAGTACAAACGTGGATTCAAGTCTTTTCCTGCTCCTTTCAATGTCGGGCATGCTGAGTCACTCGTGAAGGCACA
ACACGTGGCACCATACTCATCTTCACATAATTGTGTGACTATGATGGTGCAAATTTGTGCTAAGAGATCGACTGCGTGGACAGCATGCATGATAATGG

TTGGCGCCTTAACGGGACTTGCTCTGGCGCCGCCGAAATTGCTCAAGGACATTCTTTCTTTCCTTTTCCCTGGCAAGGATTTTGCCAGTACCCCTTTT
TATCGTTCGCTTGGCTTTGCCGCATCCCTGGAGAACATCCCCTTTGAGATAGAGGGGATGATGGAAACAGCAGAACCAATGCCAGAGGCAGCTACA
CCTGAACCAACGGATCTGGCTGATCCAGATTCTTTCAATATGTTGGTTGATGAAATAGCGTTCATCCAGGCATGCGTGGAACAACTCAAAATACCTAC
CTTAGATAAGGAGGCATTTGATGAAGTTTCACAGCGAACTTTGGAGAAAGAGTTGGACAAGGTTGAAATCCCTGAAGATTCGTTGATCAGGGTTTC
CAACATACCGCCAAGAGTGCATCATGAGTGGGCACAATTGGTGGACGGTCTCCACCACGCGATTTCTTTCGTGCGCACAAACCGTGTCGCTGACGC
ATTTGTGTTATACGTTAAAAACGCTGGTGAGAACATATACGACTTTGTCCAGCCAGTACTAGAGATCCTTTCCTTCTTTTTGCACCTTGCAGCTGAGC
ACTCCAAGCATGCTTTCGACCTCCTGTTCAAATGGCTCTGCGATTTCCTAGACCACGTGTGGGGTCTGGAAGCATCAAAAAGAGTTAAAACAGTGT
GGGGCCTAACTGGCCTTCATCGAACTGGATTGCTTGGTGTGAAAGCCAAACTTGCAGCCAACGTTGCTTATTCTGATTTCGTAGGTAGACAAGATTT
CGAATCAGACTACAACAAGTTGGTGCAAGAAGGCAAACACCTTGCTCAGGTATATGGTGCCGTCAAACGATCAAACCTTGGAGGGCCTCAGAGGA
GGCCTATAGGTTATGGAAAGCCTTTGATGTCACAATCAGAAGCTAGCCTGCTCGGTTTCGGGGCAGACGAGTTCGTGACTGATCGTGAATATGAGAA
CCGTGTGCAATCATATCTTGAACAAGGTGTTGCACAGGGTGCTGACGGAGTATTTTTGGCTGATAAGAGACCGGAGCTGATCGCCAAATCACAGCAC
CGCTATGAGCCGAAGTACCCCGCACTCACTTCAGAGGATCGTTCTATGGCCAAGGAAATAGCCCAAGCCATGTTTGATCAATATCCGGAAGTCTTTG
CTAATTGTGACGTGTTGCCACCAAGGGCAGTCCACAATTATGTAAAGCAAAAATATTCTCCAGGAACCCCATTCATCAAACCTGGGGGCTTCAAGTC
TCGTCAAGCAATGTTCGACGCTGGTTATGACCGCGTCATGCAACGACGAGCAATGCATTACTTGAAAACTGGTGAATATCCAGTACAATTCTATCACG
CTTTTGTTAAATCACAAGTGGTAGATATCAAGAAGTGCTTGAGCCCCGAGGAAGGTGGAAAAGGGAAGGACGTCCGCACTGTGGTTAGCCAAGATT
TGTTCTCCTACTACATTGACCAGTGTCTCCAAATTGAGCGCAACAAGCGCATAAATTGGGATACATATGGTGCAGGTATAGGTATGCCTCTTAACCAG
TCAATGGAGGCAATATACAATAGAATGGCGGCTGCACAGAAAACGAGAGGAGGCAGATACATCATCGCTGATGCAACCGCCTTCGACTCGTTTTGC
AAACCTTTCCTTTTTGAAGTAAATGCAAATCTTTGGGAACTCGGCTACAAAGATCATCCAAGCGGTAACGGCAAGAACCTCGCATCTGTTCTAAATG
CGTCGTATCAAGCCAGACAAAATGCTTGGATAATCGGTGTGACGGAACCAGAATATGACTCTTTGACTGTTTGCGTCCAAGACAAGAACAGTCGTG
ATCTCATAAGGCAACGATCCCCAAAGACAGTGATCTGTTTGGAAGATCTCATAGATTGGGACAAATACAATAGATTACCTGCAGAGCAACGTTCGAA
CTACGTTGCGAAACTTCGACCACCTGAAGGAAAGACGGTGGTGACCTGGGATCCTTCTCTCCGCCCATCCAGGTCAAACTGGATGGGTAAATATGA
ATATGGTGTCTTGTCTGATGTTGACAAGAAGTTCTTCGAGCATCAGACATTCACGTACGATCCTTCGAACTTTGAAGACATGTACCAAGACATATTAT
CAGTTTCAAGATCGAATTACAAACTGATCTCGAACGTACATGCGAAGAATCGTGGTGGAAGTACTGGAGGGTCAGACACATCAAATGTTAACACAC
ACGCTTTCAAAGCAGGTGTGATTTACGCTTGGTGCCAAACCACTGGCAGAGCTCCTAAGGAGTTCTTCCAGTACAATGACCTGGCCAATACATCAGA
TGACACCATTTGGCAATCGGGTGGCAAACATGGCCTCAACACTGTTGAAGATGTCGAAGTGTTTCGACATCATTGTCAATCAGTGGGCATTCATCTG
GAGATAGAGACAACGAAGAACATCTCACAAGTAGAATACTTGTCAAAATTCGTTCGCGTCCCTACTTCCGAAGATTCAGCAGCGCTGAAGGTCTGG

AGATCCCAGAAAATTAAGGCTGCTTCGAATGCCCATAAACAGTTAGGCCTGACTGTTCCCACTTCTTGGGAACAGTTCAACAACCCGCAATTTGTTG
TCGTTCAGAACCCTTCTGCGATCTTGCTCCGAAGATCTGCGTTTAGGTATTATCAATCTAACGCGAACCATTGGAAGTATGTTTCGGTAGAGAGAGGT
GCCGGTCATGCTCAAAGCACCGCCTTCCTACCTGAATTATATGGCAAGTTTGCACAAGAGTGGTGCGACGATATCAACGATCTTCTGGTCCAGCAGA
AGATTCATCGGAAGTTCAAGGTTACAAACGGCCAATTTGGACTACAGTGTGTCGAACAGGTGGACCCGAGAGCGTCGCAACAACAGCTCTCGCCG
CGTCAAGTTGCTTTCCTCTCATGGTTGAGAGGAAATATGTTCCCTAGTTACTACAGGGTACTTGACATCCACATGAACGTCGCAAAGGTAGATCCAG
AGCGGCACGCTAAGTTTCTTCGTAAGCTTGAGAAAGGTTGGCGAGGTTGGGAAGAACTGTTGCGTGAGGGGGTAGACAATCTCTACGCCTTAACTG
ATTCCATTCCAGATGAAATCAGCAAGAAGTTTCAACCTGGCCTCGATATGCTTTATGCAGAGCAACCGTTCTACACGAAGAATAAGGTGGTCGAAAA
GTTTGTTTACCTGAAACTTCTGGAGGAAAGCCCAGAGACAGAGGTCACATTTTCAGATTTTTCTGCTCGAATTCAAGAGTCACCGTATGCTTCGGTG
TGTGACCCTTATCACTTCTGGGAGAAGTTAGGGGAACCTGAGTTCAAGGGGCAGTTGATGGCTGAAGATCCTCTTAAGATACAAGGTCTCGTTTTGC
TCATAAGCACCATGTACTTATTGACGAACACAGCTGAGTCGATAGTGTTGGCCATTCCTTTTGTTGGCTTATTCTATAAACTCTTCCTGTGGTCCTTTG
TCGGACTTAATAAGGTATATGGACTGTTGAACACTATGTACTGGCATTCCGAGGGTAAGTCTTCGAGGGAGATATCTCGGATAATGCCTCGGGACCCG
TACATGGTGACAAAGCAGTTTGCTGCGTTTTGCATCGACTTTGTGCCATCACAGCTGGGTTACTTGGCCTTTATCCCGCTGCACATATTATCCTTACTT
CCAGTGCTGTTCGAAGCACTTGGGAAAGCGTGGTATGTGGCAGGGGAGGTCAAGAATGTGATGGAACCTCCTGGCAGCACTGTAAACAAGTGGGA
TCAGTACGCAGATGAGTACATACAGGCTCTGCGTTCGTCTCCCACGAACAGAAAATACGTTGCTGCCAAGACTTCAACTGGCAAATCGACCTGGTTT
ATAGCTGCCCTGTGGAAAGCTAGACACCGCCAGAAGGTCAGGAAATTTTGGCTTGTCGAACCTCGATTGGTGTTGCGCGACAATACGCATGTACCAT
TCGATGCACCTTATCAAGTGCTTAAGGATAACATCCAAGTTTCAAACACCCACGATATCCACATCTTGACGTATGGACATTTACAGTCAAGATTGTTG
GATATAGATAATGAACGTGATATCGTCATTTTTGACGAATTTCACGAACAACAAGGTGAAATGATTCTTGGGCTTGACAAGTGCAAGGCGCCAATCTT
TCTTCTGTCGGCCACGCCAGCCGAAATACCATCTCTGCACGGTTCCGGAACTCTGGTTCCAAACATTGACCGACGACATCCAATCCAGATCCACCAA
GTGGATGACGGAATGGGTGTCGTTGACATGTTCATGGAAGCCAGAAACCGTTACCCTGATTTATGCAAGAGAGCGATGATCATTGTACCAACTCACA
AGGAGGTCGCAAAAGTTATCGCTTCCCTTGATTATCTCAAGGTAGGTAAGGATGCTGAACCCGCGATCTCATCCCACGAAAATGCTCCTCGTATCGCC
CACTGTCTGTCATGCTCCGTTGTATGCGCCTCTTCCTTGATAACCTTCATTATTGCCCTTGCTTGCAGCTGTATGAAATTGTCTCTAGAACTTCTTCTCT
TCTCTTTTCTGCCGCTCTCATCTCATCCCATCTTTCATGACCCGTTCGTCTTGCCGGCCCAGCCCTTAGCAAAGCGATCTGATGCGGCGCCCGCGAGC
GTGCCTGGGTTGTATTCGGCTGGGACGTAGCCGCTTTGGGAGATTCCGCCGAGGAGGACGAAGATGGTGATGAGGACGAGGCCGAAGACGATGAG
TTTGCGGAGGGCGGGGTGGCGGGCTCCGTAGTCGGCCCAGGCGCGTTGGGCGCCGCCGAAGTGTTCCTTGAGCTTGTCTGAGCCGAGCTTGTAGTA
GACGAGACCTCCGAGGGCGATGGAGTAGCCGAAGGCTTGGAGGCCGGAGACTGGGTCGTGGAAGATCATCATGGAGGCTACGACGAGGAGGATAT
CCTTGAGGACACCGGAGAGGGTCATGACGAGGGATGAGGTCTTGCCGATCAGGAACACGACAGAGACGTTCAAGAGGAACGCGATCATGGCATTG

ATGATCAATGTCAGGTATCCGACTTTCTCCACATCTGCCAGAGTCATCTTTGGCACCTCAATGAAGAGCGCAGTGAGACCGTTCATGATGGCGCAAG
CTGGGGCGAAGTAGTAGAGTGAGACCAATGGGTCCATCTTGAACTCGGCGCTGCTCAGCAGACGCTGTACCATGACGAGACGAGTGGCTTCGAAG
ACGATTCCTCCGACCTGGTAGAGGAAACCGGTCATGTTGAATTGGATCTCGCCGTACGAGGCAATAACGACACCAATGACGATGAAGGAGACATTG
CCGAGCGTCTTGAAGTTGACTGGAGCAACTCCGAAGATCCAAGATGCGATGAGGCCGCCTTCGGCTCTGCCACGGTTGTTGTTGTTGTTTTGCTGTG
CTCAACCCAGCCTTCAATGGCCAGCGATCATTGTTCGCTATTCTAGCGGGTTCTGGCTGATTTACAATGCTCTGTGACGTCGTCCTGGCGACATATTAT
TAAGCTACCGCTTTATATCTACGCCGCCGCTGAAGCTTAACCAGCTGTTCTTTTTTACCAAAAAGCTTCCTTTCACTACATTTATTATGAAGTCCTCTC
GCGCCCATTACCGCCCCCTGTTTGGCGTGGGCGTCGGCCGGTCGCCGCCTTTTCTGGTGGCTGCACTCACCATTGGTCTCTGCATTCTGGGCGTCTCC
TACTGGCGATTGGCACTGCAGTATCATGATCTCGAAGAGCAGCTAAAGAGACTCATTCAGAAGCGGGACTCTCTGGAGGCTAATCAAAACTTCATCA
ACAAACAATTGGAAATACGTGAAGAGAACTTTAGTGAGGCTAAAGTTTCCTTACAGAAGAAGGAGCAAGAGGTGGAAGACCTGAAAAAGAAGCA
GGATCAAACGGGTAAAGAACTTGCTGAAATCAAGACAAGTTTAGAGCAAATTAAACAAGTTAATGATAAACTAAAAAGTGATACTACCGAAAAGGA
TCATTCGGTAGAGCTGCTTCGATCTCAAAACCAAGCGCTTGAATCTGATAAAAGCAAACTGAATGCTGAAATCAAAGAGCTCAAAGAAAAGCTGAC
AAACGTCACAAACACCCTAAACCAACACTCTTCGCTGCCATCATCATCTGCAGCTCTGAATCCAAATATTCCCAAGCCTGAATCGGCAGCTAGAAAC
CCCGTATACAAGCTGGTGAACCCTGCGGCCGGCAAACCTGAGACAAAAGTCGACATAAACCCGCCTCCACCGCTTCCAAAACCGAGCAACAGCAA
TGCCAACCAAATTGCACCTCCGCCGGAGCCAAAGAAGAATGTACCCTCAGAGGACAAGATGAAAGCGGAAGAGTCCAAACAGAACCCGGACATTA
TTGATCCTGCAAACATTGCCATCGATGATGACATGGAGAAGGACGACCCCGGTCACGGGGATGCGGCGCATGAGGACCTCGAAGGGGCTATGGAAG
GCCAAAAGGAGGGCAAAGTTGATAACTCAATCAACTATGTGGCCGACAACTCCAAAAACGCCGGTGGCCTGAACAACAAAAACTCGCAAGATAAT
CTCATTGAATCTATACAGAACATTTAGGGAAGCCCCCCCCTCAAAGATTGTACAAAAAAGCGCACTATCATTTTCTAAGTGTCATCATTTCTTTTCTTA
CACTGCATTTTGCCTGTTTGTGCCATCATTGTTCTTACTCACACTCTAGTAGTAGTAGTGGTTAGCACCCAAAGTATTACAGTACAAATCAATCACTTT
CATAATATGATCACTTTTTGAGTTTACTTCATTCTTGTCTGATTATGTCATGCATTTCGTATTTATAAGAAAAAAGAATACAAGAAAAGTTGGAAACAG
GGTGGATAAGAAAACAATTTACAAAAAATAAATTAAAGCAAATGAGTGTGAGTGAGGAGGAGAAGGAGATGTGAAGAGGGGAAAGAGAATAAATC
TACAAAAGGGACAGATATGAAACAGGTAGAGTGAGAGCGAGAGAAAACACAGATTACAGTTGGAAGAGCTAGAGGAGAGAGTTTTGCAGCAAGG
TTCAATGGTTCAACGGGGATTGAACGGAAGAGTGATACGGAGGTGTTATCAAAAAGAAAAGTTTTCAAGTAGCAAATATGGAAAATCGAGAAAAA
GAGTTAGTGCAAATGTCAAACAGTTTGAAAAATGGGGGGAGCGTTTAGCGAATTGTTCTGATTCAGAAAAAAAAACCGTGTCACAAAACCTGGAAAT
GAACATTTTTGAAGCGTGTGCAAATGGGGAGAAATGGAGAGGAAAAGAGGAGAAAACGAATTTGCCTTGAGTAAACACAGTATTATGGCTTGAAG
ACAGTGAGACGACCAAATGAAGAGAATGGATAAGTGCTTTTGATTATTTCGTAAGATAGAGAGAAGGGGTGATGATGAGGAGGGGGCAAATGAAGC
TTCACTTCAGCAAGCAAAGCTTTATTACCTTTGTACTATTTTGTTTCGAAAAGATCGGATTCATTACGGTTACACAGCGAAAATCATCAGTTGTTGGG

GGTGGTGCGACGTGAGAGGGTTCGGGATCGTCCACACCTCTTTCTCCCGCGTCTCCTTACTCTTCTGCGTCAGACTGTTCGTCGTCTTCGAAACGCG
ACAGATCAATCGTCTGGAGATAACCATTGCACGTGGCAAACTCCAGTAGCGCCAGACAGCAGAAGACATACTGATCGGGCATCTGAATACTGTGAG
CGCGTTGAGAGCGGATCTTCTCGACGGTGCCTTGAATGTCGATAACGCCTGATGCTTCCAGACGCTGAATGGAGATGTCAATTGTGATGAAGGTACC
AGTTCGTCCGATGCCTGCACTGCAGTGAACAACGATCGGCGGTCCTAGTGGGTGACCTTTCCACTCTATGTGCTTCAAGTATTGCTTTTGATAGTCTC
GGGTTTTCTGACGAAACTCCAACATTGCAACGGCCGATTGTGGTACACCAAAGTCCGGCCAACTGGAAAATTGCATGTGCACCACAGTTCTCTTTTC
CTCCGTGTCCTTGTTGACCAGTGTTAGATTGCTAATGACCCAGTCCTTGTCCTGCTCGATGGAGTTGTTGATGATCGTAAAGTTGCCCACCGCGCAGT
AGGTCTTCTGGTCGAGTGGCCAGTACTGATCGCACTTCACTCGACACCGCTCCACTGTCCGTGTGGTCATCACAATAACAATGCACTTGTTGTGCCA
AACCATTCTCCAAAAGTCGTGAAAGGTTCTCGTCAGTGGACCTTGGGTAGAGATGAAAGCTCGTTTCTGTCTGTAACCGTCGACATAGTTGGCATTG
ATGTAATCAGTACTGGGGTCGTCGTCAATCACCGGTAACTTTACTCTCGTGTGATCATAGCAGAGCACGTCAATGTACCGATTCTTTGTTGCATTCGCC
CTTGACCGTGCAATCTCAAAGGAGCCCGTAGGAGGATTGCTCTTGATAAAGCTGTACTCGTCGTGCAGGCCCTTCTTCCCTTTAATGTTCAGATTTTC
AATGAACTCCTGCAGTGAGACACCGGTAGCTTCGAACTCATCGGGAATCTCTTCATAGATTTCCAGCTCAGAGTATGAGGATAGTGAGCCGTTCTGT
GCGTCAGTGATCTCCGTATTGATGGCCAGTAGATCACATGAAGCATCACCGTTTGCGAGCGTTTTCGATAGATCACCATTTTTCTTTATCGTGCCCACT
GGAGAGGAGTTGGCGGAAGAGCCGGCAGAGGAGCCAGCAGGACTGCCGCTGATGGAGGAGGTAATAGTGGAGAACAGTTTTCTGTCTGCAGGTA
CTGAGGGACACAGATCACCCATGTCATCCTGGTGCACCTTCAGGCAGTGTGCAAGCCAAGTGCCATGATCAATTCTTAACGTGCCACCGAGCTCTTC
AGGCAAACACTCCTTCGGTATGTGTTGCGGTAGTTGGCTAGCGTTGATCATATGAACTCGATCACGCAGTTTCTCACGAACAAAAAGTTGCAGTATG
CGAAACGGTGCTTTGAACCACAGCGGCGCTGTTACGATGAGCACCTTCTTCAGCCGTGCTGGATAGGCGCCCTTTAGGAGGTTTAATATCTTCTGAG
ACAAGTCATAATCGAAATTGGTATATTTTGAGCCAGTCATGTTGTAGATGAAAATAATTCCGTTTCGTTGAGTATCGTAGTCATCGAGGGCCACATCAA
GCTGGTACACCACGCCTTGGAGTGTCGTCTGATGTGTCGTCTCAGTGGGAGAATGTTTTCCCGCAGAAAACAGTGCGATCATAGACCCATTTTTATC
AATTGCAGGCAGAATGGTAAACTTTCCCGTTTCTAGTTCTCGCTTCAGAGGATCTGTATGGGGATCAAACTTGACAATACCTTCTCTTATCCGTACTGT
TTCATGTGACTGGTACAGTTGAAGCGATCTTGGCACGTCAAACTTTCGAGCCATCAAGAATTTCAAAGCAGAGCGTGGCAGCATTTGCTTTTCGGCA
TTTTGATTGCGCGATTTGTTCACAATGTCGAGAAAGTCGTCCAACGCCTTTTTCTCAATCGGTGAAAGTAAGTTTATGTCCATGATATGGCAATGCGT
ACAGAGGAGTAAATAAAAGTGATTCCGATTTTGTAAAATGTCGGGGATGAAAAGAAGAGTGAAGAAGCCAGTGAAAAGCCGCCGCTGACATTAAG
AATTCTTTAAGTAAGTTAATGCGTGATTGCAAGTGGAAAAGTAGAAGGTTTGAGGAGTAGTGCCAATGACGCGGTTTCAAGAGATAAAATCGATTTA
ATTGTAAAGTGAAACGGTTTAGCTCTTATTCTTTTAAAGCGTTAAAACCTTCCGCCTAGCTACAGTTGGGGTGATGATGAAGAGTTGCAGCAATTATG
AGGAGAATTGAATACATTTTAAATGAACCGGAGAAATGTGGCCTTTGAGTGACGGAACTTGCTTCAATGCTTCAACGTGTGCGTTTGAGTTGAATGG
AACACCTGAGTGCGTTGACTGGGCGCAAATCGATTCAGGTGAGCGGCCTGTGTTGTTGGTAGATAGGACCGGGGGAGACAGGGAATGACAGCAGG

TGTTGAGCGATGTGCTAGAGCGTGGCAGGCGCAGAGGGCCTAGGTGCCGTGCATCGGTCCGTCATCTCGGATGCAGCAAAATGAACATCTGGCGCT
AGGGCCGAGTAAGAGTAAGGCCCAGGCAAGTACCAAAAACAAAACATTGGGCGACGATGTCTCCTCTTTGTGTGCCTCTCTCTCCCCTTCTTGAGC
TGATGTTAGCTGTTTTCT

> Cb_clean_k141_20910
GAGACAACACGCACATACCATTCGATGCACCTTATCAAGTGCTGAAGGACAACATACAAGTTTCAAACACCCATGATATCCACATATTGACGTATGGA
CATTTACAGTCAAGGTTGTTGGATATAGACAATGAACGTGATCGTCATCTTTGACGAGTTCCACGAACAACAGGGTGAAATGATCCTTGGACTCGAT
AAGTGCAAGGCGCCAATCTTTCTTCTCTCGGCCACGCCAGCCGAAATACCATCTCTGCCCGACTCCTCGCCATGCCATGCCATTCGATATGTATTCGC
ATCACCAATGTCAAAAACTACACCAGCAAGAAAGAAAAAAATTCTCAACTCCACTCGCTAAATCTCACGATCGCTGTCATAATCGTCCTGCGGCTGC
AGCAACCGCTGCTGTGGCCTCAACGGCTGCCACTGCGGCAGCTGCTGCAGCTGCTGTAGCCACAAGCACAGCTGCCGGAGCCACCGAAGCAGCTG
CTGTGGCGGCAACCGCGGCCGCGGCAGCTAGTGCGGCGTCGCTTGAGAGTGTTTGGGTTACGTTGCCAGCGTTAGCGAGGCAAACAGCGCTTACA
GCGGTAGCAGCCGTGCTCTTTATGGCATCTGATGTCTTTGTGGCCCGAGAAAGGTTCGTCAAAGCGGGCTCAGAAAATCAAGCCATGGGATTACCCC
TTTACTTTTGCTCTCAAC

> Cb_clean_k141_35224
GTGCAACCCGTTGTCTCCTTCATCATGTCCACAATCGAATCGGCATGTGACCCGGAATCGTCTTCCAACCGAAATCAGCAGGTTCCGGAAAGAAAG
GTGTGGTTTACCCGAGCCCACATCTATTCCAACATAAAGCTGTCGCGGAACACTTCAAGGTCCCACAGCTCGCACCCTAGGCTTGCTCCTGGAACTC
CTCATCTATCGCCATAGGGACTTGAGAGTCGCTCGGAAAGGTATCTTGACTCCGGCCATGAATGGGGATGTTGCCAGCGCTTGGGATGCCTGAGGCA
GGACTGACATCCGATACAAGGACAAGGACAATGTCCCGTATCATGTAGCATTTGAGGCAATGAACATGCGGATGTTGCGGGGGAACTGAAACATCTA
AGTACCCAGAGGAAAGGACATCAAACGAGACTCCGCTAGTAGTGGCGAGCGAACGCGGACCAGGCCAGTGGCTTATGTGAGTAAAGTGGAACAAT
CTGGAAAGGTTGGCTAGAGTGGGTGATA

> Cb_clean_k141_48216
GGAATACTCGTGTGCACCCCTTACGTCCAAACGGGTCTGGATATAAAACCACCACCAGATCTTTTGATCGATTGTGGCAAAGACTTCGTTTTGGACA
AAGGGCGTCCAGTATTTCCACTTCCGTGGACAGACAAGGACATAAACCAGCAGAGAATTGGTCGTGTTGGAAGATTAAGACCCGGAATCGTCTTCC
AACCGAAATCAGCAGGTTCCGGAAAGAAAGGTGTGGTTTACCCGAGCCCACATCTATTCCAACATAAAGCTGTCGCGGAACACTTCAAGGTCCCAC

AGCTCGCACCAATCTCGGGTGCTTTCACGGCAGATTTGCCTTTCTTGCGACCGAACAAGAAAGTGCTAAATTCTCTACCCGTCGAAAAGTCAGTTGT
TTTCATACATTCACTTGCTTTAAGTGGTGTTCGCCAAGAGCAATGGCAACACATGTATACGAGATCCGTGCAAGGGCACACTCTCGGCAACGACTAC
AAAAACGTAGATAGACTGCTTAATCATTCAAGGTACAAGCGATCAAAGCTAGTCTCCTATGGAGACGCGCTTTATCATCTTTACCGAGAACACGCAG
TCCAGTACGGATTTGTCAAAGAAGACGGAAGCACAGAGTACAAATCTGTCAAGCCAATGTCGGTCATCAACGGAAAGTGGATAGAGTTAGAACAAT
CGCCGACGACTAAGATGGATATTCAAGAGTTGCCGCAACAGGAACTTTCAAACAAATATCAATCTTTCGAATCATCAATAAGGCGATGTCGAGACTC
CATTCTTTTCAAAGCCAAGGACATGCCTGGGCATGACTTGGAACGTCTCAACTCCATCTTAGCGTTTTGAGCACTAAAAGGCGGCACTTGTAGCTGC
AATGCTACATTTAAAGACAACCGCAGTTTTGTGTTACCCAATTGATGTTTGACTTTTGTCAGACACGCTATTTCTGTTGTTGCCTCCCGACCTCTTTCG
TGACATTTGTCACATCCGTTCGTTACCTTTCTTTCTTTGTTCATAAAATACAAACAAACGCAGCGGCTAATGCCGCGGTAATAAACCGGATTCACATG
CTAACGCATGGTGTGGTTGGCCGTTTGCCAACGTAATCACCTTGTGACTTGGAGACGGTCCATTCCAAGCTTCGCATCATACGCAGTCTGTTTCACA
AAACCAGGATCGATAACGAAAAAGATGCCGTCGATTGTGATACTTGTCGATGCAATGTTAGTGGCGATGACCACCTTCCTACCACCAGGAGGAGGTG
GCTCAAAGATCTTGGAAGCAATCTCAGATGGCAATGCACCGTAGATGGGCAGGATGACGAGCTCGGGCACAGAAGGCCCAAGTGCTTTCATGCGCT
CGAACAAGATCTCGCAACTGGTGTCGATCTCTTCTTTGCCAGTTAAGAACAACAGTATGTCACCAGGCTTCTCAGTGAGATGAATCTGCATCACAGT
CGTCAAAGCTGCATCCAGATAATCGCTCTCCGGCTCCCGACTGTACATGATCTCTACTGGGAATGTTCTGCCCGGAATTGTCAAGATCGGGCATTCGT
TGAAATATGTAGAGAAC

> Cb_clean_k141_31123
TACTTGCAGAGTTGAAGCTGAAAGTGCACTGTTTTCCCCATCCCGGACTTCTGGTCACATGCAGAAGTTGATGGATGGCGTGTTGGCCCTTCGTCGC
TCTGGTCAGATGACCTGTGTCAAGAAGGAATCTCCGGCTGAGTTCGCGCGCCGTAAGTGTGAACAGAAGTGGTCTTGGCTCATCAGGCGTCTGCAG
AGGCGTTCAGCTGCTGAGCAAGCTGTGATCGCTGAACAGCGGTTAACAAAGGCCCTGCATTTGCAGGCCAATACTTCTTTCTTCGTCCCTACTGGGC
CGAAGGGTTTCAAAGGTCTGGGAGAGTTGGTACTCCCTACCCCTTCTGCTGAAGAGATCGCTTTTGGTTCCTCTTTCCTAGATGGCAAACGTGTTCG
TGTTTTGCCAAAGAGAGAGAAGAACACTCGGTTTTCCGCTTGGTGCCAGTCAAATGCCGAACGTAAGAAGGCATTAAAACTAGCCGAGCTGGAAC
GTCAAAGGTTTAGATTGTCCTCGTTGGTCGCTGATCCTCTTCGTGTGCTCGGATGTCAAATTCCTGAGACATTCAGTGGTGTCATTTCTTTCGCCCGT
GGTCTTTTTGGCACTTCTTCACGTCGCTCTGTCGTCCGAAGGGATTTCATCCATCGGGCTAGACACGCTGAGAGGTTCTTCTTCTGTCCTGTCAATGG
TTACATTATTGATCTGGTCGGGAACAAGATAAGGGTGTCCAGAAATAGGGCGGGATCTGGTGTCTCTTTTCGCATTGATGGTGGTCAGGTCGCCCACT
GCGGCCTAAAGGACGAGTATCTTTGTGCAGTTCTTCAGTCCAGGCTAAATCTCATACCAAACCCTGGCGAGCCGGAGCTCCTGACTATCGAACTGTT
TCGTTCGAGATACGAGGACTATTTCGGGAGGTTTTTAACTGAAATGGACGGAACCAACCTCGACTTGTGGTCTTTAATGACCATGGCCAAAAGGTGG

AAAAGAGATTTTGGTTGCCCTGGCAACACTTGCATCTTGTCTTTTGCGGGTGATTCGGTCTTGTTTACTGTTGTTAACGAACAAGACGATCTGCCGCC
GGCTTTGTGGTTTCGCCTTGATGGCAAAGATTTGTTCCACTGCGGCGTTGACCAGGTCAAGAGACTGCAGTTCCAGAAGAACATCTGGAAAGCAGT
TGCCAAGATGGTCATCCAGGACGGTAGCCCTGCTGAACAGCGGTTGGCAGAGAAGGTCTCCTCCTCCGCACACGTGAGTGTCCCTTGCCTGAAACC
TAACTTGTTCCACCAGTTAACAAACGGTTTCAGGGTCTGCGCCAAGTTCTCCGGGCTCAGCGTTTTTGAGCGCGGGCTCGACTTTACACGCCCTGAT
CCTCGGGTCGAAGGCGGCTTCATCACAACCAGACAGGAGAGGTTGTTCTGGGACGTAGGGGCAAGCAGGTTGGTGTTCACACCAAAAGGCCTGGC
AAAAATATTCTTCCGCAAGCACAATCAAGAAAAATGGAGGATAGCTTTGGCTGCTGCGATTGGTTTGACCTATGATGAAGGTCTGCTTTTCTTTTCAA
AGTTTCACAATGATAACACGAAACCAGATGTTGTGTTGGAGACTTTGGGTTCGTCACCCTATCACAAGCCAGATAGGGAAACAGTGACAAGAGTGG
CTTTTGTTGACGTGGAGAAACCTGTCGCACCCAACACTACGCCTCAGTCTTCCACAGTTGTTCCGCCTCCGCCGCCTCCACCGCCGGTGCAGGAAG
TCAAGCAGAAGAGGAAGAAGACAGAGTTAGCTGATGGTGACACGTGGCTTCTTGCTGAAGGTTCTGAGGGCAAGTCAAACAGAGCTCGTTTTAGA
TCTCTCTTCCCACATGATTGGTGGTTGAGGGTGTCTGAGAGGGTTATGCCTTTCATGAATCAGGATGCGTCTCGTGACGTCGGGTACAGATCCAGATC
TTGGTCTCAATCTTCCCTGGTTTCAAATCCTTCAGAGAAGTTTGATATAAACACGGTGAAGCACACACCTTCTAGTTCTTCCAAGGCATCAGGTTCAA
CGCCTTTGCGGGTTGATCCTGAAGCTTTCAAGAGACAGCTTCAGGCTAAAGCTAAGAAAGTGAGTGCAACGTCGTCCTCCCGTCCCGATGCGAGGG
AGGTGTATCTGAAGGGTGTTGGTGAGTTCAAACCGGACAAGGTTTCTCACAGAAAAGCCCTTTCTGAACCTACTCCGTCGAGGAAACCTTCCTCTG
AGTGGATGTCTATCTCTCGAGCATCATCTGATTATCTGTTCGATGATGATCATGAAGACGCAAGAGATTCGACTAGATCAGACGTTGAGACTAACCCT
GGACCTTCGATCAGGCATTTCCGTTCCAAGTCGACTTCAGATTTGGAGGGTGAAACACCGTCTCTCAATTTTACACCACCTACCCCTGGTGATGTCTT
GACTGTGCACCATCCGACTCCACCTCGTTTGAGGGTTAATCACAGGATGAATAGACACACTGTGATTGTTCCTTCACCGGGTGAAAACACGTCGAAT
TGGAATGTTGGTTACTCGTCATTTGCGTGGCCTCCTACAGAGGAGCATCTGGAACCTCGAGACTCCAGAACCGCGGATATAGAAGAAAATCCCGGG
CCAAGTGAAACCCTTGACCCCACTTTGCCCCAGTTCCGGGGTCGAAGTGGTTCACGGAGTTCAATCGCTGTTGCCACAGTGGAGCGATCACCCTCT
AGAACGCGTAGGTTCTTTGGAAAGATCGGCATTCACGGCAAAAGCGTGACGGAAGAAGTCAAGGACATTTTTGGATTCAAAGAACATGACGAGGT
GTATCCTTACACTGGCAAAAACTCATCGTTCGTAGAGAAACATTATGAATTCCACTATGTCCTTTCTCATTTACCTAAGGACAAGAAATGGCGGTCGT
CAACCTTTATTAGGTGGGTTGCCGCTGCTTCTCTGTCCGAGCTTCAAAAATCACTTGACTGTGTAGTGAAGGCTTCACATCCTTTCAACACCGCGCTT
GTGAGTAAGCTGCGTGATGAGACGAACTCCTTCTTTCAGAAGATCTCCGATTCTGAGCCGATAGGTGGTACGACCGTATCTGCCTTAGCTCATACATT
CAAGACTCTGTACGCCTGCCCTGACAATGGGTGCGGTTACGCACTAGAGATAAAACCAGAAGATGGAATGGTACTATTTCATTTGACTGAGTCTCCA
GACGAAACCGTTAAAAACAGGTTGTGGTGGGTTTTGAATGGGAATGAGATTTTGCATGGAGGGGACAAAACCCCTGTTTCCCCAAGCACCGTCTCA
TTCGGTTGGCTAGCGAAGGAATATTCGACACATGAGGTGATCATCGAGCCCAAACCGGACTCTATGCTATCACAAGAGAAACCGGGGCCGTTTGCA
ATGCCCTCGCTTTCTCAGTTTCAAAAATGTGTCGACAAGATTATCCCTTCTGGTAGTCATTTCTCTCGTTCTAAAGTCGGTGAACTCAAAGATCACAT

CAACCAGTACCTGGAATCTACAGATGTCAAGACAGTAGATCCTGTGTTGGTCGCGGCGATCGCAATGAGTTTTAAGTCGAAATTCAATTGTCCAAAA
GAAACCAGCTCTTTCGCAATTGATTTCAGACTCGACCGAAGAATGGTTGTCTTCTGTGCCACAGATTCCCCTGAAGATAAGAACAAAGGGAAACTC
TACTGGTGCATCGGTGGTAGTGTTATCGTCCATGGCGGGTTGTTAGGCATTGAAGATGGGAAGTCTTGTTCCCCATCGTTAGATCACTATAGCAAGTT
ATGGACGATGCCACTTCCGAAATCAGGTTGGGTGGACAGATCTTTTGAAGCTGAATACTTCACAAAGATTTCAACCTGGCAAAAGGCCCTCGATCG
GTGTGAACCGATCGTTGTATTCTCTGAACACACATTGCAACGCACACTCTACCAGTATTTTGATGGTGTGACAGACAAAGTTTTGTCCCCATCACATA
CTGCAATTGTTGCTTGTGTGGTAAAATCCTTGATGAAATGCCCGGAGAACGGGATGGGATATGCAGTGCAGTTTGATAGCCATGGGGCTTCACTGTA
CATATGCAGGCAACCGAATTCCGCAGAGGCGAACCGGTTGTGGTACTTTCATTGGGATTTCCCTTTCGTGCACGGGGGAAGAAAATCTTATGCTCCA
GAGCCTAGGCGGTTCTTTTATACTGAGCATCTTAACATAGATTATTCCATACCCATGCATGAGGTTTTTGTTGGAAAGGATCTACCCAAGTCCAATTCT
TCGCATTTGACTGTCAAGAGTCAACAAGAATTGTATTTCGGTTTTCAACAACAAATTGATATGTATGATGCGCCAACACCGGCCTTCATGCATTCAAG
AGCACATGGCACAGCTGAGCAAGCCGTACGTGACACCGTGCCCAAGATGAGAACTTCCCAGTTCAATTTTGAGCACATTGCTGCGATAGCCATGTG
TTATAGATCCAGGATGCAATGTCCATGTCCGGAGTCTGCTATGGCAATCGATTTTGACTTCGCCAATGAACGCATTGTGTTCTTTTCGAACCTAGGCA
GTGGGTATTTCCGCTGTCGGGGCCAAATGTGGTGGTGTATAAACGGAGTTGGTCTGATTCATACTGGGTCAGTGTTTCACACCTACCACAACCAATC
AAACCAATCTCGACCTAGTCCCCGAGTGTGTAGGTACTGGGACGAGATTGCTTCTTTTCATTTGACCGAATTCTGCCAACCCACATTGGGTTATTGGA
CATCGGATTTTCACCTGCAACCTGACAAAGAGGAACAGTTGCAGAGGGCTCAAATTGCACTGGACGCACATAATGATCCTGTTGAGCCTTTCAGCA
GGGACATTGCAATATTTCATGCGTTTGAAGCGATCAGACAAACGGCCCAAGCGGTCGATATAACTGTCATGAAGATTGAACATTTGATCGTCATGGCA
TGCATATATCGTTCGAAAATGGCGTGCCCTACACCAGGCACTTCAATGATTGTCCAGTTCGATTTGTCAAACCAAAGAGTGAGGTTCGGCGTTGCAA
GGGGAACACATCCGGAGTTGGATGGCAGAATGTGGTGGTATCTGCACGACGATTGCATGATCCACACTGGTAGGCCAGAAAAACCTCACACTGGTG
AGGTTGAAACTCCGTTGCCCGTCAGCAGTCGGTTTTCTTTTCGTGCAACTCATCGTCAGGCCAAGGTAATGGCCAACGATTTTGAAGAGAAGTGGA
GAGAAGACTCCAAGTGCAGAGCACGGACATGGCACATCGAAGGTGATGACACACCGAAGCAAAAGCTTGCTCGGTCCCATCACCCTTTGTGGTGT
CCAGACACGTTCAACGAGCGGTTCGACGAGATCTTCGTTGCCGCTTACATTTGGTTTTTCTTTCAACTCATTTGCACGTTTTATTTTTCATTTTCTCGA
CCAAATCTTCGGCGAAAACCCGACAAACACACAGAAGGAGGAGAGCATCCTCTTGGAGATGAGAGTTTTTCTGCATCATCCTCCTTGCTTGTGTGC
AAGTTAGGAACCAGAGGTGACCAAGTCCCTGTCGATTATTACGCGAACGTGGCAGCACACTATGGTGTCCGCACACATTCGCACGTGTACAAAGAC
GTGACGACAGACGATCTTGAAAAGATGAAAGTTGGAGACTTTTCATCTAGTTCCGCTGGTTATTTGAATCTTGCGTACGCTTCACAACTTGGTTATAA
AAGAGTGTTCACCCCGCATGTGGAGGTAGAACGGTCCCAAGGTGTGAGCTATACCTTGTCACCGAGTTCAAAGTGGGTGGAAGCAACAAGGTTCAT
TGATGATTGGTCAAAGGTTTCTTGGTCCAATTTCCTCCCCGCGTGGATAGCCACGAGGGTCACTGCTGTGTATGTGCCAACTTTTAGGATTGGTGCAT
TGTCCGGAAGTGACATCCCGCGTTCTCATGACGGGCTGAACCTGCTGGAAAAAGTAAAGAACACAGCGAAGTTCGATGAGGGTTGGACATCTGGC

AGCTCGTCAGTTTCAGTAATCCCTGACGAGATCAAGTTGAGATGCCCCGAAATACCATCGGGTAACCATCTTGAAATACTAAAGGATTACAAAACGG
TGCACATTCATGGTGGAGCCGGTACTGTGCAAACTGCTATTGCTTGCGGCGCACGACCGGTCATCCACGACAAGACACTCGATAGAGTGTATCATAC
GATGCCTGAGCCAAAGGATTTCAAACAACCTTCCATCGGGAAGTTCATGGGTTGGTTGGTATCCGCCGGTTTCAACGTTAATGCACCAATAGAGGTA
AGGGCGTGGTGGTTGTTACAATGGTATTGGTCCCAAAAGTGGAACTATGCGTACGACGCATTAGTTACACTTGCCAAAGCTTACACCATATCATCGTA
TGTGTACAATCATTGGGAGCTCTTCCTCATTCTTTTCTTCACCGTTCCGGCAGTGGTCTGGAGAATGTTATTCTCTTCCAAATCATTGTTCTCCATAGTC
AAAATTTGTGCGCTTGGTTTCTGGCGCTATCCTGTGTTGTGTTTGTTAGATTCGAAAGTCACGTTCGTGGCAGGAATCTGGTCAATGCAAAACTTGTG
GATGGGCGCGGCGCAAGATTTGTCTGCGCTCATGGAGACACGATCTTCACTGGTTTGGGAGCCAGTGCAAAAGACCGTAGGAACGAAGAAGTTGA
CGTTCCCTTTTCCCCTTGGGCATTGGAGCATGAAAGACAACCAAACTGGTAAAATCTACGAAGGTAGATTTGTCGGTCCCTCCGAGACTATTGGAGG
GAACTTCCGCCTTGTAAGCACCACTCGTGAATACAAGCGTGGATTTAAGTCTTTTCCTGCCCCTTTCAATGTTGGCCATGCAGAATCTCTTGTGAAGG
CCCAACACGTAGCACCATACTCATCTTCACATAATTGTGTGACTATGATGGTGCAACTTTGTGCCAAGAGATCAACTGCATGGACAGCATGTATGGTG
ATGGTGGGCGCCTTAACGGGACTTGCCCTGGCGCCGCCAAAACTACTCAAGGACATTCTTTCCTTTCTTTTCCCTGGCAAGGATTTTGCCAGTACTC
CTTTTTATCGCTCGCTTGGCTTTGCCGCGTCGTTAGAAAACATCCCATTCGAGATAGAAGGGATGGTCGAAACGACAGAGCCAATGCCAGAGACAGC
TACACCTGAACCGACAGATCTGGCGGATCCAGATTCATTCAATATGTTGGTCGATGAGATAGCGTTCATCCAGGCATGCGTGGAACAGCTCAAGATA
CCTACCTTGGACAAGGAGGCATTTGATGAGGTTTCACAGCGAACTTTGCAAAAGGAGTTGGACAAGGTCGAAATCCCTGAGGATTCGTTAATCAGG
GTTTCCAACATACCACCGAGAGTGCGTCATGAGTGGGCACAATTGGTGGATGGTCTTCACCATGCGATTTCTTTCGTGCGCACAAACCGCGTTGCTG
ACGCATTTGTGTTATATGTTAAAAACGCTGGTGAAAACATATATGACTTTGTCCAGCCGATACTGGAAATCCTTTCCTTCTTTCTGCACCTTGCAGCTG
AGCACTCTAAGCATGCGTTCGATCGCCTGTTTAAATGGCTTTGCGATTTCTTAGACCATGTGTGGGGTCTAGAAGCGTCAAAGAGAGTCAAAACAGT
GTGGGGCCTGACTGGCCTTCATCGAACGGGATTGCTTGGTGTAAAAGCCAAACTTGCAGCCAACGTTGCTTATTCTGATTTTGTAGGTAGACAAGAT
TTCGAATCAGACTACAACAAACTGGTGCAAGAAGGCAAGCACCTTGCTCAGGTGTATGGTGCCGTTAAACGATCAAACCTTGGAGGGCCTCAGAG
GAGGCCTATAGGTTATGGAAAACCTCTAATGTCACAATCGGAAGCCAGTCTGCTTGGTTTCGGACCAGACGAATTTGTGACTGATCGTGAGTATGAG
GATCGTGTGCAATCATACCTTGAACAGGGTGTTGCACAAGGTGCTGACGGGGTATTTTTGGCAGACAAGAGACCGGAGCTGATTGCCAAATCACAG
CACCGTTATGAACCAAAGTATCCCGCACTCACTTCAGAGGATCGTTCCATGGCCAAGGAGATAGCCCAAGCCATGTTTGATCAATATCCGGAAGTCT
TCGCCAATTGTGACGTTCTGCCACCAAGGGCAGTCCACAATTATGTGAAGCAAAAGTACTCTCCAGGAACCCCGTTCATCAAACCTGGAGGCTTCA
AATCTCGTCAAGCAATGTTCGACGCTGGTTATGACCGCGTCATGCAACGACGAGCAATGCATTACTTGAAAACTGGTGAATATCCAGTACAATTCTAT
CACGCTTTTGTGAAGTCACAAGTGGTAGACATCAAGAAATGCTTGAGCCCCGAGGAAGGTGGAAAAGGGAAAGATGTTCGCACTGTGGTCAGCCA
AGACCTGTTTTCCTATTACATTGACCAATGTCTCCAGATCGAGCGCAACAAACGCATAAATTGGGATACATATGGTGCAGGTATAGGTATGCCTCTCA

ATCAATCAATGGAGGCAATATACAGTAGAATGGCAGCCGCACAGAAATCGAGAGGAGGCAGATACATCATCGCTGATGCAACTGCCTTCGATTCGTT
TTGCAAACCTTTCCTTTTCGAAGTAAACGCAAATCTTTGGGAACTTGGTTACAAAGATCATCCAAGCGGTAACGGCAAGAACCTCGCATCTGTTCTA
AATGCGTCGTATCAAGCCAGACAAAATGCGTGGATAATCGGTGTGACCGAGCCGGAGTATGACTCGTTGACTGTTTGCGTCCAAGACAAAAACAGT
CGCGAGCTCATACGGCAACGATCCCCAAAGACGGTAATCTGCTTGGAAGATCTCATAGACTGGGACAAGTACAATAGATTGCCTGCTGAGCAACGT
GCGAATTACGTTGCAAAACTTCAACCACCTGAAGGGAAGACGGTGGTAACCTGGGATCCTTCTCTCCGCCCGTCCAGGTCAAACTGGATGGGTAAA
TATGAATATGGTGTCTTATCTGATGTTGACAAGAAATTCTTTGAACATCAGACTTTCACGTACGATCCTTCAAACTTTGAAGACATGTATCAAGACATA
TTATCAGTTTCACGGTCAAATTACAAACTGATGTCAAACGTGCATGCGAAGAATCGTGGTGGAAGCACTGGAGGGTCAGATACATCAAACGTTAATA
CACACGCTTTCAAGGCAGGTGTGATTTACGCTTGGTGTCAAACCACTGGCAGAGCTCCGAAGGAGTTCTTCCAGTACAATGACCTTGCCAACACAT
CAGATGACACCATCTGGCAATCGGGTGGCAAACATGGTCTTAACACTGTTGAAGATGTCGAAGTTTTTCGGCATCACTGTCAATCAGTGGGCATCCA
TCTGGAGATAGAAACGACGAAGAATATATCACAAGTAGAATACTTGTCAAAATTCGTTCGCGTCCCTACTGCAGAAGATTCAGCAGCATTGAAAGTC
TGGAGATCCCAGAAAATCAAGGCTGCTTCAAATGCCCACAAGCAGTTAGGCCTAACTGTTCCTACTTCGTGGGAACAGTTCAACAACCCACAGTTT
GTCGTCGTGCAAAACCCTTCTGCGATCTTGCTCCGAAGATCTGCGTTTAGATATTATCAATCTAACGCAAACCATTGGAAGTATGTTTCAGTAGAGAG
AGGTGCCGGTCATGCTCAAAGCACCGCCTTCCTACCTGAACTATATGGCAAGTTCGCACAAGAGTGGTGCGACGATATCAACGATCTTCTGGTGCAA
CAGAAGATCCATCGGAAGTTCAAGGTCACAAACGGTCAATTTGGACTACAATGCGTCGAACAAGTGGATCCGAGAGCGTCGCAACAACAGCTCTC
GCCACGTCAAGTTGCTTTCCTCTCGTGGTTGAGAGGAAATATGTTCCCTAGTTACTACAGGGTACTTGACATCCACATGAACGTCGCAAAAGTAGAT
CCAGAACGGCACGCCAAGTTCCTTCGTAAACTTGAGAAAGGTTGGCGAGGTTGGGAAGAACTGTTACGTGAAGGAGTAGATAACCTCTACGCCTTA
ACTGATTCCATTCCAGATGAAATCAGCAAAAAGTTTCAACCCGGCCTCGATATGCTCTATGCAGAGCAACCGTTCTACACGAAGAACAAGGTGGTCG
AAAAGTTTGTTTACCTAAAACTTCTGGAGGAAAGCCCAGAGACGGAGGTCACATTTTCAGATTTCTCTGCTCGAATTCAAGAGTCACCATACGCATC
GGTGTGTGACCCCTATCACTTCTGGGAGAAGTTAGGGGAACCTGAGTTCAAAGAGCAGCTAATGACTGAAGATCCTCTGAAGATACAAGGTCTCGT
CTTGCTTATAAGCACCATGTACCTGTTAACAAACACAGCTGAGTCAGTGGTGTTGGCCATTCCTTTCGTTGGTCTATTCTACAAACTCTTCCTGTGGT
CCTTTATCGGACTCAATAAGGTATATGGGCTGTTGAACACTATGTACTGGCATTCCGAAGGTAAATCGTCGAGAGAGATATCTCGGATAATGCCTCGG
GACCCGTACATGGTGACAAAACAGTTTGCTGCGTTTTGCATCGACTTTGTACCATCACAACTGGGTTACTTGGCCCTCATCCCGCTGCACATATTATC
CTTGCTCCCTGTGCTGTTCGAAGCACTTGGAAAAGCATGGTATGTGGCAGGGGAGGTCAAGAATGTGATGGAACCCCCTGGCAGCACCGTAAACAA
GTGGGACCAGTACGCAGACGAGTACATACAAGCTCTGCGCTCTTCTCCCACTAACAGGAAATACGTTGCTGCCAAAACTTCAACCGGCAAATCGAC
CTGGTTTATAGCTGCCCTATGGAAAGCTAGACACCGCCAGAAGGTCAGGAAATTTTGGCTTGTCGAACCTCGATTGGTTTTGCGCGACAATACGCAC
ATACCATTCGACGCACCTTATCAAGTGCTCAAAGACAACATCCAAGTTTCAAACACCCACGATATCCACATCTTGACTTATGGACATTTACAGTCAAG

ATTGTTGGATATAGACAATGAACGTGACATCGTTATATTTGACGAGTTTCACGAGCAACAAGGTGAAATGATTCTTGGGCTTGACAAGTGCAAGGCG
CCAATCTTTCTTCTCTCGGCCACGCCAGCCGAAATACCATCTCTGCACGGTTCCGGAACCCTGGTTCCGAACATCGACAGACGACATCCAATCCAGA
TCCACCAAGTGGATGACGGAATGGGTGTTGTTGACATGTTCATGGAAGCCAGAAACCGTTACCCGGATTTATGCAAGAGAGCGATGATTATTGTACC
AACCCACAAAGAGGTTGCAAAAGTTATCGCTTCCCTTGATTATCTCAAGGTAGGCAAGGTGTCCCCTCTGTCCGCACGTGATCGCGTGGTACAGGAG
GAAGGAATACTGGTGTGCACCCCTTACGTCCAGACGGGTCTAGATATAAAACCACCACCAGACCTTTTGATCGATTGTGGCAAAGACTTCGTTTTGG
ATAAAGGGCGTCCAGTATTTCCACTCCCGTGGACAGACAAGGACATAAACCAACAGAGAATTGGTCGTGTTGGAAGATTAAGACCCGGAATCGTCT
TCCAACCGAAATCAGCAGGTTCCGGAAAGAAAGGTGTGGTATACCCGAGTCCACATCTATTCCAACATAAA

> Cb_clean_k141_49715
CGTATGCTTCGGTGTGCGACCCCTATCACTTCTGGGAGAAGTTAGGGGAGCCTGAATTCAAGGAGCAGCTAATGGCTGAAGATCCTCTGAAGATACA
AGGTCTCGTCTTGCTTATAAGTACTATGTACCTATTGACAAACGCAGCTGAGTCAGTAGTATTGGCCATTCCTTTTGTTGGCCTGTTCTACAAACTCTT
CCTGTGGTCCTTCATCGGACTCAACAAGGGCAACTAACACCACTCGACACTGCACGACCATGTTCAAGTACGTAGTGTTCGCCTCCCTTCTGGCCAT
CACCACCGGCCAGGTGCTCAAAATCTCCAACGCCATCGTCGGCTCTGCTCAACCAGGAGCTGAGCAAATCATCCAACTTTCTGGAGGTGGTGGCGG
CCCTGGTGGTGCCCGAGGTGGCAGCCGAGCTTCGGCTGGTGCCAGTTCTTCCTTTGGCGGTAGTTCGAGCGGCCGACGAGTTTCTTCTTCTTCTGGC
GGCGCTAGCTTGAGCGGTCTTTTGGGCGGCGGCAGCCTTAGCCGAGCATCGGCCGCCCCGGCCATCACCCTCGGTGGTGGACAAAGCACCGCCGG
CCTCGGCAACATTGTCCTCCTGCTGCCTCCCAGTGCCATCCGAGCTCAGGCTGCCCCCAGAGCTCAGGCTGCCCCCATTTCCATTGCCCCATCTCGTC
AGGTGTCCTCACTGGGTGGACTTTCCTCCTCCTCCCTATCTTCAGGCAGTGGTTCGCGCCTCTCCTCCAGCGGCAGCTCCTCCAGCTCCAGCGGTGA
AAGCGAGAAGCCAATGCCGTACTCGTTCAGCTGGGAGTCGGAAGACGAGTTCAAGACTAAGCAGTCCCGTCAAGAGACGGCTGACGAGAAGGGC
ACCGTTACCGGCGAGTACTCCTTTGAGGCTGACGGACTGAAGCGAACGGTGAAGTACATTGCTGATGAAAACGGCTTCCGAGCTCAGGTGGAAAC
CAACGAACCGGGAACTCTCACCTCAAACCCAGCTGACGTGGAGATCAAGTCCAACGCAAACAACTAGACAGCCAGGAATAGTTCAAATAATTTAA
AACAGTTTATCATTTTTTAAGCTTTAAAATACGTAATTTCAAAATTTTTTCAACAGTTTTGCAAATTGTAAATAGTAAATTTTCTCCTGTAAGTGTAGTT
TACCAAAATTGCACTTGCAACTTTTCTTTCTTCAAGAGCACAAGTTGTAATTGAAGACTTTCGAGCAATCGCCTTCTATCTTTACACATAAACGGTAC
CCAGTGTGCACAGCAAACTTGATTATTTTGTACAGTGATAGTCCCAGCCAAATGCCTAAAATGAGGTCAATTTAGAAAAACCGTTTGCAAAACGTTA
ACTCAAAAACCATCTCACCCAAATGACCGCCAATAGTGCCTGGTTAGAATTTACTAAAATTAGTTATTTCGACTTTATACCTTTGGCTCAATTCACTTA
CCG

> Cb_clean_k141_56835
CTGTGGTGCTCTTACACAGGCCCCTCAGGGGACATATATAAGAGATGATTGCTCCACGCTTTCAGTGGATTCCATTTGTCAAAGGTGGTTTTCCTTTG
TCTGGAAGCAGATAATTTCTGCAATCGGATCCTTGCGGATCGCGGCACGTGCTATGCCGAGGCCTCTGTGAGGCCGTTGCCCTTTCCGGGCATTGTC
AATTGTGACGAGGTGCGGAGCACCGAGCTATCGCGTCTACGAGTCCGCAATGACTGTAAACTATTGCAACAGGGCGGTGTCACACCCAAATGTGTAT
AAACTTGCGCGTTTCGGAGCGTTCTCAAACGCTGGCCTACACTGGGTAAACGGATGGTGATCAGAACGGCGATGAGGTAACTGTGGACTAGTGACC
CTAGAAGTTATCCACCTCATTGTGAACCATCTTCCCTTTAAGATTTAGAAATGAGCACCGCTCTAAACGGTTAACACCATTTTGTTTTCGGCTTCCCTC
TGGACGGGACGATTCAAAATGTCTTCTATCAATTTTCTTCGTTTGGATCAACGCAAAGATCCCGGGAGGCTCCGAGTGCCTAAAGAACGGTCTGCTA
TCCGTTCATCCTATTCAAGTTCAGCCGGGACTTGGAGGCTCCGCAGGGAGTCGCTGCCGGCATGGGCCCAGCCCAAGTCTTCTCCTGTTGGTCTTCT
CGCTTGTGGTGAGCTGACCAGTACTTCTTCTTTCGGTGAGGTGGTTCAGTCCACGCCTCGCCCAGCCACCATGGCTGCCAAGGTGGACGTTTGTCA
AGTGAGGTACTTGACATTCTCCGACTCGTCTCAAGGAATAAAACACTGGTTGGTTAAAAGCCTGCCTGTCTCTCACCCTGATGTGTTAGAGATGAGG
TTACATGGCTTTGTCGATCGCCTGGCAACAGTGAGCTCTCAACATTCTGCTTGCAGAGTTGAGGCTGAAAGTGCGCTGTTTTCCCCATCCCGGACTT
CTGGTCACATGCAGAAGTTGATGGATGGAGTGTTGGCCCTTCGTCGCTCTGGTCAAATGACCTGTGTCAAGAAGGAATCTCCGGCTGAGTTCGCGC
GCCGTAAGCGTGAACAGAAGTGGTCTTGGCTCATCAGGCGTCTGCAGAGGCGTTCAGTTGCTGAGCAAGCTGTGATCGCTGAACAGCGGTTGACA
AAGGCCCTGCACTTGCAGGCCAATACTTCTTCCTTCGTCCCCACTGGGCCGAAGGGTTTCAAAGGTCTGGGAGAGTTGGTACTCCCTACCCCTTCTG
CTGAAGAGATCGCTTTTGGTTCCTCCTTCCTAAATGGCAAACGTGTTCGTGTTTTGCCAAGGAGAGAGAAGAACACTCGGTTTTCCGCTTGGTGCCA
GTCAAATGCCGAACGTAAGAAGGCATTAAAACTAGCCGAGCTGGAACGTCAAAGATTCAGATTGTCTTCGTTGGTCGCTGATCCTCTTCGTGTGCTC
GGATGTCAAATTCCTGAGACGTTTAGTGGTGTCATTTCTTTCGCCCGTGGTCTTTTTGGCACTTCTTCACGTCGCTCTGTCGTCCGAAGGGATTTCAT
CCATCGGGCTAGACACGCTGAGAGGTTCTTCTTCTGTCCTGTCAATGGTTACATTATTGATATGGTCGGGAACAAGATAAGGGTGTCCAGAAATAGG
GCGGGATCTGGTGTCTCTTTTCGCATTGATGGTGGTCAGGTCGCCCACTGCGGCCTAAAGGACGAGTATCTTTGTGCCGTTCTTCAGTCCAGGCTAA
ATCTCATACCAAACCCTGGCGAGCCGGAGCTCCTGACTATCGAACTGTTTCGTTCGAGATACGAGGACTATTTCGGGAGGTTTTTGACTGAAATGGA
CGGAACCAACCTTGACTTGTGGTCTTTAATGACCATGGCTAAAAGGTGGAAAAGAGATTTTGGTTGCCCTGGCAACACTTGCATCTTGTCTTTTGCG
GGTGATTCGGTCTCGTTTGCTGTTGTTAACGAACAGGACGATCTGCCGCCGGCTTTGTGGTTTCGCCTTGATGGCAAAGATTTGTTCCACTGTGGCG
TTGACCAGGTCAAAAGACTGCAGTTCCAGAAGAACATCTGGAAAGCAGTTGCCAAGATGGTCATCCAGGACGGTAGCCCTGCTGAACAGCGGTTG
GCAGAGAAGGTCTCCTCCTCCGCACACGTGAGTGTCCCTTGCCTGAAACCTAACTTGTTCCACCAGTTAACAAACGGTTTCAGGGTCTGCGCCAAG
TTCTCCGGGCTCAGCGTTTTTGAGCGCGGGCTCGATTTACACGCCCTGATCCTCGGGTCGAAGGCGGCTTCATCACAACCAGACAGGAGAGGTTG
TTTTGGGACGTAGGGGCAAGCAGGTTGGTGTTCACACCAAAAGGCCTGGCAAAGATATTCTTCCGCAAGCACAATCAAGAAAAATGGAGGATAGCT

TTGGCTGCCGCGATTGGTTTGACCTATGATGAAGGTCTGCTTTTCTTTTCAAAGTTTCACAATGATAACACGAAACCAGATGTTGTGTTGGAGACTTT
GGGTTCGTCACCCTATCACAAGCCAGATAGGGAAACAGTGACAAGAGTGGCTTTTGTTGACGTGGAGAAACCTGTCGCACCCAACACTACGCCTCA
GTCTTCCACAATTGTTCCGCCTCCGCCGCCTCCACCGCCGGTGCAGGAAGTCAAGCAGAAGAGGAAGAAGACAGAGTTAGCTGATGGTGACACGT
GGCTTCTTGCTGAAGGTTCTGAGGGCAAGTCAAACAGAGCTCGTTTTAGATCTCTCTTCCCACATGATTGGTGGTTGAGGGTGTCTGAGAGGGTTAT
GCCTTTCATGAATCAGGATGCATCTCGTGACGTCGGGTACAGATCCAGATCTTGGTCTCAATCTTCCCTGGTTTCAAATCCTTCAGAGAAGTTTGATA
TAAACACGGTGAAGTACACACCTTCTAGTTCTTCCAAGGCATCAGGTTCAACGCCTTTGCGGGTTGATCCTGAAGCTTTCAAGAGACAGCTTCAGG
CTAAAGCTAAGAAAGTGAGTGCGACGTCGTCTTCCCGTCCCGATGCGAGGGAGGTGTATCTGAAGGGTGTTGGTGAGTTCAAACCGGACAAGGTTT
CTCACAGAAAAGCCCTTTCTGAACCTACTCCGTCGAGGAAACCTTCCTCTGAATGGATGTCTATCTCTCGAGCATCATCTGATTATCTGTTCGATGAT
GATCATGAAGACGCAAGAGATTCGACCAAATCAGACGTTGAGACTAACCCTGGACCTTCGATCAGGCATTTCCGTTCCAAGTCGACTTCAGATTTGG
AGGGTGAAACACCGTCTCTAAATTTTACACCACCTACCCCTGGTGATGTCTTAACTGTGCACCATCCGACTCCACCTCGTTTGAGGGTTAATCACAG
GATGAATAGACACACTGTGATTGTTCCTTCACCGGGTGAAAACACGTCGAATTGGAATGTTGGTTACTCGTCATTTGCGTGGCCTCCTACAGAGGAG
CATCTGGAACCTCGAGACTCCAGAACCGCGGATATAGAAGAAAATCCCGGGCCAAGTGAAACCCTTGACCCCACTTTGCCCCAGTTCCGGGGTCGA
AGTGGTTCACGGAGTTCAATCGCTGTTGCCACAACGGAGCGATCACCCTCTAGAACGCGTAGGTTCTTTGGAAAGATCGGCATTCACGGCAAAAGC
GCGACGGAGGAAGTTAAGGACATTTTTGGATTCAAAGAACATGACGAGGTGTATCCTTACACTGGCAAGAACTCGTCGTTCGTAGAGAAACATTAT
GAATTCCACTATGTCCTTTCTCATCTACCTAAGGACAAGAAATGGCGGTCGTCAACTTTCATTAGGTGGGTTGCCGCTGCTTCTCTGTCCGAGCTTCA
AAAATCACTTGACTGTGTAGTAAAGGCTTCACATCCTTTCAACACCGCGCTTGTGAGTAAGCTGCGTGATGAGACGAACTCCTTCTTTCAGAAGATC
TCCGATTCTGAGCCGATAGGTGGTACGACCGTATCTGCCTTAGCTCATACGTTCAAGACTCTGTACGCCTGCCCTAACAATGGGTGCGGTTACGCACT
AGAGATAAAACCAGAAGATGGAATGGTACTATTTCATTTGACTGAGTCTCCAGACGAAACCGTTAAAAACAGGTTGTGGTGGGTTTTGAATGGGAA
TGAGATTTTGCATGGAGGGGACAAAACCCCTGTTTCCCCAAGCACCGTCTCATTCGGTTGGCTAGCAAAGGAATATTCGACACATGAGGTGATCATC
GAGCCCAAACCGGACTCTATGCTATCACAAGAGAAACCAGGGCCGTTTGCAATGCCCTCGCTCTCTCAGTTTCAGAAATGTGTCGACAAGATTATCC
CTTCTGGTAGTCATTTTTCTCGTTCTAAAGTCAGTGAACTCAAAGATCACATCAACCAGTACCTGGAGTCTACAGATGTCAAGATAGTAGATCCGGTG
CTGGTCGCGGCGATCGCAATGAGTTTTAAGTCGAAATTCAATTGTCCAAAAGAAACCAGCTCTTTCGCAATTGATTTTAGGCTCGACCGAGGAATGG
TTGTTTTCTGTGCCACAGATTCCCCTGAAGATAAGAACAAGGGGAAACTTTACTGGTGCATCGGTGGTAGTGTTATCGTCCATGGCGGGTTGCTAGG
TATTGAAGATGGGAAGTCTTGTTCCCCATCATTAGATCACTATAGCAAGTTATGGACGATGCCACTTCCGAAATTAGGTTGGGTAGACAGATCTTTTG
ACGCTGAATACTTCACGAAGATCTCAACCTGGCAAAAGGCCCTCGATCGGTGTGAACCGATCGTTGTATTCTCTGAACACACATTGCAACGTATACT
CTACCAGTATTTTGATGGTGTGACAGACAAAGTTTTGTCCCCATCACATACTGCAATCGTTGCTTGTGTGGTGAAATCCTTGATGAAATGCCCGGAGA

ACGGGGTAGGGTATGCAGTACAGTTTGACAGTCATGGGGCTTCACTGTACGTGTGTCGGCAACCGAATTTCGCGGAGGTGAACCGGTTGTGGTACT
TTCATTGGGATTTCCCTTTCGTGCACGGGGGAAGAAAATCTTATGCTCCAGAGCCCAGGCGGTTCTTTTATACTGAGCATCTCAACATAGATTATTCCA
TACCCATGCATGAGGTTTTTGTTGGAAAGGATCTACCTAAATCCAATTCTTCTCATTTGACTGTCAGGAGTCAACAAGAATTGTATTTTGGTTTTCAGC
AACAAATTGATATGTATGATGCGCCAACGCCAGCCTTCATGCATTCAAGAGCACATAGCACAGCCGAGCAAGCCATACGTGACACCGTGCCCAAGAT
GAGAACTTCCCAGTTCAATTTTGAGCACATTGCTGCGATAGCCATGTGTTATAGATCCAGGATGCAATGTCCGTGTCCGGAGTCTGCTATGGCAATCG
ATTTTGACTTCGCCAATGAACGCATTGTATTCTTTTCGAACCTAGGCAGTGGGTATTTCCGCTGTCGGGGCCAAATGTGGTGGTGTGTAAACGGAGTT
GGTCTGATTCATACTGGATCAGTGTTTCACACCTACCACAACCAATCAAACCAATCTCGACCTAGTCCCCGAGTGTGTAGGTACTGGGACGAGATTG
TTTCTTTTCATTTGACCGAATTCTGCCAACCCACATTGGGTTATTGGACATCGGATTTTCACCTGCAACCTGACAAAGACGAACAGTTGCAAAGAGC
TCAGATTGCACTGGATGCACATGATGACCCTGTTGAGCCCTTCAGCAGGGACATCGCAGTGTTTCATGCATTTGAGGCAATCAGACAAACGGCCCAA
ACGGTCGATATAACTGTCATGAAGATTGAACATCTGATCATCATGGCGTGCATATATCGTTCGAAAATGGCGTGCCCTACAGCAGGCACTTCAATGATT
GTCCAGTTCGATTTGTCAAACCAAAGAGTGAGATTCGGCGTTGCAAGAGGAACCCATCCGGAGTTGGATGGCAGAATGTGGTGGTATCTGCACGAC
GATTGCATGATCCACACTGGTAGGCCGGAAAAGCCTCACTCCGGTGAGGTTGGAACTCCGTTTCCTGTCAGCAGTCGGTTTTCTTTTCGTGCAACTC
ATCGTCAGGCCAAGGTGATGGCTAACGATTTTGAAGAGAAGTGGAGAGAAGACTCTAAGTGTAGGGCACGGACATGGCACATTGAAGGTGATGATA
CACCGAAGCAAAAGCTTGCTCGGTCCCATCACCCTTTGTGGTGTCCGAATACGTTCGACGAACGGTTCGACGAGATCTTCGTTGCCGCGTACATTTG
GTTTTTCTTTCAACTCATTTGCACGTTCTACTTCTCGTTCTCTCGGCCTAATCTTCGGCGAAAACCTGACAAACACACGGAAGGAGGAGAGCATCCT
CTTGGAGATGAGGATTTTTCTGCATCATCCTCCTTACTCGTGTGTAAGTTAGGAACCAGAGGTGATCAAGTCCCTGTCGACTATTACGCGAACGTGGC
GGCACACTATGGTGTCCGCACACATTCTCACGTATACAAAGATGTGACGACAGATGATCTTGAAAAGATGAAAGTCGGAGACTTTTCATCTAGTTCC
GCTGGTTATTTGAATCTCGCGTACGCTTCTCAACTTGGTTACAAGAGAGTTTTCACCCCGCACGTGGAGGTGGAGAGATCTCAAGGTGTGAGTTACA
CCTTGTCACCAAGTTCAAAGTGGGTAGAAGCAACGAGATTCATCGCTGATTGGTCGAGGGTTTCCTGGTCCAACTTTCTCCCCGCGTGGATAGCCAC
GAGGGTTACCGCTGTGTACGTACCAACTTTCAGAATTGGTGCGTTATCAGGAAGTGACATCCCGCGTTCTCATGACGGGTTGAACTTGCTGGAGAAA
GTTAAGAACACAGCAAAGTTCGATGAGGGTTGGACATCTGGCAGCTCATCAGTTTCAGTAATCCCTGACGAGATCAAGTTAAGATGCCCCGAAGTA
CCATCGGGTAACCATCTTGAAATATTAAAGGATTACAAAACGGTGCACATTCATGGTGGAGCCGGTACTGTGCAAACTGCCATTGCTTGCGGCGCAC
GACCGGTCATCCACGACAAGACACTCGATAGAGTGTATCATAGGATGCCTGGGCCAGAGGATTTCAAACAACCTTCTATTGGGAAGTTCATGGGTTG
GTTGGTATCTGCCGGTTTTAAAGTTAATGCACCAATAGAGGTAAGAGCGTGGTGGTTGTTACAATGGTATTGGTCGCAAAAGTGGAACTATGCGTAC
GACGCATTAGTTACACTTGCTAAAGCTTACACCATATCATCGTATGTGTACAATCATTGGGAACTTTTCCTCATTCTTTTCTTCACCGTTCCGGCAGTG
GTCTGGAGAATGTTGTTTTCTTCCAAATCATTGTTCTCCATAGTCAAAATTTGTGCGCTTGGTTTTTGGCGCTACCCTGTGTTGTGCCTGTTAGATTCG

AAAGTCACTTTCGTGGCAGGAATCTGGTCAATGCAAAACTTGTGGATGGGCGCGGCGCAAGATTTGTCTGCACTCATGGAGACACGATCTTCACTG
GTTTGGGAGCCAGTGCAAAAGACCGTAGGAACGAAGAAGTTGACGTTCCCTTTTCCCCTTGGGCATTGGAGCATGAAAGACAACCAAACTGGTAA
AATCTACGAAGGTAGATTTGTCGGTCCCTCCGAAACTATCGGAGGGAACTTCCGCCTTGTAAGCACTACTCGTGAATACAAACGTGGATTCAAGTCT
TTTCCTGCCCCTTTCAATGTTGGCCATGCAGAGTCTCTTGTGAAGGCTCAACACGTAGCACCATACTCGTCTTCACATAATTGTGTGACTATGATGGT
GCAACTTTGTGCCAAGAGATCAACTGCATGGACAGCATGTATGGTGATGGTGGGTGCCTTAACGGGACTTGCCCTGGCGCCGCCAAAACTGCTCAA
GGACATTCTTTCTTTTCTTTTCCCTGGCAAGGATTTTGCCAGTACTCCTTTTTATCGATCGCTTGGCTTTGCCGCGTCGTTAGAAAACATCCCGTTTGA
GATAGAAGGGATGGTCGAAACGACAGAGCCAATGCCAGAGACAGCTACACCTGAACCAACAGATCTGGCGGATCCAGATTCTTTCAATATGTTGGT
CGATGAAATAGCGTTCATCCAGGCATGCGTGGAACAACTCAAGATACCTACCTTGGACAAGGAGGCATTTGATGAGGTTTCACAGCGAACCCTAGA
AAAGGAGTTGGACAAGGTCGAAATCCCTGAGGATTCGTTAATCAGGGTTTCCAACATACCACCAAGAGTGCACCATGAGTGGGCGCAACTGGTGG
ATGGTCTACACCATGCTATTTCTTTCGTTCGCACAAATCGCGTCGCAGACGCGTTTGTGTTATACGTAAAGAATGCTGGTGAAAATATATATGACTTTG
TCCAGCCGATACTGGAAATCCTTTCCTTCTTCCTGCACCTTGCAGCTGAGCACTCCAAGCATGCATTCGATCGCTTGTTCAAATGGCTCTGCGATTTC
CTGGACCATGTCTGGGGTCTAGAAGCGTCAAAGAGAGTCAAAACAGTGTGGGGCCTGACTGGCCTTCATCGAACGGGATTGCTTGGGGTAAAAGC
CAAACTTGCAGCTAATGTTGCTTATTCTGATTTCGTAGGTAGACAGGATTTCGAATCAGATTACAACAAGCTGGTGCAAGAAGGCAAGCACCTTGCT
CAGGTGTATGGTGCCGTTAAACGATCAAACCTTGGAGGGCCTCAGAGGAGGCCTATAGGTTATGGAAAACCTCTAATGTCGCAATCTGAAGCCAGTC
TGCTCGGTTTCGAGCCAGACGAATTTGTGACTGATCGTGAGTATGAGGATCGTGTGCAATCGTACCTTGAACAGGGTGTTGCACAAGGTGCTGACG
GGGTGTTTTTGGCAGACAAGAGGCCGGAGCTGATTGCTAAATCACAGCACCGTTATGAACCAAAGTATCCCGCACTCACTTCAGAGGATCGTTCCAT
GGCCAAGGAAATAGCCCAGGCCATGTTTGATCAATATCCGGAAGTCTTCGCTAATTGTGATATTTTGCCACCAAGGGCAGTCCACAATTATGTGAAGC
AAAAATACTCTCCAGGAACCCCGTTCATCAAACCTGGGGGCTTCAAATCTCGTCAAGCGATGTTCGACGCTGGTTATGACCGCGTCATGCAACGACG
AGCAATGCATTACTTGAAAACTGGTGAATATCCAGTGCAGTTCTATCACGCTTTTGTGAAATCACAAGTGGTAGATATCAAGAAATGCTTAAGCCCCG
AGGAAGGTGGAAAAGGGAAAGATGTTCGCACTGTAGTCAGCCAGGATCTGTTCTCCTATTACATTGACCAATGCCTCCAAATTGAGCGCAATAAAC
GCATCAATTGGGATACGTATGGTGCAGGTATAGGTATGCCTCTTAATCAGTCGATGGAGGCGATATACAGTAGAATGGCAGCTGCACAGAAATCGAGA
GGAGGCAGGTACATCATTGCTGATGCAACCGCCTTCGACTCATTTTGCAAACCTTTCCTCTTTGAAGTAAATGCAAACCTTTGGGAACTCGGCTACA
AAGATCATCCAAGCGGTAACGGCAAGAACCTCGCATCTGTTCTAAATGCGTCGTATCAAGCCAGACAAAATGCATGGATTATCGGAGTGACCGAGCC
GGAATATGACTCGTTGACTGTTTGCGTCCAAGACAAAAACAGTCGCGAGCTCATACGGCAACGATCCCCGAAGACAGTAATCTGCTTGGAAGATCT
CATAGACTGGGACAAGTACAATAGATTGCCTGCTGAGCAACGTTCGAACTACGTTGCAAAACTTCAACCACCCGAAGGGAAGACGGTGGTAACCTG
GGATCCTTCTCTCCGCCCGTCCAGGTCAAACTGGATGGGTAAATATGAATATGGTGTCTTATCTGATGTTGACAAGAAGTTCTTTGAACATCAGACTT

TCACATACGATCCTTCAAATTTTGAAGACATGTATCAAGACATATTATCAGTTTCACGATCAAATTACAAACTGATGTCAAACGTACATGCGAAGAATC
GTGGTGGGAGCACTGGAGGGTCAGATACATCAAACGTTAACACACACGCCTTCAAGGCAGGTGTGATTTACGCTTGGTGTCAAACAACCGGCAGG
GCCCCAAAGGAGTTCTTCCAGTACAATGACCTTGCCAATACATCAGATGACACCATTTGGCAATCGGGTGGCAAACATGGCCTAAACACTGTTGAAG
ATGTCGAAGTGTTTCGACATCATTGTCAGTCAGTGGGAATTCATCTGGAGATAGAAACGACGAAGAACATATCACAAGTAGAATACTTGTCAAAATT
CGTTCGTGTTCCCACTGCAGAGGACTCAGAAGCATTGAAAGTCTGGAGATCCCAGAAAATCAAGGCTGCTTCAAATGCCCACAAACAACTAGGCCT
AACTGTTCCTACTTCATGGGAGCAGTTCAACAACCCGCAATTCGTCGTCGTTCAAAACCCTTCTGCGATCTTGCTCCGAAGATCTGCGTTTAGATATT
ATCAATCTAATGCAAACCATTGGAAGTATGTTTCAGTAGAAAGAGGTGCCGGTCATGCTCAAAGCACCGCCTTTCTACCTGAATTATATGGCAAGTTC
GCACAAGAGTGGTGCGACGACATCAATGATCTTCTGGTACAACAGAAGATCCATAGGAAGTTCAAGGTCACGAACGGCCAATTTGGACTACAGTGT
GTCGAACAGGTGGATCCGAGAGCGTCGCAACAACAGCTCTCACCACGTCAAGTCGCTTTCCTCTCATGGCTAAGGGGAAATATGTTCCCTAGTTACT
ACAGGGTACTTGACATCCACATGAACGTCGCAAAAGTAGATCCAGAACGACACGCCAAGTTCCTTCGTAAACTTGAGAAAGGTTGGCGAGGTTGG
GAAGAACTGTTACGTGAAGGAGTGGATAATCTCTACGCCTTAACTGATTCCATCCCAGATGAAATCAGCAAAAAGTTCCAACCCGGCCTCGACATGC
TCTATGCAGAGCAACCATTCTACACGAAGAACAAGGTCGTTGAAAAGTTTGTTTACTTGAAACTTCTGGAAGAAAGCCCAGAGACAGAGGTCACAT
TTTCAGATTTCTCTGCTCGAATTCAAGAATCACCGTATGCTTCGGTGTGCGACCCCTATCACTTCTGGGAGAAGTTAGGGGAGCCTGAATTCAAGGA
GCAGCTAATGGCTGAAGATCCTCTGAAGATACAAGGTCTCGTCTTGCTTATAAGTACTATGTACCTATTGACAAACACAGCTGAGTCAGTAGTATTGG
CCATTCCTTTTGTTGGCCTGTTCTACAAACTCTTCCTGTGGTCCTTCATCGGACTCAACAAGGTATATGGACTGTTGAACACTATGTATTGGCATTCCG
AGGGCAAGTCGTCGAGAGAAATATCTCGAATCATGCCCCGGGACCCATACATGGTAACAAAACAGTTTGCTGCTTTTTGCATCGACTTTGCACCATC
ACAGCTGGGTTACTTGGCCCTTATCCCGCTGCACATATTGTCCTTACTTCCAGTGTTGTTCGAAGCACTTGGAAAAGCATGGTATGTGGCAGGGGAG
GTCAAGAATGTGATGGAACCTCCTGGCAGCACCGTAAACAAGTGGGATCAATACGCAGATGAGTACATACAAGCTCTGCGTTCATCTCCCACAAAC
AGAAAATACGTTGCTGCCAAGACTTCAACTGGCAAATCGACCTGGTTTATAGCTGCCCTGTGGAAAGCTAGACACCGCCAGAAGGTCAGGAAATTT
TGGCTCGTCGAGCCTCGACTGGTATTACGAGACAACACGCATATACCATTCGACGCACCCTATCAAGTGCTCAAGGACAACATCCAAGTTTCAAATA
CCCACGATATCCACATCTTGACGTATGGACATTTACAGTCAAGATTGTTGGATATAGATAACGAACGTGATATCGTCATTTTTGACGAGTTTCACGAAC
AACAAGGTGAAATGATTCTTGGACTTGACAAGTGCAAGGCGCCAATCTTTCTTCTCTCGGCCACGCCAGCCGAAATACCATCTCTGCACGGTTCCGG
AACTCTAGTTCCAAACATTGACAGACGGCATCCAATCCAGATCCACCAAGTGGATGATGGAATGGGTGTTGTCGACATGTTTATGGAAGCCAGAAAC
CGTTACCCTGATTTATGCAAGAGAGCGATGATCATTGTGCCAACTCACAAGGAGGTTGCAAAGGTTATCGCTTCCCTTGATTATCTCAAGGTAGGTAA
GGTGTCCCCTCTGTCAGCACGCGACCGCGTGGTACAAGAAGAGGGAATATTGGTGTGCACCCCTTATGTCCAAACGGGTCTAGACATAAAACCACC
ACCAGATCTTTTGATCGATTGTGGCAAAGACTTCGTTTTGGATAAAGGGCGTCCAGTATTTCCACTCCCATGGACAGATAAGGACATAAACCAGCAG

AGGATTGGTCGTGTTGGAAGGTTAAGACCCGGATAATTCCTAATAAATACAATTTCAGC

> Cb_clean_k141_17023
GCCACGATCACGAAAAGACCTTTCGGTCATACCTTTTCAGGTATGGTTACTACGGCCGCTGTCCCCACTCCGTTGGATGGGACCGCGACGGCAACCA
GCGCGCTTAGAGCTGCGCGCAAAGAGTTGAAAACTCTCTGTGGTTTACTTGGGGAATCCTTTGGATTCTCCCTCGTTGTTCCACAGTTTTCTTCCCTA
AAAGCTCTCAAGGGTTCTTTGTCTGGGGCCCTTGACCCCTCTAGCGACCGCTCTTGGTTGCGGCCGGTTCTGAAGTTACCTCTTCGGGATCGGTTGT
CCGTCCTCGGGTCGATCTTCCTCTTTAGGAAGGTCCTTCCTGCCAGTACACCTGATCTTTCAGCCTACGCTGATAAGATGCAGGTTCCGG

> Cb_clean_k141_7251
TAGACAGTCCTTTTTGGGCTTCCAGTCAGTAGTTCAGTCGGGTGGTCCCCTTCCCAAGGGGGTGGGCGGTTGGCGCCTCCGTTATAAGGAGGTTCCT
TCCGCAGGGAAGTTGAGGGCTATGGGTATTCCAACTTACCGTTGGGATACCCTTGGGCCTTTGCACGAATGCCTCTATTCGTACCTTGGTGGTAAGGA
TTGGCTGCTTGTTGGTCCTCCAACAGCAGACAGGATCAGCTCGGTCTGTCAGTTTGACTGGCAGACTTCTGTTGATCTGGTAGGGGCGACTGACAA
CCTTCTGTTGGACGTCGCCGATACCATCCTTTGCGCTGCCCTCGCGCAGTGTGAGTTGGTTCCTGGTCGTGTGCGCCAGGACGCTGTGGATTCCCTT
CGTCCGACCGTTGGTGACTCTTCTGTCACTCGCGGCCAGATGATGGGCACTTACCTCTCTTTCCCCTTACTCTGCCTTCAGTCCTACGTCGCCGCCCG
CTGGGCCGTACGCGGGCGCGATGCCAGAGTGTTAATCAACGGTGACGACTGCCTCATAAGCTGTCGTACTCCCGTTCTCAATAGTGACTACCCCTCT
TGGGCAGTCATTAACGAGTCCAAAACCGGCCGTTTTCAGTCGGTAGCTGAGATTAACTCCACGTGTTTCCTTAGGGACTCACGTGGAAAGTGGAAG
GAGGTGAAGCACCTCAGGAGGGGTGGTGGTTCCCGTGACCTCCAGGGTCACATCCACCAGGCAGCTGTTTGTCGTGCTGCCGGTCCGTTGTGGGA
GCGTGCTTTCGTCCTTTCGAAACTTCGCTCGAGGTGGGTTCTACGTCCGAGTGACCTCGGCTTCGATCTCGGCGTACTTGAGACTTTCAAGTACGAG
CGGCGGCTTCACCGCCTCGGCTATGCGGTCTTGCCGCGCGCCACCGGTCTCGATGACAGTCGTTACAGGCTCAGCCTGTCCTCGACCAGCGTAGAA
AGGTTGGAGGTCTCTTTGGACCTTTGGGTGGGCGGTCGGTCTTTTCAGACCGCCAGGGATCCGCTTTCCTACAACGCTTTCAAGCGTTTGATAGTTA
AGCCTTCTAGAGCGTACCTTCGGGCTCGCTCTTTAGGTTGGCGTGGCGCCGAACTTACGTTCGGCGTTAGGTCACCGGTTCCCTGTCCTCGTCCGCG
TGGCGAGGTCGTCCTAGCGGAGTCTCGGCTGCCCTGCAGCCCTGGTCCCGTTTGTTTGGTGGAAGACGGGGTTCTTCTCGTCTGCGAACCTCCTACT
TCAGCTTACTGAGTAGGTTTCGCGCGGTGTGTCGTGCGCCGGCTGGCGCCCCCTACGGACTCTCTCCCCATACGGGCGTCCATAGTTGATCTCTCCTC
CGCAAGTGCGCGTGCTTGTCCCCTCGGGGGTACCTGTTTAAGACGGGTAAGCACGGCCGCCTTTGACAAGGCAGGGGGACCTCA

> Cb_clean_k141_17237

AGGAAGTTTCGCACGGACAGATGATGGGGAGCTACCTCTCCTTTCCCTTATTGTGCATTCACTCGTACCTCGCCGCGCTTTGGGCGCTCGGTGAGAG
AGAAGGCACAATCCTCGTTAACGGTGACGATACTCTTATTTCGTCGTCGTCTTACGTCGAAGCTTCGCATTACCCGTCGGGGTACCTTTTAAATGATG
CGAAGACTATTCGATCAAGAACGTGTGCAGAGATCAACTCGACAGCGTTCTTGAAGGACGGTAAGGGTAAGTGGCGTGAAATTCGCTCACTTAGGA
GAGGTAGTTTTCTTACCGATTATCGCGGGATGCTGCACGCTGCATCTGCTTGTCGCCGCGATGTCGCTTGGACCGATGCCTTCGTTCGGTCCCGGATC
GGTAAGAAGTGGGGGTTCCTCCCTTCCCAGTTGGGGTTACACCCCAGGTCCTACCCTGCTTTTTGCAGGGAACGATCAATGGAGAACAGGTACTTC
ACCTGTCTGCCGGAGGCTCCCAAAGAGCCTTCTCCATTACTTCGCGGGCTCCGCAGAGCCCTAGATCCTGACGAAAACCTTGCCACTGTTTTGCACC
AGTGGTCCTACGGTCGGGAGGGAGGGAGGAAGAGAGACGAATTTAACCCGTCTGTGGGTTTTGTTCGTCGGAGTTACGGGTACCGCAGCGTCAAG
CTGTGGTCCAAGCTTACTTTCTTGGGTAAGTTAGCCGGTTTGAAGTACCGGACCCGTGAAGAGAATAAGATGTGTTTTCTGCCTGCAGATTACACAT
CGATGCGAGAGGACGAAGTTCTCAGAGATCACCGCCGATTGACGTGTCTCGTTGAGGATGAGAACTTCAAGTAGAAAGGCCTCTTGGCCTAGGGCG
GCACCAGTGCCGTTGCGGGGGACATAGGAGAAGACATGCTGTTAGTGGCCAGCAGAGCCGTGACTGAACCCGTCCGGATAACCGGATTATTCCACG
CCTACGTTTTTAGTCAAGATTCGCTCTTGGACTCGATAGGTACCTCCTCAGTAATGAATGGTTTAAAGGGAAGTAAGGTAGGACCTTGAGTGCGTCCT
AATCCGGGGAAGGAAGTCGGCCGATGGCCCTTCGGGGTTGGGAGCTTAGAGACTATGTCTCGTAAGCCAGTTGAGGAATGCGCTCCGGCGCTACCG
GATACCCACACTAGCAGTAGAGTCCCTATGTTCAGGGCC

> Cb_clean_k141_36573
ACGCCACAGACAACCTGTCCCTTGTTGTGACAGAGGCGATACTGGGGACTTTACTCAGAAAGTCCACTCGTATCCCTGGCTCTGTTAAGCTTGTAGC
TTTCGAGTCACTTCGGCCTTTTGTTGATGGGCCGAATGGGATGCAGGAAGTTTCGCACGGGCAGATGATGGGGAGCTACCTCTCTTTTCCCTTATTGT
GCATTCACTCGTATCTCGCCGCGCTTTGGGCGCTCGGTGAGAGAGAAGGCACAATCCTCGTTAACGGTGACGATACTCTCATTTCGTCGTCGTCTTAC
GTCGAAGCTTCGCATTACCCGTCGGGGTACCTTTTAAATGATGCGAAGACTATTCGATCAAGAACGTGTGCAGAGATCAACTCGACAGCGTTCTTGA
AGGACGGTAAGGGTAAGTGGCGTGAGATTCGCTCACTTAGGAGAGGTAGTTTTCTTACCGATTATCGCGGGATGCTGCACGCTGCATCTGCTTGTCG
CCGCGATGTCGCTTGGACCGATGCCTTCATTCGGTCCCGGATCGGTAAGAAGTGGGGGTTCCTCCCTTCCCAGTTGGGGTTACACCCCAGGTCCTAC
CCTGCTTTTTGCAGGGAACGATCAATGGAGAACAGGTACTTCACCTGTCTGCCGGAGGCTCCCAAAGAGCCTTCTCCATTACTTCGCGGGCTCCGCA
GAGCCCTAGATCCTGACGAAAACCTTGCCACTGTTTTGCACCAGTGGTCCTACGGTCGGGAGGGAGGGAGGAAGAGAGACGAATTTAACCCGTCC
GTGGGTTTTGTTCGTCGGAGTTACGGGTACCGCAGCATCAAACTGTGGTCCAAGCTTACTTTCTTGGGTAAGTTAGCCGGTTTGAAGTACCGGACCC
GTGAAGAGAATAAGATGTGTTTTCTGCCTGCAGATTACACATCGATGCGAGAGGACGAAGTTCTCAGAGATCACCGCCGTTGGACGTGTCTCGTTG
AGGATGAGAACTTCAAGTAGAAAGGCCTCTTGGCCTAGGGCGGCACCAGTGCCGTTGCGGGGGACATAGGAGAAGACATGCTGTTAGTGGCCAGC

AGAGCCGTGACTGAACCCGTCCGGATCACCGGATTATTCCACGCCTACGTTTTTAGTCAAGATTCGCTCTTGGACTCGATAGGTACCTCCTCAGTAAT
GAATGGTTTAAAGGGAAGTAAGGTAGGACCTTGAGTGCGTCCTAATCCGGGGAAGGAAGTCGGCCGATGGCCCTTCGGGGTTGGGAGCTTAGAGA
CTATGTCTCGTAAGCCAGTTGAGGAATGCGCTCCGGCGCTACCGGATACCCACTCTAGCAGTAGAGTCCCTATGTTTAGGGCCCTAGGTTGCAGTGC
GCGTC

> Cb_clean_k141_50990
CTACGGAGGAGAAGATATCATCTGCCTGTGTTGGAAGTCACCAGACCTCAGTTGATCTGGTGAACGCCACAGACAACCTGTCCCTTGTCGTGACAG
AGGCGATACTGGGGACTTTACTCAGAAAGTCCACTCGTATCCCTGGCTCTGTTAAGCTTGTAGCTTACGAGTCACTTCGGCCTTTTGTTGATGGGCCG
AATGGGATGCAGGAAGTTTCGCACGGGCAGATGATGGGGAGCTACCTCTCCTTTCCCTTATTGTGCATTCACTCGTACCTCGCCGCGCTTTGGGCGCT
CGGTGAGAGAGAAGGCACAATCCTCGTTAACGGTGACGATACTCTCATTTCGTCGTCGTCTTACGTCGAAGCTTCGCATTACCCGTCGGGGTACCTT
TTAAATGATGCGAAGACTATTCGATCAAGAACGTGTGCAGAGATCAACTCGACAGCGTTCTTGAAGGACGGTAAGGGTAAGTGGCGTGAAATTCGC
TCACTTAGGAGAGGTAGTTTTCTTACCGATTATCGCGGGATGCTGCACGCTGCATCTGCTTGTCGCCGCGATGTCGCTTGGACCGATGCCTTCGTTCG
GTCCCGGATCGGTAAGAAGTGGGGGTTCCTCCCTTCCCAGTTGGGGTTACACCCCAGGTCCTACCCTGCTTTTTGCAGGGAACGATCAATGGAGAA
CAGGTACTTCACCTGTCTGCCGGAGGCTCCCAAAGAGCCTTCTCCATTACTTCGCGGGCTCCGCAGAGCCCTAGATCCTGACGAAAACCTTGCCACT
GTTTTGCACCAGTGGTCCTACGGTCGGGAGGGAGGGAGGAAGAGAGACGAATTTAACCCGTCCGTGGGTTTTGTTCGTCGGAGTTACGGGTACCGC
AGCGTCAAGCTGTGGTCCAAGCTTACTTTCTTGGGTAAGTTAGCCGGTTTGAAGTACCGGACCCGTGAAGAGAATAAGATGTGTTTTCTGCCTGCAG
ATTACACATCGATGCGAGAGGACGAAGTTCTCAGAGATCACCGCCGTTGGACGTGTCTCGTTGAGGATGAGAACTTCAAGTAGAAAGGCCTCTTGG
CCTAGGGCGGCACCAGTGCCGTTGCGGGGGACATAGGAGAAGACATGCTGTTAGTGGCCAGCAGAGCCGTGACTGAACCCGTCCGGATAACCGGA
TTATTCCACGCCTACGTTTCTAGTCAAGATTCGCTCTTGGACTCGATAGGTACCTCCTCAGTAATGAATGGTTTAAAGGGAAGTAAGGTAGGACCTTG
AGTGCGTCCTAATCCGGGGAAGGAAGTCGGCCGATGGCCCTTCGGGGTTGGGAGCTTAGAGACTATGTCTCGTAAGCCAGTTGAGGAATGCGCTCC
GGTGCTACCGGATACCCACACTAGCAGTAGAGTCCCTATGTTCAGGGCCCTAGGTTGCAGTGCGCGTCTA

> Cb_clean_contig_965
GCGGTAGACACGCACTGCAACCTAGAGCCCTGAACATAGGGACTCTACTGCTAGTGTGGGTATCCGGTAGCGCCGGAGCGCATTCCTCAACTGACTT
ATGGGACGTAGTCCCTAAGTTCCCAACCCCGAAGGGCCATCGGCCGACTTCCTTCCCCGGATCAGGACGCACTCAAGGTCCTGCCTTACTTCCCTTT
AAACCATTCATTACTGAGGAGGTACCTATCGAGTCCAAGAGCGAATCTTGACTAAAAACGTAGGCGTGGAATAATCCGGTGATCCGGACGGGTTCAG

TCACGGCTCTGCTGGCCACTAACAGCATGTCTTCTCCTATGTCCCCCGCGACGGCACTGGTGCCGCTCTAGGCCAAGAGGCCTTTCTACTTGAAGTT
CTCATCCTCAACGAGACACGTCCAACGGCGGTGATCTCTGAGAACTTTGTCTTCTCGCATCGATGTGTAATCTGCTGGCAGAAAACACATCTTATTCT
CTTCACGGGTCCGGTACTTCAAACCGGCTAACTTACCCAAGAAAGTAAGCTTGGACCACAGTTTGATGCTGCGGTACCCGTAACTCCGACGAACAA
AACCCACAGAAGGGTTAAACTCGTCTCTCTTTCTCCCTCCCTCCCGACCGTAGGACCACTGGTGCAAAACAGTGGCAAGGTTTTCGTCGGGATCTA
GGGCTCTGCGGAGCCCGCGAAGTAATGGAGAAGGCTCTTTGGGAGCCTCCGGCAGACAGGTGAAGTACCTGTTCTCCATTGATCGTTCCCTGCAGA
AGGCAGGGTAGGATCTGGGGTGTAACCCCAACTGGGAAGGGAGGAATCCCCATTTCTTTCCGATCCGGGACCGAACGAAGGCGTCGGTCCAAGCG
ACATCGCGGCGACAAGCCGATGCTGCGTGCAGCATCCCGCGATAATCGGTAAGAAAACTACCTCTCCTAAGTGAGCGAATTTCACGCCACTTACCCT
TACCGTCCTTCAAGAACGCTGTCGAGTTGATCTCTGCACACGTTCTTGATCGAATAGTCTTCGCGTCATTTAAAAGGTACCCCGACGGGTAATGCGA
AGCTTCGATGTATGATGATGACGAAATGAGAGTATCGTCACCGTTAACGAGGATTGTGCCTTCTCTCTCGCGGAGCGCCCAAAGCGCGGCGAGGTAC
GAGTGAATGCACAATAGGGGGAAGGAGAGGTAGCTCCCCATCATCTGCCCGTGCGAAACTTCCTGCATCCCATTCGGCCCATCAACAAAAGGCCGA
AGTGACTCGTAGGCTACAAGCTTAACAGAGCCGGGGATACGAGTGGACTTTCTGAGTAAAGTCCCCAGTATCGCCTCTGTCACGACAAGGGACAGG
TTGTCTGTGGCGTTCACCAGATCAACTGAGGTTTGGTGACTTCCAGCACAGGCAGATGATATCTTCTCCTCCGTAGGTGGTCCGACAAGGAGCCAGT
CAGTCTTCATTAAGTGACCATAAATGGTTTTATGAAGAGGCGCTAGCACTTCGGACCGCTCCTCATAAATGAGTAGCGGTCTTAACTTGCCGGCCGAC
AGGACCTCCTTATACCGGGCTAACATGGGTCTCGGAAGACCACGGGACCCATGTAAGCACTGCCTACGGAATTCTTTTCCTTTACCCTTCCAGTACTC
GTCGGCTCTTCGAGCCGTCTTCCTGGCAGTAGGGTTGGGGACATGACGCCAGACAAAATCGTCATACCGGTCATCCCAACCATATCGGAAAAGTTTA
GAGACTTCCTTGCGTACGAAGCGAAGGTAGTCTGGAGAAGGGGGAGGGGGGTTAGAGAATGCGTTCGCTTCCCAAGCGGAACGCACGGAGCTACT
GTGAAGGCGACAACCCGACGGCAGGTTTCGCTTAAGAGATGAGACGGAATGAGCAAATTCCCATCTCTCTCGCCTCCACAGTCTTTCAAGTCGACA
AAGACCGTCGACTCCCTGACGTTGCCGTCGAGGGAACCTAACAGATGCTCTCTCCTTGCCGCTTAAAAGGAGATACGAGAGGAACATTCCAAGTCT
TGATGGTTCAAGATCCGGTAACTCCGAGTTTGGCAAGCCAAACCGGATCCGAATAAGTCTTAAACCATTAGAAATGGCCTCTCTTGTGTCGCGCTCT
GTGCGTAGGCACAGACGACACGTTTGAGCACGGGAACCTGAGGAGGAGTTACCCCGTGCAGCGGTGGCAGTCGACGCTTGAAAGCTGCTACCTGA
ACTCGACATCGTAAGCCGGAAGGCGCGAGGGGTCGAGTGGAGTCCTATAACG

> Cb_clean_k141_30077
GTCCGTTATAGGACTCCACTCGACCCCTCGCGCCTTCCGGCTTACGATGTCGAGTTCAGGTAGCAGCTTTCAAGCGTCGACTGCCACCGCTGCACGG
GGTAACTCCTCCTCAGGTTCCCGTGCTCAAACGTGTCGTCTGTGCCTACGCACAGAGCGCGACACAAGAGAGGCCATTTCTAATGGTTTAAGACTTA
TTCGGATCCGGTTTGGCTTGCCAAACTCGGAGTTACCGGATCTTGAACCATCAAGACTTGGAATGTTCCTCTCGTATCTCCTTTTAAGCGGCAAGGA

GAGAGCATCTGTAAGATTCCCTCGACGGCAACGTCAGGGAGTCGACGGTCTTTGTCGACTTGAAAGACTGTGGAGGCGAGAGAGATGGGAATTTG
CTCATTCCGTCTCATCTCTTAAACGTAACCTGCCGTCGGGTTGCCGCCTTCACAGTGTCTCCGTGCGTTCCGCTTGGGAAGCGAACGCATTCTCTAAC
CCCCCTCCCCCTTCTCCAGACTACCTTCGTTTCGTACGTAAGGAAGTCTCTAAACTTTTCCGATATGGTTGGGATGACCGGTATGACGATTTTGTCTGG
CGTCATGTCCCCAACCCTACTGCCAGGAAGACGGCTCGCAGAGCCGACGAGTACTGGAAGGGTAAAGGAAAAGAATTCCGTAGGCAGTGCTTACA
TGGCTCTCGTGGTCTTCCGAGACCCATGTTAGCCCGGTATAAGGAGGTCCTGTCGGCCGGCAAGTTAAGACCGCTACTCATTTATGAGGAGCGGTCC
GAAGTGCTAGCGCCTCTTCATAAAACCATTTATGGTCACTTAATGAAGACTGACTGGCTCCTTGTCGGACCACCTACGGAGGAGAAGATATCATCTG
CCTGTGTTGGAAGTCACCAGACCTCAGTTGATCTGGTGAACGCCACAGACAACCTGTCCCTTGTTGTGACAGAGGCGATACTGGGGACTTTACTCA
GAAAGTCCACTCGTATCCCTGGCTCTGTTAAGCTTGTAGCTTTCGAGTCACTTCGGCCTTTTGTTGATGGGCCGAATGGGATGCAGGAAGTTTCGCA
CGGGCAGATGATGGGGAGCTACCTCTCCTTTCCCTTATTGTGCATTCACTCGTACCTCGCCGCGCTTTGGGCGCTCGGTGAGAGAGAAGGCACAATC
CTCGTTAACGGTGACGATACTCTCATTTCGTCGTCGTCTTACGTCGAAGCTTCGCATTACCCGTCGGGGTACCTTTTAAATGATGCGAAGACTATTCG
ATCAAGAACGTGTGCAGAGATCAACTCGACAGCGTTCTTGAAGGACGGTAAGGGTAAGTGGCGTGAAATTCGCTCACTTAGGAGAGGTAGTTTTCT
TACCGATTATCGCGGGATGCTGCACGCTGCATCTGCTTGTCGCCGCGATGTCGCTTGGACCGATGCCTTCATTCGGTCCCGGATCGGTAAGAAGTGGG
GGTTCCTCCCTTCCCAGTTGGGGTTACACCCCAGGTCCTACCCTGCTTTTTGCAGGGAACGATCAATGGAGAACAGGTACTTCACCTGTCTGCCGGA
GGCTCCCAAAGAGCCTTCTCCATTACTTCGCGGGCTCCGCAGAGCCCTAGATCCTGACGAAAACCTTGCCACTGTTTTGCACCAGTGGTCCTACGGT
CGGGAGGGAGGGAGGAAGAGAGACGAATTTAACCCGTCCGTGGGTTTTGTTCGTCGGAGTTACGGGTACCGCAGCGTCAAGCTGTGGTCCAAGCT
TACTTTCTTGGGTAAGTTAGCCGGTTTGAAGTACCGGACCCGTGAAGAGAATAAGATGTGTTTTCTGCCTGCAGATTACACATCGATGCGAGAGGAT
GAAGTTCTCAGAGATCACCGCCGTTGGACGTGTCTCGTTGAGGATGAGAACTTCAAGTAGAAAGGCCTCTTGGCCTAGGGCGGCACCAGTGCCGTT
GCGGGGGACATAGGAGAAGACATGCTGTTAGTGGCCAGCAGAGCCGTGACTGAACCCGTCCGGATAACCGGATTATTCCACGCCTACGTTTTTAGTC
AAGATTCGCTCTTGGACTCGATAGGTACCTCCTCAGTAATGAATGGTTTAAAGGGAAGTAAGGTAGGACCTTGAGTGCGTCCTAATCCGGGGAAGGA
AGTCGGCCGATGGCCCTTCGGGGTTGGGAGCTTAGAGACTATGTCTCGTAAGCCAGTTGAGGAATGCGCTCCGGCGCTACCGGATACCCACACTAG
CAGTAGAGTCCCTATGTTTAGGGCCCTAGGTTGCAGTGCGCGTCTACCGC

> Cb_clean_k141_57947
GTCCGTTATAGGACTCCACTCGACCCCTCGCGCCTTCCGGCTTACGATGTCGAGTTCAGGTAGCAGCTTTCAAGCGTCGACTGCCACCGCTGCACGG
GGTAACTCCTCCTCAGGTTCCCGTGCTCAAACGTGTCGTCTGTGCCTACGCACAGAGCGCGACACAAGAGAGGCCATTTCTAATGGTTTAAGACTTA
TTCGGATCCGGTTTGGCTTGCCAAACTCGGAGTTACCGGATCTTGAACCATCAAGACTTGGAATGTTCCTCTCGTATCTCCTTTTAAGCGGCAAGGA

GAGAGCATCTGTAAGATTCCCTCGACGGCAACGTCAGGGAGTCGACGGTCTTTGTCGACTTGAAAGACTGTGGAGGCGAGAGAGATGGGAATTTG
CTCATTCCGTCTCATCTCTTAAACGTAACCTGCCGTCGGGTTGCCGCCTTCACAGTGTCTCCGTGCGTTCCGCTTGGGAAGCGAACGCATTCTCTAAC
CCCCCTCCCCCTTCTCCAGACTACCTTCGTTTCGTACGTAAGGAAGTCTCTAAACTTTTCCGATATGGTTGGGATGACCGGTATGACGATTTTGTCTGG
CGTCATGTCCCCAACCCTACTGCCAGGAAGACGGCTCGCAGAGCCGACGAGTACTGGAAGGGTAAAGGAAAAGAATTCCGTAGGCAGTGCTTACA
TGGCTCTCGTGGTCTTCCGAGACCCATGTTAGCCCGGTATAAGGAGGTCCTGTCGGCCGGCAAGTTAAGACCGCTACTCATTTATGAGGAGCGGTCC
GAAGTGCTAGCGCCTCTTCATAAAACCATTTATGGTCACTTAATGAAGACTGACTGGCTCCTTGTCGGACCACCTACGGAGGAGAAGATATCATCTG
CCTGTGTTGGAAGTCACCAGACCTCAGTTGATCTGGTGAACGCCACAGACAACCTGTCCCTTGTTGTGACAGAGGCGATACTGGGGACTTTACTCA
GAAAGTCCACTCGTATCCCTGGCTCTGTTAAGCTTGTAGCTTTCGAGTCACTTCGGCCTTTTGTTGATGGGCCGAATGGGATGCAGGAAGTTTCGCA
CGGGCAGATGATGGGGAGCTACCTCTCCTTTCCCTTATTGTGCATTCACTCGTACCTCGCCGCGCTTTGGGCGCTCGGTGAGAGAGAAGGCACAATC
CTCGTTAACGGTGACGATACTCTCATTTCGTCGTCGTCTTACGTCGAAGCTTCGCATTACCCGTCGGGGTACCTTTTAAATGATGCGAAGACTATTCG
ATCAAGAACGTGTGCAGAGATCAACTCGACAGCGTTCTTGAAGGACGGTAAGGGTAAGTGGCGTGAAATTCGCTCACTTAGGAGAGGTAGTTTTCT
TACCGATTATCGCGGGATGCTGCACGCTGCATCTGCTTGTCGCCGCGATGTCGCTTGGACCGATGCCTTCATTCGGTCCCGGATCGGTAAGAAGTGGG
GGTTCCTCCCTTCCCAGTTGGGGTTACACCCCAGGTCCTACCCTGCTTTTTGCAGGGAACGATCAATGGAGAACAGGTACTTCACCTGTCTGCCGGA
GGCTCCCAAAGAGCCTTCTCCATTACTTCGCGGGCTCCGCAGAGCCCTAGATCCTGACGAAAACCTTGCCACTGTTTTGCACCAGTGGTCCTACGGT
CGGGAGGGAGGGAGGAAGAGAGACGAATTTAACCCGTCCGTGGGTTTTGTTCGTCGGAGTTACGGGTACCGCAGCGTCAAGCTGTGGTCCAAGCT
TACTTTCTTGGGTAAGTTAGCCGGTTTGAAGTACCGGACCCGTGAAGAGAATAAGATGTGTTTTCTGCCTGCAGATTACACATCGATGCGAGAGGAT
GAAGTTCTCAGAGATCACCGCCGTTGGACGTGTCTCGTTGAGGATGAGAACTTCAAGTAGAAAGGCCTCTTGGCCTAGGGCGGCACCAGTGCCGTT
GCGGGGGACATAGGAGAAGACATGCTGTTAGTGGCCAGCAGAGCCGTGACTGAACCCGTCCGGATAACCGGATTATTCCACGCCTACGTTTTTAGTC
AAGATTCGCTCTTGGACTCGATAGGTACCTCCTCAGTAATGAATGGTTTAAAGGGAAGTAAGGTAGGACCTTGAGTGCGTCCTAATCCGGGGAAGGA
AGTCGGCCGATGGCCCTTCGGGGTTGGGAGCTTAGAGACTATGTCTCGTAAGCCAGTTGAGGAATGCGCTCCGGCGCTACCGGATACCCACACTAG
CAGTAGAGTCC

> Cb_clean_k141_51878
GGTCCTGTCGGCCGGCAAGTTAAGACCGCTACTCATTTATGAGGAGCGGTCCGAAGTGCTAGCGCCTCTTCATAAAACCATTTATGGTCACTTAATGA
AGACTGACTGGCTCCTTGTCGGACCACCTACGGAGGAGAAGATATCATCCGCCTGTGTTGGAAGTCACCAGACCTCAGTTGATCTGGTGAACGCCA
CAGACAACCTGTCCCTTGTTGTGACAGAGGCGATACTGGGGACTTTACTCAGAAAGTCCACTCGTATCCCTGGCTCTGTTAAGCTTGTAGCTTACGA

GTCACTTCGGCCTTTTGTTGATGGGCCGAATGGGATGCAGGAAGTTTCGCACGGGCAGATGATGGGGAAGC

> Cb_clean_k141_18635
CTTCTCCTCCGTAGGTGGTCCGACAAGGAGCCAGTCAGTCTTCATTAAGTGACCATAAATGGTTTTATGAAGAGGCGCTAGCACTTCGGACCGCTCC
TCATAAATGAGTAGCGGTCTTAACTTGCCGGCCGACAGGACCTCCTTATACCGGGCTAACATGGGTCTCGGAAGACCACGAGAGCCATGTAAGCACT
GCCTACGGAATTCTTTTCCTTTACCCTTCCAGTACTCGTCGGCTCTGCGAGCCGTCTTCCTGGCAGTAGGGTTGGGGACATGACGCCAGACAAAATC
GTCATACCGGTCATCCCAACCCTCTCGGAAAAGTTTAGAGACTTCCT

> Cb_clean_k141_57307
TTTATGAAGAGGCGCTAGCACTTCGGACCGCTCCTCATAAATGAGTAGCGGTCTTAACTTGCCGGCCGACAGGACCTCCTTATACCGGGCTAACATG
GGTCTCGGAAGACCACGAGAGCCATGTAAGCACTGCCTACGGAATTCTTTTCCTTTACCCTTCCAGTACTCGTCGGCTCTGCGAGCCGTCTTCCTGG
CAGTAGGGTTGGGGACATGACGCCAGACAAAATCGTCATACCGGTCATCCCAACCGTATCGGAAAAGTTTAGAGACCTCCTTACGTACGAAACGAA
GGTAGTCTGGAGAAGGGGGAGGGGGGTTAGAGAATGCGTTCGCTTCCCAAGCGGAACGCACGGAGACACTGTGAAGGCGGCAACCCGACGGCAG
GTTACGTTTAAGAGATGAGACGGAATGAGCAAATTCCCATCTCTCTCGCCTCCACAGTCTTTCAAGTCGGCAAAGACCGTCGACTCCCTGACGTTGC
CGTCGAGGGAACTTTACAGATGCTCTCTCCTTGCCGCTTAAAAGGAGATACGAGAGGAACA

> Cb_clean_k141_5161
TGGAGATTGCTTGTCTTTCTCCAAGGCTGTGAAGGCGCTCCTTGCGGATTGCCCGTCCGACCTCGAAGAGGAACGGATGGCCTGGCAATCAACTAA
GAAGCTCCTGCCTGCTTCGTGCAAGTGCATGGAACTTCCTTTGTTACAAGGGGTGGTCAATGGTTTCGCGCGCCCTGCGCGAAGCCTTCCTTCTGGT
TACCTTCAATTCGTTGAGAAGGAGACCCGCCGCCTTTTTCCTAAGGCGTGGGATTCCGGTCTTTACGAAGAGAGGGTTCTTTCTACTTCTCCCGGCC
TTTCTGGCACCGTTGACAACGTCCGTTCAGACGGCGGTTGTCATGCCGATTGGAAAGGCAAGCATTCCGACTTCCTGGATGGCGCCCTTAGGGGCTA
CTATCCGGGAGGTATCGAACGTTGTGCGGAGCTTATGGTAGTTCAATCTGCTGGTAAACCTCGTCCTCTGACGAAGTTTTCCGGGGAAACACTACTC
CTCAAGCCTTTGCACACATCGATCTACGATCGGTTACGGAGCCAACTTTGGCTTTCCGTGGGCGACGTATCGGATTCTTCTCTTGCTAGAGCCGGGTT
CAGCTCGAAGGATGGCGATGTCCTCACTTCTGGCGACTATAAGTCGGCAACTGACCAGTTGTCGATAGAAGTCGCAGAGAGGATCCTTGGCACCATT
CTCGCCAATGCGATCTGTGTCCCCCCTGGTCTTCGCGAGGAAGCCTTAAAGATCCTTCGGCCAATTCTCTTTCATGAGAAGTTGGTTCCGAATGGTAT
CGAGCCGCGAGTTGGACAGATGATGGGTAGCTTTCTTAGCTTCCCACTTCTCTGTCTCCAAAACCGCTTCGCCTTTTTGTGGGCGATGCGAGTTGGA

GGCTTAAGTCCTGCAGCTGCGGAAAAGGTTCCTTGCTTGATCAACGGGGACGACATCCTGTTTCGTTCAACGCCTGCGTTGAGCGAAGTTTGGATG
AAGACGGTTGGCGAGCTTGGTTTAGAGGTCGAGCGTACTAAGACGTCTGTCTCCCCCAGTTTCGGTTCTCTGAACTCGACACTGTTACGTTGGAAA
GGGGTTCACCTTCGGGTGATCCCTACCCTTCGTTTCGGGAGACTACGATCTTCGCAGTACGTGAACTCGTTGTCACGTGAGTTTCGACAGTTTGTCG
CAGGGTTTAAGTCGAATCAGCGCTTCCGCGCTGGCGTCGTCTTTTTCCGCTGGCACCTTTCTGCTCTTAGGTCAACTAGATTGACTCTGTTAGAGCTC
GGGTTTCGCGGAAGCCTTGCTTGCAGACTTTCGATTCTCTTTCGGTTGGTCCCGGAGGAGCAGCCACGGTTTGAGGTTCCACCAGCCCCAGTCGGT
CACAATGTGGTCGTTTCTAACGACCTTGCCGACTGGGTCCCCGAGGAATCTCTTTCCCATGAGCTCTCCGTTCTCAACGCGAGGGAGACCGCTTCTT
GGAAGTTCGGACTGAACTTTCAGAACGACAGGGACAGGGCTACCATTCGGTACTTCCTGAAGCTGTCTTCTGTCAGACGTCCTTGCTTTTCCTTTTC
GGTCCGGGAGGGGTGGAGGAGTAGGTTGACTCCGGAAACAAGTGGCGAGCGTCTCAGACGTCTCAAGGCTTGGTTTTCGCAGCCTTTAGAAAAGA
AGGAAAAACGGTTGGCTCTTTTTCGGATTCTCATAGATTGGACCCAGTCCGATTCTTATGATTGTCCACCGAGCTACTCAGAGACCGTGGGGGGGG
TTTGGGGTCGCGCGAGCTTGTCTCGCCGCAGGACCCGCTTAAGAAGTGAACGGACACGCGGCCTTCGGGCCGGGCTGTAACGGCCCAGCAATCCA
AAACGGTCCGCCGGGATAGAACTACTCACTGAAAGAGTGACAAAGTTTTCCGGTGTGTCAGAAGGGGCATTCCCCGAACCGTAATTGGGAGAACT
CCCGGACGAGGTAGCGCTAGACGAACGGTAACGCGTGTGGGCAGAAATGCACATGCGTAGTCCCTAGTAGTGGCAATACCCTTAGGAGATCTTTATG
AGCGAACGTGGTGAGGCGGCTTAAAATCCGCGGCCTGAAAAGTCAATATACAGGTGCTCTTCTGAGTGGAAAGAAGGA

> Cb_clean_k141_3984
TAATAAGTCTCGAACATGTCGAGCAAATTAAGTATCCAGTTTCCCTCATTGAAGAGGAAAATAATGGATGAGAGGGTCTGGTCCGAGATGACCAGTG
TCCCTCTTTGGTACAACCCGACCGCAGATAGCGTTCGAGTTGTCCCGGATGCTCTCCTACCATACATGGAGGGGAAGCATTTCCTTTCGACAAAACA
AGTGCCGTCGGCACTTATTTTGGCGAAGGCCGGTTCGTTGACAGTGTCAGCGAACCGGTCCAATCTGTTCAAAAGAGGATTCTCTGTTGAACAGGT
TGACTGGGATAATAATGGTGAATCCAAGATTATCTCAGTAGCCGGGCACGAAGGTGTAGCACCTTCGTACTCGGCGTCGCTGTCCTCTTTTCTTAAAG
AGTCCAGCGGCGTGATGGAGGCAGTTAGTGAAAACGCTGACTCCATCGACGAGGAAGCAGAACATTCCGGTTCTGAAACCTCGGACGTAGAAGGG
TTACTGCAAGGCAGTAGCTCTACTACGATTATTCGGAAAAGAAATCGACTTTCTCACCGAATAAAGTACGAATGTCCATGGAAACTCCATGCGGCAT
GCGCACTCGCTACTCTCAGATCCGAACCCGGACCTGAGGTAGTCGTCTGGTCTGGAGACGGCATCCGTCTCCAAGACCCGCTCCCACCTAGGTTGTT
TGGACCGAAGTGGGATGGATCTCTGCGAAACAAGTTAAGGTTTCACAAGATCGCGGATGGTAACGTGAAATCTCACGTTATCTACCGACACACTCA
CTGGGGACGACGTCTCCAGCGAGAGTGCGCAGACCCAGAGTCTAAAATTAGATACTGGGCCTGTACCCTCCGCAGCCGAATTAATCGGTTTTTGGC
GGGTAAATCCGATCCCATCTGGACTTCAGATGAGAAAAGGATTCTGTCGCGGGACGGGTCTTATCAGACCCGTGACCGTGCAGCACGGTCGTTAAG
GTTGCTTGAACTCTTAAAGACCGTGGACGGGGTATTCACACAGAGATATCTGGCGAACCCCGCTGAAGTGTGGACATGGGACAGATACGACCTGTT

CACACTTGGAACTCTATCCCTATTAATAGGAGACGAGTTCTTAGACGGCGAACTACCTCTAGAGGTATTACCCGTCAGCACTTCCTATTCCACACTTA
AGTGGACTAGAAAGTGGTTCAAGAAGGTGTCTCATAGAGATCTTCTGGAACGTGAGACCAAGCCCCCGCCCGAAGGGCAGGAATGGTCTCGGCTG
CTTTGGAGAACATGGAAGGTTCTTCAATCAGCCTCGGGATACGAGCGCCTATTGATAATAGGCATTCTATCTCAGACTAGGGGTTGCGGTACACCGCC
ACCACTGGTCGTACTCCAATCGAAACGGAAATTCCTCGAGACCGTTTCGCTGGAGCCCCCCGAAGAGAGCGCCACAGTGCGCTCCCTCCGGAGGAT
GGCAGTGCAGGAGGTTTTGGAAAACCTCCCGCCTGTCGCCGTGACAGGACTCGCGACCAAGTCGCGTGTAACTGTCACGTCCGCCGCTTGCTGGG
AAAAGACCCGGAAAGAAGGCGGAACGACGGAGCAGATCAAGCAAATGATCTGCGACCTCGATCCTATGTCCCAAGTTCCCGTGAGGAACCTGGAC
ACAGGTAGGGTCGAACGCTGGAAGTTCCAGGACGAGTTCGACTCTGTTGGAGAACTTATATTCTGGGTCAGTCTTGACCGAGTTCTCCACACACCA
CCAGTGGAGCTTCGCAAAGCTTTTCTCACTGTGGTGAAGGAGCCTGGTAAAGCTAGAAGCGTTACCAAGGCCCGTGCTTGCCTCAAGATCGTGCTC
GATCTTGTTAGCAAGTTATGCTCTGAGCCCTTTATAAAAGGAATCAGAAGCAGCCAGTCTGGAATGAGCGCATCAAACCACGGCTGGAACTTTTTCA
ACTCATTCTCGAATGAGATTGAAAGAGAAGAGACGTTTTCTCTTTTATCAAGAGAAGAACGCTCGTTCGAAGGGTATGTTGAACGAACAGATACCTT
CGAAGACCTCTATGTGTCCTCCACGGACTACAGTGAGGCTACCGACCGGCTCCAACACCAAGTTGGAATCGATCTGGGAGTGCCTTTGATGACCAA
ATGTGGTATACCAAAGGTACTGCAGGGTATTGTAGTAGAAACTTGCTACAATACCAGGCAGATCTACTTCAAGGCCACTGGCCTTTTGGAAGATCTG
GGTGAGCGTACGGATTCCGAAGGAATCCACGTAATCACCCTTCGTCAAGGTGTCCTCATGGGGGACCCCTTGACGAAACCGGTCCTACACCTGATTA
ATGTGTGTGACCGGCTCCTGCAAAAGCGGCTCCTAGAGCCCGACTTTTACGGCAGGTTGGGAAATTTCCATGAAATTTCCCAACTTCTCTCACGCGT
TAAGAGTAAACTTAACGAGTGAGATCTATCCGTGGTTGAGATTTTAGACTCACCTGCGGGTAACGTATAGCCCCTAACTGGGGAGCATTTACGTTACG
TTAACTTAATAAGTCTTG

> Cb_clean_k141_57522
TCCCCTCCCATACCTGGAGGGGAAGCATTTCCTTTCGACAACACCAGCGCCGTCGCCAATTATTTGGCGACGGCCGGTTCGTTTACAGGGTCAGCG
AACCGGTCCAATCTGTTCAAAAGAGGATTCTCTGTTGAGCAGGTTGACTGGGATAATAATGGTGAATCCAAGATTATCTCAGTAGCCGGGCACGAAG
GTGTAGCACCTTCGTACTCGGCGTCGCTGTCCTCTTTTCTTAAAGAGTCCAGCGGCGTGATGGAGGCAGTTAGTGAAAACGCTGACTCCATCGACGA
GGAAGCAGAACATTCCGGTTCTGAAACCTCGGACGTAGAAGGGTTACTGCAAGGCAGTAGCTCTACTACGATTATTCGGAAAAGAAATCGACTTTC
TCACCGAATAAAGTACGAATGTCCATGGAAACTCCATGCGGCATGCGCACTCGCTACTCTCAGATCCGAGCCCGGGCCTGAGGTAGTCGTCTGGTCT
GGAGACGGCATCCGTCTCCAAGACCCGCTCCCACCTAGGTTGTTTGGACCAAAGTGGGATGGATCTCTGCGAAACAAATTAAGGTTTCACAAGATC
GCGGATGGTAACGTGAAGTCTCACGTTATCTACCGACACACTCACTGGGGACGACGTCTCCAGCGAGAGTGCGCAGACCCAGAATCTAAAATTAGA
TACTGGGCCTGTACCCTCCGTAGCCGAATTAATCGGTTTTTGGCGGGTAAATCCGATCCCATCTGGACTTCAGATGAGAAAAGGATTCTGTCGCGGG

ACGGGTCTTACCAGACCCGTGACCGTGCAGCACGGTCGTTAAGGTTGCTTGAACTCTTAAAGACCGTGGACGGGGTATTCACACAGAGATATCTGG
CGAACCCCGCTGAAGTGTGGACATGGGACAGATACGACCTGTTCACACTTGGAACTCTATCCCTATTAATAGGAGACGAGTTCTTAGACGGCGAACT
ACCTCAAGAGGTATTACCCGTCAGCACTTCCTACTCCACACTTAAGTGGACTAGAAAGTGGTTCAAGAAGGTGTCTCATAGAGATCTTCTGGAACGT
GAGACCAAACCCCCGCCCGAAGGGCAGGAATGGTCTCGGCTGCTTTGGAGAACGTGGAAAGTTCTTCAATCAGCCTCGGGATACGAGCGCCTATTG
ATAATAGGCATTCTATCTCAGACCAGGGGTTGCGGTACACCGCCACCACTGGTCGTACTCCAATCGAAACGGAAATTCCTCGAGACCGTTTCGTTGG
AGCCCCCCGAAGAGAGCGCCACAGTGCGCTCCCTCCGGAGGATGGCAGTGCAGGAGGTTTTAGAAAACCTCCCGCCTGTCGCCGTGACAGGACTC
GCGACCAAGTCGCGTGTAACTGTCACGTCCGCCGCTTGCTGGGAAAAGACCCGGAAAGAAGGCGGAACGACGGAGCAGATCAAGCAAATGATCT
GCGACCTCGATCCTATGTCCCAAGTTCCCGTGAGGAACCTGGACACAGGTAGGGTCGAACGCTGGAAATTCCAGGACGAGTTCGACTCTGTTGGAG
AACTTATATTCTGGGTCAGTCTTGACCGAGTTCTCCACACACCACCAGTGGAGCTTCGCAAAGCTTTCCTCACTGTGGTGAAGGAGCCTGGTAAAGC
TAGAAGCGTTACCAAGGCCCGTGCTTGCCTCAAGATCGTTCTCGATCTTGTTAGCAAGTTATGCTCTGAGCCCTTCATTAAAGGAATCAGAAGCAGC
CAGTCTGGAATGAGCGCATCAAACCACGGCTGGAACTTTTTCAACTCATTCTCAAATGAGATTGAAAGAGAAGAGACGTTTTCTCTTCTGTCAAGA
GAAGAACGCTCGTTCGAAGGGTATGTTGAACGAACAGATACCTTCGAAGACCTCTATGTGTCCTCCACGGACTACAGTGAGGCTACCGACCGGCTC
CAACACCAAGTTGGAATCGATCTGGGAGTGCCTTTGATGACCAAATGTGGTATACCAAAGGTACTGCAGGGTATTGTAGTAGAAACTTGCTACAATA
CCAGGCAGATCTACTTCAAGGCCACTGGCCTTTTGGAAGATCTGGGTGAGCGTACGGATTCCGAAGGAATCCACGTAATCACCCTTCGTCAAGGTGT
CCTCATGGGGGACCCCTTAACGAAACCGGTCCTACACTTGATTAATGTGTGTGACCGGCTCCTGCAAAAGCGGCTCCTAGAGCCCGACTTTTACGGC
AGGTTGGGGAATTTCCATGAAATTTCCCAACTTCTCTCACGCGTTAAGAGTAAACTTAACGAGTGAGATCTATCCGTGGTTGAGATTTTAGACTCACC
TGCGGGTA

> Cb_clean_k141_40198
TCGTTCACCATCGCTTTGGTGACGACTTAGACGTCGATCTTGCCACCCTACGACCACTCCTGCATCTCTCGCTGTTGCGTCTGCGCGACATATCCCAT
CTGCGGCCGCGACCCAGGAAACAATACCGAAACGGCCTCATCTCCGCATCAGCAGCCAAGGAGCCTACACCCGCATACCATGTTATAGCCGTGTGG
ACGTCTTCCCAGCCCTGTCAACACACCCATCTGCGTCGAGCGACTTCTACCATCCTGCGCTAAGCGCCTGCGCCGCCACTCTGGCCGCTCAGTCGCG
CGCCGCTGCTGTGCGCCTCGCAACTCAAAGCACCACCGCCGGCGACAGCCATGTCGTCGACCACCTCGAGCGCTCCGGCGTCGACCACCACCGGC
ACCGACACCGGTAAAGGCGGACCCACGAGCTCGCCGCTGTTGTTCTTCGTCGCCCTCGGATTCGGTGTAGTCTTTACGAACCTATGGATCATCGTCG
GCGTAAAATACTGCTTCAGATATAACCAACGCAATCGTGCAGCGCGTGCCTTGAACGAAAATGGCGAACCCATTGACCTTGCGCAAATGCCGCACCC
GAGACGGAGGAGGCGCGAGAAGAAGCTCATGTCCATGGAAGATGTGAACGAGCGCTTCCCATTGATCAAGTACAAGACATGGCGATCATCGCGCG

AAGCCCAAGGCCTTCCTGCAGAGGGTGGAGTGACCGCACCGACCAGCAGAGCTGGGAGTATGAGAGACGAAGAGGGTACGATTGAAGCGTTTGG
CAAGAACTCCGTCGATACCGCACGGCCTGGAACGGCGCTCAGCATCGCACAGAAGGATCACGAAAATGCGCTTGGGGACCCACCGAAAGCGCAAG
CTACTGCCAGCGGAGAGAAGGGGCCGGAAATGGTTACCATCGAAGATATGGAGAAAGCCAGGAAGAGGAGCATTGATCGAAACAACTCGGCTGTC
ACAGATTTGGACGACGACGAGGACGACCCAATAGCAACGGCAGCGCCACCTGAGATGCTTGCTGCACCTGGTGACACATGCGCCATCTGCCTGGAT
AACCTCGATGATGACGACGATGTGCGAGGGCTCACATGCGGGCACGCATTTCACGGATCCTGCGTCGACCCATGGCTCACAAGTAGGAGGGCTTGC
TGTCCACTTTGTAAAGCCGACTACTATGTGCCCAAGCCCAGGCCGGATGCAGACGGGGCCAATGCAGCCGCAGCTCGCAACGGCCGGTTGCCTCAA
GAGCCACCTCCAGCACGACGACACGGTCTGCCATTTGCACCGCGCGTAATGCTCTTTGCCACACCACGCTTCTTTGTCAGCGACGGAACACGCGCG
GAGCAGCCGAATCCAGCAGCTGCAGAACGTGCACGGCGAGAGCGGGACGTTTATCAGGATCCCGCCATTCAAGCAGAACCCACATCGCGAAGCTG
GAGGTGAGGATGTTGAGGTGTTCCGCAAGCTGACCGGGACCGTCATATCTCCAGCCATTGACAGCAATCTTGCCTTTGCCGTCCACATTTCTTATGTC
GATGGTGACAATTTCGTCCGAGGTAGAATTGAATGGCCCGAGCAAACCCTGCGTAATGAACTTCACTTTGGTTTCGAGGGGAGCAGGGCCGGGTGG
CAGTGATTTGGGATCAACTGGTTGAACATGTCGAGCAAATTAAGTATCCAGTTTCCCTCATTGAAGAGGAAAATAATGGATGAGAGGGTCTGGTCCG
AGATGACCAGTGTCCCTCTTTGGTACAACCCGACCGCAGATAGCGTTCGAGTTGTCCCGGATGCTCTCCTACCATACATGGAGGGGAAGCATTTCCT
TTCGACAAAACAAGTGCCGTCGGCACTTATTTTGGCGAAGGCCGGTTCGTTGACAGTGTCAGCGAACCGGTCCAATCTGTTCAAAAGAGGATTCTC
TGTTGAACAGGTTGACTGGGATAATAATGGTGAATCCAAGATTATCTCAGTAGCCGGGCACGAAGGTGTAGCACCTTCGTACTCGGCGTCGCTGTCC
TCTTTTCTTAAAGAGTCCAGCGGCGTGATGGAGGCAGTTAGTGAAAACGCTGACTCCATCGACGAGGAAGCAGAACATTCCGGTTCTGAAACCTCG
GACGTAGAAGGGTTACTGCAAGGCAGTAGCTCTACTACGATTATTCGGAAAAGAAATCGACTTTCTCACCGAATAAAGTACGAATGTCCATGGAAAC
TCCATGCGGCATGCGCACTCGCTACTCTCAGATCCGAGCCCGGACCTGAGGTAGTCGTCTGGTCTGGAGACGGCATCCGTCTCCAAGACCCGCTCCC
ACCTAGGTTGTTTGGACCGAAGTGGGATGGATCTCTGCGAAACAAGTTAAGGTTTCACAAGATCGCGGATGGTAACGTGAAATCTCACGTTATCTAC
CGACACACTCACTGGGGACGACGTCTCCAGCGAGAGTGCGCAGACCCAGAGTCTAAAATTAGATACTGGGCCTGTACCCTCCGCAGCCGAATTAAT
CGGTTTTTGGCGGGTAAATCCGATCCCATCTGGACTTCAGATGAGAAAAGGATTCTGTCGCGGGACGGGTCTTATCAGACCCGTGACCGTGCAGCAC
GGTCATTAAGGTTGCTTGAACTCTTAAAGACCGTGGACGGGGTATTCACACAGAGATATCTGGCGAACCCCGCTGAAGTGTGGACATGGGACAGAT
ACGACCTGTTCACACTTGGAACTCTATCCCTATTAATAGGAGACGAGTTCTTAGACGGCGAACTACCTCAAGAGGTATTACCCGTCAGCACTTCCTAT
TCCACACTTAAGTGGACTAGAAAGTGGTTCAAGAAGGTGTCTCATAGAGATCTTCTGGAACGTGAGACCAAGCCCCCGCCCGAAGGGCAGGAATG
GTCTCGGCTGCTTTGGAGAACATGGAAGGTTCTTCAATCAGCCTCGGGATACGAGCGCCTATTGATAATAGGCATTCTATCTCAGACTAGGGGTTGCG
GTACACCGCCACCACTGGTCGTACTCCAATCGAAACGGAAATTCCTCGAGACCGTTTCGCTGGAGCCCCCCGAAGAGAGCGCCACAGTGCGCTCCC
TCCGGAGGATGGCAGTGCAGGAGGTTTTGGAAAACCTCCCGCCTGTCGCCGTGACAGGACTCGCGACAAAGTCGCGTGTAACTGTCACGTCCGCC

GCTTGCTGGGAAAAGACCCGGAAAGAAGGCGGAACGACGGAGCAGATCAAGCAAATGATCTGCGACCTCGATCCTATGTCCCAAGTTCCCGTGAG
GAACCTGGACACAGGTAGGGTCGAACGCTGGAAGTTCCAGGACGAGTTCGACTCTGTTGGAGAACTTATATTCTGGGTCAGTCTTGACCGAGTTCT
CCACACACCACCAGTGGAGCTTCGCAAAGCTTTTCTCACTGTGGTGAAGGAGCCTGGTAAAGCTAGAAGCGTTACCAAGGCCCGTGCTTGCCTCAA
GATCGTGCTCGATCTTGTTAGCAAGTTATGCTCTGAGCCCTTTATAAAAGGAATCAGAAGCAGCCAGTCTGGAATGAGCGCATCAAACCACGGCTGG
AACTTTTTCAACTCATTCTCAAATGAGATTGAAAGAGAAGAGACGTTTTCTCTTTTATCAAGAGAAGAACGCTCGTTCGAAGGGTATGTTGAACGAA
CAGATACCTTCGAAGACCTCTATGTGTCCTCCACGGACTACAGTGAGGCTACCGACCGGCTCCAACATCAAGTTGGAATCGATCTGGGAGTGCCTTT
GATGACCAAATGTGGTATACCAAAGGTACTGCAGGGTATTGTAGTAGGAACTTGCTACAATACCAGGCAGATCTACTTCAAGGCTACTGGCCTTTTGG
AAGATCTGGGTGAGCGTACGGATTCTGAAGGAATCCACGTAATCACCCTTCGTCAAGGTGTCCTCATGGGGGACCCCTTGACGAAACCGGTCCTAC
ACCTGATTAATGTGTGTGACCGGCTCCTGCAAAAGCGGCTCCTAGAGCCCGACTTTTACGGCAGGTTGGGAAATTTCCATGAAATTTCCCAACTTCT
CTCACGCGTTAAGAGTAAACTTAACGAGTGAGATCTATCCGTGGTTGAGATTTTAGACTCACCTGCGGGTAACGTATAGCC

> Cb_clean_k141_72534
TAAGTCTCGAACATGTCGAGCAAATTAAGTATCCAGTTTCCCTCATTGAAGAGGAAAATAATGGATGAGAGGGTCTGGTCCGAGATGACCAGTGTCC
CTCTCTGGTACAACCCGACCGCAGATAGCGTTCGAGTTGTCCCGGATGCTCTCCTACCATACATGGAGGGGAAGCATTTCCTTTCGACAAAACAAGT
GCCGTCGGCACTTATTTGGCGAAGGCCGGTTCGTTGACAGTGTCAGCGAACCGGTCCAATCTGTTCAAAAGAGGATTCTCTGTTGAACAGGTTGA
CTGGGATAATAATGGTGAATCCAAGATTATCTCAGTAGCCGGGCACGAAGGTGTAGCACCTTCGTACTCGGCGTCGCTGTCCTCTTTTCTTAAAGAGT
CCAGCGGCGTGATGGAGGCTGTTAGTGAAAACGCTGACTCCATCGACGAGGAAGCAGAACATTCCGGTTCTGAAACCTCGGACGTAGAAGGGTTA
CTGCAAGGCAGTAGCTCTACTACGATTATTCGGAAAAGAAATCGACTTTCTCACCGAATAAAGTACGAATGTCCATGGAAACTCCATGCGGCATGCG
CACTCGCTACTCTCAGATCCGAGCCCGGACCTGAGGTAGTCGTCTGGTCTGGAGACGGCATCCGTCTCCAAGACCCGCTCCCACCTAGGTTGTTTGG
ACCGAAGTGGGATGGATCTCTGCGAAACAAGTTAAGGTTTCACAAGATCGCGGATGGTAACGTGAAATCTCACGTTATCTACCGACACACTCACTG
GGGACGACGTCTCCAGCGAGAGTGCGCAGACCCAGAGTCTAAAATTAGATACTGGGCCTGTACCCTCCGCAGCCGAATTAATCGGTTTTTGGCGGG
TAAATCCGATCCCATCTGGACTTCAGATGAGAAAAGGATTCTGTCGCGGGGCGGGTCTTATCAGACCCGTGACCGTGCAGCACGGTCATTAAGGTTG
CTTGAACTCTTAAAGACCGTGGACGGGGTATTCACACAGAGATATCTGGCGAACCCCGCTGAAGTGTGGACATGGGACAGATACGACCTGTTCACA
CTTGGAACTCTATCCCTATTAATAGGAGACGAGTTCTTAGACGGCGAACTACCTCAAGAGGTATTACCCGTCAGCACTTCCTATTCCACACTTAAGTG
GACTAGAAAGTGGTTCAAGAAGGTGTCTCATAGAGATCTTCTGGAACGTGAGACCAAGCCCCCGCCCGAAGGGCAGGAATGGTCTCGGCTGCTTT
GGAGAACATGGAAGGTTCTTCAATCAGCCTCGGGATACGAGCGCCTATTGATAATAGGCATTCTATCTCAGACTAGGGGTTGCGGTACACCGCCACC

ACTGGTCGTACTCCAATCGAAACGGAAATTCCTCGAGACCGTTTCGCTGGAGCCCCCGAAGAGAGCGCCACAGTGCGCTCCCTCCGGAGGATGGC
AGTGCAGGAGGTCTTGGAAAACCTCCCGCCTGTCGCCGTGACAGGACTCGCGACCAAGTCGCGTGTAACTGTCACGTCCGCCGCTTGCTGGGAAA
AGACCCGGAAAGAAGGCGGAACGACGGAGCAGATCAAGCAAATGATCTGCGACCTCGATCCTATGTCCCAAGTTCCCGTGAGGAACCTGGACACA
GGTAGGGTCGAACGCTGGAAGTTCCAGGACGAGTTCGACTCTGTTGGAGAACTTATATTCTGGGTCAGTCTTGACCGAGTTCTCCACACACCACCA
GTGGAGCTTCGCAAAGCTTTTCTCACTGTGGTGAAGGAGCCTGGTAAAGCTAGAAGCGTTACCAAGGCCCGTGCTTGCCTCAAGATCGTGCTCGAT
CTTGTTAGCAAGTTATGCTCTGAGCCCTTTATAAAAGGAATCAGAAGCAGCCAGTCTGGAATGAGCGCATCAAACCACGGCTGGAACTTTTTCAACT
CATTCTCGAATGAGATTGAAAGAGAAGAGACGTTTTCTCTTTTATCAAGAGAAGAACGCTCGTTCGAAGGGTATGTTGAACGAACAGATACCTTCGA
AGACCTCTATGTGTCCTCCACGGACTACAGTGAGGCTACCGACCGGCTCCAACACCAAGTTGGAATCGATCTGGGAGTGCCTTTGATGACCAAATGT
GGTATACCAAAGGTACTGCAGGGTATTGTAGTAGAAACTTGCTACAATACCAGGCAGATCTACTTCAAGGCCACTGGCCTTTTGGAAGATCTGGGTG
AGCGTACGGATTCCGAAGGAATCCACGTAATCACCCTTCGTCAAGGTGTCCTCATGGGGGACCCCTTGACGAAACCGGTCCTACACCTGATTAATGT
GTGTGACCGGCTCCTGCAAAAGCGGCTCTTAGAGCCCGACTTTTACGGCAGGTTGGGGAATTTCCATGAAATTTCCCAACTTCTCTCACGCGTTAAG
AGTAAACTTAACGAGTGAGATCTATCCGTGGTTGAGATTTTAGACTCACCTGCGGGTAACGTATAGCCCCTAACT

> Cb_clean_k141_15570
GGGTCGGTGGTCGTCGCGGAGGACATGATTGCATGTTCACGGCTAAGAATAAATGGCCCTACTTAATTGCTGGTGTGACCATCATCACTAGCCTGAC
AATTGAACCACTCCTCTGCGACGACCACCGACCCCACATTGCTCATGCGGTTATGGCGATGCCCGATATTCCTTCTTTCAGTGAAGACGTTAGTGGAG
AAGTCAGAGACGTCGCCAGGTCGAAGTTGAGCTTAGGTCGGGGTGGCAACGGTTGTGGTGGAAGCGGCGTGTTGGGAGGTGCAGACATGGTATTG
CGCTGGAGCTGGAGGCGTGTCGTCGATGCTGTAGAGCAAAGGCGCTCGCCGTTTCCAGGCGATTATATACAGTGTCGACTAGACCTTCGATATCTCC
TTCACGGCTGCCGATGGCGCATTCTCAACATGTCGTGCTTACCAAAGCCCCGACCGGCCCTTATGGTAGATGGTGAAAGGCGACTCTGTTTCGTCCT
AGTCCCTAAATGGCATTGTCCACCACTCTTTGGGGGCATTGACTTCTTCCATCCCGCCATCATTCACGCGTGGCCATAGTGCACTCTGCACGGTGTAT
AAATTATAACGTTGCTCGGCGAGGTTGTTTGCCACAGATGGCATCCCTTCATCATCGCTCGTCAGTCATATTCTTTTCGTGTGTCGCTCTAGCTCAAGC
TGGTTGTTCCTTGTCCAAATCGTGCTTGAAAAAGCAGCCGGCAGCCATCTTATGCGTGTACGCATTTGGCCACGGAGTATAGTGATGGTTCATTCGCT
CTGCAAGCTTTCGTCAGGCCCGTGACACCGCCTTGCTCATGGTTGATATAAAGCGGAGGTCATCATGTACTCCGAATTCTGAGACACGCTCAGCACC
CGCTCTTGCAGATTAAGGCGTGAAGGAGCTGAAGTTCAGTGTTCCCTTCTGACCATAGAGCATGTCGGACAAAGATGTGCTGCCGTTGCGCGCTTGT
CTCGATCAGACAAGCGTCTTTTACGAAGGAGAGTCGAACTCACCCATGTCGTCTGTGATTGGGCCGCGCTCATTCAGCAAGTCGACAACTTCCTGA
CCGTCTCTTTGATCGACTCTGCCCATGTCATGATCCTGTTCGGCATGTGGTATTCCATACTCCGGAGGCCGCGTGTCGACTGGGAATGTCGAAGCCTG

CGCACTCGAATTCGTCGAGCAAATTAAGTATTGAGTATGAATTCATTTAAGAACTATTACTCAATCCTAGACGAGGAAGTCTCCGACTTCACCGCCTG
GGATCCCTGCGAGGTCATGCAAGACATGGCACCGCAGAGTCTGGAAGTCTGTACAAGTATCTTGTCACAGATGTTCCTGTCGGCAGCTCACCTTTGT
AATGAATGGGAGCTCCCGACCTTCGTATCCGACGACGTCAACGTCTTCGGAATGGAGGTAGGGGACGACCGCACCGACGCGAGTCGCAACCCCTAT
AAACTTGCGCTTGGGCTGATAGCCCAAGAACAAGTTTTGCATATGAAAACGTCTGATGAGGACACTTTCATATCGCATTCAATTTGGAATTTCTGCGA
AATTTCAAATGGAATACCTGCAGATATCACTGAAACCTTTGATTTCTGTAGGAAGAACGGCGACTTCGAGAAGCTCGGATTCGTCGATTCTCCTCCTT
TGCGGATCATCTTGGATGTCAGCAAAGGAGATGGCAGTCATTCTTCGACATCGTCGGGAAAGGCTGCTATGCTGGCCTCGAGGATGCGCACCCCGA
GGACCAGTATGCTGCGAGTTTTAAACCTCGCAAGCTTCATGCAGGACGGAATGCTCCGAACTAGCATGAATTCCGACCCAAAATACCTACCAAGGAT
AATGGGCGGATCAGGTGCCCGGGCGTTGTTTGACAACCCCGAGAACTTGTACCTCTACACTCTTGCATACAAGGGTGGTAGATGTAACAGGGTTTAC
GGGTCGGCGACTCGTGAACTCCAGACGTGTCTTGCAGAGCTATCACGCTCTAAAGCCACGATGCCGGTGCTGTGCAGAATGCTTAGCGACCGGCAA
GAATATCTTCACGGAACGTATTCCAACATGATATTCGCTCCAAAGCTATCATTTAAGGATAGTCAAATGGAGAAGCTCCCGGCTCCTCTAATAGAGTC
ACCGGGAGGTGCAAACCGGTTCAATGCATCATTGAACCGGCTTGTCCGCACGAAGCACCTAGTTACTAGGTCCTCCGCGCTTCGCGAATGGCAATAT
TCCAAAACCATTCGTGCGCGCCTTCTGTCGAGATTATCGACAGAGAGGTACGAGTCCGAGGACCTGCTGCGAAAGCAGGCTCTTAGGAAGGCGTTC
GGGTGTGCACTCAGTGCAAACACCGCGTTCGCTAACATGCTTTCCAGGAAGGCCACCTGGAAAGATGTTGAGACGTTAATGCAATCGGAGAACCAC
ATTACATTAACGTCCGGATCACCTGAGTTCACATTATATGATGCTCAGTGGATCTGCTCCGGTGCGAAGTATGAAAACTTCAGCATCGAAGACCTGAC
GATCACGGAGGATTTACACTCTCGTGAAGACGTCAGCGAGGAACAAACCTTCAAAGTTGGTGGTATTGTCCTCCGTCCCATCATAGGCAACAAAATT
GCCCATGTTGAGACCTCGACCCGCGTTGGTCTCTACGAGATCAACGGGTCGATGCAGGAGTGGGCTAATGATCTTCATAGCCGGCTCCTCTCTCATC
GCGTCGACGGTAAACCGCTTCGACGCGACGATGTACTCACCGAGATCATGAAAGATCCCGAGTGGGTAAACGACGACACAGGACTAATAGAAATGT
GTCTTCGCGACACTCAGTCACTCCATCAAAGGAGTGCTAGAGTGGTTCTGGTCTCGCTCGACAAGCGTCTCGCGAACCAGATGTCCAACACTTGTA
ATGTACAAGTGGAAAGACTACCCCCGTTGGCGTACATTGTACGTATGATGGAGGAAGGCCTGGATCCTATCAAAGATAGGGACAAAGCCTCTCCTCT
CCTGTCAGGCTCAATACCTAGCAGAGAGAGGTCGGACCCGATCCGCGCGTTCTACGTGGATACGGGTTCTGTTGCCCATGTTCTTACCAATCTAGAA
GAATATGAGCAAGCTGCTTCCGGTTTCGTAAATTTACGAAAACGGGAACACGTGGACAGAGGGAGCAACAAAGGCTCCCGCTGGACACAGTACGT
TCTCAGGGATATCCCTGATCGCGTGCTCGCTCTCAATACAACGCCCGTCAGGCCCATATTGAGAGACCGGAAGTTCCCCCACTCACAGAGTCGGAGA
GCCTCTTTCCGCACAAACAGTTGGAGAACTGAGAGTGCGGCTAGTCAGACCAGGGTGTAAGGACCCTGGGACTGACTACTATCCCGACCTGGCATT
TAAGAGCTGGTTCGGGGTAACGTATAGCCCCTAACTGGGGAGCATTTACGTTAAAAA

> Cb_clean_k141_3161

CTTGAAACGGCAGCCAGCTAAGAGTTCGGCAAGAGGGGCGGGTTATCGAGATCGACGACTGACTCTACCAGGGAGCCCTGGAACGCAAGTAAGAC
TCCGAGGGCGGAGGAATGGCCGGTTTTATCCCAGACCAGAGAAGAAACCGCAGATACTATTCTAGACCAAACAACCAGTCCAATAAGCCTTGCTAT
GGACCTCAACTTATGAAGCTCAACGTAACTCAGAAGGGTCGCAAGATCAAAGGAAAGCCCAAGAAGAAACAAGGTAGCGATAAGACTTGTTACGA
TTGTGGCAAACCAGGCCACTTCGCGAGAGATTGCCGTTCGAAGAAGATGGTGCAGCGGCAGCCTTTCAACACGATGCAAAGGATAGAAGAAGGTC
GAGGGGCCTATCACGACAATAGATCGCCCCAGCAAATCCGACTCCTAGGCATGATGACCCAAAAGCGCAAACGGAAGAAACTATCGAGGAAGCC
CGTCAAGTCAAGGAAGATAAAAGACTCCAAGCCCTTCTCGGCAAGGACAAGAATGCGCCAACCTCCGAAAGGAAGAAGGAGCAACACGAGCCAA
GGACATTCGGAATGATGCAGCGACGAAATATCCCGAACCAAGTCCTCTGGGAACAGCGTAACGAACATGGAAGCCTTCACTGGCGATTCTGTTACG
ACGATACATGCCAAACACACGTATCTTCCAAGAGGGAGAACAAATTCTATCCGGTGGGACCATTGTGGGAAGAAAATCCTCCAATCCGGCGATATGA
TGACGGAAGTAGGCCATTGCCTGTTCCAGAGGAGCTACAAGATCTAGCGCCACACCTCAGACTCGCTGCTAACGAATCTTGTTGCGAAACCCCAGA
GAATATCCCACCTGAAAAGGAGGAAGAACAAGACTCGTCTGCAGAAGAAGACACGACGGCCGAGCCATCTGACGAAGAAGCAGACCAAGATGAC
GAGATCACAACATCCACATGTACCTAAACATGGATCATGCGAGAACTTGAGCTATTTCTGGCTATACGACAAGAGCAAATATTCCCAAGGATCGGAA
ACAAGCGATACTTACACCCGACCAAGTTCGACCGAGCCCTAAGAGAGCATCTAACTAAGCTCTGGAACCAACCATGAATCTTGATACAATACGACTT
CAGGACGCTCATACAAGAACTTTTTCGCATCGGAAGCACAATCGCGCCATTACGATGTGTCATGCCAAGCGAAGAACTCGTACTAAATCAGGTATGG
GAAAGCATACGCCAAATCTTGAAAAGATACGTCATGCTACAACATTGCCAATGCACATACGCTTGCATTGAAACAGCAGAAGAAAGAGCAATTGAC
CAAATGGTTGAACACACGTCACATCAGATCCACTAGTGGCATGCAACACCAATCACCGAGATCCAATCGGGAAAAGGATCGACTCTCAAGGAACGA
GCGTCCCAAGAGAGTTCTGCAACATTGAAAGTGCTCAAGCATCAACATTAGTCTTTCGCACCAACATTACCGTCAACGGACACAAATTTCAAGCCAT
GATCGATTCTAGAGCAAGCGGGAACTTTGCCTTCTGATCATTCGTGAAGCGATATGTGATAGCTACCCAGTCGAAGGACGTCGGGTACGAATTGACC
GCGGAACCAGGCCTTGTTCATTTCCTTGCCAGAGAAGATGACATAAGGTGGTACAGACCAGCCTTTGGCATTAACACAGGCAATAACAGTTTGCCAT
TGTGTTGAATCAGAAACCAAGGCTTTAGGTCGTTGCCTACGCTCTGTGCCAGTGAC

> Cb_clean_k141_38712
GAGGGCCCTTGAAACGGCAGCCAGCTAAGAGTTCGGCAAGAGGGGCGGGTTATCGAGATCGACGACTGACTCTACCAGGGAGCCCTGGAACGCAA
GTAAGACTCCGAGGGCGGAGGAATGGCCGGTTTTATCCCAGACCAGAGAAGAAACCGCAGATACTATTCTAGACCAAACAACCAGTCCAATAAGCC
TTGCTATGGACCTCAACTTATGAAGCTCAACGTAACTCAGAAGGGTCGCAAGATCAAAGGAAAGCCCAAGAAGAAACAAGGTAGCGATAAGACTT
GTTACGATTGTGGCAAACCAGGCCACTTCGCGAGAGATTGCCGTTCGAAGAAGATGGTGCAGCGGCAGCCTTTCAACACGATGCAAAGGATAGAA
GAAGGTCGAGGGGCCTATCACGACAATAGATCGCCCCAGCAAATCCGACTCCTAGGCATGATGACCCAAAAGCGCAAACGGAAGAAACTATCGA

GGAAGCCCGTCAAGTCAAGGAAGATAAAAGACTCCAAGCCCTTCTCGGCAAGGACAAGAATGCGCCAACCTCCGAAAGGAAGAAGGAGCAACAC
GAGCCAAGGACATTCGGAATGATGCAGCGACGAAATATCCCGAACCAAGTCCTCTGGGAACAGCGTAACGAACATGGAAGCCTTCACTGGCGATTC
TGTTACGACGATACATGCCAAACACACGTATCTTCCAAGAGGGAGAACAAATTCTATCCGGTGGGACCATTGTGGGAAGAAAATCCTCCAATCCGGC
GATATGATGACGGAAGTAGGCCATTGCCTGTTCCAGAGGAGCTACAAGATCTAGCGCCACACCTCAGACTCGCTGCTAACGAATCTTGTTGCGAAAC
CCCAGAGAATATCCCACCTGAAAAGGAGGAAGAACAAGACTCGTCTGCAGAAGAAGACACGACGGCCGAGCCATCTGACGAAGAAGCAGACCAA
GATGACGAGATCACAACATCCACATGTACCTAAACATGGATCATGCGAGAACTTGAGCTATTTCTGGCTATACGACAAGAGCAAATATTCCCAAGGAT
CGGAAACAAGCGATACTTACACCCGACCAAGTTCGACCGAGCCCTAAGAGAGCATCTAACTAAGCTCTGGAACCAACCATGAATCTTGATACAATAC
GACTTCAGGACGCTCATACAAGAACTTTTTCGCATCGGAAGCACAATCGCGCCATTACGATGTGTCATGCCAAGCGAAGAACTCGTACTAAATCAGG
TATGGGAAAGCATACGCCAAATCTTGAAAAGATACGTCATGCTACAACATTGCCAATGCACATACGCTTGCATTGAAACAGCAGAAGAAAGAGCAAT
TGACCAAATGGTTGAACACACGTCACATCAGATCCACTAGTGGCATGCAACACCAATCACCGAGATCCAATCGGGAAAAGGATCGACTCTCAAGGA
ACGAGCGTCCCAAGAGAGTTCTGCAACATTGAAAGTGCTCAAGCATCAACATTAGTCTTTCGCACCAACATTACCGTCAACGGACACAAATTTCAA
GCCATGATCGATTCTAGAGCAAGCGGGAACTTTGCCTTCTGATCATTCGTGAAGCGATATGTGATAGCTACCCAGTCGAAGGACGTCGGGTACGAAT
TGACCGCGGAACCAGGCCTTGTTCATTTCCTTGCCAGAGAAGATGACATAAGGTGGTACAGACCAGCCTTTGGCATTAACACAGGCAATAACAGTTT
GCCATTGTGTTGAATCAGAAACCAAGGCTTTAGGTCGTTGCCTACGCTCTGTGCCAGTGACAACCATAGTATTCTGACAGTGTCCAAGCCTGAATCC
ACTC

> Cb_clean_k141_27828
GGGCGGAGGAATGGCCGGTTTTATCCCAGACCAGGGAGGGCCCTTGAAACGGCAGCCAGCTAAGAGTTCGGCAAGAGGGGCGGGTTATCGAGATC
GACGACTGACTCTACCAGGGAGCCCTGGAACGCAAGTAAGACTCCGAGGGCGGAGGAATGGCCGGTTTTATCCCAGACCAGAGAAGAAACCGCA
GATACTATTCTAGACCAAACAACCAGTCCAATAAGCCTTGCTATGGACCTCAACTTATGAAGCTCAACGTAACTCAGAAGGGTCGCAAGATCAAAGG
AAAGCCCAAGAAGAAACAAGGTAGCGATAAGACTTGTTACGATTGTGGCAAACCAGGCCACTTCGCGAGAGATTGCCGTTCGAAGAAGATGGTGC
AGCGGCAGCCTTTCAACACGATGCAAAGGATAGAAGAAGGTCGAGGGGCCTATCACGACAATAGATCGCCCCAGCAAATCCGACTCCTAGGCATGA
TGACCCAAAAGCGCAAACGGGAAGAAACTATCGAGGAAGCCCGTCAAGTCAAGGAAGATAAAAGACTCCAAGCCCTTCTCGGCAAGGACAAGAA
TGCGCCAACCTCCGAAAGGAAGAAGGAGCAACACGAGCCAAGGACATTCGGAATGATGCAGCGACGAAATATCCCGAACCAAGTCCTCTGGGAAC
AGCGTAACGAACATGGAAGCCTTCACTGGCGATTCTGTTACGACGATACATGCCAAACACACGTATCTTCCAAGAGGGAGAACAAATTCTATCCGGT
GGGACCATTGTGGGAAGAAAATCCTCCAATCCGGCGATATGATGACGGAAGTAGGCCATTGCCTGTTCCAGAGGAGCTACAAGATCTAGCGCCACA

CCTCAGACTCGCTGCTAACGAATCTTGTTGCGAAACCCCAGAGAATATCCCACCTGAAAAGGAGGAAGAACAAGACTCGTCTGCAGAAGAAGACA
CGACGGCCGAGCCATCTGACGAAGAAGCAGACCAAGATGACGAGATCACAACATCCACATGTACCTAAACATGGATCATGCGAGAACTTGAGCTAT
TTCTGGCTATACGACAAGAGCAAATATTCCCAAGGATCGGAAACAAGCGATACTTACACCCGACCAAGTTCGACCGAGCCCTAAGAGAGCATCTAA
CTAAGCTCTGGAACCAACCATGAATCTTGATACAATACGACTTCAGGACGCTCATACAAGAACTTTTTCGCATCGGAAGCACAATCGCGCCATTACG
ATGTGTCATGCCAAGCGAAGAACTCGTACTAAATCAGGTATGGGAAAGCATACGCCAAATCTTGAAAAGATACGTCATGCTACAACATTGCCAATGC
ACATACGCTTGCATTGAAACAGCAGAAGAAAGAGCAATTGACCAAATGGTTGAACACACGTCACATCAGATCCACTAGTGGCATGCAACACCAATC
ACCGAGATCCAATCGGGAAAAGGATCGACTCTCAAGGAACGAGCGTCCCAAGAGAGTTCTGCAACATTGAAAGTGCTCAAGCATCAACATTAGTCT
TTCGCACCAACATTACCGTCAACGGACACAAATTTCAAGCCATGATCGATTCTAGAGCAAGCGGGAACTTTGCCTTCTGATCATTCGTGAAGCGATAT
GTGATAGCTACCCAGTCGAAGGACGTCGGGTACGAATTGACCGCGGAACCAGGCCTTGTTCATTTCCTTGCCAGAGAAGATGACATAAGGTGGTAC
AGACCAGCCTTTGGCATTAACACAGGCAATAACAGTTTGCCATTGTGTTGAATCAGAAACCAAGGCTTTAGGTCGTTGCCTACGCTCTGTGCCAGTG
ACAACCATAGTATTCTGACAGTGTCCAAGCCTGAATCCACTCTCATCAAAGTTGTATATGTCGTGCTCTGTGATTCCATAGAAGGCTATTATGTTTTGA
ACCAGTGTAAACCACTCTGTAATAACCTCTGGGTCCTCATTC

> Cb_clean_k141_41134
AGCGATAAGACTTGTTACGATTGTGGCAAACCAGGCCACTTCGCGAGAGATTGCCGTTCGAAGAAGATGGTGCAGCGGCAGCCTTTCAACACGATG
CAAAGGATAGAAGAAGGTCGAGGGGCCTATCACGACAATAGATCGCCCCAGCAAATCCGACTCCTAGGCATGATGACCCAAAAGCGCAAACGGGA
AGAAACTATCGAGGAAGCCCGTCAAGTCAAGGAAGATAAAAGACTCCAAGCCCTTCTCGGCAAGGACAAGAATGCGCCAACCTCCGAAAGGAAG
AAGGAGCAACACGAGCCAAGGACATTCGGAATGATGCAGCGACGAAATATCCCGAACCAAGTCCTCTGGGAACAGCGTAACGAACATGGAAGCCT
TCACTGGCGATTCTGTTACGACGATACATGCCAAACACACGTATCTTCCAAGAGGGAGAACAAATTCTATCCGGTGGGACCATTGTGGGAAGAAAAT
CCTCCAATCCGGCGATATGATGACGGAAGTAGGCCATTGCCTGTTCCAGAGGAGCTACAAGATCTAGCGCCACACCTCAGACTCGCTGCTAACGAAT
CTTGTTGCGAAACCCCAGAGAATATCCCACCTGAAAAGGAGGAAGAACAAGACTCGTCTGCAGAAGAAGACACGACGGCCGAGCCATCTGACGA
AGAAGCAGACCAAGATGACGAGATCACAACATCCACATGTACCTAAACATGGATCATGCGAGAACTTGAGCTATTTCTGGCTATACGACAAGAGCA
AATATTCCCAAGGATCGGAAACAAGCGATACTTACACCCGACCAAGTTCGACCGAGCCCTAAGAGAGCATCTAACTAAGCTCTGGAACCAACCATG
AATCTTGATACAATACGACTTCAGGACGCTCATACAAGAACTTTTTCGCATCGGAAGCACAATCGCGCCATTACGATGTGTCATGCCAAGCGAAGAA
CTCGTACTAAATCAGGTATGGGAAAGCATACGCCAAATCTTGAAAAGATACGTCATGCTACAACATTGCCAATGCACATACGCTTGCATTGAAACAGC
AGAAGAAAGAGCAATTGACCAAATGGTTGAACACACGTCACATCAGATCCACTAGTGGCATGCAACACCAATCACCGAGATCCAATCGGGAAAAG

GATCGACTCTCAAGGAACGAGCGTCCCAAGAGAGTTCTGCAACATTGAAAGTGCTCAAGCATCAACATTAGTCTTTCGCACCAA

> Cb_clean_k141_45057
TGCTTGCAAAGTAACCACTTTCCCTGGCCTCCTGCACGGCTTCTTGGCCAGCCTGGGCGGCTCTGTACTTGCTGATGGAGTGCTTCTTGGCTTGCTT
CAGAATTCGCTGGCCCTGCGGTCGTTTGAGTGCGCTTTTAACGTTGTTCACAGAAGATGCGCCTGCTCGGCGCCTACGGCCAACCTCGCCACCGAAT
AAAGTACTCGTGTTGCGTTTCCAGCACCCTCTTGGCCTCCCACTCGCCTTCCCCATCTGAGAGCGGCAAGCTGTCCGCCTGCGTCTTGTCGCCGCGC
CACGGCTCAAGAAGAGATATGTGGAAGATGTTGTGGATACGCGAATCAGACGGCAGTGCTAGGCGGTACGCCTGCTTTTCGACTGCGCCAATTACCT
TGAAAGCTCCAGCGAGCTTACCTGCAAGCTTCTTTGATGGCACCTTGAA

> Cb_clean_k141_21308
GGGCAGGCAGTCTATAGAAGATAATCTACAAAAGTTAGCATTATGAAGTAGCAGTCGACCAGGAGCACTCGCCCTCCAGATGACGCTACCGCTTATT
GTGTCCAATTGTTCCGGCAGGTCAGGGTTCTCTAAAACTCCATGCGACCCTGATTCCGGTTGTCCCGGCGGTTCGGAGTTTACCAGACTCTATGCGAT
CCCGAGAAGTGGTCACCCCACCTTGAGCCGCTGCCCAAGGTCCTGATATCTTCCCATTTCAACCGCCTTGCTCTTGCCCGCGATTTTGCCGCTACTGG
TATCGAGATGGTTCCTTGCTCCGCTCGCAGGAACGCACGCCCGAAGGCTGGCGAGCCTGGACCTCGATGCATCGTTGGTCCCCGCTCCGGGCGATG
CTCGGAGTATGTTTGGAAGGATTACTCGAAGTGTGATGCGAAGATGTCCAAGCCGGAGTGGGCGAGGCTTCGCGAGATCCGTGACCGGATAGAGGG
GGAGTTGGGTGAGGTGGTTGGAAAGGAGATCGCCTTGCTCCACGAGCTAGCGAAACACCGTTCTAAGAAGCTGCGCCTGCAGAAGCAGCTGAAAT
TTGCGCAAGGGCGGCAAACCGAAGAGATGGCTAAAGAGATAGACGCTCTTGACGAAGTCGACGAGTTGGAGCGTGAGATCTTCGGTGTCATGGAA
GAGGAGCAGGATGTCGCAGCCCCTAGCATTCCTTTCTCGCTGGACAAGGAATTGGAAATGTTCCCTGATTGCTGGAATCTTGTTGATCCTGGAGAGT
GGTGGATTCTTTGTGAGATTCCTGGTGGAACCGCTGCATAGGCTGGTGGCAGTTCGCAAGGCGGTGCTTATCCGAGCCACTTCTTTGGTGCCCTGAG
CCACTTGGCGAAGGCATGTGCAATGGAGAGATCTGGCCTTAGAGCGCAGAACTCGTCTTATCCTGCTCGCTTGCTTGCTTGCGATTCCTTCGATCACT
TCGTTTCCTGGTCTGTGGAATTCCATGAGTTTCTAGTAGCAATGGGACCGATGTTCGGATGGTTCCCAGGTGTTGTCGGACTCTGGGTACCCTTTTTC
ACTTGATAAAGTATTCGTTGCCATCCCATGCTTCGATTCTTTCGACTTCGTACTCATCTTCTTCCTCAGATTGGAAGTGGAATAACTCCTGACACGGGG
TGTCGGCGTCGGCGGGTTCCAGTAGTGATACATGGAACACTGGGTGGATCTTTGCGTCCTTTGGTAGCTTCAGTCGGTAGTCAACTGGTCTTCTGGC
TTCGTCAATCAAAACTGGTCCGACCCGAGCACTTTCTCCCTGCCCGCCAGCGCTCGCCGGTCCATCTCATCCGCGGCCTCGTACTTGCCTTGGTGTT
GCGGCACGAGCGCCAGATTGCTCACGCTCGCCAGCGTGGAGGGATTCTCCACCCCAAGCACTTTCTTCCAGCCCTCCGTGGCTCGCAGGTTCATCT
CCTCCGCCACCTCGTACATGCCTTGCTCTGTTGGATACCCGCCAATTGGTCCATCCTAGGCTCTCAACAATGTTCGACATGTCCCACACGATGAGTCA

TTCAAGAGAATGGAATGATGGTCCGCTCGAATCAAGCACATGTCCATGCCCCCAGGGTGTGATTTTGGGGTTTGAAACACGCAAGAAAACGCGTTC
ACTGCGGATACCTCTACTTCCGG

> Cb_clean_k141_31340
CGGTTGTCTTAGCTAACGCTTATTATTATATCCAATTGTCCCGGCAGGTCAGAGTTCTCTAGTTCTATGCGACTCTTTTTCCAGCTCTTCCGGCGGTTCG
AAGTTTACCAGACTCTATGCGACTTTCATAAGCCGTCTACCCACCTTGAGCCGCTGCCCAAGGTCCTGATATCTTCCCATTTCAACCGCCTTGCTCTT
GCCCGCGATTTTGCCGCTACTGGTATCGAGATGGTTCCTTGCTCCGCTCGCAGGAACGCACGCCCGAAGGCTGGCGAGCCTGGACCTCGATGCATCG
TTGGTCCCCGCTCCGGGCGATGCTCGGAGTATGTTTGGAAGGATTACTCGAAGTGTGATGCGAAGATGTCCAAGCCGGAGTGGGCGAGGCTTCGCG
AGATCCGTGACCGGATAGAGGGGGAGTTGGGTGAGGTGGTTGGAAAGGAGATCGCCTTGCTCCACGAGCTAGCGAAACACCGTTCTAAGAAGCTG
CGCCTGCAGAAGCAGCTGAAATTTGCGCAAGGGCGGCAAACCGAAGAGATGGCTAAAGAGATAGACGCTCTTGACGAAGTCGACGAGTTGGAGC
GTGAGATCTTCGGTGTCATGGAAGAGGAGCAGGATGTCGCAGCCCCTAGCATTCCTTTCTCGCTGGACAAGGAATTGGAAATGTTCCCTGATTGCTG
GAATCTTGTTGATCCTGGAGAGTGGTGGATTCTTTGTGAGATTCCTGGTGGAACCGCTGCATAGGCTGGTGGCAGTTCGCAAGGCGGTGCTTATCCG
AGCCACTTCTTTGGTGCCCTGAGCCACTTGGCGAAGGCATGTGCAATGGAGAGATCTGGCCTTAGAGCGCAGAACTCGTCTTATCCTGCTCGCTTGC
TTGCTTGCGATTCCTTCGATCACTTCGTTTCCTGGTCTGTGGAATTCCATGAGTTTCTAGTAGCAATGGGACCGATGTTCGGATGGTTCCCAGGTGTT
GTCGGACTCTGGGTACCCTTTTTCACTTGATAAAGTATTCGTTGCCATCCCATGCTTCGATTCTTTCGACTTCGTACTCATCTTCTTCCTCAGATTGGA
AGTGGAATAACTCCTGACACGGGGTGTCGGCGTCGGCGGGTTCCAGTAGTGATACATGGAACACTGGGTGGATCTTTGCGTCCTTTGGTAGCTTCAG
TCGGTAGTCAACTGGTCTTCTGGCTTCGTCAATCAAAACTGGTCCGACTTTGACGCGATCCAATCTCTTTGTCTTTCTTCTC

> Cb_clean_k141_14865
GACCTGCTCCTTTCTCTCAGCAACACCAAAGAAGAGGACTGACTTCACTCTCTTGCGAAACACAGCAAGCATACCACATTGGTGTTGTTATTGGATG
CAAAAGGCAAATGTGACAAAGAAGATAGCAAATTGTGACTGTCCTGCATCTTTGCAGTTCCCAGGTGCAGCTTCTCTTCCTATGTCGAAAACTGTTA
GAATGCAGCATCTCGACTTCAGTCTTAAGTTCTCAAAATTACTACCTGGTGGCAATTTCAACATGAACATCTACATGACTGCTTATGGTAATCCAATTC
CAATTGGAGATATACCTCAAGAGGTAGCTTCTGTGCTTGGGGTTGCGGGGTCATCAGACAACTATGTGTTGAGTGATGAAGATGTGAGGACTTACAG
CCAAGCATTAGTCTCACAGCCTCTTAGATGATTTTCTTTCTAACTGGAGTTCAAAGCACAAAGCCTAAATCTAAAATCAAAAACCACATGGGATTAGT

> Cb_clean_k141_25120

CCCTACAAATAATCAAGCTGGACCAACTAATTTCACAAGAGCAGTTATTCGACGAGACGACTCAACCTCACACTAGAACAGAATCAGGAAGTTCTA
CAGAATCCTTTAAATCAGCCTCCTCTATTGCAAGTAAAAGATCTTCAAAAGGGAGATTTCCAGTTAAAATGTCTGAGCAAACAGCACAGGGACAGA
AGTCAAAGGCTGGTGAGACAGGTGGCCGTCAAAGCCCCATTTCTGAGCCTGTTGGATCTATTTTAGGAATTGAGGGAACACGAGTCGAAGGCGCAG
CTATTCTTGTCAGAGAAGGTAAGAGTAAGGATGGCAAGTCATTTCGTTTCGATGTTTGGAATGACGTTGTTAAAGGCATGGATGTCAAGAAGCTCGA
GACTGCTCTTGCAAAAGCGAAAGTTGATCCAACGTATGATATGACAGGATTCATCAGAGCCATTGAATACCAAGGATTTGATCGTGAATTCTATATAC
AGCATGCTTTGTCAAAGATGTCTGTCTCAGTCTTTTGCAGGTTTGCGATCATCGGAGCAATCCGCGGGTCAAAGTTTGAGAAAATTTCTCAGGGGCA
GCCCAGTTCCCGCTCGGCGCGCGAACTTCAGACGT

> Cb_clean_k141_47347
GTTGGATCCATTCTAGGGATTGAAGGAACACGAGTCGAAGGCGCAGCTATTCTTGTCAGAGAAGGTAAGAGTAAGGATGGCAAATCCTTTCGTTTC
GATGTTTGGAACGACGTTGTGAAAGGCATGGATGTCAAGAAGCTTGAGACTGCTCTTGCAAAGGCAAAAGTTGATCCGACGTATGACATGACAGGA
TTCATAAGAGCCATTGAATACCAAGGATTTGATCGCGAGTTCTATATACAGCATGCTTTGTCAAAGATGTCTGTCTCTGTCTTTTGCAGGTTTGCGATT
ATTGGTGCGATCCGAGGGTCAAAGTTTGAGAAAATTTCTCAAACTTGTGAGGGAATGCCTAGTGATTTGGTGAGTGCCTTCCAAACCTGCTCTTTTC
TTTCGACAACACCAAAGAAGAGAACTGACTTCACTCTGTTGAGAAACACAGCAAGTATACCGCATTGGTGTTGTTACTGGATGCAAAAGGCAAATG
TGACAAAGAAGATTGCGAATTGTGAATGTCCTGCATCTTTGCAGTTCCCAGGTGCAGCTTCTCTTCCTATGTCGAAAACTGTCAGAATGCAGCATCT
CGACTTCAGTCTTAAGTTCTCAAAATTACTATCTGGTGGCAATTTCAACATGAACATCTACATGACTGCTTATGGTAATCCAATTCCAATTGGAGATAT
ACCTCAAGAGGTAGCTTCTGTGCTTGGGGTTGCTGGGTCATCAGACAACTACGTGTTGAGTGATGAAGATGTGAGGACTTACAGCCAAGCATTGGT
CTCACAGCCTCTTAGATGATTTTCTTTCTAAATGGAGTTCAAAACACAAAGCCTAAATCTTAAATCCACAAAAACATGG

> Cb_clean_k141_13474
AGCACCCCACAAAAGTAAAACTGGACCAACTAACATCTCAAGAGAAGCTATTTGACGAATCGAGTCAACTTGCACCACACACAAGAACAAGCTCA
TCTGATTCAACAGAATCATATAAGTCCGCCTCTTCTATAACAAGCAAAAGATCATCAAAAGGTAGATTTCCCATCAAGATGTCTGGAAATAATGCTCA
GGATCAGAAGGCCAAGCATGGCGAGACTTCTGAGAAACAAGTCCCCAGCTCCGCACCAACTGGTAGTGTTCTGGGCTTTGGCGGTGACAGAATCA
CCGGTGAACCTCCGCTGGTCAGAAATGTAAAGGACAAGGAAGGTAAAGCCACCAAGCGCGATTTTTGGAATGAAGTTATTAAGGGGCTTGACGCTA
AAAAGCTTGAGACTACTCTGGCTACTGCTAAGACTGATGCCACCTATGATATGACTGGTTTCATTAGGGCCATTGAGTACCAGGGATTTGATCGTGAA
TTCTACATAAAACATGCATTGACTAAAATGAGTGTCTCTCAATTTTGTCGATTTGCCATCATCGGCGCAGTCCGTGGATCTAAATTTGAGAAAATTGCC

AACACTTGTGATGGCATGCCAGCAGACCTCGTTAATGCCTTTCAAATCTGTGGATTTCTTTCAGGAACCCCAAAGAAGAAAACTGATTTCACTCTTT
TGAGGAATACTGCAAGCATCCCTCATTGGTGCGTTTACTGGATGGACAAAGCGAAAATCTCAAAGAAGATTACGAACACCACCTGCCCAGCTGCAC
TTCAGTTTCCAGGAGCAGCTTCTCTTCCTATGTCACGAGAGATTCGGATTGCTCACCTTGATTTCAGCTTAAAATTCTCAAAGCTTTTGCCAGGTGGC
AACTTCAACATGAACATTTACATGACAGCTTATGGAAATCCGATACCAATTTCTGACATACCTGTGGAAGTTGCTGAAATCTTAAAGATACAGGCTGA
GTCCGATAATTATAGGTTATCCGATGAAGAGATTCAGACTTATAGTCAGTCACTTGTTCAGATGCCTAGAAGGTAATCCACCTCCCACAATGAAATTCG
TGTCTATAGGAGAATGGAGTTTGGATTCCATGTTCAAATGGAGTTTTATGGTTTTGTTTCTGGTT

> Cb_clean_k141_12481
TAACGTTATGCCCAAGATTAACAATGATGGATACATAAAGATGATGAACTATCTTTTGAAGTACAAACCAAAGTGTGCTTCTGAGGAATTTGCCTTTC
AACTGATAATCAGGACATTTGACATGAATGAGCTAGTGGTGGTGGCCAACCCATTCAAGCTTCCTTTCATAGAAGTTGGAATGCAAATAACAGATTC
AACCCTTGTCAAATGTTTCACTTTATTAGAGAATGAATATCGAGCACTTTGGTTTGACCAATTAAAGCACCTTCACATCCCAGCATTTCATGATGAATT
TTCAGAGTTTTCCACAAATGATTTCTCAAAGAGAAGATCAGAATCACAAAGC

> Cb_clean_k141_67601
ATCTCCCGAGCACTGCTATGTTACTGGGCTTCTGTGGAACAAAATAACAGGCGATGGCTGCTCGGACCTGGGGACTCGAGCCTATTCGACGATATGC
TGTTGATGGCCTGTGTGATGACCCATGCCAGGCATCGAGGCTGGATAGATGTCGCCGCCGAGAAGCCCGAATGCTGAATTCAGCTCACGGGTGGTA
AATATACCCTCACTACGTCTACAACAAACCAAACAACCAACTTCAACCTAGCAGAACTCTCACAGAATGTCTCCAAACATGATTGCACCATTGCTAA
AGAAGCAGATGATCGAGTCCCAAACCCCCAATGGTAAGTGTTATCTGAGATTAATATGTGTTTTGCCATCCTTTCCTGCCTGTGACCCAGCTCTCCTT
GAAGAAGTCATTGTCCACATGACGTTGTCCTTGAGATCCAATCCCAAAGGACTGGATGTTCTTCATTCCCTTTGGAATGCAGAGAAGATTTTGCTGG
ACGAGAAAGATAGTGCAATTGGAATTGAGGATCATGAGTTGAATCTCACAACTGATAGCCCGGTTCCGCTATGGTTCATTGAATCAATAATGTCGGCT
CCATTTAGATTTGGGTTTGAAGATAGAGGCGGAGTGATGCATGTAACATACTATCTAAAAGATACCACAGATTGCCAAGATTCAGGTTCAGAGATTGA
AACAGAACTCGGGTTTGCTTCTAGATTTATATTGCAAATGATGTGTAATCAGGGTAGACACATAGGGGCGACTATTGATCTTGATGACATATTTTGCGA
GATGCAGGAAGAGATTGCCCAGGGTGATTTTGAACCGAGTGAGTCTTATTTCTGTGAGACACCAACTAATATGCCACCCACTCCAAGCTTCCCAATA
GAACACAACATCCACACTGGTCTTTATGATCCAGATCCAATTAAGCGTGAGACTCAATCCCGGCTGGAGGATGTGTTCAACAGAATGGAGGGCGGG
AGGTCAACAACTCCAGATAAACAACGTGAAAGTAAAGACCATACAACAGAACTTCTAGAGAGAATGATGAGAATGGAAGAGAAGATGAATTCTGT
CCTGTCTATAAATAAGGAACAAACAGTGACCCCTGATGATTCAAGTAGTAACATTGGGAGGTATGAGAAGAAGTTCATGAAGCCTGGAACAGTTGTT

GGAAGTCAACATGTTTACAACCACACCCGTGGTGGAACAATGTTGGCAATAAAGGAAGAAAACCTTCCCCTAGACATAACAAAAGATGTTGCAGTT
GGGTTTGTCAAAACAAAAGATATGCGAGTTAAGGAGATTAAACAACTGACAAAAATTGCTCCAATAAATGGTCTAGCAAATCCATTCAAAAGCTCA
AGATTAAACATGCTCATGCATTTCCATACAGCTGTTCACAATGTTATGCCCAAGACTAACAATGATGGATACATAAAGATGATGAACTATCTTCTGAAA
TACAAACCAAAGTGTGCTTCTGAAGAATTTGCCTTTCAGCTGATAATAAGGACATTTGACATGAATGAGCTAGTGGTGGTGGCCAACCCATTTAAGC
TTCCTTTCATAGAAGTTGGAATGCAAATAACAGACTCAACCCTTGTTAAATGTTTCACTTTGCTAGAGAATGAATATCGAGCACTTTGGTTTGACCAA
TTAAAACACCTTCACATCCCAGCATTTCATGATGAATTCTCTGAGTTTTCTACAAATGATTTCTCAAAGAGAAGATCAGAATCACAAAGCCAATCAAG
TTCAAAGAGCTCAAAGCAGAATGGATATTATAGTAAGACACGTGGCGGTAGAAGTGTCATGTCCATATACAGGTGATTCTGATGGTTAAATGTAAATA
AAATTTG

> Cb_clean_k141_23000
TCCAAAAGTGTGAAGTAAATGGGGTCAATGGTCTCAAACTGAAATTAAGGCTTTCGAATTCGTTCAGATTTAAACGGCTCAATGATATGCAGAAATT
CAAGATTGTAACGAATCACAACAGTAAAACAGGTGAGATTGAAGTTTACTTAGAAGAAGATGGTGTCAGAACTGCAACGCTTTGTCATAGCAATGG
CAACTACTATCCTGTTGAGATACCACCAGGATACAAACTCAGTAAACCTGTCCAATATCTTGGTGTGGATCTCTCCAAGCTTCTTCAAAACAAGTCAT
GGTTTTACAATTTCAAATTGCCAATGATGTCTGAGGCAGAAAGATTAAGCTTTTTGACGGATAGTGTTGATTATTCTGAGATTTTATCACAAACAACG
AAATCAATACAGCGAA

> Cb_clean_k141_67281
AACCACCTATTCTGAGTTCCTCCAGAGGTCTGTCAATAAAGCAAGAGATTCAAAAATTGACACCAAATCCATGCTCAAGATAATATTCCCTCAATGGA
AATCCTTTGATGTAATAGCATCATTTGTTGGCAAGTTCGGTGCTGTCAAAAGTTCAAACAAACACTATTCACAAGCAGTCAGAACTTGGACTGTTAA
CAACTTCAACTATGAATTCAACTCAAGTTTAAAGTCAATACTTGAAACAAGTTTCGGAACTTCTCAAGAAGCTACAGAAGAAGATGTAGCAGAGTTT
AAGAGAATGATGTCTCTAAAGCTTGAAAATCTTGATCAATTCACTGACGAGTGTAGGAGAAAAGGACTCAGACCACTCGATGTTTTTTACTACATGA
CAAAAATATATAAATCTTCAAGAACATCAAAGGTTCAGACTTTTGCAAATGGTCCATCCACTAGCGGTTTGCACATGACTGCATTGGCAATCAAGCGC
TACAATCACATTCCCGGTGCATCAGTTCATATGGATGTTGGAATGGATGAGAGTGACCTTCACTCAGAGTCATCCTTGAGCATAAAGCTAGACAAGAT
AAAGTTATTATACAATATAATAATAATGAAAAAGAATGAAATGATCACTGGCAAGTTGACTGATTTCGACATGGTCTCACAAAATGGTTCATTGTCGG
ATGAATGTAGAACAATTGTGAGATCAATTAGATCATTGGCTGGATTTGACAACGTCACACAAAAAACACTAAAATTGTGTGCCTGCGAGCTTCTTCC
GATGTCAGAATTGAAAGAAAAGCTTGTCTCATGGAGAAGCATTAACTACAATTTCATAAGAAAACAACAAAAGAGAATCACCAAGTCAGGCTCTGT

TATTTGGTCTGGTGACATGTCTGTTCTTGTGAACTCAGAAGATGAATGCTTTACAATGAATGAAACAAATAACATAAGATATATATCATCTAGAGAGAT
CAAAGATCTTTCCTCTCTATATAGATCAATGAGGCAGATGTGTAAATCATTAGACATGGAGTTCCAGACTTTCTTTCAGATGTCTGTCATGTCAGTGGG
TGATGTTTATTTGTCAACAAACTCGAAGTCCCTCCAAAAATGTGAAATAAATGGGGTCACTGGTCTTAAACTAAAATTAAGGCTCTCCAATTCGTTCA
GATTTAAACGGCTCAATGATATGCAGAAGTTCAAGATTATAACAAATCACAATAGTAAAACAGGTGAGATTGAAGTTTACTTGCAGGAAGACGGTAT
CAGAACTGCAACGCTTTGTCATAGCAACGGCAACTACTATCCTGTTGAGATACCACCAGGATATAAACTCAATGCACCTGTCAAGTATCTTGGTGTGG
ATCTCTCTAAACTTCTTCAAAACAAGTCATGGTTTTATAATTTTAAATTACCAATGATGTCTGAAACAGAAAGATTGAGCTTTTTGGCAGATAGTGTTG
ATTATCCTGAGATTTTATCACAAACGACCAAGTCAATACAGCGAATCAATGATTATCTTGAGGTTCGAGAAGAAGTGAATGAGGAAGCATTTAGCATT
ATCAGAGATGCCAGGAACTCATCAATGGGATTCAACATATCAAGTGAAATTCAGGACGGAATGTCGATCAATGACTTGTTTAGACAAACAATGGACA
CAACTGAAATGCCAACTCAGCTAACAGAATTTAATGAGGATCTTGGTGATTGGGCTGACCATGTCAATGATCAAATGGAAAACGACCTTAGGTTGCT
AACAGAAGATCTTGAGAATGATGAGAATCTTGAGCTAACTAGAGCACTCGGATTCAAACGACCAGCGCCCAAGAGAGTTATGGCAACAATATCTGG
ATTACAGCAAGGATCAATCCTGAAATCAAGAATCCTGGATTGTTTTTTCAGTCACAAAATATACGAAACGAGAAATCACATCAATTGCCACACCAAT
ATGTCTGGTTGATGGGTGTGAAGATGGACCATCAGAAATTAGGTGAGCACTTGAGAGAAGAGCTTAAGGATCATATATTATCAGCCCTTGTTATGACA
AGTGGTGCGACTAAGAGGATCTTAAGACAGAAGATTGAGAATAGCAACACATATGGAATACCAATTCAAATGCTTTACAACATCCTCAACTCAACTA
CAATAACTACAACAAATATATTCGATGAAATACTAGAAGAGCAAAATGAAGACTATGCCTCTGACAGTGGTGATAGTGATGTGTGATCAAACCTCGC
ATCATGCAATCATAATGGACTTAATGGTTGAAATTTGATCTCTAATTCTTTTTCTGGTCGTGGGGGGGAC

> Cb_clean_k141_54286
CAGTGATATTCATGAATACGAGGTTGAATTTCCTGATTCATTTACATAAGCCCCTTCAAGAGATGTTATCAGTTGATAACACTTATCCGAGTCCTGATG
CTTTTGAGAAACTCTCAAACTTCATGTCTAGGCAGAGGGGCAAGGAGAGAAATCCACACAATGAGCTACTTTACCAAGTTATAAAGACAACATTGA
AGGGTCACAAAGTGAGGGCGAACCCTTTCAACTTGCCAATGCTGGAGGTTGGAATGTATTTGAATGACAAGTTAATCTATATGAGTTTCATGCAGTT
GTATCAAGAGTTTCAAATTGAATGGTTTAAATCTATGAAAGATATTGATCCACCAAAATTCCACAATCAGTTTAAGGATTTCAAATCCTTATCAAAATT
ATCCTCACGAACCCCTCACCATCAAAGTCGTCCATCTAGAAGAAAAGCAGGATCTGTCATTTCGAGCTTTGGGTTTTAAAACTTATACAAGTTGACTT
CAGTCACGGAACATACAAAAACAATATTGACATTGTTTGTCTGGCGTAATGGAATTGGACTGTGTTGAAAAGGTTTG

> Cb_clean_k141_12535
AGTTTTGATGCCAAAGGATTCATTGACAATATTAAATATCAGGGATTCAATCGGGAAGACTACATACGAGTGTCTCTCATGAAAATAACACCCCACCA

GATGCTGAGGCTCGCTCTCATGGGAGCAATTCGTGGTGCAAACTTTGACAAAATCATCAAAAGTGCTGCTGGCATCGAAGAAGATTTGCTGAACCT
GGCAAAGAGCAACACAATCGTCAGATCTGCTCGCCGCTCTGATGATATAACTATATTGAGATGCACAGCTGCAATTCCTCAATGGGCCTCATACTTCA
TGGGCAGAGCGAGTGTTGCTAAGAAGTTGCCGAACTTGGCATGCCCTGCCGTTCTGCAGTTCCCTGCTGCGGCGTCGTTGCCAATGTCTGCAGA

> Cb_clean_k141_33118
CCATTGCCTTCTCCAAAGTCATTAAGGGAACTTTTAATGAGAATATTTACATGGCAATGTTCAACAATCAGTTGCCCTTGAGTGAGATACCAGACGAG
TTGAAACTGGTTCTCAATGTCAAAACCGAATCCGATTCAATGGCTGTTGACGTTGCAGGCATTATTACGAGCATGATGTCCCAGGCTCAGGGCGGTG
GTGTTAGAGTTTGAAACTTATACAATTCGAATCAGTTACGAGAACATTGAAAGAAACTCATTGAAAAAAATCATGAAGATGTGCCAGATCATCTCGG
CGTAAACTAGGAGTATTTGACATGTGATTTTGGAAGGGGA

> Cb_clean_k141_39151
ACAGAACAACGGCCAAATAACTCACACATTTTTGAGAGCAGACGTGAATGAGATTATGTCATACAAAAGATGGACGAAAGGGCTACTAGACCTCCG
GTACTCTGGAAAATCATCTTTTGAGTTGTGTACCCTGTATAGATCTTACTGGGCAGTCGACCTGAAGGGAGTGACAAATGAGCAACCACTGGACTAC
ACTCATTGGAAAGCCCCCGACAAGAAGCCAAGCTCGATATACTGGTCGTCTCACTCTGCGGATAACCTACTCGATACAAGAGGACCACTGACAGGA
TATATAGGGACTGCCACCCGAGAGAAAAGGTCAGAGCACGGATACAAGATTGTGGACACCGGGGCCCCGTCTAGATCAGTTATGAAGCTTCAACTG
ATCCGTAGCCAAGCATACGGAAATCCGTCGTTCAACAACTTGTTGGACCGAATATCTCTCACTAGATCTCCAGTCCCTCTTTCCACAATATCCGATGTA
TTACCGAAAGTTGTCGGAGGGTCAATCTCGCACCGATATGCATCATCAATCAGGTCCATGGGAGCCTCTTACGTCGGCCCCTTAAACTTTGCAACTCA
CACCCGAGTCGACACAGACAACATCTCTAAACTGAGTGGTTCTGTTGTCAACTACCCTGTGATGCTTCAAGAATTTATCATCATGGCTCAAGCGGGA
GCAAAGATGATGCACTTACACTTCCGGTCATCAGCAGGTGGATTATATCTTCCATTTGATTCACTGATACCTTTGGACGATGACTCGCTATCGTGTGAC
CCCCCTGATTTTGAACCGAAGCTCTTACCTCGCAGTACCCTGTCGTATAGTCCCACCTTAGCACTGAAACGCACTCTAGAAACAGATTTGGGTAGCAT
ACCGCGAGGGTCGTTGATCCCGCCTATGGACTATCAGAGCTTTCCGATAGTGTGGCTCAGTGTCGTGAATTTCTTCACATCAACTCTTCGAGATTCAA
ACCGAGCGAAGACCATAGCCGACACGCGGGGTCATATTTCACTACCGTCCAGCTTCCAGATTGACATTGCCGAGGCACATGCCTTAGGAGCAATGA
GGCTGGGAAGAGCAATCGCGCATGCTGTGACTCACATCGTGATTCGAGACACATTCAGGACACTTCACCTTCATCCGGAGCGCTGGGATGAGAGTT
TGTTCCTCGATCATAACATACGAGTGTGCGTGAAGACGATCCTTGCCTACTTAACTCACCCCATATTCCAGACACATAAAGATTATCTCAGTCTGCGGT
ATTCTACCCTGAGATACGGAAGAGCACTGTCCGTTACTAACCGGATGATCGCGCTGATACGACGAGAGGTGAGGTTGATCTTCAGCAAAGCAGATCA
CCCGTTCTGGACAGAGAGAACAGCCGTGTTCTCAGGAGAAGGGCCATCAGCATTAACTGAGGCGTTGGTGATACATGCGGCAATTAGACTCAGGTG

GTTATATGCGATAGGTCATCCGAAGGCCTCCTTGTACTCACGCACATTTAACGGTTTCACTATAAGTCCGAGGGGAGTGACTTTGACCCCGCAGGAG
AGTTTGAACCGCACCGCTTTACAGCTAACACGCCTCGCCAAGTTCTACCGAACGGACGGGGATGAGCTGTTGTCTGGATTTATGATGAAGTTGTCGT
CTTTCCAAGGACTAGCGATATTCAACGACGACCAGCGAACTGTCATGAGATTTTCCCGGGCCATCTCACCGACGCTGTACTGCGTACGGAATGTCAA
GCCGACCAGTGTGGTCAAAACTCTTGAATCTGCCCCAACACACTGCTTGAACTGTTGTCCGCCCAGCTCGCACAAGCATACTACTATGTGGTCACGC
TACAAGACCAGGAAGCACGGTGGGGTAAGCTCAGCAGGGTACACATGGCTCCCCATCGTGTCTACATTAGAGATCCAGCCCACCTGCTTTGTCATTG
GTAATGGAAACGGAGGGTTGGCAGATCTGCTCCTCACCAGTTATAACGTGGAAGTAGTAGGGCTTGATCTCGAGTCTGATATGCCTACCAATTCTGC
AACACTCCTTAACTACTTTCCTCTCGGAATTTCGGTGAAAAATAAAACTAGATTCAGACAATCAGATGTGTCAATCAACACCACTGGAAATTGGCTA
GAAAAGTCAGTAAGGACCCTTCTGATGACTGAAACCAATACTACATCAACTGTTATTGCGGACATCACAGGTCCCGCTGCTATCGACGTTCTCAGTT
CCACACTGGATGCTATGGACACCAGGTCTGTGGTATCTGCATACTGCAGGCTGATAGGCTCGAGAGATGACATCTCTGGATGTATACGCTCTCTTCCT
CGAGTCGTAACGTCTCGTCTGTGGGTAGTTTCACACTCAACGTTTGAAATTGAGATAATCCTTGAACTATCTCGTACAAACAGCAGCGCTCACCTGT
GCACAGGTGCTGGTCCCCTCATAGACTATCCTCTTGATCGCGCGCACCACGAGTTGATCCCCCAGAGACGAGACGAACTGCTGGAAGCAGCTACTC
GATCGGTCCACGCGTGGACTGATGAAACGTTCCCGAGCATGTATGAAATCACTCGATCACTATGCAAAAGTCTGCTCAATAAACCGAAGTCTACACA
GTTAAGATACACTGAGCGCTATGACCTGATGTTGGCGTACTGTGTGTTGACAGTGCGAGTTTCCCCCTCACCAGTGGAACTGGTGCAGTCGTGGATA
CTAGACGAGAGAGCTGAGACAGATCTGTTTTACTACTATCTGAACGAGTCGACTATATCACATCTCATCAGATACTCTGCCAGACTGTTGACGTGGTC
AGATACCTCAATGACCGTGTGAATCTCCTCTCCTCACTGGAAACTTAGGCAAAGTCATCACTTACCCAAGTGCTTGATGCAACAGTTCCAACAGTTT
TAAGTAAAACCGTGCTTAAAGCAAAGCTTAAATCTTCTTTCATCCGACCGAAACGCATTCCCCACCATTTTATTCATC

> Cb_clean_k141_21666

GCGAAGACCATAGCCGACACGCGGGGTCATATTTCACTACCGTCCAGCTTCCAGATTGACATTGCCGAGGCACATGCCTTAGGAGCAATGAGGCTGG
GAAGAGCAATCGCGCATGCTGTGACTCACATCGTGATTCGAGACACATTCAGGACACTTCACCTTCATCCGGAGCGCTGGGATGAGAGTTTGTTCCT
CGATCATAACATACGAGTGTGCGTGAAGACGATCCTTGCCTACTTAACTCACCCCATATTCCAGACACATAAAGATTATCTCAGTCTGCGGTATTCTAC
CCTGAGATACGGAAGAGCACTGTCCGGTACTACCCGGATGATCGCGCTGATACGACGAGAGGTGAGGTTGATCTTCAGCAAAGCAGATCACCCGTT
CTGGACAGAGAGAACAGCCGTGTTCTCAGGAGAAGGGCCATCAGCATTAACTGAGGCGTTAGTGATACATGCGGCAATTAGACTCAGGTGGTTATAT
GCGATAGGTCATCCGAAGGCCTCCTTGTACTCACGCACATTTAACGGTTTCACTATAAGTCCGAGGGGAGTGACTTTGACCCCGCAGGAGAGTTTGA
ACCGCACCGCTTTACAGCTAACACGCCTCGCCAAGTTCTACCGAACGGACGGGGATGAGCTGTTGTCTGGATTTATGATGAAGTTGTCGTCTTTCCA
AGGACTAGCGATATTCAACGACGACCAGCGAACTGTCATGAGATTTTCCCGGGCCATCTCACCGACGCTGTACTGCGTACGGAATGTCAAGCCGACC

AGTGTGGTCAAAACTCTTGAATCTGCCCCAACACACTGCTTGAACTGTTATCCGCCCAGCTCGCACAAGCATACTACTATGTGGTCACGCTACAAGA
CCAGGAAGCACGGTGGGGTAAGCTCAGCAGGGTACACATGGCTCCCCATCGTGTCTACATTAGAGATCCAGCCCACCTGCTTTGTCATTGGTAATGG
AAACGGAGGGTTGGCAGATCTGATCCTCACCAGTTATAACGTGGAAGTAGTAGGGCTTGATCTCGAGTCTGATATGCCTACCAATTCTGCAACACTC
CTTAACTACTTTCCTCTCGGAATTTCGGTGAAAAATAAAACTAGATTCAGACAATCAGATGTGTCAATCAACACCACTGGAAATTGGCTAGAAAAGT
CAGTAAGAACCCTTCTGATGACTGAAACCAATACTACATCAACTGTTATTGTGGACATCACAGGTCCCGCTGCTATCGACGTTCTCAGTTCCACACTG
GATGCTATGGACACCAGGTCTGTGGTATCTGCATACTGCAGGCTGATAGGCTCGAGAGATGACATCTCTGGATGTATACGCTCTCTTCCTCGAGTCGT
AACGTCTCGTCTGTGGGTAGTTTCACAC

> Cb_clean_k141_40192
GATAGCCTCTCGGACGTTGGCATCGCTGGAACACAAGTACCACGTCTCCGAGGCTCCAAATATAGCAACTCTCATCGACAACCCTTATGGCTTGCCT
CTTGAAACGGCGGTCTCACCCCTGAGCAAGGTAGGGGCACTGACGTTGGATGCTTTTCGAGGAGTGGTAGACAACCGAGACATCGCCCCATTACTA
CGAACCTCCGTCGACAAAGCTGAGTTGACGCTCAAGGAGGACTTACTGAAGATTCGACCCTTCAACCCCATTCTCATACACGACCTATTTGAGGCAT
CTGGGTTCGGGTCGATAAAACTTATGAAGAAGATGTTCGTGCACACACGAACTATTCAGACGGTGGCTCAACAGAACAACGGCCAAATAACTCACA
CATTTTTGAGAGCAGACGTGAATGAGATTATGTCATACAAAAGATGGACGAAAGGGCTACTAGACCTCCGGTACTCTGGAAAATCATCTTTTGAGTT
GTGTACCCTGTATAGATCTTACTGGGCAGTCGACCTGAAGGGAGTGACAAATGAGCAACCACTGGACTACACTCATTGGAAAGCCCCCGACAAGAA
GCCAAGCTCGATACACTGGTCGTCTCACTCTGCGGATAACCTACTCGATACAAGAGGACCACTGACAGGATATATAGGGACTGCCACCCGAGAGAA
AAGGTCAGAGCACGGATACAAGATTGTGGACACCGGGGCCCCGTCTAGATCAGTTATGAAGCTTCAATTGATCCGTAGCCAAGCATACGGAAATCCG
TCGTTCAACAACTTGTTGGACCGAATATCTCTCACTAGATCTCCAGTCCCTCTTTCCACAATATCCGATGTATTACCGAAAGTTGTCGGAGGGTCAATC
TCGCACCGATATGCATCATCAATCAGGTCCATGGGAGCCTCTTACGTCGGCCCCTTAAACTTTGCAACTCACACCCGAGTCGACACAGACAACATCT
CTAAACTGAGTGGTTCTGTTGTCAACTACCCTGTGATGCTTCAAGAATTTATCATCATGGCTCAAGCGGGAGCAAAGATGATGCACTTACACTTCCGG
TCATCAGCAGGTGGATTATATCTTCCATTTGATTCACTGATACCTTTGGACGATGACTCGCTATCGTGTGACCCCCCTGATTTTGAACCGAAGCTCTTA
CCTCGCAGTACCCTGTCGTATAGTCCCACCTTAGCACTGAAACGCACTCTAGAAACAGATTTGGGTAGCATACCGCGAGGGTCGTTGATCCCAC

> Cb_clean_k141_33467
GCACGGATGAAGGCAAAGATGACAGACAAGGAGGAGTCGGTAGCCAAGACGACACATCTCAGTAACCGGAACCTGATGTCAGGGCTATACACTAT
CGTCGAAGAGGTCCATGAAGTTCGCTCGATCGTCGAACTGTTTGGGTGTCTCAAGACCAGTGGACATCCTCTAATTGATACAGAACTTGGAGGACT

GTCAGCAGCTGAGGCAGCCAGGTCTGACGATAAGACATCCGTGGTGGACTCTCAGCGTCTGCGTAACACATTTTGTCACATCGTCCTAACTGCTTAC
ATCGCGAAGCACGGATCTTG

> Cb_clean_k141_54717
GCCTCCTTGTACTCACGCACATTTAACGGTTTCACTATAAGTCCGAGGGGAGTGACTTTGACCCCGCAGGAGAGCTTGAACCGCACCGCTTTACAGC
TAACACGCCTCGCCAAGTTCTACCGAACGGACGGGGATGAGCTGTTGTCTGGATTTATGATGAAGTTGTCGTCTTTCCAAGGACTAGCGATATTCAA
CGACGACCAGCGAACTGTCATGAGATTTTCCCGGGCCATCTCACCGACGCTGTACTGCGTACGGAATGTCAAGCCGACCAGTGTGGTCAAAACTCT
TGAATCTGCCCCAACACACTGCTTGAACTGTTGTCCGCCCAGCTCGCACAAGCATACTACTATGTGGTCACGCTACAAGACCAGGAAGCACGGTGG
GGTAAGCTCAGCAGGGTACACATGGCTCCCCATCGTGTCTACATTAGAGATCCAGCCCACCTGCTTTGTCATTGGTAATGGAAACGGAGGGTTGGCA
GATCTGCTCCTCACCAGTTATAACGTGGAAGTAGTAGGGCTTGATCTCGAGTCTGATATGCCTACCAATTCTGCAACACTCCTTAACTACTTTCCTCTC
GGAATTTCGGTGAAAAATAAAACTAGATTCAGACAATCAGATGTGTCAATCAACACCACTGGAAATTGGCTAGAAAAGTCAGTAAGGACCC

> Cb_clean_k141_40049
AAGACGGTGGACATAAAGGCTATAGTCGATAGGGTGAGTGCTCGAGATGTCCCCAATGACTGGAAAATCGTGAGTTTGTATCCTAAGGAGAGGGAA
TTCAAGCTTGAACCTCGAATGTTTGCGATGTTGGTTCTCGAGATGAGGTGCTTCTTTACTGCAATTGAAGCTAACATAGCTGAAAATGTGTTCAGGTA
CTTGCCGCAGCAGACAATGACGAAGTCTAAGACCCAAAACCAAGAAAGGTTTCTCAAGTTCACAGATCCTTTAAGATCTCGAGCTGACTACACCTT
GTTTTTGGAAATTGATCTGACAAGGTGGAACCTCAACTGGCGAGAATTGAGTATCCACGCAATAGGACACGATCTCAATAGAATGTTTGGCATGAAG
GGGACATTCACGGTGACACAC

> Cb_clean_k141_2670
GGTGGATTATATCTTCCATTTGATTCACTGATACCTTTGGACGATGACTCGCTATCGTGTGACCCCCCTGATTTTGAACCGAAGCTCTTACCTCGCAGT
ACCCTGTCGTATAGTCCCACCTTAGCACTGAAACGCACTCTAGAAACAGATTTGGGTAGCATACCGCGAGGGTCGTTGATCCCGCCTATGGACTATCA
GAGCTTTCCGATAGTGTGGCTCAGTGTCGTGAATTTCTTCACATCAACTCTTCGAGATTCAAACCGAGCGAAGACCATAGCAGACACGCGGGGTCAT
ATTTCACTACCGTCCAGCTTCCAGATTGACATTGCCGAGGCACATGCCTTAGGAGCAATGAGGCTGGGAAGAGCAATCGCGCATGCTGTGACTCACA
TCGTGATTCGAGACACATTCAGGACACTTCACCTTCATCCGGAGCGCTGGGATGAGAGTTTGTTCCTCGATCATAACATACGAGTGTGCGTGAAGAC
GATCCTTGCCTACTTAACTCACCCCATATTCCAGACACATAAAGATTATCTCAGTCTGCGGTATTCTACCCTGAGATACGGAAGAGTACTGTCCGTTAC

TAACCGGATGATCGCGCTGATACGACGAGAGGTGAGGTTGATCTTCAGCAAAGCAGATCACCCGTTCTGGACAGAGAGAACAGCC

> Cb_clean_k141_3378

CTTAACTACAGAAAAGAGCAACGGATTGGGGGTTTCTATCGGATTAATTTTGAACCAAGTCTGCGATTACTCTAAAACTGCTCCACACGAAACTTCG
GCCCTCGGCGACCAAAATCTTGAACCTTCAACGTTGACTCTAGCCTGTGAAATCAAACTCAAGAATCATGAACTTCGAAACCGAGTTTGACGATCTC
GTCTCAGCGAGCCGAGTGTTTTTCCTTGACACTAACCTGAGCAGTCCAGTTCTGTTGACCGAAATTGAAGACACACGCAAACTCATCCATGAGTGC
ATCCTGAAAACGACAAACAAGTCAGAATACTATCGAACTGTTAAATTCCATCGTCAGAATTCAGTAGAATTTCTCATCAAGCTGTGTAAGCTTCGGG
ATTGGAACGTCACCTTGCTTAAACGGTTTGGTCCACTCCAACCGACAGAGTATGGAAAGATTGAGCGGCACATGCTCAATCCACCTGACTCTAGATA
CACGAGCTATTACGACGAGGCTAAAGAGGCTGCTCAAGAGCTGGAGAGCGCTCTCGTTGCTGGACTCCGAGCCAGAGGGCTACCGGAAGACGAAA
TCAAAGGGACAACAGAGATGTTAGGAGGAAACTCGTGTCTCCCATACAGGAGAGCCTATGAAGAATTCTGTAACTTGTTCGCAAGAGCGGAAAGTC
GTGTTAAAAACTCACCTCACACGTGGACGCTAGGAACAGTAGGAGCTGTAAGATTCTGGATCACAAGCACAAACGCTCTGATAGAGTTTGACTCTC
AGATGTATTACTCAAGTCTAAATCAGGTTCTGATGCTCAAAGATAAAGTTGCAACTCGGTACATGATGCTCGAGCATGTAGGTCCCCTCGGTCTTTCG
CTGGCTCTCGAGAGTCATCTGCTCAAGCTATATAAATGGCAGGATTGTACACTCGAGTACTACGGGAACCAGGCGTACAATCTCCTGAAGGCTGTTG
AGCCTATGTTCAAGACCTGGTTGTCTCACAATGTTGACAACATATTCGGAAGTGATACTGCTTACACACGGATGAAGGCAAAGATGACAGACAAGG
AGGAGTCGGTAGCCAAGACGACACATCTCAGTAACCGAAATCTGATGTCAGGGCTATACACTATCGTCGAAGAGGTCCATGAAGTTCGCTCGATCGT
CGAACTATTTGGGTGTCTCAAGACCAGTGGACATCCTCTAATTGATACAGAACTTGGAGGACTGTCAGCAGCTGAGGCAGCCAGGTCTGACGATAA
GACATCCGTGGTTGACTCTCAGCGTCTGCGTAACACATTTTGTCACATCGTCCTAACTGCTTACATCGCGAAGCACGGATCTTGGCCCAAATTATATC
ACGCGAACTCCCAAACCAGTCTGAAAAAGTTGAACGACAGGCAATATCGCAACCTAACTCGGTCGAGTTACCCCCTTTCTGACTGGAATACAACTG
AGTGGACAAAACTGTTTGATTTTGATTACTTCCCAAATTTCCTGGAACTCATGGACGACAAGTCTATATCCTTGTATCGATCCGACAAACACCTCACA
TGGGATAAAGGACCTCGACCGAAATCGAACCGTCGTCTTCTCCTTGAGTGTCTTAAAAGAAAGACGGTGGACATAAAGGCTATAGTCGATAGGGTG
AGTGCTCGAGATGTTCCCAATGACTGGAAAATCGTGAGTTTGTATCCTAAGGAGAGGGAATTCAAGCTTGAACCTCGAATGTTTGCGATGTTGGTTC
TCGAGATGAGGTGCTTCTTTACCGCAATTGAAGCTAACATAGCTGAAAATGTGTTCAGGTACTTGCCGCAGCAGACAATGACGAAGTCCAAGACCC
AAAACCAAGAGAGGTTTCTCAAGTTCACAGATCCTTTAAGATCTCGAGCTGACTACACCTTGTTTTTGGAAATTGATCTGACAAGGTGGAACCTCAA
ATGGCGAGAATTGAGTATCCACGCAATAGGACACGATCTCAATAGAATGTTTGGCATGAAGGGGACATTCACGGTGACACACTGGTTCTTTGCTGCA
GCTCAGATTGTGGTACGGGTCGGAGGCTTACGTCCAGAAGGAATCGAGCAAGACAACCCTCCTATCTCCAACTTGGCATGGCGCGATCACAAAGGG
GGGTTTGAAGGACTCAACCAAAAACTCTGGACAGCTGCCACATACGCTATGGTCGAGATGGCACTGAGCCCGTTGCTAGAGGCTAAAACCATCAGT

GACTATGAAGTTATAGGTCAGGGAGATAACCAAGTTGTTCGGGTGTCCATTCCCGCTAGCGGTCGTTGCCGGGAAGATGTAATTCCAGAAGTTCGAG
ATCTCCTCAACGAAGCACTCGAGAGGACGTGTGCAAGTGTGAACCAAGAGGTGAAACCAGAGGAAAACATCGAATCCACATCTGTTCTTACTTATT
CGAAGGATGTTTTGGTCCGAGGGGTGGAGTACCCTACCTCTCTCAAGAAACACAGCAGGTTGTTCCCTGTCACATCCATGGACTTTCCTTCCGTGAT
CTCAAACACGCGGGCTATTCTAGCAGGGGCTATTGCTGGAGGAGAGAACGCACTGTACCCCTTACGAAGCGCAATTATCGGACACTATCACGCTTAT
CGCTATTTGAAAGCTGCATCCTCCGGGTACTCAATTCACGCTAAAGCATACCCTAAATTAAATCACATAGACATACAGACAGCTTTAATCATCCCGTCT
TCACTCGGTGGGCTTTGTGGACCAGCGTACGCATCTTTCTTCTACAAAGGAGGATCGGATCCTCTAGGTAAAGAGATCAGTGGAGTTAAATTCTTAG
CCGACGGAGATACGTGCCTAGGCGTGATAGCCTCTCGGACATTGGCATCGCTGGAACACAAGTACTACATCTCCGAGGCTCCAAACATAGCAACTCT
CATCGACAACCCTTATGGCTTGCCTCTTGAAACAGCGGTCTCACCACTGAGCAAAGTAGGGGCACTGACGTTGGATGCTTTTCGAGGAGTGGTAGA
CAACCGAGACATCGCCCCATTACTACGAACCTCCGTCGACAAAGCTGAGCTGACACTCAAGGAGGACTTACTGAAGATTCGACCCTTCAACCCTAT
TCTCATACACGACCTATTTGAGGCGTCTGGGTTCGGGTCTATAAAACTTATGAAGAAGATGTTCGTGCACACACGAACTATTCAGACGGTGGCTCAA
CAGAACAACGGCCAAATAACTCACACATTTTTGAGAGCAGACGTGAATGAGATTATGTCATACAAAAGATGGACGAAAGGGCTACTAGACCTCCGG
TACTCTGGGAAATCATCTTTTGAGTTGTGTACCCTGTACAGATCTTACTGGGCAGTCGACCTTAAAGGAGTGACAAATGAACAACCACTGGACTACA
CTCATTGGAAAGCTCCCGACAAGAAGCCAAGCTCGATACACTGGTCGTCTCACTCCGCGGATAACCTACTCGATACAAGAGGACCACTGACAGGAT
ATATAGGGACTGCCACCCGAGAGAAAAGGTCGGAGCACGGATACAAGATTGTGGACACCGGAGCCCCGTCTAGATCAGTTATGAAGCTTCAACTGA
TCCGTAGCCAAGCATACGGAAATCCGTCGTTCAACAACTTGTTAGACCGAATATCTCTCACTAGATCTCCAGTCCCTCTTTCCACAATATCCGATGTAT
TACCGAAGGTTGTCGGGGGATCAATCTCGCACCGATATGCATCATCAATCAGGTCCATGGGAGCCTCTTACGTTGGGCCCTTAAACTTTGCAACTCAT
ACCCGAGTCGACACAGACAACATCTCTAAATTGAGTGGTTCCGTTGTCAACTACCCTGTGATGCTTCAAGAATTTATCATCATGGCTCAAGCTGGAG
CAAAGATGATGCACTTGCACTTCCGGTCGTCAGCAGGTGGATTGTATCTTCCATTTGATTCACTGATACCTTTGGACGATGACTCGCTATCGTGTGAC
CCCCCTGATTTTGAACCGAAGCTCTTACCTCGCAGTACCCTGTCGTATAGTCCCACCTTAGCACTGAAACGCACTCTAGAAACAGATTTAGGTAGCAT
ACCGCGAGGGTCGTTGATCCCGCCTATGGACTATCAGAGCTTTCCGATAGTGTGGCTCAGTGTCGTGAATTTCTTCACATCAACTCTTCGAGATTCAA
ATCGAGCGAAGACCATAGCAGACACGCGAGGTCATATTTCACTACCGTCCAGCTTCCAGATCGACATTGCCGAGGCACATGCCTTAGGAGCAATGAG
GCTGGGAAGAGCAATCGCGCATGCTGTGACTCACATCGTGATTCGAGACACATTCAGGACACTTCACCTTCATCCGGAGCGCTGGGATGAGAGTTT
GTTCCTCGATCATAACATACGAGTGTGCGTGAAGACGATCCTTGCCTACTTAACTCACCCCATATTCCAGACACATAAAGATTATCTCAGTCTGCGGTA
TTCTACCCTGAGATACGGAAGAGCACTGTCCGTTACTAACCGGATGATCGCGCTGATACGACGAGAGGTGAGGTTGATCTTCAGCAAAGCGGATCAC
CCATTCTGGACAGAGAGAACAGCTGTGTTTTCAGGAGAAGGGTCGTCAGCATTAACGGAGGCGTTGGTGGTACATGCAGCAATTAGACTCAGGTGG
CTATATGCGATAGGTCATCCGAAGGCCTCCTTGTACTCACGCACATTTAACGGCTTCACTATAAATCCGAGGGGAGTGACTTTGACCCCACAGGAGA

GTTTGAACCGCACCGCTTTACAGCTAACACGCCTCGCCAAGTTCTACCGAACGGACGGGGATGAGCTGTTGTCTGGATTTATGATGAAGTTGTCGTC
TTTCCAAGGACTAGCGATATTCAACGACGACCAGCGAACTGTCATGAGATTTTCCCGGGCCATCTCACCGACGCTGTACTGCGTACGGAATGTCAAG
CCGACCAGTGTGGTCAAAACTCTTGAATCTGCCCCAACACACTGCTTGAACTGTTGTCCGCCCAGCTCGCACAAGCATACTACTATGTGGTCACGCT
ACAAGACCAGGAAGCACGGTGGGGTGAGCTCAGCGGGGTACACATGGCTCCCCATCGTGTCTACATTAGAGATCCAGCCCACCTGCTTTGTCATTG
GTAATGGAAACGGAGGGTTGGCAGATCTGCTCCTCACCAGTTATAACGTGGAAGTAGTAGGGCTTGATCTTGAGTCTGATATGCCTACCAATTCTGCA
ACACTCCTTAACTACTTTCCTCTCGGAATTTCGGTGAAAAATAAAACTAGATTCAGACAATCAGATGTGTCAATCAACACCACAGGAAATTGGCTAG
AAAAGTCAGTAAGGACCCTTCTGATGACTGAAACCAATACTACATCAACTGTCATTGTGGACATCACAGGTCCCGCTGCTATCGACGTTCTCAGTTC
CACACTGGATGCTATGGACACCAGGTCTGTGGTATCTGCATACTGCAGGCTTATAGGCTCGAGAGATGACATCTCTGGATGTATACGCTCTCTTCCTC
GCGTCGTAATGTCTCGTCTGTGGGTAGTTTCACACTCAACGTTTGAAATTGAGATAATCCTTGAACTATCTCGTACGAACAGCAGCGCTCACCTGTGC
ACAGGTGCTGGTCCTCTCATAGACTATCCTCTTGATCGCGCGCACCACGAGTTGATTCCCCAGAGACGAGACGAACTGTTGGAAGCGGCTACTCGAT
CAGTCCACGCGTGGACTGATGAGACGTTTCCGAGCATGTATGAAATCACTCGATCACTATGCAAAAGTCTGCTCAATAAACCGAAGTCTACACAGTT
AAGATACACTGAGCGCTATGACCTGATATTAGCGTACTGTGTGTTGACAGTGCGAGTTTCCTCCTCACCAGTGGAACTGGTGCAGTCGTGGATACTA
GACGAGAGAGCTGAGACAGATCTATTTACTACTGTCTGAACGAGTCAACTATATCACATCTCATCAGATACTCTGCCAGACTGCTGACGTGGTCAG
ATACCTCGATGACCGTGTGAATCTCCTCCCCTCACTGGAAACTTAGGCAAAGTCATCACTTACCCAAGTGCTTGATGCAACAGTTCCAACAGTTTTA
AGTAAAACCGTGCCGAAAGCAAAGCTTAAATCTTCTTTCATCCGACCGAAACGCATTCCCCACCATTTTATTCATCTAGTTAGTT

> Cb_clean_k141_36621
AGTCGAGACAATCGTGAAACAGGTCAGCACAAGAAACATTCCGGAAGATTGGAAAATTGTTAGTTTGTACCCGAAGGAAAGAGAATTCAAACTTG
AACCTAGGATGTTTGCCATGTTGGTGCTTGAAATGAGATGTTTCTTCACTGCCGTTGAGGCAAACATCGCATCTAACATATTCAAGTACCTCCCTCAG
CAGACGATGACAAAAACCAAGACACAGAATCAGGAGAGGTTCCTGCGATTTACAGATCCGTCAAAGAACAAGACAGACTATACCCTCTTCTTGGAG
ATCGACCTGACCCGCTGGAACCTGA

> Cb_clean_k141_39090
CTACCTGATCCAACTGACAGAATCGACCACTGTGAACAGTGTGTTCCATCCGAGCAATCAGTCAATATGATCATCTGGGATCGGAATAAGCAGAGAA
TAAACGGAGGACTGAGCTCTGCTGGGTATACGTGGATACATATCCTCCCTTACCTAACTCTCACTTCGTCAACACTCATTGTCGGGAATGGAAATGGT
GGTCTGGCGAATCTCCTGCTCAACTGCTTCGACACCCAAGTGATAGGCATAGATCTAGAGTCGGACATGCCCAGTCAAGTCGCCACCTTGCTCAATT

```
ACATCCCCCCTGGAGTCGAATTACACAACCGAGACCGTTTTATCCAGTCAGATTGGTCAATAAATTCATCAGGAAATTGGTTATCTGGTGATGTTCGA
GCGGGCGTGCTGAATTCTCTTCCTGGTGTCTCAACAATCTTCCTCGATATGACAGGTCCATCGGTTGGTGATCTTGTTCCGACAATCGAGGACGCAAT
GGACCATTCTCTGGTGGAGTCCGTCTGCGGCAGACTAATCGGTCGAACTGAGGAGATATTTGATGTAGTCAGATACTTAAGACAGTCGCATGAGGTC
CAGGCGTGGGTTTTCTCACGGTCAACAGCGAGTACTGAAGTCCTAATTTCCATCACACGCACTCGTCGGTGTCTTCATGAGTGCAATGGAGCGACA
GCGCTCATACACAAAGAGGTCAGTGCACGCCAGCACGCATTGATCCCTGAATTGTGGGACGAATTATTAGAAGCTGCTACGCTGTCGTCAGTACGAT
GGGATGGAGAGTCGACTCACGAAGCCTTCAACTATATGAGGAACCTCTGCAAAAGTCTACTAAACAAACCGAAATCGTCGCAGCTGAAGTACCGTG
ACAGGTACTCACTCCTGATAGGCTACTCTGTTCTGTTTGCGGTGAACTCAGAGATGCCTGTGCAAACCATACAAGGATGGATTGCAGAAGAAGAGAT
TGACACCGATCTGTACACCTACAAGCTTAATAGAGCTACAGTCACTCATCTCCTTCGGTACGTC

> Cb_clean_k141_44574
CAGCGGTCGAATCTTGATGAGATCCTCTCGCAAGGTCTTCTCTGCATCAGTCACAGTTGCTCGAAGTAGACTGTTGATATCAGTGTTGGACACAGAG
CCCCTGAATGCTTCCAGAGTCAAGTGTCCGACTCTACTGAGGGCAGACGAGGGAGTTTCCAGAGGAAGACCATACGGATTGTCGAGTAAAATCTCG
AGATTTGGTTCTTCAGACATCCAGTACTTGCATTCCACAGCTTTCAGACTTGACGAAGCAATGGATCCAATTGTTCCTTCTGATTCAGCCAACAGTCT
TAATCCGCTTATCTCCTTTCCGAGCGGGTCAGATCCCCCCTTGTAGAAGTAAGACGCAAAGGCAGGACCGCATAAGCCTCCAATACTCGAA

> Cb_clean_k141_57732
CACGCAGTAGGACCATCGTTCATTCTCAGGGCGATCTCAACGTCTATCATACTATCAACAGTTCGGGATACTTTCCGAACACTGCAACTTCATCCAGA
ACGATGGGACGAAAGTTTATTCCTGGCTCACAATATCAAGATCTGTCTCAAGGCTTGTGGAGCATACTTCGGGCATCCGCTGTTCAACACTCACAGA
GAGGCATATAGGTATCGTGCTTCAAGCCTTCGATATGCAGGAAGCATGAGTCTCGAACAGAGACTGACCGCAGCTGTACGGCGAGAAATCGCAACA
TCGTTCGGAACGGCTGGACACTCTGTGTGGAGACAAGAGATTCCGGTGTTCTCCGGCGATCACAGCTCAGCATTGGTTGAGGCTGTGACACTCTCA
GCTGCTCTTCGTATCCGTGAACTGTTTATGGCCGGTGATGAAAATGCAGTGTTATTTGCGAATCTCTTCTCTAGCTACATGCGAATTCCACGTGCAATA
GCGAACACTACCGGACAGACTCTTGAGATAGTGCGTCTTCGTTGTAC

> Cb_clean_k141_64068
ATCATTTCAGAGTACGAAGTCATCGGTCAGGGCGATAATCAGGTTGTCCGAGTCTCCATCCCAAACTCAGGGCGAACCAGAGAAGATATCGTTCCGG
AGGTCCGTGATATGCTCAACGATGCTCTCGAGAGGACATGTGCCAGTGTGAATCAGGAAGTTAAACCTGAAGAGAATATTGAGTCCACAGCTGTGC
```

TTACTTACTCCAAAGACGTTCTCGTCAGGGGAGTGGAGTATCCAACCTCCCTGAAGAAACACAGCCGACTTTTCCCGGTCACTTCTATGGATTTCCC
GTCGGTCGTTTCGAACGCTCGAGCTATATTAGCTGGGGCAACGGCAGGCGGAGAGAATGCCCTGTACCCCTTGAGAAGCGCAATCATAGGACACTA
CCATGCGTACCGGTACCTTCTTGCTGCAGGG

> Cb_clean_k141_25946
AAGAGCAACGGATTGGGGGTTTCTATCGGATTAATTTTGAACCAAGTCTGCGATTTCTCTAAAACTGTTTCACACGAAACTTCGGCCCTCGGCGACC
AAAATCTTGAACCTCCAACGTTGACCCTAGCCTGTGGAATCAAACTCAAGAGTCATGAACTTCGAAACCGAGTTTGACGATCTCGTCTCAGCGAGC
CGAGTGTTTTTCCTTGACACTAACCTGAGCAGTCCAGTTCTGTTGACCGAAATTGAAGACACACGCAAACTCATCCGTGAGTGCATCCTGAAAACG
ACAAACAAGTCAGAATACTATCGAACGGTTAAATTCCATCGTCAGAATTCAGTAGAATTTCTCATCAAGTTGTGTAAGCTTCGGGATTGGAACGTCA
CATTGCTTAAACGGTTTGGTCCACTCCAACCGACAGAGTATGGAAAGATTGAACGGCACATGCTCAATCCACCTGACGCTAGATATACGAGCTATTAC
GACGAGGCTAAAGAGGCTGCTCAAGAGCTGGAGAGCGCTCTCGTTGCTGGACTCCGAGCCAGAGGGCTACCGGAAGATGAAATCAAAGGGACAA
CAGAGATGTTAGGAGGAAACTCGTGTCTCCCATACAGGAGAGCCTATGAAGAGTTCTGTAACTTGTTCGCAAGAGCGGAAAGTCGTGTTAAAAACT
CACCTCACACGTGGACGCTAGGAACAGTAGGAGCTGTAAGGTTCTGGATCACGAGCACAAACGCTCTGATAGAGTTTGACTCTCAGATGTATTACTC
AAGTCTAAATCAGGTTCTGATGCTCAAAGATAAAGTTGCAACTCGGTACATGATGCTCGAGCATGTAGGTCCACTCGGTCTTTCGCTGGCTCTCGAG
AGCCATCTGCTCAAGCTATATAAATGGCAGGATTGTACACTCGAGTACTACGGGAACCAGGCGTACAACCTCCTGAAGGCTGTCGAGCCTATGTTCA
AGACCTGGTTGTCTCACAATGTTGACAACATATTCGGAAGTGATACTGCTTACACACGGATGAAGGCAAAGATGACAGACAAGGAGGAGTCGGTAG
CCAAGACGACGCATCTCAGTAACCGGAACCTGATGTCAGGGCTATACACTATCGTCGAAGAGGTCCATGAAGTTCGCTCGATCGTCGAACTGTTTGG
GTGTCTCAAGACCAGTGGACATCCTCTAATTGATACAGAACTTGGAGGACTGTCAGCAGCTGAGGCAGCCAGGTCTGACGATAAGACATCCGTGGT
GGACTCTCAGCGTCTGCGTAACACATTTTGTCACATCGTCCTAACTGCTTACATCGCGAAGCACGGATCTTGGCCCAAATTATATCACGCGAACTCCC
AAACCAGTCTGAAAAAGTTGAACGACAGGCAATACCGCAACCTAACTCGGTCGAGTTACCCTCTTTCTGACTGGAATACCACTGAGTGGACAAAAC
TGTTTGATTTTGATTACTTCCCCAATTTCCTGGAACTCATGGACGATAAGTCTATATCCTTGTATCGATCCGACAAACACCTCACATGGGATAAAGGAC
CTCGACCGAAATCGAACCGTCGTCTTCTCCTTGAGTGTCTTAAAAGAAAGACGGTGGACATAAAGGCTATAGTCGATAGGGTGAGTGCTCGAGATGT
CCCCAATGACTGGAAAATCGTGAGTTTGTATCCTAAGGAGAGGGAATTCAAGCTTGAACCTCGAATGTTTGCGATGTTGGTTCTCGAGATGAGGTGC
TTCTTTACTGCAATTGAAGCTAACATAGCTGAAAATGTGTTCAGGTACTTGCCGCAGCAGACAATGACGAAGTCTAAGACCCAAAACCAAGAAAGG
TTTCTCAAGTTCACAGATCCTTTAAGATCTCGAGCTGACTACACCTTGTTTTTGGAAATTGATCTGACAAGGTGGAACCTCAAATGGCGAGAATTGA
GTATCCACGCAATAGGACACGATCTCAATAGAATGTTTGGCATGAAGGGGACATTCACGGTGACACACTGGTTTTTTGCTGCAGCTCAGATTGTGGT

ACGGGTCGGAGGCTTACGTCCAGAAGGAATCGAGCAAGACAACCCTCCTATCTCCAACTTGGCATGGCGCGACCACAAAGGGGGGTTTGAAGGAC
TCAACCAGAAACTTTGGACAGCTGCCACATACGCTATGGTCGAGATGGCGTTGAGCCCGTTGCTAGAGGCTAAAAACATCAGTGACTATGAAGTTAT
AGGTCAGGGAGATAACCAAGTCGTTCGAGTGTCCATTCCCGCTAACGGTCGTTGCCGGGAAGATGTAATTCCAGAAGTTCGAGATCTCCTCAACGA
AGCACTCGAGAGGACGTGTGCAAGTGTGAATCAAGAGGTGAAACCAGAGGAAAACATCGAATCCACATCTGTTCTTACTTATTCGAAGGACGTTTT
GGTCCGAGGAGTGGAGTACCCCACCTCTCTCAAGAAACACAGCAGGTTGTTCCCTGTCACATCCATGGACTTTCCTTCCGTGATCTCAAACACGCG
GGCTATTCTAGCAGGGGCTATTGCTGGAGGAGAGAACGCACTGTACCCATTGCGAAGCGCAATTATCGGACACTATCACGCTTATCGCTATTTGAGAG
CTGCATCCTCCGGGTACTCGATTCACGCTAAAGCATACCCTAAATTAAATCACATAGACATACAAACAGCTTTAATCATCCCGTCTTCACTCGGTGGG
CTTTGTGGACCGGCGTACGCATCTTTCTTCTACAAGGGAGGATCGGATCCTCTAGGTAAAGAGATCAGTGGAGTTAAGTTCTTAGCCGACGGAGATA
CGTGCCTAGGCGTGATAGCCTCTCGGACGTTGGCATCGCTGGAACACAAGTACCACGTCTCCGAGGCTCCAAATATAGCAACTCTCATCGACAACCC
TTATGGCTTGCCTCTTGAAACGGCGGTCTCACCCCTGAGCAAGGTAGGGGCACTGACGTTGGATGCTTTTCGAGGAGTGGTAGACAACCGAGACAT
CGCCCCATTACTACGAACCTCCGTCGACAAAGCTGAGTTGACGCTCAAGGAGGACTTACTGAAGATTCGACCCTTCAACCCCATTCTCATACACGAC
CTATTTGAGGCATCTGGGTTCGGGTCGATAAAACTTATGAAGAAGATGTTCGTGCACACACGAACTATTCAGACGGTGGCTCAACAGAACAA

> Cb_clean_k141_11227
AACGTTGACCCTAGTCTGTGGAATCAAACTCAAGAGTCATGAACTTCGAAACCGAGTTTGACGATCTCGTCTCAGCGAGCCGAGTGTTTTTCCTTGA
CACTAACCTGAGCAGTCCAGTTCTGTTGACCGAAATTGAAGACACACGCAAACTCATCCATGAGTGCATCCTGAAAACGACAAACAAGTCAGAATA
CTATCGAACGGTTAAATTCCATCGTCAGAATTCAGTAGAATTTCTCATCAAGTTGTGTAAGCTTCGGGATTGGAACGTCACCTTGCTTAAACGGTTTG
GTCCACTCCAACCGACAGAGTATGGAAAGATTGAACGGCACATGCTCAATCCACCTGAC

> Cb_clean_k141_32680
CCTCCGTCGACAAAGCTGAGTTGACGCTCAAGGAGGACTTACTGAAGATTCGACCCATTACTACGAACCTCCGTCGACAAAGCTGAGTTGACGCTC
AAGGAGGACTTACTGAAGATTCGACCCTTCAACCCCATTCTCATACACGACCTATTTGAGGCATCTGGGTTCGGGTCGATAAAACTTATGAAGAAGA
TGTTCGTGCACACACGAACTATTCAGACGGTGGCTCAACAGAACAACGGCCAAATAACTCACACATTTTTGAGAGCAGACGTGAATGAGATTATGT
CATACAAAAGATGGACGAAAGGGCTACTAGACCTCCGGTACTCTGGAAAATCATCTTTTGAGTTGTGTACCCTGTATAGATCTTACTGGGCAGTCGA
CCTGAAGGGAGTGACAAATGAGCAACCACTGGACTA

> Cb_clean_k141_5086
CCTCCGTCGACAAAGCTGAGTTGACGCTCAAGGAGGACTTACTGAAGATTCGACCCATTACTACGAACCTCCGTCGACAAAGCTGAGTTGACGCTC
AAGGAGGACTTACTGAAGATTCGACCCTTCAACCCCATTCTCATACACGACCTATTTGAGGCATCTGGGTTCGGGTCGATAAAACTTATGAAGAAGA
TGTTCGTGCACACACGAACTATTCAGACGGTGGCTCAACAGAACAACGGCCAAATAACTCACACATTTTTGAGAGCAGACGTGAATGAGATTATGT
CATACAAAAGATGGACGAAAGGGCTACTAGACCTCCGGTACTCTGGAAAATCATCTTTTGAGTTGTGTACCCTGTATAGATCTTACTGGGCAGTCGA
CCTGAAGGGAGTGACAAATGAGCAACCACTGGACTA

> Cb_clean_contig_154
GCCGTGTTCAGGCTGTGCAGGATCTGCTAGAATGGGCATTGAGTCTGGTCGGTCGTCATTCGAAGGTCGAGATCGATTTGTCTTGTTGCTCTTGTTAC
TCTTTTGGCCACAATACACTTTTCAAATTCTCATTATAATCATAAAGAAATGTCTTACTCAGTACAAAGGTCCATCACAACCAGATACCAAGCAACCTC
AGTTGTTTCTACAAACATTAGTAAGAGGTTAAATGTCTCAAGTGACCTTGAAAGATTTGCATCTGGGATGAGTGATGAATACATTCCCAGGACACTTT
CAGAGTACAAACAGTACAGAACCTTTGCACATGACATTCTCTGTTTGGGTTTAATTTCAGGGAATTTTTATGATGATGAGTCATTCACAATAGACAAG
ATCAACTTAAAGAGTAATGATCCCAAGATTATCACACAGAAACCAGACCTATACTCTATAGAAGGAAATTTACTGAAGCTATGTGAGGTGTCTCTCAC
GTATGATTTGGAGACCACTGAGAACAATAAGAACTCAAAATATGATGACTTAATCGAATTCATAGAGAGATCAACTGATCTGAATGTCAATTACACCA
CATTATTAATTGATTTGACTGACGCAGAATGGACAGATAGTATCCCCTCAATACCTGAAAATCACAAAGTTCTCTTGGAGAGATTCATTGACAATTTG
GTCAAAATTCACTCAACCAAGCTAGGAATCGAATTCAGAAAAACACTCTCTCTTAATGTTAACATATCTTTCCCATTTAGCTACAATGAATCAGACTT
GTATAATCAAATAAAATCACATCTCAACATAACGGAAGATCTTAAAGAAGATTTTGATATTTACACAAGAGGGTTCGGAAACTTAAATTTGGAGTCTT
CAGAAGTTAAAAATTATCTAGATAAAGTGACAGACACAATATTGACAATGGACAAACATGACAGGCCTTTTCCTTGCAATCATCGAATAGATCCAATT
ACATACAGAGGTGAATGGGACGAATTTCTTTCAAAACCTTCCACCACTGAGAAGATACCAGTAGTCTTACAGCTTGGGATGCCAAACTTAACAGAC
GAGATTCAGACTTTATCTAAGACAGAGATTTATCAAAATTTCATGGACGACAAGAAGCACTATGGCGGCTATGTTGATCTGATAAAATCGTCAAACAC
CTCTGTCGACGAATATTTAGAGAATGGAATCATGAAGTTATTCCTAACAGAATCTCAACTTGCCTCTGAAATGATGTCTGGACCAGGCAGGAAAAAG
TATCTAAAGCAAAATTCCATAAAGTTAGATAGAAAGGCACCAACACATTTGAAACCAGACATTTCCCATGAATCACTGTTATCAGAATTGACAGATCA
AATAAATGAAATAATGCCTCAAGAGTTCCTGAAAACTTACGGAAGAGCAGATTACAATGTTTGTGGAAATAATTTAGGATCATCTGTCAATTATTGCT
TAAACAAGCTATCTGAAAATGGTACTGATTTTCTTCTTCAATTTTACACAAGATTATCAACTGAAGTTATATTAAACTCAATGAGGCGTAGAAAACAA
CGAGAATACGTTCTTGGACACAGCGGTTTTGAAGGCGTTTACTTTCTCATAGCACCAGGCCCTCAATTGAGGACTGAAGCCAACGTTGAATTCATTA
AAATCATTAGCTTTGTAGAACCCATTTATAATAAATTATCAAGAGAGTGGCATCTAATTGGAGATCACTGGGAGTCTAGCTGGCTTTCTGTAGACACG

GATAGGTTAAAACACTGGTCTCGTAGCAGGCATCGTGTAAATCTATCTCTGATCGGGTCTGCAGACAAACTATTGAGACCCGGAGTGACTCTTACTA
ATGCATTGTCCGAAGAAATCAACAACTCGAATTACACACTCATGAGTCTCATTTACCTTGAAAACAAGTCCAATACTTCTACAACCATTCAGACAACT
AGATATTTCTTGATGAAGTGTCTCGGCGACAGAAACTTGAAAGGACTGGTCTCAAAATTCCCAAGCAGAGTTAATTCAATATTACAGTCCGTTATACT
ACAACGAAACAACTCCTTTGTTTTGGACATGTGCTCTCACAAAGTGGATGATTTTGTTAAACTTCAGCATGTAAAGCGAGATGAGAACATAGGACAT
TTGGATGAAACAACGACCGGTGTTGTTGGAGCGATACCCAGGATATTCACAAAAGGAAATTATGTACCCATAAAATACTCTATTAATGAAATATACTG
GTGTATGATGTACAACAAAGATAGACAGAATCAAACTCAAGATGCTATGAAAATTCTGAACAAGATTGCCAAGGAAGAGAGAAAGTTTGACAAAGA
AATACAGTCTAGAAACACTGACAAGGAGAAACTAGCACATTGTTTTGGATTTCACACTACATCCCAAGATATACACCACATAACAACAAAAACACCC
GAATCACATTACTTTAGCGCTCTGGCCGTTCATATAGGTGTCGCATTACAAGATAGGCATCCCGAGAACTTCTCGCCTCAATCAGGCTGGATGTCTGA
CAATAAGCTCAATGAGATTTTATCAAAAAATCTGTCTGAATACGCCACATTCAAAGCATCTGTGAACACCATAAAAGATTATGTGAGAAATGATGATC
TTTCAGAGCTAAAGGAAATTGGCCGAAGGACAAAATGTATTGAGCTGATCTATGAGATTGTGAACAATGAAAAATTGACAACTGCTGCTGACATTGC
CATGAGCTTTTCAGGCAACAAATCAACTCAGTTTAAAACCGTCATACAAATATTCAAAAAGAATCAAATTGGTGGCGTGAGAGAGATCCTCATATTAT
ACATAAAAGCTAGAGTCCTCATAAATCTGTGTGAAGAAATATCAAGACTTCTTTCTAAGTCTGATAAACGTGAGACATTGACAAAGGGTCGTGACAA
AAGGCTCATGATGCGCGGTGATTACGAAGAACTAACTTCCAAATTTGAAGAAGGCACTCCATTATACATAGTAAAAAACTCTTACGATATGTCTACTT
GGTGTCAAAAATTCATCCCAACAATATTTCTCCCTATATATAATCATCATCATGCCTGTTTGGGTAATCTGTTATCCTTGTGCAGGTTTGTTTTGATTAAG
CATTGTCAGAAAGAAATTGAATACCCTCAGAAGCTCGTGACAGAATGGATAAAACATCCAGATGTTAAACATGATGAAGATCATATGCAATATTATAA
AGAAAAGTTTCAGTCAGATAGGCAACCGAAAATGCTAAACTTCAGCAACATGGGACAAGGAATTCTGCATTATAATTCAACAGTTTTGGCGCTGTCT
TGTCAGAGTTTGAGGGACAAGTTGTTTTACCTGTGTCTTGAAAAACTATCCAGGCCCATTGCGATAAGATGGAAGACTCGTGTAGGATCAGATGATA
AAGGAGACACAATCTTTGCAGATATGTCTAAAGAAGATTATATATTTCAATGTCAACTTCTAGAACAGTGCGCATCAGTTTCGGAGCGCCTGCATTCA
ATGGACTTATCAGTTAAATCTGCTTCGGGAAATGTTATTTACGAGTTTAACTCCGCTTATATGGCAAATCTTGAAGTACAGTCACCTGTCGTGAAATTC
ACCATGGCAGCGGTTGACATGATCGGAACAGATAGCTGTACAGAGTTTGTTAATGAATCTTATTCTAGAATAAGGCAGCTTCGCGAGAATGGTGCAA
ACTCCCTTCTATGTTACATCGCCCATATTTATAACAAAGATCATTTTGACGAAATCTTTAGAACAGGAGAATCAATGACGAATGATCCAACCAAAATAT
TCAACCTTCCACTATCTAAAATACCTTATGATCTTGGAAACTACCCAATATATGACTGTGATGTTCAAGATATGGTAGGTCCAGAATATCATAATTATAG
AATTTTCAACGATCCAGAAACACCAGAAAGAATCTTAAGGTTATTGTACACTTCTCATTTTGATGCTGATAAATCTAGCGTGATACCAGATGATGACG
AAGGCCTGTTTAAGAAGGATGATTTTAGGATAGCTCAAGGGATGGTCAGGCAGTTATCAAACATGAGAAATAGACTGAATCTGGATAGAGAATCTAT
ACATAAATTTTTAGAAGAAAACCCTTTTTTAATTGTCCGTGGCCCTATGACAGTCGAAGAAACAGCCATTCTAATTGCAGCAAAATTATACACCAGAG
GTGCATCTAAAAGCTTAAGAAGAACATCACCTGCAATCTATCTAGGCAGACTAGCTGCCTTTGAATCCGCGAGGGCTTGGAATCTGTTTCAACAAGA

TGGCGCAGAAGAAACAAAGGTGATAAAGCTTACGTTCTCAGAATATATAAAAACTCTATATGAGTCAATCAAAATAGAGCCTAACTCAGATTCAATA
AAGAAATTCAAACCACTCATATTCCCGCAATACAATTCTTTTGATGTGGTTACTCAATATGTTTCAAATTTTGGTATCAGGAAAGAGAGTCATAAATTG
TTTTCTCAATCAATAAGAACCTGGATCTTAAATAATTTCAACTATAATTTCACCAACAGCTTAAAAAGCATTCTCGAAACCTCTTTTAACATGCGCAAA
TCAGCAACCAGGGAAGATGTTATGGAACTAAGAAAAGTCGTGCCTTTTGACATCAGCAATTATGACACCTTTATAAACAATTGTCGAGAAAACAATG
TTAGGCCACTAGATTTATTTTATTATCTAACAAAGTTTTACAAGAACACCAATCTAAGAAGAGCACAAGTCTTTGCAGTAGGGCCTAGCACTAGTTCT
TTGAACCTGACATTGAGTAATCTCAAGAGATATAACCACCTAACTGGATCAATCATGGAACTTGACACAGGCATTGATATGCAAAGAATAGAGACAG
CCTCAACTTTAGATCGAGATTTCGAAATAATGAAATTATCTTTCAATTTCTTGATGCTAGAAACACAGGGGACACTAACGAACAAATATGGTTTAATTG
AACAAGGGATACTTAATGAAATCAGTCTTGATGGAGTTACATTTCACTCGCTAACAGAGACAATAATTCGGAATATAAATAGCATATCTGGATTTGACA
ATCACATTAAGCGAATCATAATCCTTCTTGCATCTAAAATATTAACAAAATCGGAGTTCTTGTCAAAAATATTGTCATGGAAACAGTTTAGCTTTAATTA
TCTAAAAAAGCAAAAGAGAGACATTCATGGAGATTGGCATGGAGATCTCTCTGTTCTAATTAACTATTCAAACGAATGTTTCACAATAAACCAGACA
AATAATTACAAATACATTGAAATCATGAGAGTTAACGATTTAAGAGACTTCCAGATTGCCTTCAGAAAATCATTACGATTACTTGGTTTCACTGAAAG
TGAGTTCACAACTAATGGACCAATAAAACCAACAGAGTATTACGTCTCAAACTCGAACGTTTACGAGAGCAGAGGATACTCTAAATTAAAACAAAG
ATTGAACATTCATAAGAACCCAAACTTCAAATTCTTAAAACTGACAGATTTGACGAATTTTAAAGTGAAATTTGAAGTAATAAAGACTGGTGCCACT
ATCATAAAACTAGTGGATCAACATTCGCTTAAGCACATTGATGTGGCTCATTTTCCTGGTCATTACTACCCTGTGGAAATTCCCTTAAAGTTCAAAATG
TCAGAAAACATATTCGTCAATGGTTTACGAGGTCTAAAACTATTCAAAAATAGAAAATGGTTTTTTGATGGAAGATTGTATTCATTTAACACAAAGGG
TGCAATTGACATATTACAAAATCATATTAATTTAGAACACTTGAAGGCTGTGAATATAAGTTCCAAAGCTAGACTTCAAGAATACATTAACGATATGGA
ATATGATCAAGAAGATTATGAGGGGAATTATGATGTTGAAGTGAATCCAATAACAGAAGTCCAACTCAGGATGGACCTGGTTAGACAGACTGATATC
ATGAATAATAACAGATCAATAAAAGATATGTTCCAAGATGCTGCAAACAAGCTCATGACAGAAGATTATACGAATTTAATGCCAGATGAGGACGTTCC
TTTGATTGATCAGTTTAAGGAGGATGCCGGTGTCTCTGATTTTATAAGAGCCTTAGGACAGAATAAGTACAGAAAGAAAAAAGATTTTTATATGGTCA
CAAACTTACGATTAAATACAACATTCGTAAACAGAGTTTTGGACCTCTTCTTCAAAGGAAACTCTATAATGTCAGAACAGCCCAATGACCTCCCTGA
CTATGGAATACACTGTTTATCTGTTAGAGATCAACCTGAAGCAGATTACAACCTCATTTCAATGTTGTTTAATTACATTTGTCTAAGAATCTCATCTTTA
ACTGGGAAAGACATGAATTATATACAAGTTGAGATATTAAAACTGAAGGATAGAAAGCATAATTTTAACACATTGCCAAGATTAAACAAGTATCTAAC
AAAATTCTCTCCAGGATTGTATGAATTACTTGATCAAAATGCATTTGATGGACAGGATGAGGAGTCTGAGGGAGAGTTTGATTTTTGAATATCATGGA
TAATGAAAGTTTGTTGTTTTGTTGGAAAAAATCTATCTTTTTAAC

> Cb_clean_k141_63059

ACAAAACTGAAACAAACCAAAACAAAATGGATCGAACCTCCATTGCCAAATCCATTGCCACCATCCAAGGAGCAGCAACCAAGGTTTTTGACATCA
TATCAGGTACATCTTCTTTTGATTATATGCCTGAAAACTCTGGCCAATATTCTCAATACAGAAGATGCATGCATGATGTGATTTGTGCGGCATTACTCGA
TTCCGGCCATTACAAGTATACTGAATTGACTCTATCAGACTTTGGTATCACCATCATGGACAAGAAGATCAAAATGCAAAAGCCTGATCTTTTCATGG
TTCAAGAAAAGGAAGTGTTAATCGGTGAAGTCACATGTACATACTCACCTGAGCAATCTATAAAAGAGAAAATAGGCAAGTATCATGAATTCATAAA
AATCCTTGAAAATAATGGATTCAATGTGACTTTCACTGTGCACACAGTGGACTTGACAAATCCTGATTGGTCTGAATCACTTCCTTTTATCAGCAAAC
AATATGTTGATATATTTGAAGATATGTTAACAGTACTCAGAAACATCCACACAAATCCCAAGTTTTCAGCCATACGGAAGTCTGAATCTGGATCTTATA
GTATTGACAGATTTCAATTCAAGCTTGAAGATGAGACACTAACAAGTCTAGTTGAGGAATGCACAACAATAAATATGGAACCATCTAAAATAAAGGC
TAAACTAAAGGGCACATCAAGATCTTCTGATCAATTTTATGTGGATCAGATTGCTCAGTCTATTCTGTCAAACAACTCTCATCCAAGACCACGACCAA
ACCCTAGATCATTTGAACCAACCATACTTATAAAAGACCTTGAACGTATGAAATCAGTTAAACCGACGACATCAAAGCTACCAAGAATTCTTCAATTA
GGTTGCCCCTCAAGAATTGTTCGCCACAAAATAAGTTTTGAAGATTCAATTCTTGAGTTGAAAAGCTCAGGATGCCATGGTGGTTATCTTGATTACAT
TCTGAGCTCTTTACAAGACGCAGAAGTCCCAAAGACGAGGATGATAAAGCTTGCTCTCCCCATCTCAAAGGTGGAATCTGAGCAGAAATCTGGACC
TGGTCGCAAATCATACATGAAGAAGCATGGTATCTCAACCCATCGTGAGGTTCCGACTCATATTGGTATCTCAGCAAACCATATAAAGGTTTTGGATG
ACTTCATCTCACAGATAAAGTCTGACATAGTTGATGTTGACTTGCCTCAAATAAAACAGCAAGATGATGAAACAGCTGGGTTGTCTGCATCCCTGAG
TCTTGATTTCATGGAGTCAAAATTTAGGACTGACAAGTCTACAGCAATCTCATACTTCTATCAAGTTTTATCAAACGAAATTGTTTTGAACAGCATGA
GGAGACGATCCACTAGAGAATATGCATTAGGTTACACATCAATCAGAGGTGTTTATTTTATAGTAGCCCCCGGACCACAGTTAAGGACTGAGTCAAAT
GTTGAATTTATAAAGATAATATCATTAGTTCCTGGTATATCAAATGGTCTCTCATCACCTTGGCACTCGGAGGGGGATCACTGGGAATCTGATTGGTTG
TCTGTGGACACAGATAGATTGAAACATTGGCAACGATCAAGAGATCGAACCATAATGTGTACAGTATCTAATGCAGAGCGTTTAGTAAGACCAGGTG
TTAAACTCCGTGATGCAATAAAGGTGGAGTTGGAGAGCAATAATCATGCATTAATGACATTAACATATCTTGAGAATAAACAGACAACATCCACAACT
AATCAGACAATCAGATATCTGTGGATGAAGTCATTAGGTGACAAGGATTTCAAAGATCTGCTAAAAAAGATGCCACAAAGAGTTAACTCAATTATCC
AATCCTCGATGCTTCAGAAAGCAGTCGAAACTTGCATCAGAATCTCAAAGACAGACCTGACTGAGCTTGTCACAGTTGGTAAGGTGATTAGAGATG
ATGATACTGGGAACTACGATGAGTCAACTACCGGTGTAGCTAACCTAATGCCACGACTTTTCACTTATGGTGGTCCTGTCCCTTTGTCATACAATCTCA
ATGAAATCTATTGGTGTATGGCTTATAACAAGGATAGACAGAACAAAACACAAGATGCTATGAGAATTCTCGATAAAATATTAAAGGAAGAGCAGAA
ATATGACAACGAAATAAATAGCAGACTAAAACCAGAAGCAAAAGTGAACTATTTGCTCGGAACAACGACAATACAAGATGATATAAATCACATACAT
ACAGACAAGCCTGAAAGCCACTATTTTAGTTACCGAGCCGTCCAAGTTGGCATCTCCTTACAAGATTCACATATAGAAAACAGGGGTGAACACGGAT
CTTGGCTCAACAATCAAAGACTCTCTGATATACTCTCAAAGAATCTGTCAGAATTTTCAACATTCAAAGCATCAGTCAAAAAGATCGAACGATCAAT
TGACCCAAATGATCTGAATGAAATAAAGAAAATAGGCCAAAGGACAAAGGCTATTGAACTTGTTGCAGAGATAGTGCAGGATGAGAAACTTCAAAC

ATCTATGGATGTGGCTATGACCTTCTCTGGGATAAACAACAGGAATTTTGAAATTTTAATACAAATATTCAAAAAGAATCAAATTGGTGGCGTTCGTG
AAATAATAATCCTCATAATAAAGGCAAGGATATTGTTTAATATAGTGGAGGAGGTTGCAAGACTCTTAAGCAAATCTGACTCAAGGGAGATCCTGACA
AAGGGGAAAGACAAACGATTGATGATGAGATCTGATTATGAAGCAGTTTTGTCCTCCTTTGATAAAGGAACACCAATCCAAATTGTTAAGGAATCAT
ATGACATGACCACATGGGCTCAAAAGTTCATACCAACAATCTTCATCCCTTTATTTGAGCACCACTTCCGGGAGTTTCCAGGCATGATTGATTTATCAC
GGCTTATTTTCTTATCACATTCGAATAAGAAAATGGAATATCCAAAAGGTTTGCTAGAACAATGGATTAAACATCCCACTATTAAACATGACTCTGAGG
CGGTCCAGTCGCATAAGGAAGAATTCATTAAAACTGGCAAACCTTATTTTGTCAACCACTCAAACATGTGTCAAGGGATACCTCATTATAACTCAACA
GTGCTGGCTCTTGCTTGTCTGAGTCTCCGTGATTCGTTGTTTAAATCATGTCTCAAGCAACTGAATGTTCAACAACACATACAATGGAAGACTCGAGT
AGGTTCCGATGATAAAGGAACTATAATTGGAATCGACAGATCAAAATCAGATTCATACTTCCAATATATTCTTCTTGGTCAATGTGAGAGGGCAGCAG
AGCGTCTCTTCTCCATGGAGCTTTCGGTGAAGTCAGCTAGTGGACATCTAATGTATGAGCTGAACTCAGCCTTCATGGCCAACCTGGAAACTTTATCA
CCAACAATAAAGTTCGCTTGTGCATCAACAGACACTATCGGCACATCTTCATGTACATCATTTATAAATGAGTCTTACTCGAGGATAAGACAAATGAG
AGAGAATGGATGTAGTTCAATTGTGTGTTCATTCGCACACTCACTAAATATGGACCATTTCTATAATATCTTTTCAACTGGTTTTGGTATGGAAAATGAT
GTATCAATGATCTTCAAGTGTCCCCCTAGTGAAATACCATATGATTTTGGAATCTACCCATTGTATGATGTTGACCTCCAAGATATTGTCGGCCCTGAGT
ATCATAATTATAGAATATTGAAACAAAACCCGAAATCAAATTTGTGCAAACTGCTCTACACTGAGATCAACTCCAGAAATAAAGATGAGTTATTTCCA
GCAGAAGATGCACCCCTATTAAAGAAGGATCACTTTGGTATACATCAAGGGCTCGTCAGACAGCTTGAGAACATGAAGACACGACTAAATGCGAAA
TCTGAAGATGTAGAAAAGTTCTTTGAAGAAAACCCGTTCTTAATGATTCGAGGTCCCGAAACGCTGGAGGAAACAAAATATTCCATCATCTCAAAGT
TGTTCACAAAGGGTGCTGCAGAATCACTCAGGAGAACCTCAGCAGCAATTTACATCGGCAGGTTGTCAGCTTTTAAGACATCTGAATCCTGGATAGC
ACCCTATGATATTCAAACAGTTTTTGATCTTGAAAGTGGAGAATCAGACATTAAAACAACATATATAAAAACAACATACTCGGAGTTTCTAAAAAGAT
CTCTAACAAAAGTCGAAAATTCTAAGAGCTCCATTGATTTAGAATCTATGAAGAGGATAATATTCCCACAATGGAAATCTTTTGATGTTGTATCGACAT
TTGTGGGTAGGTTTGGTGCAGTTAAGACTTCAACAAAGCATTACTCACAAGCAGTTAGGACATGGACAGTCAACAATTTTAACTATGAGTTCAGTTC
TAGCTTGAAATCAATAATAGAGACCAGCTTCGGCCTTTCTCAAGATGCGACAGAGGAGGACATTGCTGAATTCAAAAGAATGATGTCAATGAAACTC
TCAAGCATTGATCAGTTCAAAGATGAGTGTAAATCAAAAAACTTGAGACCACTGGATGTTTTCTATTACATGACAAAAATTTATAAGTCTTCTAGAAC
ATCCAAAATACAAACATTTGCCAACGGTCCAACAACTAGTGGTTTACATATGACAGCTCTAGCATTGAAGAGATATAATCATATGCCTGGTGCCTCAG
TCCAACTTGATGTTGGGATGGACGAGGATGACTTATACGAAGAAACCTCATTGAGCAATAAGCTTGACAAGATAAAATTATTTTACAACATATCAGTC
ATGAAACTCAATGATTTAATCACAGGGAATGTTTCTGATTTTGACATGGTGAGCGAAACTGGATCACTTAGTGACGAGTGTCGGACAATAATCCGGT
CTATAAGATCCCTATCAGGGTTCGATAACATAACTCAGAAGACACTGAAGATGGCAGCTTGCCAACTCCTTCAGAAGTCTGAATTGAAAGCGAAATT
ACTTTCATGGCGGAGCATAAATTATAGCTACATAAAGAAACAAATCAAACGGATCTTACCATCTGGGTCAGTCAAATGGTCCGGTGACCTATCAATAT

TAGTGAACTCAGAAGATGAGTGCTTCACTTTGAACGAGAGTAATGGGTCAAGATTCATTGTCGCAAGAGAGGTGAATGATTTGTCCTCAATATATAG
ATCACTCCGATCTATGTGTTCAATATTGGACTTTGAATTCACAACTATGTTTGTCAGGTGTAAATTGGATGTTGGTGATGTGTGCTTATCATCAAATTCC
AAATCTTTGATAAAATGTGAGATAAAGGGAGTTGAAGGGTTGAAATTGAAGATGAATCTGACACCAAACTTTCGATTTAGAAGATTGACTGACATGC
AATCTTTTGATGTTAAATTCACACACAACAAGAAAACAGGTGAAATAGAGGTGTATTTGACTGAAAATGGCATGAGGACTGCAACAATTTGTCACAG
CAACGGAAATTACTATCCAGTCGAAATACCACCTGGTTACCACCTGACACATAGGGTTGAATACCTGGGTGTCGACTTGGAGAGCATCTTCAGAAAT
AGGTCATGGTTCTATAACTTTCGTCTGCCAATGATGAACGAGAAAGAAAGACTTAGATTTTTAAATGAGTGTGTTGACTACGACATTATCTTATCTCA
GACAACAAAATCGATGCAGCGGATACATGATTATATGGAGGTCAGAGAAGAAGTTAATGAAGAATCATTTAGTGTGATCCGTGACCATATGCAGATG
ATCAGGACATATGATACAAATACATATGAAGGGGTGAATATGATGGAAGTTTTCAGGCAAACAATGGACTCAACAGACATGCCCACAAGTTTGACAA
AATTCTCAGAAAACTTGGAAGACTGGGCTCAAGACGTCCAGGATAGCCTCGATAATGAAATACAAAATTTAGTTAGTGAAGAAACACTTGAATTGA
CAAGAGGCCTTGGTTACAAAAAACCGGCACCAAAGCGTGCAATGGCGACCATCTCTGGATTGCAACAGGGGTCAATCCTCAAAGATAGAGTTCTTG
ATTGTTTCTTCCAATCCCAAAACATAAGAAACGAAAGTTCAAATCAGCTTCCACATATGTTTGTTTGGTTGGATCAACAATCTCTTGAGGCCACAAAA
TTTCCAGATTCTTTACTGACTGAACTTAAGAACCATATAATAAAATCAATTGTGATGTCATCTGGGGCAACCCCAAATTATATAAGAAATAAGATAACC
GAAAGTATGACCTATTCAATACCCATTCAGATGCTTTATAATTTGTTAAACTCCAGAACAATAACTACAACAAACATCATGGAAGAGATTCTGAATGA
GACCAATGATGATTACAATTCTGAAAGTGGTGATAGTGATCTAGATGTCTAGTGAAGAGGCTCTTAGTGAGGATTAGGGTGAAGTGAAGTCAAGGTT
TGTGTCTGGTT

> Cb_clean_k141_61155
GTGATTCGTTGTTTAAATCATGTCTCAAGCAACTGAATGTTCAACAACACATACAATGGAAGACTCGAGTAGGTTCCGATGATAAAGGAACTATAATT
GGAATCGACAGATCAAAATCAGATTCATACTTCCAATATATTCTTCTTGGTCAATGTGAGAGGGCAGCAGAACGTCTCTTCTCCATGGAGCTTTCGGT
GAAGTCAGCTAGTGGACATCTAATGTATGAGCTGAACTCAGCCTTCATGGCTAACCTGGAAACTTTATCACCAACAATAAAGTTCGCTTGTGCATCA
ACAGACACTATCGGCAC

> Cb_clean_k141_13335
CCCTGGGATGTCATCTTTTGCAAAATTCTTATTCTTGTCACATTGCAACAAGAAAATGGAGTACCCTAAAGGTTTAATTGAACAGTGGATCAAGCACC
CATCTACAAAACATGAATCAAAAAACATTCAGCAACACAAGGAGTCTTTCTTTGAAAATGGTGAGCCTTTTTTTGTCAATCATTCAAATATGTGCCAA
GGCATTCCTCATTACAATTCAACAGTCCTCGCACTCTCTTGTCTCAGTCTTCGTGATTCATTGTTTAACTCATGTCTGAAACAGCTTGGTCGGACAAA

GTCCATATTTTGGAAAACTCGAGTTG

> Cb_clean_k141_15719
TGTCAAAGAATTTGTCTGAATTTGCTACTTTCAAAGCATCTGTTAAGAAGATAGAACGGAAGGTTGATCCTTTAGATCTTAATGAAGTATCAAAGATC
GGTCAAAGAACAAAAGCAATTGAACTTGTAGCAGAGTTGGTTCAAAACGAGAAACTTCAAACTGCAATGGAAGTTGCCATGTCTTATTCTGGAATT
AATAATTCAAACTTTGAAATAATAATTCAAATATTTAAGAAAAATCAGATTGGAGGTGTTCGAGAGATAATAATTCTTTTAATAAAAGCAAGAATTTTA
TTCAACATAGTTGAGGAAATATGCAGACTACTGAGTAAATCAGATGCGAGAGAGATCCTCACAAAAGGTAAAGACAAACGGCTAATGATGAGGTCT
GATTATGAAGAAGTTTTGTCATCTTTTGAAAAGGGAACACCTGTCCAGATA

> Cb_clean_k141_26880
GTCTGATCTGGAAAAAATGAAGTCTGTCAAACCAACTACAAACAAACTTCCAAGAATATTACAGCTCGGGTCCCCATCGAAGATCATAAAATCAACT
GACAAATTTGAAGAATCTATAAACAAATTGAGGTCTTCTGGTTCACATGGTGGTTATTTGGATTATATATTGAGTTCGCTTATGGTGGACCCAAACCCA
AAGGATAGATTAATACAATTAGCCCTTCCAATTAGTAAGGTTGAAGATGAACAGAAGTCAGGGCCCGGTAGGAAATCTTATATGAAGAAGCATGGTAT
AACAATGGCTAGAGAAGTCCCAACACATATTGGTGTATCACCACAGCACATAAAGGTTCTTGATGATTTTATGACTGACTGCTCAAACAGCATAATTG
AGGTTGACCTTCCACAGATTAAACAACAAGATGACTCAGAAACAGGTATATCAGCATTCACCACACTGGATGATATTGAATCAAGATTCAGATCAGA
CAAGTTTACT

> Cb_clean_k141_6617
CCCCCACAAAACTGAAAAAGAATTTAACAAAATGGATAGAACTACCATTGCGCAAAGCATCACAACCCAGCATGGAGCAGCAACCAAAGTCTTTGA
TATCATTGCTGGTACGACAATTTTTGATTATATGCCTGAGACAACTGGACAATACTCTCAGTTTCGACGATGCATGCATGATATCATTTGTATGGCTTTG
ATTGATTCTTCACATTATCAACACACTGAACTCAAGCTTTCAGATTTTGGCATATCAGTCTCAGACAAAAAGCTAAATCAACAGAAACCTGACTTGTT
CACTGTTGATGAGAAGATAATCTCTATTGGTGAAGTTGTTTGCACATATTCACCTGAGTCTTCAATTAAGGAGAAGATATCAAAGTATGACCCCTTCAT
CTCAACGCTCGAACAACATGGGTTTGATGTGACAATGACTGTTCATTGTGTTGACCTCACCAGCCCAGAGTGGCAATCTTCGCTCCCAAGAATTTCT
GATATATTCATCGAAATTTTTGAAGATCTTCTAAGAACCTTGAGATTTATACACACAAATCCCAAATTTGCATCTATAAGGAAAAATGAATCTGGTCTT
TATAGTGTCGACAGATTTCAATTCAAGTTGGAGGATAATCATCTATCATCATTGATTGAAGAAGCAACAGGGTGTTCTATGGATTCTGCAAAAATTGAT
GCTAAACTCATTGGGTCATCTAGGGAGGATGATCAACAGTATATTGACACTATTGCAAAATCAATCATTGACAAGCAGATGCATGAACGACCAAGGC

CAAATCCAAGATCTTTTGAGCCTGAGATTCTCTTGTCTGACTTGGAAAAGATGAAATCTGTCAAACCAACTACAAACAAGCTTCCAAGAATATTACA
GCTTGGGTCTCCATCAAAGATCATAAAATCAACTGATAAATTCGAAGAATCTATTGACAAGTTGAGATCTTCTGGCTCACATGGTGGCTATTTAGATTA
TATATTGAGTTCGCTTATGGTAGATCCAAACCCAAAGGACAGATTGATACAATTGGCCCTTCCAATCAGTAAGGTTGAGGACGAACAAAGGTCAGGA
CCCGGTAGGAAATCTTATATGAAGAAGCATGGGATAACAATGGCTAGAGAAGTCCCTACACATATTGGTGTCTCACCACAACATATAAAGGTTCTTGA
TGACTTCATGACTGATTGCTCGAACAGCATAACTGAGGTTGACCTTCCGCAAATTAAACAGCAGGATGACTCAGAGACAGGTATATCAGCATTCACC
ACACTGGATGATATCGAATCACGGTTTAGATCTGACAAGTTTACTGCAATTGCATATTTCTACCAATGCATCTCAAATGAGATAGTTTTAAATAGCATG
AGGAGAAGGTCCACACGCCAGTATGCTATTGGATTTTCAGGCTTGGATGGTATTTATTTTATAATAGCTCCAGGTCCTCAACTGCGAACCGAGTCAAA
TGTTGAGTTTGTGAAGATAATCTCACTTCATCCAGGTGTTCCACATGGGTTATCAGCACCTTGGCACCTCGAAGGCGACCATTGGGAATCAGACTGG
TTGTCAGTTGACACAGATAGGCTCAAACACTGGCAAAGATCACGCGACAGAACAATCATGTGCACCTTATCAAACTGTGAGAGGCTAGTTCGACCT
GGAGTGAAACTCCGTGATGCCATGAAAGAAGAGTTGAACTCATACAATCATGCTCTGCTGGTTCTCACATACATTGAGAACAAGCAAACCACATCA
ACAACAAATCAAACAATAAGATACCTTTGGATGAAATCACTGGGTGATAAAAACTTCAAGGGGTTGTTGAAGAAGATGCCCCAGAGAGTAAACTCT
GTCATCCAATCAACAATGCTTCAGCGATCGGTGAATACTTGTGTGGAGATATCTCGAACTGATCTAACCCAGCTCGTCACTGTTGGCAGAACATTGAG
GGATGATGAAACTGGACACTATGACGAATCTACTACAGGAGTTACAAATTTGATGCCCAGACTGTTCACCCTTGGAGGTCCAGTTCCAATTTCTTACA
ATCTGAATGAGATATACTGGTGTATAGCTTACAACAAGGACAGACAGAACAAAACTCAAGATTCTATGAAAATTTTAGACAAGATCCTTTTAGAGGA
AGGGAAATTTGATGATGAACTGTCAAGCCGGAAGACTAATGAAGCGAAGGTCAACTACTTCCTTGGAACAACGACAACAGATCAAGACATTGAAC
ACATACACTCTCAGAACCCAGAAAGTCATTATTACAGCTACAGAGCTGTTCAAGTTGGCATCTCACTCCAAGATAAACACCCTGAAAATCGAGGTGA
CAATGGCTCATGGATGAATAGTCAGAGAATGTCAAATATACTGTCAAAGAATTTGTCTGAGTTTGCTACTTTCAAAGCATCTGTCAAAAAGATCGAAC
GGAAGATTGATCCTTTGGACCTTAATGAGGTATCAAAGATTGGCCAAAGAACAAAAGCAATCGAACTAGTAGCAGAGTTGGTTCAGAACGAGAAAC
TTCAAACTGCAATGGAAGTTGCCATGTCTTACTCTGGGATCAATAACTCAAACTTTGAAATAATAATTCAAATATTTAAGAAAAATCAGATTGGAGGT
GTTCGGGAGATAATAATTCTTTTGATAAAAGCAAGGATCTTGTTTAATATAGTTGAAGAAATCTGTAGACTATTGAGTAAATCAGACGCACGAGAGAT
CCTCACAAAGGGTAAAGACAAGCGGTTGATGATGAGATCTGACTATGAAGAAGTTTTGTCATCTTTTGAAAAAGGAACACCTGTCCAGATAATAAA
AGAATCTTACGACATGACAACATGGGCACAAAAATTCATACCAACAATCTTCATACCTTTATATCAGCATCATTTCAAAGAGTTTCCTGGGATGTCCTC
TTTTGCAAAATTCTTATTCTTATCACATTGCAACAAGAAAATGGAGTACCCTAAAGGCTTGATTGAGCAGTGGATCAAGCACCCATCTATAAAACATG
AGTCAAAAAACATTCAGCAACACAAGAATTCCTTCTTTGAGAATGGTGAGCCCTTTTTTGTCAATCATTCAAATATGTGCCAAGGCATTCCTCACTAC
AACTCAACAGTCCTTGCACTCTCTTGTCTCAGTCTTCGTGACTCATTATTCGACTCATGTCTGAAGCAGCTTGGTCGGTCAAGGTCCATATTTTGGAA
AACTCGAGTTGGATCTGATGATAAAGGAACTATAATCGGTCTAGATAAGTCAAAATCTGATAGCTACTATCAATATGTTCTTCTGGGACAATGTGAGA

GAGCGTCCGAGCGGCTTCATTCAATGGAGCTCTCTGTGAAATCTGCTAGTGGTCACCTGATGTATGAATTGAATTCTGCATACATGGCAAATCTTGAG
ACTCTATCACCAACAATCAAGTTTGCCTGTGCTTCCACCGACACAATCGGCACAGACTCATGCTCGAAGTTTATAAATGAGTCTTATTCCAGAATTAG
ACAGATGCGCGAGAATGGTTGCAGCTCCATTTTGTGCTCATTTGCGCATTCTATAAACTCAGATCACTTCTACAAAGTTTTTCAACTGGATTCGGTC
AAGAGAATGATGTTGTCAACACATTTCGACTTCCAAAAGAGGAAATACCATTCGATTTTGGAATCTACCCAACATATGATATAGATCTTCAAGATCTA
ATTGGCCCAGATTTCTACAATTACAGGATTGTCAAGTCAAACCCAAACTCACCAGTTGTCAAGCTTCTGTTTACTGAAATAAACCGTGAACGCAACG
ACGAGCTGTTCCCATCAGATGATCAACCACTACTAAAGAAAGACCATTTTGGAATACACCAAGGGTTAGTTAGACAACTTGAGAACATGCGGAAGA
GACTTAATGCCAACCCAGATGATGTTGAGGATTTTTTCAACGAAATCCATTTTTAATGATTCGTGGCCCAGAGACACTGGAGGAAACCAAGTACTC
AATAATCTCAAAACTGTTCACCAAAGGAGCATCTGAATCTCTTAGAAGGACATCACCAGCCATCTACATTGGCAGGTTGTCGGCATTCAAAGCTTCA
AAGGCATGGACTGCACCTTATGAGTCTCAACGAATTATTGATCCTGAAACCAATGAGATTGAGTACATTGACACATATATAAAAACCACCTATTCTGA
GTT

> Cb_clean_contig_1357
CGCTGAACAGCGGTTGGCGAAAGCTCTGCACCTACAGGCCGACATTTCTTCCTTTGTCCCGAGCGGATCAAAGGGTTTTCATGGTTTGGGAGAGTT
GGTACTCCCTACCCCTTCCGCCGAAGAAGTCGCTTTCGGTTCTTCTTTTCTTGGTGGCAAGCGCATTCGCACCTTGCCAAAGAGGGAGAAGAACAG
CCGGTTTTCTGCTTGGTGCCAGTCAAATGCCGAACGAAAGAAGGCATTGAGACTGGCCGAGGTGAAACGCCAGAAATTCCGATTGTCTTCGTTGGT
GGCAGACCCTCTTCGTGTACTCGGATGTCAAATTCCTGAGACATTGGATGGTGTAGTCTCTTTTGCTCGCGGCCTCTTCGGTTCCTCTTCGCGTCGCT
CTGTGGTCCGTAGGGATTTCATTCATCGGGCCAGGTACGCTGGGAAGATTTTCTCTTGCCCTACCAGTGGTTTTATCATCAACCTGGTTGACAATAGG
GTGAAAGTGTCGAGGAACAGGACCGGGTCCGGTGTTTCTTTCCGTGTTGATGGTAATCAGATTGCGCACTGTGGCTTAAAGGATGAGTACCTCTGTG
CCGTCCTACAGTCCAGACTCAATCTGATACCAAATCCTGGTGAGGCAGAGTTCCTGACCATCGACTTGTTCCGTTCGAGATACGAAGAATACTTCGG
AAGGTTCCTCGCCGAGATGGATGGAACCAACTTGGACTTGTGGTCCTTAATGACCATGGCTAAAAGATGGAAGCGGGATTTCGGATGTCCTGGCAAT
ACTTGCGTCTTATCTTTTGCGGGTGACTCAGTCTCATTCACCGTTATCAATGAACAAGTCGAGTTACCGCCGGCATTGTGGTTTCGTCTTGATGGAAA
GGATCTATTCCATTGCGGGGTTGACCAAGTTAAAAGACTGCAATTCCAAAAGAACATTTGGAAGGCGGTTGCCAAACTGGTCATTCAGGATGGTAGT
CCGGCCGAACAGCGATTGGCCGAGAAAGTCTCTTCTTCTGCGCATGTGAGTGTCCCTTGTCTGAAACCTAACCTGTTCCATCAGCTGACGAACGGTT
TCAGGGTTTGTGCCAAGTTTTCCGGTCTGAGCGTCTTCGAACGCGGACTTGATTTCACACGACCTGACCCTCGGGTTGAGGGTGGTTTCATCACCAC
CAGGCAGGAGAGATTGTTTTGGGATACAGGAGCAAGTAGGTTGGTGTTCACACCGAAGGGCCTGGCCAAAATATTCTTCCGTAAACACAATCAAGA
AAAATGGAGAATAGCTCTGGCTGCCGCGATTGGTTTGACCTATGATGAAGGTCTGCTCTTCTTCTCGAAGTTTCACAATGATAGTACGAAACCAGAT

GTCGTGTTGGAGGTTCTGGGGTCATCACCCTACCACAAGCCGGACAAGGAAACGGTAACAAGAGTAGCTTTCATTGACGTGGAGAAACCGGTTTCA
TCCAGGCCCATACCTCGGCTTCCCACAGTTGTTCCGCCTCCTCCGCCTGTACCACCAGTGCAGGGAGTTAAGCAGAAGGGACCCAAGGTGGAACTG
GCTGATGGTGACACGTGGCTGCTTGCTGAAGGTTCTGAAGGTAAGTCAAATAGAGCTCGCTTCAGATCTCTCTTCCCACATGATTGGTGGTTGAGAG
TGTCCGAAAGGGTCATGCCCTTTATGAATCAAGACGCATCCCGTGACGTTGGATACAGGTCCCGATCTTGGTCCCAATCTTCCCTTGTCTCAGATCCC
TCAGAGAAGTTTGATGTCAACGCCCAAAAATACACACCTCCTAGCTCTTCCAAAGCATCAGGTTCAACACCTTTACGTGTTGATCCTGACGCCTTTA
GAAGGCAGTTGCAAGCCAAGGCTAAGAAAGTGGGTGCGACAGCGTCTTCCCGACCTGACGCGAGGGAGGCGTATCTGAAGGGTGCCAACGAGTT
CAAACCGGAGAAAGTTTCTCATAGAAAAGCCCTTTCTGAGCCCACTCCGTCGAAGAAACCTTCTTCTGAATGGATGTCTATTTCGCGAGCATCCTCT
GATTATCTCTTTGATGATGATCATGAGGACGCGAGAGATTCGACGAAATCGGACATCGAAACTAATCCCGGACCTTCTATCAGGCATTTCCGTTCTAA
ATCGACTTCAGATTTGGAGGGTGAAACACCGTCATTGAATTTCACACCACCGACCCCTGGTGATGTCTTGACTGTGCACCATCCGACACCACCTCGT
TTGAGGGTCAATCACAGGATGAACAGACATACCGTGATTGTACCTTCACCGGGTGAGAACACGTCGAATTGGAATGTAGGTTACTCGTCATTTGCAT
GGCCTCCAACAGAGGAACATCTGGAACCTCGAGACTCCAGAACCGCGGATATAGAAGAAAATCCCGGACCATGTGAAACCCTTGACCCCACTTTGC
CTCAGTTTCGAGGTCGGAGTGGTTCTCAGAGTTCAGTTGCCGCTGTCACTTCGGAGCGATCACCTTCAAGAACACGTAGGTTTCTTGGGAAGATCG
GCATCCATGGCAAAAGCGCAACGGAAGAAGTTAAGGACATTTTTGGATTTAAAGAACATGATGAGGTGTATCCGTACACTGGTAAAAACTCATCTTT
TGTTGAAAAGCATTATGAATTCCACTATGTTCTTTCTCATTTACCTAAAGACAAGAAGTGGCGGTCATCAACCTTTATCAGGTGGGTTGCCGCTGCTT
CTTTGTCCGAGCTTCAAAAATCACTTGACTGTGTCGTTAAGGCTTCCCACCCTTTCAACACCGCGCTAGTGAGTAAGTTGCGTGATGAGACAAACTC
CTTCTTCCAGAAGATCTCTGATTCTGAGCCAGTAGGCGGTACAACCATGTCTGCCTTAGCTCACACGTTCAAGACCCTATACGCCTGTCCAAACAATG
GATGCGGTTATGCACTGGAGGTGAAACCGGAAGATGGAATTGTACTGTTCCATCTGACAGAGTCCCCAGACGAAATCGCCAAGAATAGGTTGTGGT
GGGTCTTGAATGGGAATGAGATTTTGCACGGAGGGGACAAGGCTCCTGCCTCTCCAAGCACAATCTCATTCGGTTGGCTGGCAAAGGAGTATTCAA
CACATGAGGTGATAATTGAGCCCAAACCGGACTCTATGTTATCACAAGAGAAGCCAGGGCCGTTCGCGATGCCCTCACTTTCTCAGTTTCAGAAATG
TGTTGACAGGATTATCCCTACTGGTGGTCATTTCTCCCGCTCTGAGGTCGACGACCTCAAAAATCACATCAACCAATACCTGGAGTCTACAGATGTCA
AGATAGTAGATCCAGTGTTGGTTGCAGCGATGGCAATGAGCTTCAAGTCAAAGTTCAATTGTCCAAAAGATGTCAGCTCTTTTGCAATTGATTTTAGA
CTTGACCGGAGGATGGTTGTTTTCTGCGCCACAGATTCCCCTGAAGACAAAAATAAGGGGAAGCTTTATTGGTGCATTGGTGGTAGTGTCATTGTCC
ATGGCGGGCTGCTAGGTATCGAAGATGGGAAGTCTTGTTCCCCTTCACTGGATCACTATAGCAAGTTGTGGACGATGCCGCTTCCAAAATCGGGTTG
GGTGGACAGGTCATTCGACGCTGAATACTTCACAAAGATCTCAACCTGGCAAAAGGCCCTCGACCGGTGTGAACCGATCGTTGTATTCTCTGAACA
CACATTGCAACGCACACTTTACCAATACTTTGATGGTGTCACTGACAAGGTGTTATCACCATCACATACTGCAATCGTTGCTTGTGTGGTAAAATCCC
TGATGAAATGCCCTGAGAGCGGGGTAGGTTATGCTATACAGTTCGATAGTCATGGGGCTTCGCTGTATATGTGTCGACAACCGAACTCCGCAGAGGT

GAACCGGTTGTGGTACTTTCACTGGGATTTTCCTTTCGTGCATGGGGGAAGAAAATCTTATGCTCCGGAACCCAGGCAGTTTTTCTACACTGAGCAT
CTCAACATAGATTATTCGATACCCATGCGCGAAGTTTTTGCTGGGGAAGGTCTGCCCAAGTCCAATTCCTCACATTTGACTTTCAGGAGTCAACAGG
AATTGTTTTTCGGTTTCCAGCAGCAAATAGATTTGTATGATGTGCCGACACCGGCATTCATGCATTCGAGAGCACATGGTGTGGCTGAACAAGCCATT
CGTGACACAATACCCAAAATGAGAACTCTCCAGTTCAATTTTGAGCAAGTTGCCGCGATAGCCATGTGTTATAGATCAAGGATGCAATGCCCATGTCC
GGAGTCAGCCATGGCTGTTGACTTTGACTTCGCCAATGAGCGCATTGTGTTCTTTTCGAACTTAGGCAGTGGAAGTGTCCGCTGTCGAGGTCAAATG
TGGTGGTGTATAAATGGAGTCGGACTAGTGCACACTGGTTCTACATTCCACACCTATCACAACCAGTCCAACCAATTTCGACCCGGTGCCCGAGTAT
GTAGGTACTGGGACGAGATTGTTTCTTTTGACTTGACTGAGTTCTGCCAGCCTACGTTGGATTATTGGACATCGGATTTCCATTTGCAGCTTGACAAA
GACGAGCAGCTACAGAGGGCTCAGATTGCATTGGATGCGCATAGTGACCCTGTTGAGCCTTTCAGCAGGGACATTGCCATATTTCATGCATTTGAATC
GATCAGACAAACAGCCCAAGCGGTCGATATAACTGTCATGAAGATTGAACATTTGATCATCATGGCATGCATATATCGTTCGAAAATGGCATGCCCTA
CTGTAGGCACCTCAATGATTGTCAAGTTCGATTTGTCAAATCAGAGAGTGAGGTTTGGCGTTGCAAGAGGAACGTATCCTGAGTCAGATGGCAGGAT
GTGGTGGTATCTGCACGATGATTGCATGATCCACACGGGTAGGCCGGAAAAGCTTCACACCGCTAGGGTGGAAGCGCCGTTGCCTGTCAGCAGTCG
ATTTTCCTTTCGTGCAACTCACCGTCAGGCCTGTGAAATGGCCGACGGTTTTGAGAAAACTTGGAGAAAAGACCAAAGATCCGTTTGCAAAGACAA
GGGTGAAAACCACAATCATCATCCCCTTTTCTGCGGATCAACTTTTGACGAGAGGCACGATGAGGTATTCGTGTCCGCTTACATATGGTTCTACATCC
AACTCATTTGCACGTTTTATTTCTCCTTTTCCCGGCCTAATCTGCGGCGAAGACCTGACGGTCACACAGAAGGGGGAGAGTATCCCGTTGCGGATGA
GAGTTTTTCAGCCTCATCTTCCCTCCTAGTGTGCAAGTTAGGGACTAGAGGTGACCAAGTTCCTGTTGATTATTACGCGAACGTGGCGGCACACTATG
GTGTCCGTACACATTCCCATGTGTATAAAGACGTAACAACAGACGATCTTGAAAAGATGAAAGTCGGAGACTTTTCATCTAGTTCCGCTGGTTACCT
GAATCTTGCGTATGCTTCTCAACTTGGTTACAAGAGACTGTTCACTCCGCATGTGGAGGTGGAAAGGTCTCAAGGTGTAAGTTATACTTTATCACCAA
GCTCAAAGTGGGTGGAAGCAACAAGGTTCATTTCCGATTGGTCGAAGGTTTCCTGGTCCAACTTTCTCCCTGCGTGGATAGCCACGAGGGTCACTG
CTGTGTATGTGCCAACTTTCAGAATTGGTGCGCTGTCCGGAAGCGACATCCCGCGTTCTCACGATGGGTTAAACTTATTGGAGAAAGTTAAAAACAC
AGCAGAGTATGAGGAAGGTTGGACATCCGGCAGCTCGTCAGTTTCAGTGATCCCTGACGAGATCAAGTCGAGATGCCCCGAAATACCATCGGGTAA
TCATCTTGAAATATTGAAGGATTACAAAACGGTGCACATCCACGGGGGGGCCGGCACTGTGCAGACCGCCATTGCCTGCGGTGCACGACCGGTTATC
CACGACAAGACACTCGATAGAGTGTATCATAGGATGCCTGAACCAAAGGATTTCAAACAACCCTCCATTGGCAAGTTTATGGGTTGGTTGGTGTCTG
CTGGTTTCAAAGTTGACGCGCCCATTGAGGTGAGAGCATGGTGGTTGTTACAATGGTATTGGTCCCAAAAATGGAACTATGCGTACGACGCATTAGT
TACACTTGCGAAAGCCTATACTATATGTTCATATGTATATAACCATTGGGAACTTTTCCTCATTCTTTTCTTTACCGTTCCAGCAGTGATCTGGAGGATG
CTTTTCTCTTCCAAGTCATTGTTCGCCATAGTCAAAATCTGTGCGCTTGGTTTTTGGCGCTACCCTGTGTTATGCTTGCTAGATTCGAAAGTCACGTTT
GTGGCGGGCATCTGGTCAATGCAAAACTTGTGGATGGGCGCAGCACAAGATTTCGCGGCACTTATGGAGACACGTTCCTCGCTGGTTTGGGAGCCA

GTGCACAAGATCGTGGGAACGAAGAAGCTGACATTCCCTTTTCCCCTTGGGCATTGGAGCATGAAAGACAATCAAACCGGCAAGATCTATGAAGGT
AGATTTGTCGGCCCCTCTGAAACGATCGGAGGAAATTTTCGTCTTGTAAGTACCACTCGTGAGTACAAGCACGGATTTAAGTCTTTTCCGGCTCCTTT
CAATGTTGGCCACGCGGAGTCACTCGTAAGAGCACAACACGTGGCACCATACTCATCTTCACATAATTGTGTGACCATGATGGTGCAACTTTGTGCT
AAGAGATCGACTGCATGGACAGCATGCATGATAATGGTTGGTGCTCTGACAGGGTTGGCACTGGCACCACCAAAATTGCTCAAGGATGTTCTTTCTT
TTCTTTTTCCTGGCAAGGATTTTGCTAACACCCCTTTTTATCGCTCGCTTGGTTTCGCCGCGTCCCTAGAAAACATCCCGCTTGAAATAGAGGGGATG
TTAGAAGCGGCAGAACCAATGCCACAGATAGCTACACCAGAACCTACTGATCTGGCCCACCCAGATTCTTTCAGTATGTTGGTTGATGAGGTAGCGT
TTATCCAGGCATGCGTGGAACAACTCAGGATACCCACCTTAGATAAGGAGGCATTTGACGAAGTTTCACAGCGGACTTTAGAAAAGGAGTTGGACA
AGGTCGACATCCCTGAAGACTCTCTGATCAGGGTGTCAAACATACCGCCAAGAGTGCACCATGAGTGGGCGCAACTGGTGGATGGTCTACACCATG
CTATTTCTTTCGTTCGCACAAACCGCGTCGCAGACGCGTTTGTGTTATACGTCAAAAATGCCGGTGAAAACGTATATGACTTTGTCCAGCCAATACTG
GAGATCCTTTCCTTCTTTCTGCACCTTGCAGCTGAGCACTCCAAGCATGCGTTCGATCGCCTGTTCAAGTGGCTTTGCGATTTCTTAGACCATGTGTG
GGGTCTAGAGGCGTCAAAAAGAGTCAAAACAGTGTGGGGCCTGACTGGCCTTCATCGAACTGGATTGCTTGGTGTCAAAGCCAAACTTGCAGCCA
ATGTTGCTTATTCTGATTTCGTGGGTAGGCAGGATTTTGAATCAGATTACAACAAATTGGTGCAAGAGGGAAAACACCTCGCTCAGGTATATGGTGCT
GTCAAACGATCAAACCTTGGAGGGCCTCAGAGGAGGCCTATAGGCTATGGAAAACCTCTAATGTCACAATCTGAAGCCAGCCTGCTCGGTTTCGAG
TCAGGTGAGTTTGTGACTGATCGTGAGTATGAGACACGTGTGCAATCATACCTTGAACAAGGTGTTGCACAAGGTGCTGACGGAGTATTCTTGGCCG
ATAAAAGACCGGAGCTGATTGCCAAGTCACAGCACCGTTATGAGCCAAAGTACCCCGCACTTACCTCTGAGGATCGTTCCATGGCTAAGGAAATAGC
CCAAGCCATGTTTGATCAATACCCGGAAGTCTTCGCTAATTGTGATGTTCTGCCGCCAAGGGCAGTTCACAATTATGTGAAGCAAAAATATTCCCCAG
GAACCCCGTTCATCAAGCCTGGCGGCTTCAAGTCTCGTCAGGCGATGTTCGACGCTGGTTATGACCGCGTCATGCAACGACGAGCAATGCATTACTT
GGAAACTGGTGAATATCCAGTACAATTCTATCATGCTTTTGTGAAATCACAAGTGGTAGATATCAAGAAGTGCTTGAGCCCCGAGGAAGGTGGGAAA
GGAAAAGATGTTCGCACTGTGGTCAGCCAGGATCTGTTCTCCTACTACATTGACCAATGTCTCCAAATCGAGCGCAACAAACGCATAAATTGGGATA
CATATGGTGCAGGTATAGGTATGCCTCTTAATCAGTCAATGGAGGCAATATACAACAGAATGGCAGCTGCACAGAAGTCGAGAGGAGGCAGGTACAT
CATCGCTGATGCAACCGCCTTCGACTCGTTTTGCAAACCTTTCCTCTTCGAAGTAAACGCAAACCTTTGGGAACTCGGCTATAAAGATCATCCAAGC
GGTAACGGCAAGAACCTCGCATCTGTTCTAAATGCTTCGTATCAAGCCAGACAAAATGCTTGGATAATCGGTGTGACCGAGCCGGAATATGACTCGT
TGACTGTTTGCGTCCAAGATAGAAACAGTCGCGAAATCATACGACAACGATCCCCAAAGACAGTAATCTGTTTGGAAGATCTCATTGACTGGGACA
AGTACAACAGATTGCCTGCTGAACAACGTTCGAACTACGTTGCAAAACTTCAACCACCTGAAGGGAAGACGGTGGTAACCTGGGATCCTGCTCTCC
GCCCATCCAGATCAAACTGGATGGGTAAATACGAATATGGTGTCTTATCTGATGTTGACAAGAAATTCTTTGAACATCAAACATTCACATACGATCCTT
CAAATTTTGAAGACATGTATCAAGACATATTATCGGTTTCACGATCAAATTACAAACTGATGTCAAACGTACATGCGAAGAATCGTGGTGGAAGCACT

GGAGGGTCAGACACATCAAACGTTAATACACACGCCTTTAAGGCAGGTGTGATTTACGCTTGGTGTCAAACCACTGGCAGAGCTCCAAAGGAGTTC
TTCCAGTACAATGACCTTGCCAATACATCAGATGACACCATCTGGCAATCGGGTGGTAAACATGGCCTTAACACTGTTGAAGATGTCGAAGTTTTCC
GGCATCATTGTCAATCAGTGGGTATCCATCTGGAAATAGAGACAACGAAGAATATATCACAAGTAGAATACCTGTCAAAATTCGTTCGTGTTCCCACT
TCAGAGGATTCAGCAGCATTGAAAGTCTGGAGATCCCAGAAAATCAAGGCTGCTTCAAATGCCCACAAACAATTAGGCCTAGCTGTTCCTACTTCAT
GGGAGCAGTTCAACAACCCAAACTTTGTCGTCGTACAAAACCCTTCTGCGATCTTGCTCCGAAGGTCGGCGTTTAGGTATTACCAATCAAACGCAA
ACCATTGGAAGTACGTCTCAGTAGAGAGGGGTGCCGGTCATGCTCAAAGCACCGCCTTCCTCCCTGAGTTGTATGGCAAGTTCGCACAAGAGTGGT
GCGACGACATCAATGATCTTCTAATACAACAGAAGATCCACCGGAAGTTTAAAGTTACAAATGGACAATTTGGATTGCAATGTGTCGAACAAGTAGA
TCCGAGAGCGTCACAACAACAGCTCTCGCCGCGTCAAGTTGCTTTCCTCTCCTGGTTGAGAGGAAACATGTTCCCTAGTTACTATAGGGTACTTGAC
ATCCACATGAACGTCGCTAAAGTAGATCCAGAAAGACACGCCAAATTCCTTCGTAAGCTTGAAAGAGGTTGGCGAGGTTGGGAGGAACTGCTACGC
GAAGGAGTAGACAATCTCTACGCCTTAACTGATTCCATTCCAGATGAGATCAGCAAGAAGTTCCAACCCGGCCTCGATATGCTCTATGCAGAGCAAC
CGTTCTACACGAAGAACAAGGTAGTCGAAAAATTTGTTTACCTAAAACTGCTGGAAGAAAGCCCAGAAACAGAAGTTACCTTTTCAGACTTCTCTG
CTCGAATTCAAGAGTCACCATATGCTTCGGTATGTGATCCCTATCACTTCTGGGAGAAGTTAGGGGAACCTGAATTCAAAGAGCAACTCATGTCTGA
AGATCCTCTGAAGATACAAGGTCTCGTTTTGCTCATAAGCACCATGTACCTATTGACAAACACAGCTGAGTCGGTAGTATTAGCCATTCCGTTTGTCG
GCTTGTTCTACAAACTTTTCCTGTGGTCCTTTATCGGGCTCAATAAGGTATATGGGTTGTTAAACACCATGTACTGGCATTCTGAGGGCAAGTCGTCTA
GAGAGATATCTCGAATCATGCCCCGAGACCCTTACATGGTGACAAAACAGTTTGCTGCGTTTTGCATTGACTTTGTGCCATCACAGCTGGGTTACTTG
GCTCTTATCCCGCTGCACATACTATCTTTGCTTCCAGTGTTGTTCGAAGCACTTGGAAAAGCATGGTATGTGGCAGGGGAGGTCAAGAATGTGATGG
AACCTCCTGGCAGTACCGTAAACAAGTGGGATCAATACGCAGACGAGTACATAGAAGCTCTGCGTTCATCTCCGACAAATAGAAAATACGTTGCTGC
CAAGACTTCAACTGGCAAATCGACCTGGTTCATAGCTGCCTTGTGGAAAGCTAGACACCGCCAAAAGGTCAGAAAGTTTTGGCTTGTTGAACCTCG
ATTGGTGTTGCGAGACAACACGCACATACCATTCGATGCACCTTATCAAGTGCTGAAGGACAACATACAAGTTTCAAACACCCATGATATCCACATAT
TGACGTATGGACATTTACAGTCAAGGTTGTTGGATATAGACAATGAACGTGATATCGTCATCTTTGACGAGTTCCACGAACAACAGGGTGAAATGATC
CTTGGACTCGATAAGTGCAAGGCGCCAATCTTTCTTCTCTCGGCCACGCCAGCCGAAATACCATCTCTGCACGGTTCCGGAACTTTGGTCCCAAACA
TTGACAGACGACATCCGATCCAAATCCACCAAGTGGACGATGGGATGGGTGTTGTTGACATGTTTATGGAAGCCAAAAACCGCTACCCTGATTTATG
CAAGAGAGCGATGATTATTGTGCCAACTCATAAGGAAGTTGCAAAAGTTATCGCTTCCCTTGATTACCTCAAGGTAGGCAAAGTGTCTCCTCTATCTG
CCCGCGATCGCGTGGTACAGGATGAGGGAATACTGGTGTGCACCCCCTACGTCCAAACGGGTCTAGATGTAAAACCACCACCCGATCTCTTGATCGA
CTGTGGAAAAGATTTCGTTTTGGATAAGGGGCGTCCAGTTTTCCCACTTCCGTGGACAGACAAGGACATAAACCAACAGAGAATTGGTCGTGTTGG
AAGATTAAGACCCGGAATCGTCTTCCAACCGAAATCAGCAGGTTCCGGAAAGAAAGGTGTGGTATACCCGAGTCCACATCTATTCCAACATAAAGCT

GTCGCGGAACATTTTAAGGTCCCACAGCTTGCACCAATCTCGGGTGCCTTTACGGCAGATTTACCTTTCTTAAGGCCGAACAAGAAAGTGTTAAATT
CTTTACCCGTCGAGAAATCAGTCGTTTTTATACATTCACTTGCGTTAAGTGGTGTGCGCCAAGAGCAGTGGCAACACATGTATACGAGATCCGTGCA
AGGGCACACTCTCGGCAACGATTACAAAAACGTAGATAGACTACTCAATCATTCAAGGTACAAACGATCAAAGCTAGTCTCGTATGGAGACGCACTT
TATCATCTTTACCGAGAACATGCAGTCCAGTACGGATTTGTCAAGGAAGACGGAAGTACAGAATACAAGTCTGCCAAGCCAATGTCTGTCATCAATG
GAAAGTGGATAGAGTTAGAACAATCGCCGACGACTAAGATTGATGTTCAAGAGTTACCACAACAGGAACTTTCAAACAAATATCAATCTTTCGAATC
ATCAATAAGGCGATGTCGAGACTCCATTATTTCAAAGCCAAGGACATGCCTGGGCATGACTTGGAACGTCTCAACTCCATCTTAGCGTTTTGAGCA
CTAAAAGGCGGCACTTGTAGCTGAAATGCTACATTTAAAGACAACCGCAGTTTTGTGTTACCCAATTGACGTTTGACTTTTGTCAGACACGCTATTTC
TGTTGTTGCCTCCCGACCTCTTTCGTGACATTTGTCACATCCGTTCGTTACCTTTCTTTCTTTGTCCGTAAAACTACAAACAAACGCAGCGGTTAATG
CCGCGGTAATAAACCGGATTCACATGCTAACGCATGGTGTGGTTGGCCGTTTGCCAACATAATCACTAGGGTGGTTAATTCCACCCTATGAGAAGGCT
CTCGATTGACTTCTTGCCGTACGGGTTGGTCTTGTAGGTCTCGATGCCGTCGTGCACGGCTGGGTAGGAGGTGATGTGGGACAGGAACTTGGACTGT
GGCACAATTTCTCTTAGCCAACTCCTTACCTCACCACTCTTCTCTCTCACTCGCGCACTGCAAGGTTATCGCTCTCTGTACTAAAGGTATTACCAATTC
GTGCTCTCCGCCATTCACACCACTGGCCCGTTCACCTGGGTGCAAAGGCTTAGCGTAGCCTTTGCTGGCCTCTCACTGGCTTTGCCGATTGCCTGAA
TCG

> Cb_clean_contig_1024
TGAGGTCCCCCTGCCTTGTCAAAGGCGGCCGTGCTTACCCGTCTTAAACAGGTACCCCCGAGGGGACAAGCACGCGCACTTGCGGAGGAGAGATC
AACTATGGACGCCCGTATGGGGAGAGAGTCCGTAGGGGGCGCCAGCCGGCGCACGACACACCGCGCGAAACCTACTCAGTAAGCTGAAGTAGGAG
GTTCGCAGACGAGAAGAACCCCGTCTTCCACCAAACAAACGGGACCAGGGCTGCAGGGCAGCCGAGACTCCGCTAGGACGACCTCGCCACGCGG
ACGAGGACAGGGAACCGGTGACCTAACGCCGAACGTAAGTTCGGCGCCACGCCAACCTAAAGAGCGAGCCCGAAGGTACGCTCTAGAAGGCTTA
ACTATCAAACGCTTGAAAGCGTTGTAGGAAAGCGGATCCCTGGCGGTCTGAAAAGACCGACCGCCCACCCAAAGGTCCAAAGAGACCTCCAACCT
TTCTACGCTGGTCGAGGACAGGCTGAGCCTGTAACGACTGTCATCGAGACCGGTGGCGCGCGGCAAGACCGCATAGCCGAGGCGGTGAAGCCGCC
GCTCGTACTTGAAAGTCTCAAGTACGCCGAGATCGAAGCCGAGGTCACTCGGACGTAGAACCCACCTCGAGCGAAGTTTCGAAAGGACGAAAGCA
CGCTCCCACAACGGACCGGCAGCACGACAAACAGCTGCCTGGTGGATGTGACCCTGGAGGTCACGGGAACCACCACCCCTCCTGAGGTGCTTCAC
CTCCTTCCACTTTCCACGTGAGTCCCTAAGGAAACACGTGGAGTTAATCTCAGCTACCGACTGAAAACGGCCGGTTTTGGACTCGTTAATGACTGCC
CAAGAGGGGTAGTCACTATTGAGAACGGGAGTACGACAGCTTATGAGGCAGTCGTCACCGTTGATTAACACTCTGGCATCGCGCCCGCGTACGGCC
CAGCGGGCGGCGACGTAGGACTGAAGGCAGAGTAAGGGGAAAGAGAGGTAAGTGCCCATCATCTGGCCGCGAGTGACAGAAGAGTCACCAACGG

TCGGACGAAGGGAATCCACAGCGTCCTGGCGCACACGACCAGGAACCAACTCACACTGCGCGAGGGCAGCGCAAAGGATGGTATCGGCGACGTC
CAACAGAAGGTTGTCAGTCGCCCCTACCAGATCAACAGAAGTCTGCCAGTCAAACTGACAGACCGAGCTGATCCTGTCTGCTGTTGGAGGACCAA
CAAGCAGCCAATCCTTACCACCAAGGTACGAATAGAGGCATTCGTGCAAAGGCCCAAGGGTATCCCAACGGTAAGTTGGAATACCCATAGCCCTCA
ACTTCCCTGCGGAAGGAACCTCCTTATAACGGAGGCGCCAACCGCCCACCCCCTTGGGAAGGGGACCACCCGACTGAACTACTGACTGGAAGCCC
AAAAAGGACTGTCTA

> Cb_clean_k141_35317
CACGGCCAACCACACCATGCGTTAGCATGTGAATCCGGTTTATTACCGCGGCATTAACCGCTGCGTTTGTTTGTAGTTTTACGGACAAAGAAAGAAA
GGTAACGAACGGATGTGACAAATGTCACGAAAGAGGTCGGGAGGCAACAACAGAAATAGCGTGTCTGACAAAAGTCAAACGTCAATTGGGTAACA
CAAAACTGCGGTTGTCTTTAAATGTAGCATTTCAGCTACAAGTGCCGCCTTTTAGTGCTCAAAACGCTAAGATGGAGTTGAGACGTTCCAAGTCATG
CCCAGGCATGTCCTTGGCTTTGAAAATAATGGAGTCTCGACATCGCCTTATTGATGATTCGAAAGATTGATATTTGTTTGAAAGTTCCTGTTGTGGTAA
CTCTTGAACATCAATCTTAGTCGTCGGCGATTGTTCTAACTCTATCCACTTTCCATTGATGACAGACATTGGCTTGGCAGACTTGTATTCTGTACTTCC
GTCTTCCTTGACAAATCCGTACTGGACTGCATGTTCTCGGTAAAGATGATAAAGTGCGTCTCCATACGAGACTAGCTTTGATCGTTTGTACCTTGAAT
GATTGAGTAGTCTATCTACGTTTTTGTAATCGTTGCCGAGAGTGTGCCCTTGCACGGATCTCGTATACATGTGTTGCCACTGCTCTTGGCGCACACCA
CTTAACGCAAGTGAATGTATAAAAACGACTGATTTCTCGACGGGTAAAGAATTTAACACTTTCTTGTTCGGCCTTAAGAAAGGTAAATCTGCCGTAA
AGGCACCCGAGATTGGTGCAAGCTGTGGGACCTTAAAATGTTCCGCGACAGCTTTATGTTGGAATAGATGTGGACTCGGGTATACCACACCTTTCTT
TCCGGAACCTGCTGATTTCGGTTGGAAGACGATTCCGGGTCTTAATCTTCCAACACGACCAATTCTCTGTTGGTTTATGTCCTTGTCTGTCCACGGAA
GTGGGAAAACTGGACGCCCCTTATCCAAAACGAAATCTTTTCCACAGTCGATCAAGAGATCGGGTGGTGGTTTTACATCTAGACCCGTTTGGACGTA
GGGGGTGCACACCAGTATTCCCTCATCCTGTACCACGCGATCGCGGGCAGATAGAGGAGACACTTTGCCTACCTTGAGGTAATCAAGGGAAGCGAT
AACTTTTGCAACTTCCTTATGAGTTGGCACAATAATCATCGCTCTCTTGCATAAATCAGGGTAGCGGTTTTTGGCTTCCATAAACATGTCAACAACAC
CCATCCCATCGTCCACTTGGTGGATTTGGATCGGATGTCGTCTGTCAATGTTTGGGACCAAAGTTCCGGAACCGTGCAGAGATGGTATTTCGGCTGG
CGTGGCCGAGAGAAGAAAGATTGGCGCCTTGCACTTATCGAGTCCAAGGATCATTTCACCCTGTTGTTCGTGGAACTCGTCAAAGATGACGATATCA
CGTTCATTGTCTATATCCAACAACCTTGACTGTAAATGTCCATACGTCAATATGTGGATATCATGGGTGTTTGAAACTTGTATGTTGTCCTTCAGCACTT
GATAAGGTGCATCGAATGGTATGTGCGTGTTGTCTCGCAACACCAATCGAGGTTCAACAAGCCAAAACTTTCTGACCTTTTGGCGGTGTCTAGCTTT
CCACAAGGCAGCTATGAACCAGGTCGATTTGCCAGTTGAAGTCTTGGCAGCAACGTATTTTCTATTTGTCGGAGATGAACGCAGAGCTTCTATGTAC
TCGTCTGCGTATTGATCCCACTTGTTTACGGTACTGCCAGGAGGTTCCATCACATTCTTGACCTCCCCTGCCACATACCATGCTTTTCCAAGTGCTTCG

AACAACACTGGAAGCAAAGATAGTATGTGCAGCGGGATAAGAGCCAAGTAACCCAGCTGTGATGGCACAAAGTCAATGCAAAACGCAGCAAACTG
TTTTGTCACCATGTAAGGGTCTCGGGGCATGATTCGAGATATCTCTCTAGACGACTTGCCCTCAGAATGCCAGTACATGGTGTTTAACAACCCATATAC
CTTATTGAGCCCGATAAAGGACCACAGGAAAAGTTTGTAGAACAAGCCGACAAACGGAATGGCTAATACTACCGACTCAGCTGTGTTTGTCAATAG
GTACATGGTGCTTATGAGCAAAACGAGACCTTGTATCTTCAGAGGATCTTCAGACATGAGTTGCTCTTTGAATTCAGGTTCCCCTAACTTCTCCCAGA
AGTGATAGGGATCACATACCGAAGCATATGGTGACTCTTGAATTCGAGCAGAGAAGTCTGAAAAGGTAACTTCTGTTTCTGGGCTTTCTTCCAGCAG
TTTTAGGTAAACAAATTTTTCGACTACCTTGTTCTTCGTGTAGAACGGTTGCTCTGCATAGAGCATATCGAGGCCGGGTTGGAACTTCTTGCTGATCT
CATCTGGAATGGAATCAGTTAAGGCGTAGAGATTGTCTACTCCTTCGCGTAGCAGTTCCTCCCAACCTCGCCAACCTCTTTCAAGCTTACGAAGGAA
TTTGGCGTGTCTTTCTGGATCTACTTTAGCGACGTTCATGTGGATGTCAAGTACCCTATAGTAACTAGGGAACATGTTTCCTCTCAACCAGGAGAGGA
AAGCAACTTGACGCGGCGAGAGCTGTTGTTGTGACGCTCTCGGATCTACTTGTTCGACACATTGCAATCCAAATTGTCCATTTGTAACTTTAAACTTC
CGGTGGATCTTCTGTTGTATTAGAAGATCATTGATGTCGTCGCACCACTCTTGTGCGAACTTGCCATACAACTCAGGGAGGAAGGCGGTGCTTTGAG
CATGACCGGCACCCCTCTCTACTGAGACGTACTTCCAATGGTTTGCGTTTGATTGGTAATACCTAAACGCCGACCTTCGGAGCAAGATCGCAGAAGG
GTTTTGTACGACGACAAAGTTTGGGTTGTTGAACTGCTCCCATGAAGTAGGAACAGCTAGGCCTAATTGTTTGTGGGCATTTGAAGCAGCCTTGATT
TTCTGGGATCTCCAGACTTTCAATGCTGCTGAATCCTCTGAAGTGGGAACACGAACGAATTTTGACAGGTATTCTACTTGTGATATATTCTTCGTTGTC
TCTATTTCCAGATGGATACCCACTGATTGACAATGATGCCGGAAAACTTCGACATCTTCAACAGTGTTAAGGCCATGTTTACCACCCGATTGCCAGAT
GGTGTCATCTGATGTATTGGCAAGGTCATTGTACTGGAAGAACTCCTTTGGAGCTCTGCCAGTGGTTTGACACCAAGCGTAAATCACACCTGCCTTA
AAGGCGTGTGTATTAACGTTTGATGTGTCTGACCCTCCAGTGCTTCCACCACGATTCTTCGCATGTACGTTTGACATCAGTTTGTAATTTGATCGTGAA
ACCGATAATATGTCTTGATACATGTCTTCAAAATTTGAAGGATCGTATGTGAATGTTTGATGTTCAAAGAATTTCTTGTCAACATCAGATAAGACACCA
TATTCGTATTTACCCATCCAGTTTGATCTGGATGGGCGGAGAGCAGGATCCCAGGTTACCACCGTCTTCCCTTCAGGTGGTTGAAGTTTTGCAACGTA
GTTCAAACGTTGTTCAGCAGGCAATCTGTTGTACTTGTCCCAGTCAATGAGATCTTCCAAACAGATTACTGTCTTTGGGGATCGTTGTCGTATGATTT
CGCGACTGTTTCTATCTTGGACGCAAACAGTCAACGAGTCATATTCCGGCTCGGTCACACCGATTATCCAAGCATTTTGTCTGGCTTGATACGAAGCA
TTTAGAACAGATGCGAGGTTCTTGCCGTTACCGCTTGGATGATCTTTATAGCCGAGTTCCCAAAGGTTTGCGTTTACTTCGAAGAGGAAAGGTTTGC
AAAACGAGTCGAAGGCGGTTGCATCAGCGATGATGTACCTGCCTCCTCTCGACTTCTGTGCAGCTGCCATTCTGTTGTATATTGCCTCCATTGACTGA
TTAAGAGGCATACCTATACCTGCACCATATGTATCCCAATTTATGCGTTTGTTGCGCTCGATTTGGAGACATTGGTCAATGTAGTAGGAGAACAGATCC
TGGCTGACCACAGTGCGAACATCTTTTCCTTTCCCACCTTCCTCGGGGCTCAAGCACTTCTTGATATCTACCACTTGTGATTTCACAAAAGCATGATA
GAATTGTACTGGATATTCACCAGTTTCCAAGTAATGCATTGCTCGTCGTTGCATGACGCGGTCATAACCAGCGTCGAACATCGCCTGACGAGACTTGA
AGCCGCCAGGCTTGATGAACGGGGTTCCTGGGGAATATTTTTGCTTCACATAATTGTGAACTGCCCTTGGCGGCAGAACATCACAATTAGCGAAGAC

TTCCGGGTATTGATCAAACATGGCTTGGGCTATTTCCTTAGCCATGGAACGATCCTCAGAGGTAAGTGCGGGGTACTTTGGCTCATAACGGTGCTGTG
ACTTGGCAATCAGCTCCGGTCTTTTATCGGCCAAGAATACTCCGTCAGCACCTTGTGCAACACCTTGTTCAAGGTATGATTGCACACGTGTCTCATAC
TCACGATCAGTCACAAACTCACCTGACTCGAAACCGAGCAGGCTGGCTTCAGATTGTGACATTAGAGGTTTTCCATAGCCTATAGGCCTCCTCTGAG
GCCCTCCAAGGTTTGATCGTTTGACAGCACCATATACCTGAGCGAGGTGTTTTCCCTCTTGCACCAATTTGTTGTAATCTGATTCAAAATCCTGCCTAC
CCACGAAATCAGAATAAGCAACATTGGCTGCAAGTTTGGCTTTGACACCAAGCAATCCAGTTCGATGAAGGCCAGTCAGGCCCCACACTGTTTTGA
CTCTTTTTGACGCCTCTAGACCCCACACATGGTCTAAGAAATCGCAAAGCCACTTGAACAGGCGATCGAACGCATGCTTGGAGTGCTCAGCTGCAA
GGTGCAGAAAGAAGGAAAGGATCTCCAGTATTGGCTGGACAAAGTCATATACGTTTTCACCGGCATTTTTGACGTATAACACAAACGCGTCTGCGAC
GCGGTTTGTGCGAACGAAAGAAATAGCATGGTGTAGACCATCCACCAGTTGCGCCCACTCATGGTGCACTCTTGGCGGTATGTTTGACACCCTGATC
AGAGAGTCTTCAGGGATGTCGACCTTGTCCAACTCCTTTTCTAAAGTCCGCTGTGAAACTTCGTCAAATGCCTCCTTATCTAAGGTGGGTATCCTGAG
TTGTTCCACGCATGCCTGGATAAACGCTACCTCATCAACCAACATACTGAAAGAATCTGGGTGGGCCAGATCAGTAGGTTCTGGTGTAGCTATCTGTG
GCATTGGTTCTGCCGCTTCTAACATCCCCTCTATTTCAAGCGGGATGTTTTCTAGGGACGCGGCGAAACCAAGCGAGCGATAAAAAGGGGTGTTGGC
AAAATCCTTGCCAGGAAAAAGAAAAGAAAGAACATCCTTGAGCAATTTTGGTGGTGCCAGTGCCAACCCTGTCAGAGCACCAACCATTATCATGCA
TGCTGTCCATGCAGTCGATCTCTTAGCACAAAGTTGCACCATCATGGTCACACAATTATGTGAAGATGAGTATGGTGCCACGTGTTGTGCTCTTACGA
GTGACTCCGCGTGGCCAACATTGAAAGGAGCCGGAAAAGACTTAAATCCGTGCTTGTACTCACGAGTGGTACTTACAAGACGAAAATTTCCTCCGA
TCGTTTCAGAGGGGCCGACAAATCTACCTTCATAGATCTTGCCGGTTTGATTGTCTTTCATGCTCCAATGCCCAAGGGGAAAAGGGAATGTCAGCTT
CTTCGTTCCCACGATCTTGTGCACTGGCTCCCAAACCAGCGAGGAACGTGTCTCCATAAGTGCCGCGAAATCTTGTGCTGCGCCCATCCACAAGTTT
TGCATTGACCAGATGCCCGCCACAAACGTGACTTTCGAATCTAGCAAGCATAACACAGGGTAGCGCCAAAAACCAAGCGCACAGATTTTGACTATG
GCGAACAATGACTTGGAAGAGAAAAGCATCCTCCAGATCACTGCTGGAACGGTAAAGAAAAGAATGAGGAAAAGTTCCCAATGGTTATATACATAT
GAACATATAGTATAGGCTTTCGCAAGTGTAACTAATGCGTCGTACGCATAGTTCCATTTTTGGGACCAATACCATTGTAACAACCACCATGCTCTCACC
TCAATGGGCGCGTCAACTTTGAAACCAGCAGACACCAACCAACCCATAAACTTGCCAATGGAGGGTTGTTTGAAATCCTTTGGTTCAGGCATCCTAT
GATACACTCTATCGAGTGTCTTGTCGTGGATAACCGGTCGTGCACCGCAGGCAATGGCGGTCTGCACAGTGCCGGCCCCCCCGTGGATGTGCACCGT
TTTGTAATCCTTCAATATTTCAAGATGATTACCCGATGGTATTTCGGGGCATCTCGACTTGATCTCGTCAGGGATCACTGAAACTGACGAGCTGCCGG
ATGTCCAACCTTCCTCATACTCTGCTGTGTTTTTAACTTTCTCCAATAAGTTTAACCCATCGTGAGAACGCGGGATGTCGCTTCCGGACAGCGCACCA
ATTCTGAAAGTTGGCACATACACAGCAGTGACCCTCGTGGCTATCCACGCAGGGAGAAAGTTGGACCAGGAAACCTTCGACCAATCGGAAATGAA
CCTTGTTGCTTCCACCCACTTTGAGCTTGGTGATAAAGTATAACTTACACCTTGAGACCTTTCCACCTCCACATGCGGAGTGAACAGTCTCTTGTAAC
CAAGTTGAGAAGCATACGCAAGATTCAGGTAACCAGCGGAACTAGATGAAAAGTCTCCGACTTTCATCTTTTCAAGATCGTCTGTTGTTACGTCTTT

ATACACATGGGAATGTGTACGGACACCATAGTGTGCCGCCACGTTCGCGTAATAATCAACAGGAACTTGGTCACCTCTAGTCCCTAACTTGCACACT
AGGAGGGAAGATGAGGCTGAAAAACTCTCATCCGCAACGGGATACTCTCCCCCTTCTGTGTGACCGTCAGGTCTTCGCCGCAGATTAGGCCGGGAA
AAGGAGAAATAAAACGTGCAAATGAGTTGGATGTAGAACCATATGTAAGCGGACACGAATACCTCATCGTGCCTCTCGTCAAAAGTTGATCCGCAG
AAAAGGGGATGATGATTGTGGTTTTCACCCTTGTCTTTGCAAACGGATCTTTGGTCTTTTCTCCAAGTTTTCTCAAAACCGTCGGCCATTTCACAGGC
CTGACGGTGAGTTGCACGAAAGGAAAATCGACTGCTGACAGGCAACGGCGCTTCCACCCTAGCGGTGTGAAGCTTTTCCGGCCTACCCGTGTGGAT
CATGCAATCATCGTGCAGATACCACCACATCCTGCCATCTGACTCAGGATACGTTCCTCTTGCAACGCCAAACCTCACTCTCTGATTTGACAAATCGA
ACTTGACAATCATTGAGGTGCCTACAGTAGGGCATGCCATTTTCGAACGATATATGCATGCCATGATGATCAAATGTTCAATCTTCATGACAGTTATAT
CGACCGCTTGGGCTGTTTGTCTGATCGATTCAAATGCATGAAATATGGCAATGTCCCTGCTGAAAGGCTCAACAGGGTCACTATGCGCATCCAATGC
AATCTGAGCCCTCTGTAGCTGCTCGTCTTTGTCAAGCTGCAAATGGAAATCCGATGTCCAATAATCCAACGTAGGCTGGCAGAACTCAGTCAAGTCA
AAAGAAACAATCTCGTCCCAGTACCTACATACTCGGGCACCGGGTCGAAATTGGTTGGACTGGTTGTGATAGGTGTGGAATGTAGAACCAGTGTGC
ACTAGTCCGACTCCATTTATACACCACCACATTTGACCTCGACAGCGGACACTTCCACTGCCTAAGTTCGAAAAGAACACAATGCGCTCATTGGCGA
AGTCAAAGTCAACAGCCATGGCTGACTCCGGACATGGGCATTGCATCCTTGATCTATAACACATGGCTATCGCGGCAACTTGCTCAAAATTGAACTG
GAGAGTTCTCATTTTGGGTATTGTGTCACGAATGGCTTGTTCAGCCACACCATGTGCTCTCGAATGCATGAATGCCGGTGTCGGCACATCATACAAAT
CTATTTGCTGCTGGAAACCGAAAAACAATTCCTGTTGACTCCTGAAAGTCAAATGTGAGGAATTGGACTTGGGCAGACCTTCCCCAGCAAAAACTT
CGCGCATGGGTATCGAATAATCTATGTTGAGATGCTCAGTGTAGAAAAACTGCCTGGGTTCCGGAGCATAAGATTTCTTCCCCCATGCACGAAAGG
AAAATCCCAGTGAAAGTACCACAACCGGTTCACCTCTGCGGAGTTCGGTTGTCGACACATATACAGCGAAGCCCCATGACTATCGAACTGTATAGCA
TAACCTACCCCGCTCTCAGGGCATTTCATCAGGGATTTACCACACAAGCAACGATTGCAGTATGTGATGGTGATAACACCTTGTCAGTGACACCATC
AAAGTATTGGTAAAGTGTGCGTTGCAATGTGTGTTCAGAGAATACAACGATCGGTTCACACCGGTCGAGGGCCTTTTGCCAGGTTGAGATCTTTGTG
AAGTATTCAGCGTCGAATGACCTGTCCACCCAACCCGATTTTGGAAGCGGCATCGTCCACAACTTGCTATAGTGATCCAGTGAAGGGGAACAAGAC
TTCCCATCTTCGATACCTAGCAGCCCGCCATGGACAATGACACTACCACCAATGCACCAATAAAGCTTCCCCTTATTTTTGTCTTCAGGGGAATCTGT
GGCGCAGAAAACAACCATCCTCCGGTCAAGTCTAAAATCAATTGCAAAAGAGCTGACATCTTTTGGACAATTGAACTTTGACTTGAAGCTCATTGCC
ATCGCTGCAACCAACACTGGATCTACTATCTTGACATCTGTAGACTCCAGGTATTGGTTGATGTGATTTTTGAGGTCGTCGACCTCAGAGCGGGAGA
AATGACCACCAGTAGGGATAATCCTGTCAACACATTTCTGAAACTGAGAAAGTGAGGGCATCGCGAACGGCCCTGGCTTCTCTTGTGATAACATAGA
GTCCGGTTTGGGCTCAATTATCACCTCATGTGTTGAATACTCCTTTGCCAGCCAACCGAATGAGATTGTGCTTGGAGAGGCAGGAGCCTTGTCCCCT
CCGTGCAAAATCTCATTCCCATTCAAGACCCACCACAACCTATTCTTGGCGATTTCGTCTGGGGACTCTGTCAGATGGAACAGTACAATTCCATCTTC
CGGTTTCACCTCCAGTGCATAACCGCATCCATTGTTTGGACAGGCGTATAGGGTCTTGAACGTGTGAGCTAAGGCAGACATGGTTGTACCGCCTACT

GGCTCAGAATCAGAGATCTTCTGGAAGAAGGAGTTTGTCTCATCACGCAACTTACTCACTAGCGCGGTGTTGAAAGGGTGGGAAGCCTTAACGACA
CAGTCAAGTGATTTTTGAAGCTCGGACAAAGAAGCAGCGGCAACCCACCTGATAAAGGTTGATGACCGCCACTTCTTGTCTTTAGGTAAATGAGAA
AGAACATAGTGGAATTCATAATGCTTTTCAACAAAAGATGAGTTTTTACCAGTGTACGGATACACCTCATCATGTTCTTTAAATCCAAAAATGTCCTTA
ACTTCTTCCGTTGCGCTTTTGCCATGGATGCCGATCTTCCCAAGAAACCTACGTGTTCTTGAAGGTGATCGCTCCGAAGTGACAGCGGCAACTGAAC
TCTGAGAACCACTCCGACCTCGAAACTGAGGCAAAGTGGGGTCAAGGGTTTCACATGGTCCGGGATTTTCTTCTATATCCGCGGTTCTGGAGTCTCG
AGGTTCCAGATGTTCCTCTGTTGGAGGCCATGCAAATGACGAGTAACCTACATTCCAATTCGACGTGTTCTCACCCGGTGAAGGTACAATCACGGTA
TGTCTGTTCATCCTGTGATTGACCCTCAAACGAGGTGGTGTCGGATGGTGCACAGTCAAGACATCACCAGGGGTCGGTGGTGTGAAATTCAATGAC
GGTGTTTCACCCTCCAAATCTGAAGTCGATTTAGAACGGAAATGCCTGATAGAAGGTCCGGGATTAGTTTCGATGTCCGATTTCGTCGAATCTCTCGC
GTCCTCATGATCATCATCAAAGAGATAATCAGAGGATGCTCGCGAAATAGACATCCATTCAGAAGAAGGTTTCTTCGACGGAGTGGGCTCAGAAAG
GGCTTTTCTATGAGAAACTTTCTCCGGTTTGAACTCGTTGGCACCCTTCAGATACGCCTCCCTCGCGTCAGGTCGGGAAGACGCTGTCGCACCCACT
TTCTTAGCCTTGGCTTGCAACTGCCTTCTAAAGGCGTCAGGATCAACACGTAAAGGTGTTGAACCTGATGCTTTGGAAGAGCTAGGAGGTGTGTATT
TTTGGGCGTTGACATCAAACTTCTCTGAGGGATCTGAGACAAGGGAAGATTGGGACCAAGATCGGGACCTGTATCCAACGTCACGGGATGCGTCTT
GATTCATAAAGGGCATGACCCTTTCGGACACTCTCAACCACCAATCATGTGGGAAGAGAGATCTGAAGCGAGCTCTATTTGACTTACCTTCAGAACC
TTCAGCAAGCAGCCACGTGTCACCATCAGCCAGTTCCACCTTGGGTCCCTTCTGCTTAACTCCCTGCACTGGTGGTACAGGCGGAGGAGGCGGAAC
AACTGTGGGAAGCCGAGGTATGGGCCTGGATGAAACCGGTTTCTCCACGTCAATGAAAGCTACTCTTGTTACCGTTTCCTTGTCCGGCTTGTGGTAG
GGTGATGACCCCAGAACCTCCAACACGACATCTGGTTTCGTACTATCATTGTGAAACTTCGAGAAGAAGAGCAGACCTTCATCATAGGTCAAACCA
ATCGCGGCAGCCAGAGCTATTCTCCATTTTTCTTGATTGTGTTTACGGAAGAATATTTTGGCCAGGCCCTTCGGTGTGAACACCAACCTACTTGCTCC
TGTATCCCAAAACAATCTCTCCTGCCTGGTGGTGATGAAACCACCCTCAACCCGAGGGTCAGGTCGTGTGAAATCAAGTCCGCGTTCGAAGACGCT
CAGACCGGAAAACTTGGCACAAACCCTGAAACCGTTCGTCAGCTGATGGAACAGGTTAGGTTTCAGACAAGGGACACTCACATGCGCAGAAGAAG
AGACTTTCTCGGCCAATCGCTGTTCGGCCGGACTACCATCCTGAATGACCAGTTTGGCAACCGCCTTCCAAATGTTCTTTTGGAATTGCAGTCTTTTA
ACTTGGTCAACCCCGCAATGGAATAGATCCTTTCCATCAAGACGAAACCACAATGCCGGCGGTAACTCGACTTGTTCATTGATAACGGTGAATGAGA
CTGAGTCACCCGCAAAAGATAAGACGCAAGTATTGCCAGGACATCCGAAATCCCGCTTCCATCTTTTAGCCATGGTCATTAAGGACCACAAGTCCAA
GTTGGTTCCATCCATCTCGGCGAGGAACCTTCCGAAGTATTCTTCGTATCTCGAACGGAACAAGTCGATGGTCAGGAACTCTGCCTCACCAGGATTT
GGTATCAGATTGAGTCTGGACTGTAGGACGGCACAGAGGTACTCATCCTTTAAGCCACAGTGCGCAATCTGATTACCATCAACACGGAAAGAAACA
CCGGACCCGGTCCTGTTCCTCGACACTTTCACCCTATTGTCAACCAGGTTGATGATAAAACCACTGGTAGGGCAAGAGAAAATCTTCCCAGCGTACC
TGGCCCGATGAATGAAATCCCTACGGACCACAGAGCGACGCGAAGAGGAACCGAAGAGGCCGCGAGCAAAAGAGACTACACCATCCAATGTCTCA

GGAATTTGACATCCGAGTACACGAAGAGGGTCTGCCACCAACGAAGACAATCGGAATTTCTGGCGTTTCACCTCGGCCAGTCTCAATGCCTTCTTTC
GTTCGGCATTTGACTGGCACCAAGCAGAAAACCGGCTGTTCTTCTCCCTCTTTGGCAAGGTGCGAATGCGCTTGCCACCAAGAAAAGAAGAACCG
AAAGCGACTTCTTCGGCGGAAGGGGTAGGGAGTACC
