# Supplementary material for: Characterization of the Mycovirome from the Plant-Pathogenic Fungus Cercospora beticola
Source: Viruses. 2021 Sep 24;13(10):1915. doi: 10.3390/v13101915 (PMC8537984; doi:10.3390/v13101915)
Supplement: Supplementary file 1 [file viruses-13-01915-s001.zip › viruses-1368835-supplementary Table S5.pdf]

Table S5. Pairwise alignments of sequences assigned to the same virus

Virus name	Representative contig sequence	Contig of subject sequence	Nucleotide identity (%)	aa identity (%)
CbHV1	contig1357	contig340	89.73	92.88
		k141-14799	89.97	98.54
		k141-5852	91.58	98.35
		contig1993	91.34	98.59
		k141-48216	86.48	97.01
		k141-31123	87.69	94.28
		k141-35317	95.33	99.97
		contig2265	94.71	98.4
		contig3558	88.41	98.4
		contig4473	77.47	93.87
		k141-56835	81.7	94.24
		k141-9569	97.41	99.24
		k141-13420	83.72	86.53
		contig2550	86.15	91.71
		contig3466	84.35	92.83
		k141-29364	87.39	92.51
		k141-6204	86.29	92.78
		k141-7467	85.88	93.48
CbOV1	contig965	k141-17237	96.41	98.18
		k141-36573	96.47	98.59
		k141-50990	96.7	98.59
		k141-30077	97.27	98.7
		k141-57947	97.36	98.7
CbNV1	k141-72534	k141-40198	99.27	100
		k141-3984	99.28	100
		k141-57522	98.19	100
-	k141-52449	k141-53721	95.72	99.7
		k141-44306	95.82	99.55
		k141-54540	99.78	99.7
		k141-68964	94.17	99.7
-	k141-27828	k141-41134	100	100
		k141-38712	100	100
		k141-3161	100	100
-	k141-21308	k141-31340	95.02	100
CbNSV3	k141-3378	k141-39151	97.69	99.03
		k141-21666	97.84	98.16
		k141-40192	97.06	100
		k141-25946	97.8	99.31
CbOV2	contig1024	k141-7251	100	100
