# Supplementary material for: Characterization of the Mycovirome from the Plant-Pathogenic Fungus Cercospora beticola
Source: Viruses. 2021 Sep 24;13(10):1915. doi: 10.3390/v13101915 (PMC8537984; doi:10.3390/v13101915)
Supplement: Supplementary file 1 [file viruses-13-01915-s001.zip › viruses-1368835-supplementary Table S6.pdf]

Table S6. Details of the 93 contigs obtained from 139 strains of *Cercospora beticola*

Number	Name of putative virus	Gene ID	Length (bp)	Best matched protein	Identity (%)	Sequence start	Sequence end	Genome type	Family/Genus	E-value
1	Cercospora beticola hypovirus 1	Cb_clean_Contig1357	12624	polyprotein [Wuhan insect virus 14]	52.9	1518	3818	(+)ssRNA	*Hypoviridae*	0.00E+00
2		Cb_clean_Contig3558	12361		54.8	1519	3644	(+)ssRNA		0.00E+00
3		Cb_clean_Contig4473	10768		58.3	1901	3530	(+)ssRNA		0.00E+00
4		Cb_clean_Contig340	9796		50.2	1564	2698	(+)ssRNA		0.00E+00
5		Cb_clean_Contig2265	9611		53.8	1518	3359	(+)ssRNA		0.00E+00
6		Cb_clean_Contig2550	4313		45.8	1564	2321	(+)ssRNA		0.00E+00
7		Cb_clean_Contig1993	1180		59.2	3299	3591	(+)ssRNA		1.00E-103
8		Cb_clean_Contig2381	1146		49.4	3490	3818	(+)ssRNA		9.00E-100
9		Cb_clean_Contig3466	1112		49.5	760	899	(+)ssRNA		1.00E-09
10		Cb_clean_Contig1397	807		28.2	3666	3799	(+)ssRNA		1.00E-10
11		Cb_clean_Contig2817	579		44.6	1162	1269	(+)ssRNA		6.00E-16
12		Cb_clean_Contig1712	159		58.5	3994	4042	(+)ssRNA		1.00E-07
13		Cb_clean_k141_56835	12570		55.8	1519	3605	(+)ssRNA		0.00E+00
14		Cb_clean_k141_35317	12185		54.0	1518	3818	(+)ssRNA		0.00E+00
15		Cb_clean_k141_31123	11701		55.1	1519	3631	(+)ssRNA		0.00E+00
16		Cb_clean_k141_9569	7609		39.4	1519	2003	(+)ssRNA		8.00E-101
17		Cb_clean_k141_6204	5405		37.5	334	1269	(+)ssRNA		1.00E-83
18		Cb_clean_k141_5852	3885		57.4	2655	3818	(+)ssRNA		0.00E+00
19		Cb_clean_k141_29364	2513		43.6	1519	1897	(+)ssRNA		2.00E-86
20		Cb_clean_k141_7467	1996		38.22	2230	2919	(+)ssRNA		2.60E-10
21		Cb_clean_k141_14799	1914		62.7	2814	3451	(+)ssRNA		0.00E+00
22		Cb_clean_k141_13420	1861		46.7	1564	2003	(+)ssRNA		8.00E-113
23		Cb_clean_k141_48216	1569		48.4	3545	3818	(+)ssRNA		2.00E-77
24		Cb_clean_k141_49715	1457		50	3153	3244	(+)ssRNA		9.00E-17
25		Cb_clean_k141_63002	914		61.7	3347	3637	(+)ssRNA		2.00E-111
26		Cb_clean_k141_20910	695		66.7	3421	3465	(+)ssRNA		2.00E-16
27		Cb_clean_k141_49228	575		70.2	2318	2505	(+)ssRNA		9.00E-87
28		Cb_clean_k141_45450	573		40.4	3035	3072	(+)ssRNA		4.00E-11

29		Cb_clean_k141_41826	552		39.3	3630	3684	(+)ssRNA		3.00E-07
30		Cb_clean_k141_35224	510		65	3605	3644	(+)ssRNA		4.00E-08
31		Cb_clean_k141_55778	425		73.6	3545	3595	(+)ssRNA		6.00E-15
32		Cb_clean_k141_1313	415		60.2	3372	3464	(+)ssRNA		3.00E-29
33		Cb_clean_k141_13671	359		44.9	3022	3053	(+)ssRNA		6.00E-07
34	-	Cb_clean_k141_17023	382	RNA dependent RNA polymerase [Plasmopara viticola lesion associated ourmia-like virus 22]	62	70	119	(+)ssRNA	*Botourmiaviridae*	3.00E-09
35	Cercospora beticola ourmia-like virus 2	Cb_clean_Contig1024	1440	RNA dependent RNA polymerase [Plasmopara viticola lesion associated ourmia-like virus 42]	78.6	202	615	(+)ssRNA		0.00E+00
36		Cb_clean_k141_7251	1440		81.2	202	615	(+)ssRNA		0.00E+00
37	Cercospora beticola ourmia-like virus 1	Cb_clean_Contig965	2273	RNA dependent RNA polymerase [Erysiphe necator associated ourmia-like virus 10]	66.8	23	600	(+)ssRNA		0.00E+00
38		Cb_clean_k141_30077	2276		67.9	23	600	(+)ssRNA		0.00E+00
39		Cb_clean_k141_57947	2237		67.9	23	600	(+)ssRNA		0.00E+00
40		Cb_clean_k141_50990	1424		71.7	266	600	(+)ssRNA		1.00E-160
41		Cb_clean_k141_36573	1359		72.4	287	600	(+)ssRNA		1.00E-151
42		Cb_clean_k141_17237	1199		73.1	333	600	(+)ssRNA		3.00E-131
43		Cb_clean_k141_57307	542		67.9	76	248	(+)ssRNA		5.00E-58
44		Cb_clean_k141_51878	362		68.1	224	341	(+)ssRNA		1.00E-41
45		Cb_clean_k141_18635	338		66.5	157	268	(+)ssRNA		2.00E-38
46	Cercospora beticola ourmia-like virus 3	Cb_clean_k141_5161	2108	RNA dependent RNA polymerase [Soybean thrips ourmia-like virus 1]	81.1	43	625	(+)ssRNA		0.00E+00
47	Cercospora beticola narnavirus 1	Cb_clean_k141_40198	4127	RNA dependent RNA polymerase [Erysiphe necator associated narnavirus 13]	49.7	1	810	(+)ssRNA	*Narnaviridae*	0.00E+00
48		Cb_clean_k141_3984	2527		49.7	1	810	(+)ssRNA		0.00E+00
49		Cb_clean_k141_72534	2487		75.2	1	810	(+)ssRNA		0.00E+00
50		Cb_clean_k141_57522	2324		49	46	810	(+)ssRNA		0.00E+00
51	-	Cb_clean_k141_15570	3450	hypothytical protein [Botrytis cinerea binarnavirus 2]	45.7	25	720	dsRNA	*Birnaviridae*	0.00E+00

52	Cercospora beticola metavirus 1	Cb_clean_k141_3161	1783	putative gag homologue [Cladosporium fulvum T-1 virus]	28.5	176	639	ssRNA-RT	*Metaviridae*	1.00E-35
53		Cb_clean_k141_38712	1830		28.5	176	639	ssRNA-RT		2.00E-35
54		Cb_clean_k141_27828	1964		28.5	176	639	ssRNA-RT		3.00E-35
55		Cb_clean_k141_41134	1234		28.2	176	639	ssRNA-RT		2.00E-34
56		Cb_clean_k141_21308	1671	env homologue [Cladosporium fulvum T-1 virus]	62.2	31	104	ssRNA-RT		2.00E-16
57		Cb_clean_k141_31340	1247		64.3	21	104	ssRNA-RT		6.00E-21
58		Cb_clean_k141_45057	437		31.8	39	164	ssRNA-RT		2.00E-06
59	-	Cb_clean_k141_54286	566	NS1 [Penicillium discovirus]	49.2	212	337	(-)ssRNA	-	1.00E-35
60		Cb_clean_k141_12535	386	NC [Penicillium discovirus]	61.9	66	191	(-)ssRNA		5.00E-48
61		Cb_clean_k141_33118	332		68.3	203	262	(-)ssRNA		3.00E-17
62	Cercospora beticola associated mycobunyavirales-like virus 1 segment L	Cb_clean_k141_6617	4197	RNA dependent RNA polymerase [Plasmopara viticola lesion associated mycobunyavirales-like virus 4]	69.4	1	366	(-)ssRNA		6.00E-173
63		Cb_clean_k141_67281	2314		46.7	362	1106	(-)ssRNA		0.00E+00
64		Cb_clean_k141_23000	405		55.2	734	858	(-)ssRNA		2.00E-38
65		Cb_clean_k141_13335	320		77.27	1	43	(-)ssRNA		2.00E-13
66	Cercospora beticola associated mycobunyavirales-like virus 1 segment S	Cb_clean_k141_13474	1134	NP [Plasmopara viticola lesion associated mycobunyavirales-like virus 4]	56.6	1	281	(-)ssRNA		2.00E-108
67		Cb_clean_k141_47347	855		62.5	28	285	(-)ssRNA		1.00E-113
68		Cb_clean_k141_14865	488		67.6	148	285	(-)ssRNA		2.00E-63
69		Cb_clean_k141_25120	615		61.6	1	133	(-)ssRNA		5.00E-44
70		Cb_clean_k141_67601	1854	NS [Plasmopara viticola lesion associated mycobunyavirales-like virus 4]	44.2	8	468	(-)ssRNA		2.00E-125
71		Cb_clean_k141_12481	346		53.5	368	481	(-)ssRNA		2.00E-37
72	Cercospora beticola negative-stranded virus 3	Cb_clean_k141_3378	6191	RNA dependent RNA polymerase [Plasmopara viticola lesion associated mononegaambi virus 5]	61.2	1	1954	(-)ssRNA		0.00E+00
73		Cb_clean_k141_25946	3186		65	1	1011	(-)ssRNA		0.00E+00
74		Cb_clean_k141_39151	2992		51	1011	1954	(-)ssRNA		0.00E+00
75		Cb_clean_k141_21666	1389		46.2	1336	1797	(-)ssRNA		2.00E-138
76		Cb_clean_k141_40192	1259		64.9	890	1308	(-)ssRNA		2.00E-174
77		Cb_clean_k141_39090	1035		57.7	1597	1941	(-)ssRNA		2.00E-117
78		Cb_clean_k141_54717	673		37.7	1507	1726	(-)ssRNA		5.00E-47
79		Cb_clean_k141_2670	672		58.2	1275	1467	(-)ssRNA		1.00E-73

80		Cb_clean_k141_57732	533		58.1	1360	1536	(-)ssRNA		5.00E-62
81		Cb_clean_k141_5086	424		59.7	952	1072	(-)ssRNA		5.00E-43
82		Cb_clean_k141_32680	422		59.7	952	1072	(-)ssRNA		5.00E-43
83		Cb_clean_k141_64068	417		72.2	685	821	(-)ssRNA		3.00E-77
84		Cb_clean_k141_40049	408		80.6	482	617	(-)ssRNA		4.00E-72
85		Cb_clean_k141_44574	382		62	848	974	(-)ssRNA		2.00E-55
86		Cb_clean_k141_11227	350		53.3	1	101	(-)ssRNA		1.00E-23
87		Cb_clean_k141_36621	314		74.84	486	589	(-)ssRNA		2.00E-48
88		Cb_clean_k141_33467	308		69.3	291	391	(-)ssRNA		3.00E-40
89	Cercospora beticola negative-stranded virus 1	Cb_clean_Contig154	6604	RNA dependent RNA polymerase [Aspergillus fumigatus negative-stranded RNA virus 1]	57.3	3	2122	(-)ssRNA		0.00E+00
90	Cercospora beticola associated mycobunyavirales-like virus 2	Cb_clean_k141_63059	6457	RNA dependent RNA polymerase [Plasmopara viticola lesion associated mycobunyavirales-like virus 10]	63.6	1	1827	(-)ssRNA		0.00E+00
91		Cb_clean_k141_26880	499		53.4	266	430	(-)ssRNA		4.00E-46
92		Cb_clean_k141_15719	440		80.8	786	931	(-)ssRNA		1.00E-70
93		Cb_clean_k141_61155	310		85.3	1045	1146	(-)ssRNA		1.00E-52
